# Substituent Effects from the Point of View of Energetics and Molecular Geometry in Acene, Polyene, and Polyyne Derivatives

**DOI:** 10.1021/acs.joc.2c02936

**Published:** 2023-06-02

**Authors:** Mozhgan Shahamirian, Paweł A. Wieczorkiewicz, Tadeusz M. Krygowski, Halina Szatylowicz

**Affiliations:** † Department of Chemistry, Faculty of Science, Islamic Azad University, Sarvestan Branch, Sarvestan 73451-173, Iran; ‡ Faculty of Chemistry, Warsaw University of Technology, Noakowskiego 3, 00-664 Warsaw, Poland; § Faculty of Chemistry, University of Warsaw, Pasteura 1, 02-093 Warsaw, Poland

## Abstract

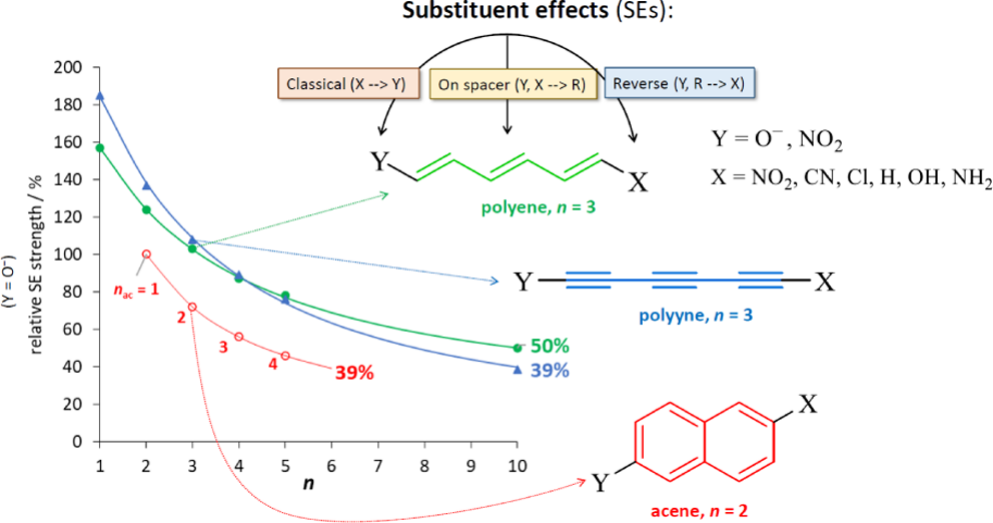

The substituent effect (SE) is one of the most important topics in organic chemistry and related fields, and Hammett constants (σ) are commonly used to describe it. The results of the computational studies carried out for Y–R–X systems (reaction sites Y = NO_2_, O^–^; substituents X = NO_2_, CN, Cl, H, OH, NH_2_; spacers R = polyene, polyyne, acene with *n* = 1–5 repeatable units) show that the substituent properties depend significantly on *n*, the type of R, and Y. Results of the analysis of the substituent effect stabilization energy and geometrical parameters of the Y–R–X systems reveal that (i) the SE strength and its inductive and resonance components decay with the increase in spacer length, its weakening depends on the Y and R type; quantitative relations describing decay are presented; (ii) the ratio between inductive and resonance effect strength changes with *n* and depends on Y; (iii) differences in the substituents’ properties are examples of reverse SE; (iv) in general, structural parameters are mutually well correlated as well as with the SE descriptors; (v) due to the strong O^–^ resonance effect, the changes in π-electron delocalization within R are well correlated with the SE strength only for Y = O^–^ systems.

## Introduction

The substituent effect (SE) is one of the most important and most frequently used terms in organic chemistry. The first quantitative approach in describing the SE was proposed by Hammett in 1937,^1^ but its full presentation was given in his fundamental monograph 3 years later.^2^ Hammett introduced as a quantitative characteristic of the SE, the substituent constant termed σ, defined as the difference between the logarithms of the ionization constant of substituted and unsubstituted benzoic acid. The basic assumption of Hammett’s concept was that changes in the physicochemical properties of other substituted Y–R–X systems (X is a substituent, R is a transmitting moiety, and Y denotes a functional group, on which the physicochemical process takes place, often called the reaction site) would be similar to those observed in benzoic acids. Thus, these properties, *P*(*X*), should be correlated with the substituent constants σ(*X*) in the form of eq 1, called the Hammett equation
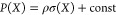
1where ρ, termed the reaction constant, describes the sensitivity of the property *P* to the SE in given conditions (e.g., solvation, temperature). Equation 1 describes the so-called classical SEs, that is, the effect of X on Y.

The properties of X depend both on the position in R and on the nature of the group Y. Effect of R–Y on X is, in turn, termed the reverse SE. The effect of R is represented by different substituent constants for *para* and *meta* positions (σ_p_ and σ_m_)^1^ whereas that of Y can be illustrated by the substituent constants σ^+^ and σ^–^, which describe SEs in molecules with positively and negatively charged reaction sites, respectively. Swain and Lupton,^3^ 30 years after Hammett’s publication,^1^ mentioned 20 different substituent constant scales. Moreover, they showed that the SE can be decomposed into inductive (or field, *F*) and resonance (*R*) contributions, according to eq 2.
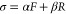
2Here, α and β are sensitivities or weighting factors, different for each set of substituent constants.

While the σ constants are derived experimentally, there are several measures of the SE, which are calculated using quantum chemistry methods.^4−6^ Importantly, unlike the σ constants, these measures are not based on the approximation that the SE in a system of interest is similar to that in benzene derivatives (R = benzene). Most important measures of this type are the substituent effect stabilization energy (SESE),^4,7,8^ charge of the substituent active region (cSAR),^6^ and the electrostatic potential calculated at certain positions (*V*).^5,9,10^ In short, SESE is the energy that can be attributed to the interaction between X and Y and is calculated using homodesmotic reactions, while cSAR and *V* are based on the distribution of electrons within the molecule. It should be mentioned that SESE and σ describe the SE for the entire molecule (globally), whereas cSAR and *V* describe it locally, that is, they describe the properties of a certain molecular fragment.

Polyene, polyyne, and acene derivatives are very important because they are the precursors of various interesting compounds with diverse applications. Functionalized polyenes, namely, halogen derivatives of polyacetylenes, were the first conductive organic polymers to be synthesized.^11^ This discovery, which was awarded the 2000 Nobel Prize in Chemistry, paved the way for the modern chemistry of organic semiconductors. Polyacetylenes have not found applications in electronic industry due to their poor stability in air and hard processing (lack of solubility). However, functionalization enhances their properties, and some interest in them is still being shown.^12^ For example, in 2022, functionalization of polyacetylene with −BR_2_, NR_2_, PR_2_, and POR_2_ groups was reported, which increased its solubility and resulted in interesting light absorption properties in the visible and near-IR region.^13^ Polyynes have drawn the attention of biochemists due to their natural occurrence^14^ and the attention of material scientists^15^ and organic chemists since they are precursors of various compounds.^16,17^ Regarding the latter, 1,3-diynes have found many uses in the organic synthesis of heterocycles, for example, 3,5-disubstituted pyrazoles^18^ and 2,5-disubstituted furans,^19^ and several protocols for their synthesis have been developed.^20,21^ In materials chemistry, polyynes are potentially useful as conducting molecular wires^22^ and have interesting optical properties.^23,24^ Due to their shape and reactivity, polyynes may find another application in organic electronics in self-assembly-driven functionalization of carbon nanosheets.^25^ Therefore, much effort is being made to develop synthesis methods and enhance their stability.^26,27^ In 2022, polyynes with up to 14 triple bonds were shown to be stable after functionalization with bulky endgroups and protecting the chain with two 2,6-pyridylcycloparaphenylene nanohoops.^28^ Interestingly, polyynes consisting of up to 8 carbon atoms are linear, but longer ones are slightly bent, which has been observed using atomic force microscopy^27^ as well as in crystallographic measurements.^23,29−31^ Moreover, in 2019, cyclo[18]carbon, a synthetic C_18_ carbon allotrope, was synthesized, and it was shown that it has a structure of cyclic polyyne with alternating single and triple bonds (*D*_9*h*_ symmetry).^32^ This structure is highly reactive and stable only in temperatures of several kelvins, but such molecules may be useful in on-surface synthesis of larger carbon-based nanostructures.^33,34^ Acenes arouse some interest in the field of astrophysics as such compounds may form in the interstellar medium and be responsible for absorption bands observed in the visible and IR regions in spectra of distant stars and galaxies.^35^ Additionally, due to the small HOMO–LUMO gap, acenes and their heterocyclic analogues are studied in the field of organic electronics as semiconductors and parts of light-emitting devices.^36,37^ A recent example of acene-derived compounds is helicenes, which exhibit interesting chiral optical properties.^38^

Regarding the SEs in polyenes, polyynes and acenes, some interesting results were presented by Divya et al.^39^ In this case, the research objects were Y–R–X systems, where Y = −CH=CH_2_, X = NH_2_, OH, CH_3_, H, F, Cl, CF_3_, CHO, CN, NO_2_, and R = alkyl, polyene, polyyne, polyphenyl, polythiophenyl, up to three repeatable units, or acene, from naphthalene to tetracene. The property (*P*(*X*) from eq 1) for which SE was studied was the molecular electrostatic potential at its minimum (*V*_min_) above the double bond of the reaction site (Y = −CH=CH_2_). For all series of X derivatives of each spacer (R) of particular length (*n*), Hammett plots (eq 1) of *V*_min_ against σ_p_(*X*) were created. The series of decreasing SE strengths for R was determined as polyene > polyyne > polythiophenyl > polyphenyl > acene. The SE weakens with an increase in the length of R (from *n* = 1–3); the largest weakening was observed in R = phenyl systems, where three phenyl rings are connected by single bonds, and therefore only inductive SE is possible. A strong transmitting property of polyyne linkers was also observed by Fonseca Guerra’s group; in this case, the SE of O^–^, OH, and OH_2_^+^, through polyyne linkers (*n* = 1–10), on the hydrogen bonds of the guanine–cytosine base pair was considered.^40^ Another interesting theoretical research on this topic was published by Sadlej-Sosnowska,^41^ where disubstituted polyenes (Y = phenyl and X = 15 substituents of different character) were studied. It was found that the strength of SE decays with *Ar*^–2^, where *r* is the distance between the C–X carbon atom and the most distant carbon atom of Y. Moreover, through-bond and through-space contributions to the SE were evaluated using models in which polyene linkers were removed. Further study by Sadlej-Sosnowska concerned the geometry, HOMO–LUMO gap, and polarizability in disubstituted polyynes and cumulenes.^42^ The main conclusion was that some combinations of X and Y groups cause higher changes in polarizability and the HOMO–LUMO gap than others. Hence, it is possible to tune optical and electric properties of polyynes and cumulenes by substitution. In a study by Varkey et al., mono- and disubstituted polyacetylenes, polyynes, and polythiophenes (up to *n* = 24) were investigated by means of geometric and molecular properties such as bond length alternation (BLA), rotational barrier heights, polarizabilities, and chemical shifts.^43^ It was shown that regardless of the type of X and Y groups, the SE on all evaluated properties decays exponentially with an increase of *n*. Additionally, cooperative effects of X and Y groups depending on their electronic properties were discussed. In this case, the cooperative effect for the combination of electron-donating and -withdrawing groups was stronger than for the two groups with similar properties.

**Scheme 1:**
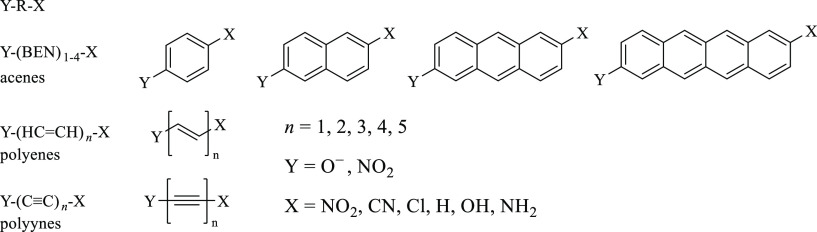
Studied Y–R–X Systems The carbon chain numbering throughout this paper is from Y to X.

The idea behind this work is to compare the SE in Y–R–X systems, transmitted through different spacers (R)—linear acenes, polyenes, or polyynes of different length. All combinations of the following Y and X groups were considered: Y = O^–^, NO_2_, and X = NO_2_, CN, Cl, H, OH, NH_2_. Studied systems are shown in Scheme 1.

Such choice of systems allows one to compare how the size of the transmitting moiety (1–5 repeatable units *n*, so 2–10 C atoms between Y and X) and the type of bonding (double, triple, aromatic) affect the SEs. When comparing the acenes with other systems, it should be remembered that the *n* = 1 systems (benzene derivatives) have the same amount of CC bonds between Y and X groups as *n* = 2 polyene (butadiene) and polyyne (butadiyne) derivatives. The substituent constants of all considered Y and X groups, as well as Y groups studied by Divya^39^ and Sadlej-Sosnowska,^41^ are summarized in Table S1 (Supporting Information). The Y and X groups have been characterized by structural parameters (bond lengths and valence angles) and the SE by SESE and σ_p_ constants. The spacers were described by the structural parameters (CC bond lengths), BLA index,^44,45^ and the geometric harmonic oscillator model of aromaticity (HOMA) index.^46^

## Results and Discussion

Nowadays, one of the most widely available molecular data is the structural data. It can be easily obtained, through either the crystallographic databases or theoretical calculations. The question then arises how the geometric parameters change due to the SE, and how these changes depend on the spacer and the functional groups involved. Describing quantitatively the relationships between these factors would facilitate the design of molecules with the desired properties. In order to answer the above questions, calculations were performed for systems presented in Scheme 1. The first two sections discuss the reverse SE evaluated by SESE and geometric parameters, the next one, the classical SE from the geometric point of view, and the last one, the effect on spacers. Computational methods used are explained at the end of the paper. The obtained SESE values are presented in Table S2, while the structural parameters for all considered derivatives are collected in Tables S7–S12 (Supporting Information).

### How Does the Spacer Affect the SE and Its Nature?

Let us start by discussing the relationships between the two global SE descriptors, SESE and σ. The statistical data on these dependences for all studied systems are presented in Table 1. Excluding the decapentayne series, the obtained determination coefficients are greater than 0.94. This confirms that the SESE concept can be used to describe the SEs. In the case of Y = O^–^ systems, σ_p_^–^ correlates with SESE better than σ_p_ (Table S13), which results from the negatively charged reaction site.

**Table 1:** Statistical Data on the Correlation of SESE = *a*σ_p_(^−^) + *b* in Y–R–X Systems (Y = NO_2_, O^–^ and X = NO_2_, CN, Cl, H, OH, NH_2_), Where *n* is the Number of Repeatable Units in the Transmitter R (Scheme 1)^a^

		Y = NO_2_ SESE = *a*σ_p_ + *b*				Y = O^–^ SESE = *a*σ_p_^–^ + *b*			
R	*n*	*a* ± se	*r*^2^	%_shortest_	%_benz_	*a* ± se	*r*^2^	%_shortest_	%_benz_
polyene	1	–9.25 ± 0.96	0.959	100	193	30.14 ± 1.98	0.983	100	157
	2	–6.77 ± 0.45	0.983	73	141	23.84 ± 2.34	0.963	79	124
	3	–4.99 ± 0.30	0.986	54	104	19.67 ± 2.17	0.953	65	103
	4	–3.93 ± 0.23	0.987	42	82	16.72 ± 1.98	0.947	55	87
	5	–3.18 ± 0.19	0.987	34	66	15.01 ± 1.57	0.958	50	78
polyyne	1	–8.35 ± 0.87	0.958	100	174	35.39 ± 3.49	0.963	100	185
	2	–5.86 ± 0.74	0.940	70	122	26.18 ± 2.51	0.964	74	137
	3	–4.50 ± 0.47	0.957	54	94	20.69 ± 2.16	0.958	58	108
	4	–3.62 ± 0.33	0.968	43	75	17.09 ± 1.85	0.955	48	89
	5	–2.61 ± 0.65	0.799	31	54	14.54 ± 1.59	0.955	41	76
acene	1	–4.80 ± 0.14	0.997	100	100	19.17 ± 1.75	0.968	100	100
	2	–3.20 ± 0.15	0.992	67	67	13.82 ± 1.52	0.954	72	72
	3	–2.15 ± 0.10	0.992	45	45	10.80 ± 1.26	0.948	56	56
	4	–1.60 ± 0.08	0.989	33	33	8.82 ± 1.08	0.944	46	46

a Slopes *a*, their standard error (se), and determination coefficients *r*^2^, %_shortest_ and %_benz_ indicate how the slopes relate to *n* = 1 and the benzene system (in %).

The slopes of the obtained relations (reaction constants) indicate the SE strength of X groups in Y–R–X systems with fixed R and Y groups. Observed changes in the strength of the SE with regard to the Y and R spacer length are examples of the reverse SE. Note that the slopes of linear equations for NO_2_–R–X and O^–^–R–X derivatives (Table 1) have the opposite sign. This results from the different nature of the Y = NO_2_ and O^–^ groups, which are electron-attracting and electron-donating, respectively. The absolute values of the slopes indicate stronger intramolecular interactions for the Y = O^–^ than the Y = NO_2_ systems. Moreover, their absolute values decrease with the increase in number of repeatable units (*n*) in the spacer R. Similar behavior has been observed in previous studies using various properties on the *y* axis.^39,41,43^ The percentages in Table 1 indicate the strength of the SE relative to the shortest (*n* = 1) system (%_shortest_) for each R or to the *para* benzene derivative (%_benz_). From these values, it can be noticed that the SE diminishes quicker with an increase of *n* in Y = NO_2_ than in Y = O^–^ systems. Comparing the shortest and the longest spacers for a given R-type, the decrease in the strength of the SE is approximately threefold in Y = NO_2_ systems and twofold in Y = O^–^ systems. The greatest decrease is observed in the case of acene derivatives. Interestingly, as indicated by the %_benz_ values, in Y = O^–^ systems with *n* ≤ 4, the polyyne chain is a better SE transmitter than the polyene chain, which is the opposite case to Y = NO_2_. Values in Table 1 can be compared to the other results in the literature. Divya et al.,^39^ for the Y = CH_2_ = CH– reaction site, obtained a 62% decay of the SE between *n* = 1 and 3 in polyenes and 58% in polyynes, whereas in acenes, for *n* between 2 and 4, 59% (our values are 49% for Y = NO_2_ and 64% for Y = O^–^). From data presented by Sadlej-Sosnowska,^41^ for Y = C_6_H_5_–, R = polyene, we can obtain a value of 33.8% (33.7% when cSAR is used instead of σ) between *n* = 2 and 6 in polyenes; our ratios between the extrapolated value for *n* = 6 and that calculated for *n* = 2 are 34.5 and 50.6% for Y = NO_2_ and O^–^, respectively. The differences clearly come from the difference in the nature of Y groups (Table S1).

**Figure 1:**
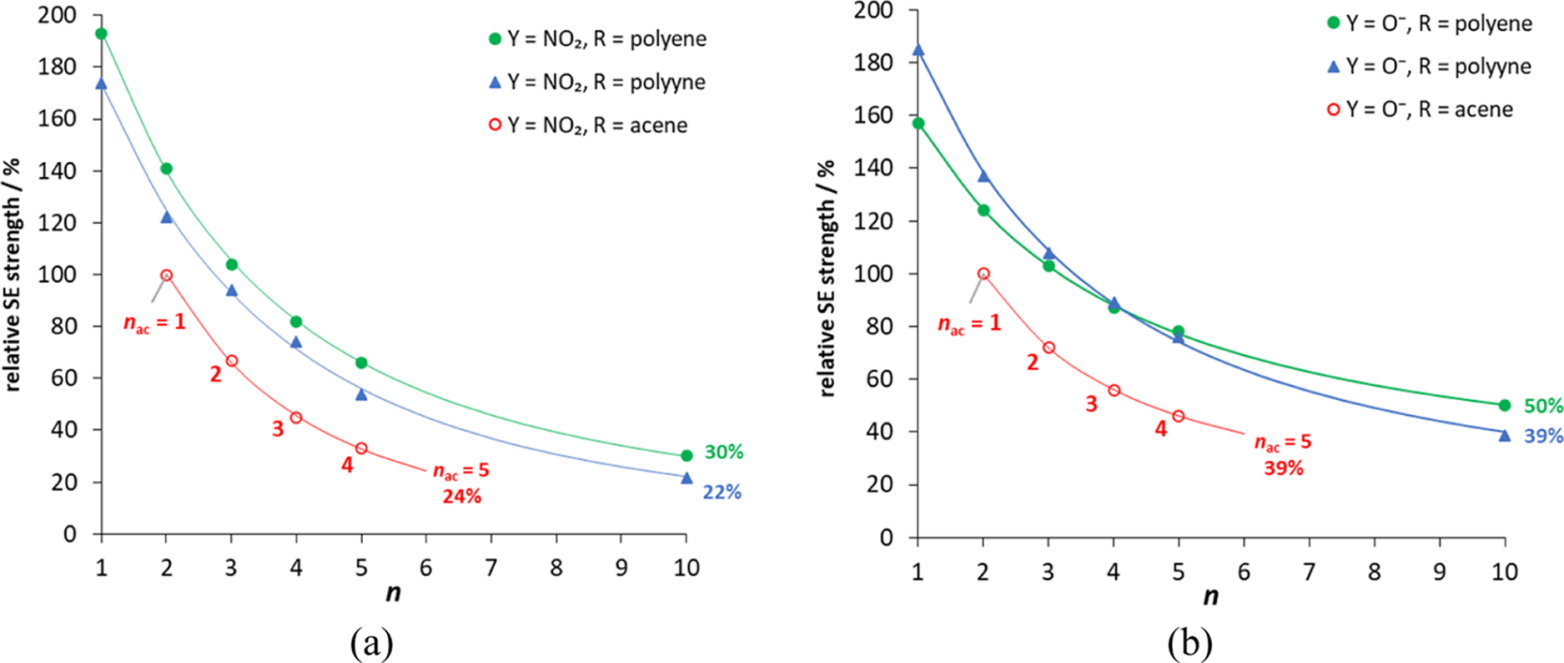
Strength of the SE in Y = NO_2_ (a) and O^–^ (b) systems relative to the appropriate benzene derivative as a function of repeatable units in spacer R. Values of *n* for acenes, *n*_ac_, start at *n* = 2 in order to compare R systems with the same number of bonds between Y and X.

Figure 1 illustrates the relative SE strength (%_benz_ from Table 1) as a function of *n*. We can see how changes in R and Y modify the strength and decay of the SE. Additional calculations for *n* = 10 polyene and polyyne systems were performed in order to improve the fitting (Table S2). The decrease in strength of the SE with *n* (in range 1–10) can be approximated by exponential functions [*y* = *a*·exp(*b*/(*n* + *c*))], for which the fitting parameters are presented in Table S3 (other types of fit, such as *y* vs 1/*n* linearization, do not correctly fit our data, see Figure S1). For both Y groups, the weakest effects are observed in acene derivatives, which can be explained by the high resistance of the aromatic π-electron structure of acenes to the SE, as previously reported.^47^ In addition, only acenes up to *n* = 5 (extrapolated value) are shown in Figure 1 because for *n* ≥ 6, their ground state changes to a diradical open-shell singlet state.^48^ A crossover between polyynes and polyenes in Y = O^–^ near *n* = 4 can be noticed. This can be explained by the different strengths of inductive effects discussed below. In addition, the dependences of the range of SESE variability on the length of the R spacer for both studied series (shown in Figure S2) also confirm the above observations.

Taking into account the field and resonance contributions to σ (eq 2) and the fact that SESE and σ are well correlated, one can write
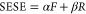
3

The use of this two-parameter regression makes it possible to estimate both contributions in the studied systems. The statistical data on these regressions are summarized in Table 2 (details in Tables S4 and S5). As before, except for the decapentayne (*n* = 5) series, the obtained determination coefficients are greater than 0.91. The different nature of the reaction sites is again reflected in the opposite sign of the coefficients of eq 3, negative for the group Y = NO_2_ and positive for O^–^. In general, the absolute values of α and β decrease with an increase of *n*. Moreover, in all Y = NO_2_ polyenes and polyynes with *n* > 1, the ratio α/β is greater than 1, contrary to all Y = O^–^ systems where it is lower than 1. Thus, in Y = NO_2_ systems, the field (inductive) effect is dominant, and in Y = O^–^, the resonance effect. This is in agreement with the values of *F* and *R* constants for both reaction sites Y (Table S1). The differences between these groups and the fact that in polyynes inductive effects decay faster than in polyenes may be responsible for the crossover observed in Figure 1b.

**Table 2:** Statistical Data on SESE = α*F* + β*R* Correlations (Equation 3) in Y–R–X Systems, Y = NO_2_, X = NO_2_, CN, Cl, H, OH, NH_2_^a^

		Y = NO_2_				Y = O^–^			
R	*n*	α	β	*r*^2^	α/β	α	β	*r*^2^	α/β
polyene	1	–7.39	–10.63	0.945	0.70	27.97	45.91	0.943	0.61
	2	–7.06	–5.03	0.971	1.40	22.12	37.16	0.954	0.60
	3	–5.11	–3.91	0.978	1.31	18.85	29.98	0.956	0.63
	4	–3.87	–3.36	0.975	1.15	16.28	25.26	0.958	0.64
	5	–3.15	–2.73	0.980	1.15	15.08	21.66	0.954	0.70
polyyne	1	–9.32	–4.89	0.942	1.91	36.79	49.00	0.951	0.75
	2	–6.92	–2.87	0.955	2.41	27.37	34.57	0.925	0.79
	3	–5.08	–2.62	0.959	1.94	21.99	26.59	0.919	0.83
	4	–3.96	–2.37	0.969	1.67	18.55	21.62	0.927	0.86
	5	–2.89	–1.87	0.853	1.55	15.87	17.83	0.911	0.89
acene	1	–4.42	–4.72	0.991	0.94	16.80	31.46	0.957	0.53
	2	–2.80	–3.47	0.996	0.81	12.03	23.24	0.965	0.52
	3	–1.90	–2.31	0.995	0.82	9.41	18.20	0.966	0.52
	4	–1.41	–1.72	0.995	0.82	7.78	14.75	0.966	0.53

a α/β is the ratio between coefficients for field (α) and resonance (β) parameters.

In the case of nitro derivatives, the α/β ratio decreases with the elongation of the transmitter; for polyenes from 1.40 to 1.15 and for polyynes from 2.41 to 1.55. For analogous O^–^ derivatives, the α/β ratio increases with the spacer elongation; for polyenes from 0.60 to 0.70 and for polyynes from 0.79 to 0.89. For both Y, the ratios in polyynes are higher than in polyenes, which indicates a larger contribution of the inductive effect. This possibly results from the shorter distance between Y and X in polyynes as the CC bonds are shorter. Additionally, for both R, the inductive effect weakens with respect to resonance as *n* increases.

The resonance effect is also dominant in both Y = O^–^ and NO_2_ acene derivatives. However, it is stronger in O^–^ (α/β ≈ 0.5) than in the NO_2_ derivatives (α/β ≈ 0.8), again in agreement with the values of *F* and *R* constants. Interestingly, the ratio does not change while adding more rings, not counting the nitrobenzene derivatives. Moreover, in *para*-X-nitrobenzenes, the strength of both effects is almost similar (α/β = 0.94), while in *para*-X-phenolates, the resonance effect is about twice as strong as the field effect (α/β = 0.53).

### Reverse SE from the Point of View of Geometry

**Figure 2:**
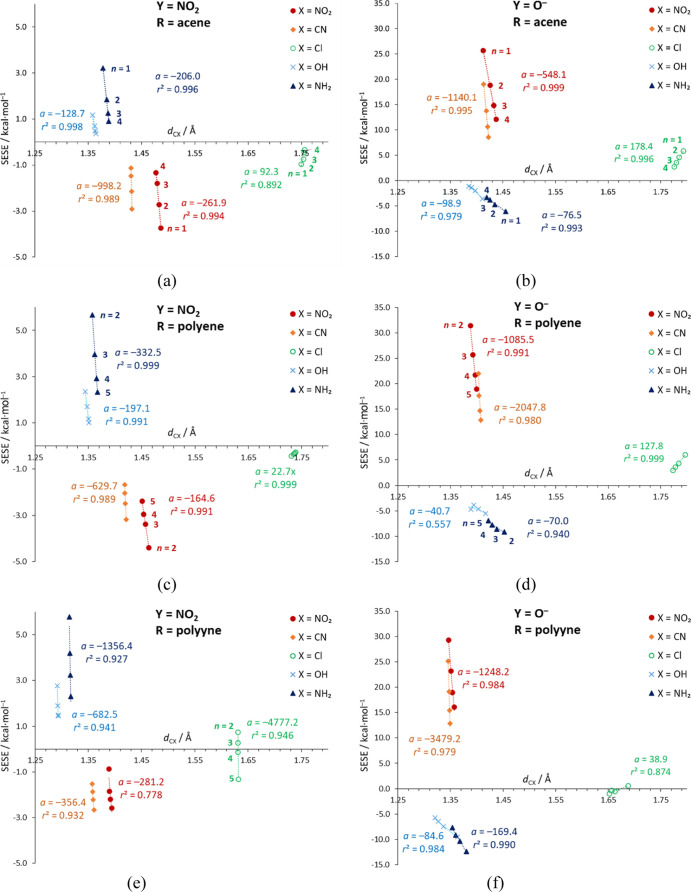
Relationships between SESE and the C–X bond length (*d*_CX_) for Y = NO_2_ and O^–^ acene (a,b), polyene (c,d), and polyyne (e,f) systems (*n* = 2–5, n = 1–4 for acenes). Values of *n*, slopes (*a*) and *r*^2^ of SESE = *ad*_CX_ + *b* correlation for each substituent X, are included.

The reverse SE is also revealed in the plots between SESE and the C–X bond length, *d*_CX_ (Figure 2, Tables S14 and S15). Here, each series consists of four systems with the same Y and X but varying in the length of the spacer (*n*); for easier comparison, all subfigures have the same ranges on the *x* axes. It should be noted that, in general, the data correlate very well. The systems in which the Y and X groups have opposite electronic properties are located in SESE > 0 part of the plots, and their C–X bond lengths increase with the increase of *n*. When both X and Y are electron-donating or -withdrawing, SESE < 0 and the C–X bond lengths increase as *n* decreases. In that regard, the chlorine substituent behaves like an electron-withdrawing group, with the exception of the Y = NO_2_, R = polyyne system (Figure 2e), where its π-electron-donating properties emerge, possibly due to the high resonance-transmitting abilities of the polyyne spacer. In all but the above case, the C–Cl bonds have the opposite sign of *a* (slope) compared to the other C–X bonds. All C–X bonds are the shortest in polyyne derivatives, which results from strong resonance between Y–R and X in these systems and no steric hindrance from hydrogens. Additionally, when comparing different R spacers, the polyyne derivatives have the highest absolute values of *a* coefficients, which indicates the lowest susceptibility of C–X bonds to the SE in these systems. Apart from the long and susceptible C–Cl bond, the lowest absolute values of the slopes occur in Y = O^–^ systems with electron-donating groups X = NH_2_ and OH. This results from the conflict between two electron-donating groups; elongating the spacer weakens the unfavorable interactions between them, which allows the C–X bond to shorten. Changes in bond lengths in % relative to monosubstituted systems are presented in Table S25.

**Figure 3:**
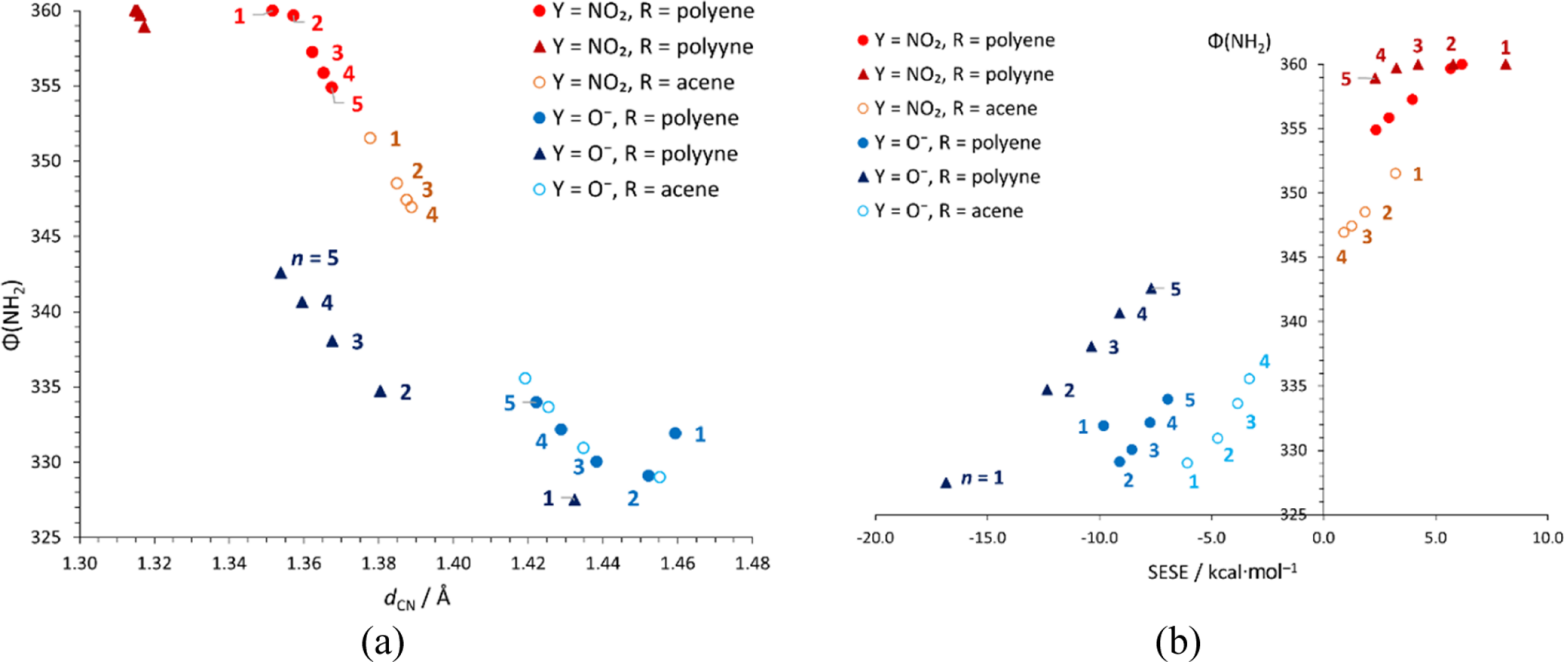
Pyramidalization of the amino group, Φ (NH_2_), in Y–R–X systems (X = NH_2_) plotted against C–NH_2_ bond length, *d*_CN_ (a) and SESE (b). Each series consists of *n* = 1–5 repeatable units within R (*n* = 1–4 for acenes). Digits near the markers indicate the value of *n*.

It is well known that the amino group can fluently change the geometry from tetrahedral, where the orbitals at the central N atom are sp^3^-hybridized to planar with sp^2^ hybridization.^49^ The geometry of NH_2_ depends on the intra- and intermolecular interactions, for example, strong coupling with the substituted system increases planarity. Therefore, it is worth looking at the pyramidalization of the X = NH_2_ group in systems with different Y and R moieties; its changes are the manifestation of the reverse SE. It can be evaluated by the sum of valence angles at the N atom, Φ(NH_2_). A value of 360 indicates the planarity of NH_2_, whereas lower values indicate a more tetrahedral shape. In Figure 3a, it can be noticed that the points for double-bonded polyenes and acenes (circles) follow a single line [apart from some points at the lower Φ(NH_2_) values], while the triple-bonded polyynes (triangles) form a similar line at C–NH_2_ bond lengths (*d*_CN_), shorter by ∼0.04 Å. Along these two lines, Φ(NH_2_) correlates linearly with *d*_CN_ for the Φ(NH_2_) values between 359 and 329. The relation between Φ(NH_2_) and SESE (Figure 3b) further splits the points into three series for polyynes, polyenes, and acenes. In Y = NO_2_ systems, the amino group is closer to planarity, which is a consequence of strong resonance interactions between the electron-donating NH_2_ and -accepting NO_2_ groups. Additionally, Φ(NH_2_) decreases with an increase of *n*. In polyynes, NH_2_ is fully planar up to *n* = 3, in polyenes deviations from planarity start after *n* = 2, and in acenes, planar geometry is not observed. It follows that the resonance interactions between the NO_2_–R and the substituent X = NH_2_ are the strongest in polyynes, weaker in polyenes, and the weakest in acenes. Interestingly, in Y = O^–^ derivatives, the opposite is observed, that is, Φ(NH_2_) increases with an increase of *n*. Thus, the strong unfavorable interaction between the two electron-donating NH_2_ and O^–^ groups forces the tetrahedral shape of NH_2_ and increases the sp^3^ character of the N orbital. Increase in *n* weakens this interaction, which allows NH_2_ to adopt a more planar geometry and higher sp^2^ character of the N orbital. Similar to the case of Y = NO_2_, the highest planarity in Y = O^–^ derivatives is still observed in polyynes.

### Classical SE from the Point of View of Geometry

In order to describe the classical SE, we should look at the relationship between the properties of the fixed group Y and the substituents X. A suitable geometric property of Y that can be used is the C–Y bond length, *d*_CY_. As for the substituents X, they can be characterized by Hammett constants (Table 3), SESE (Table S6), or by *d*_CX_ (Figures S3 and Table S16). It should be emphasized that the first two descriptors were used for series with a specific Y and R, and the last one for systems differing in *n* (with Y and X fixed).

**Table 3:** Statistical Data on *d*_CY_ = *a*σ + *b* Correlations in Y–R–X Systems with a Given Number (*n*) of Repeatable Units in R for a Series of Substituents X = NO_2_, CN, Cl, H, OH, NH_2_

		Y = NO_2_*d*_CY_ = *a*σ_p_ + *b*				Y = O^–^*d*_CY_ = *a*σ_p_^–^ + *b*			
R	*n*	*a*·10^2^	±se·10^2^	%_shortest_	*r*^2^	*a*·10^2^	±se·10^2^	%_shortest_	*r*^2^
polyene	1	2.587	0.835	100	0.706	–1.864	0.125	100	0.982
	2	1.635	0.240	63	0.921	–1.321	0.055	71	0.993
	3	1.230	0.118	48	0.964	–1.009	0.075	54	0.978
	4	0.981	0.069	38	0.981	–0.813	0.072	44	0.970
	5	0.809	0.040	31	0.990	–0.685	0.062	37	0.968
polyyne	1	1.701	0.463	100	0.771	–1.890	0.434	100	0.826
	2	1.209	0.251	71	0.853	–1.311	0.163	69	0.941
	3	0.898	0.158	53	0.889	–1.000	0.080	53	0.975
	4	0.693	0.108	41	0.911	–0.774	0.061	41	0.976
	5	0.548	0.075	32	0.930	–0.625	0.052	33	0.973
acene	1	1.663	0.303	100	0.882	–1.162	0.047	100	0.994
	2	0.961	0.106	58	0.954	–0.810	0.073	70	0.969
	3	0.616	0.034	37	0.988	–0.600	0.055	52	0.967
	4	0.445	0.018	27	0.993	–0.483	0.052	42	0.956

The slopes of the linear equations with respect to the σ constants for the NO_2_–R–X and O^–^–R–X derivatives (Table 3) have the opposite sign (due to the different natures of the reaction sites). Their absolute values indicate, for all types of R, the weakening of the SE with the elongation of the spacer. This weakening (%_shortest_) is somewhat similar to that predicted by the SESE vs σ relations (Table 1) but generally slightly faster in all cases. The sequence of changes in the SE strength for different spacers R differs from that shown in Table 1 for *n* < 4. However, for *n* ≥ 4, the sequence is the same: polyene > polyyne > acene (acenes with the same number of CC bonds between Y and X). The differences may result from the steric effects, which affect the bond lengths and their variability depending on R.

In accordance with previous studies,^39,50^ the transmission power of the spacer (transmitting moiety) can be quantified by the transmission coefficient (γ). It is defined as γ = *a*/*a*_0_, where *a* is the reaction constant (ρ from eq 1) for a series of Y–R–X systems differing only by substituents X, and *a*_0_ is the same value for the reference series, where R = benzene. Values of *a* were taken from Table 3. The meaning of this parameter is similar to %_benz_ values discussed earlier (Table 1). The calculated values of γ as well as those obtained for the CH_2_ = CH–R–X derivatives by Divya and co-workers^39^ are shown in Table 4; the rows of these tables show systems with the same number of CC bonds between Y and X. Our γ values were calculated from the geometric data, whereas Divya used electrostatic potentials.

**Table 4:** Obtained Transmission Coefficients (γ) from Hammett’s Equations Using the Substituent Constant^a^

	Y = NO_2_*d*_CY_ = *a*σ_p_ + *b*			Y = O^–^*d*_CY_ = *a*σ_p_^–^ + *b*			Y = CH=CH_2_^39^*V*_min_ = *a*σ_p_ + *b*		
*n*/R	polyyne	polyene	acene	polyyne	polyene	acene	polyyne	polyene	acene
2	0.727	0.983	1.0	1.128	1.137	1.0	1.247	1.296	1.0
3	0.540	0.740	0.578	0.861	0.869	0.697	0.914	0.989	0.728
4	0.417	0.590	0.371	0.666	0.700	0.517			0.571
5	0.329	0.486	0.267	0.538	0.590	0.415			0.429

a Similar values calculated using SESE instead of σ_p_ are shown in Table S17.

For *n* = 2 systems with Y = O^–^ and CH=CH_2_ reaction sites, the transmission coefficients are greater than 1.0 (Table 4). This means that the transmission of the SE in butadiene and butadiyne spacers is stronger than in benzene. In the case of *n* = 2 nitro derivatives, the opposite is observed. In all *n* ≥ 3 systems, the transmission power is lower compared to that of benzene derivatives. In general, it decreases in the order polyene > polyyne > acene, so the sequence is similar to the one obtained from SESE vs σ relations (Table 1).

**Scheme 2:**
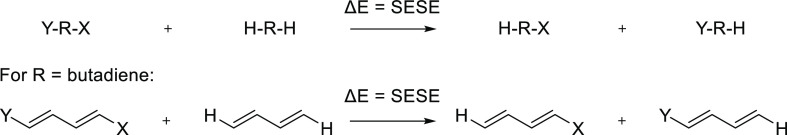
Homodesmotic Reaction for SESE Calculations

Using SESE instead of σ (*d*_CY_ = *a*SESE + *b*) reveals that all slopes are negative (Table S6), which means that in every Y–R–X series (fixed Y and R), *d*_CY_ decreases monotonically as interactions between Y and X become more stabilizing (SESE increases). The range of variation of SESE for the Y = O^–^ derivatives is higher than for Y = NO_2_ (Figures 2 and S2), while the variation of the bond lengths is similar, so the *a* coefficients are larger for the nitro derivatives (Table S6). In general, the slopes of the above Hammett equation indicate weakening of the SE with an increase in *n*, although the changes in *a* are small, especially in the Y = NO_2_ systems. Mostly, tendencies of the SE decay are preserved; only for the Y = NO_2_, R = polyene systems, the absolute values of the slopes slightly increase, but their changes are within the error limits. Taking the above into consideration, despite good correlations between *d*_CY_ and SESE for fixed Y, R, and *n*, such relations make it hard to compare the strengths of the SE between different Y, R, and *n*. Comparing the values of *a* for various *n* and R may not lead to a proper description of the decay of the SE with *n* and the differences in the transmitting power of spacers R. This possibly comes from the fact that the C–Y bond lengths depend not only on the interactions with the X group but also on the spacer R and its length *n*. For example, *d*_CY_ is much smaller in polyynes than in polyenes, while increasing *n* shortens the bond due to the increased π-conjugation in longer spacers. By its definition (Scheme 2, Computational Details), SESE only covers the energetics of the interaction between X and Y, omitting the conjugation between Y and R. Because the parameter on the *y* axis, *d*_CY_, does not omit this effect, unlike the parameter on the *x* axis, we observe the above. It should be mentioned that this also applies to the *d*_CY_ vs σ relations, which may be a reason for the discussed differences between data in Tables 1 (SESE vs σ) and 4 (*d*_CY_ vs σ), as well as S6 (*d*_CY_ vs SESE).

In Figure S3 are presented the dependences between *d*_CY_ and *d*_CX_ for given Y–R–X (Y, R, and X are fixed, *n* varies). In most cases, we see well-correlated series of data. However, a direct assessment of the SE decay based on the obtained slopes is not possible as the bonds between different atoms have different susceptibilities to change in length. Additionally, as discussed earlier (Table 2), the contribution of the resonance and inductive effects to the SE depends on the reaction site (Y), the spacer R, and also changes with its length *n*, especially in the Y = NO_2_ derivatives. This makes interpretation of slopes in these systems hard. However, regarding the Y = O^–^ derivatives, where the resonance effect dominates, some conclusions can be drawn. First, shortening of CO bonds with the extension of the transmitter is observed for all substituents. The same applies to the C–X bond for the substituents X = Cl, OH and NH_2_, but its elongation is observed for the NO_2_ and CN groups, resulting in the negative slope values shown in Figure S3b,d,f. These changes are in line with the resonance constants of the substituents (Table 1). The absolute values of the slopes for X = NO_2_, CN, and NH_2_ increase in the following order: acene < polyene < polyyne, while for Cl and OH: polyyne < acene < polyene. The reordering may be due to the fact that the inductive effect of the Cl and OH groups counteracts their resonance effect (see Table S1). It is also worth mentioning that other geometric parameters of the NO_2_ group (NO bond lengths, *d*_NO_, and ONO angle) correlate well with *d*_CN_ (Table S18).

### SE on the Properties of the Transmitting Moiety

**Figure 4:**
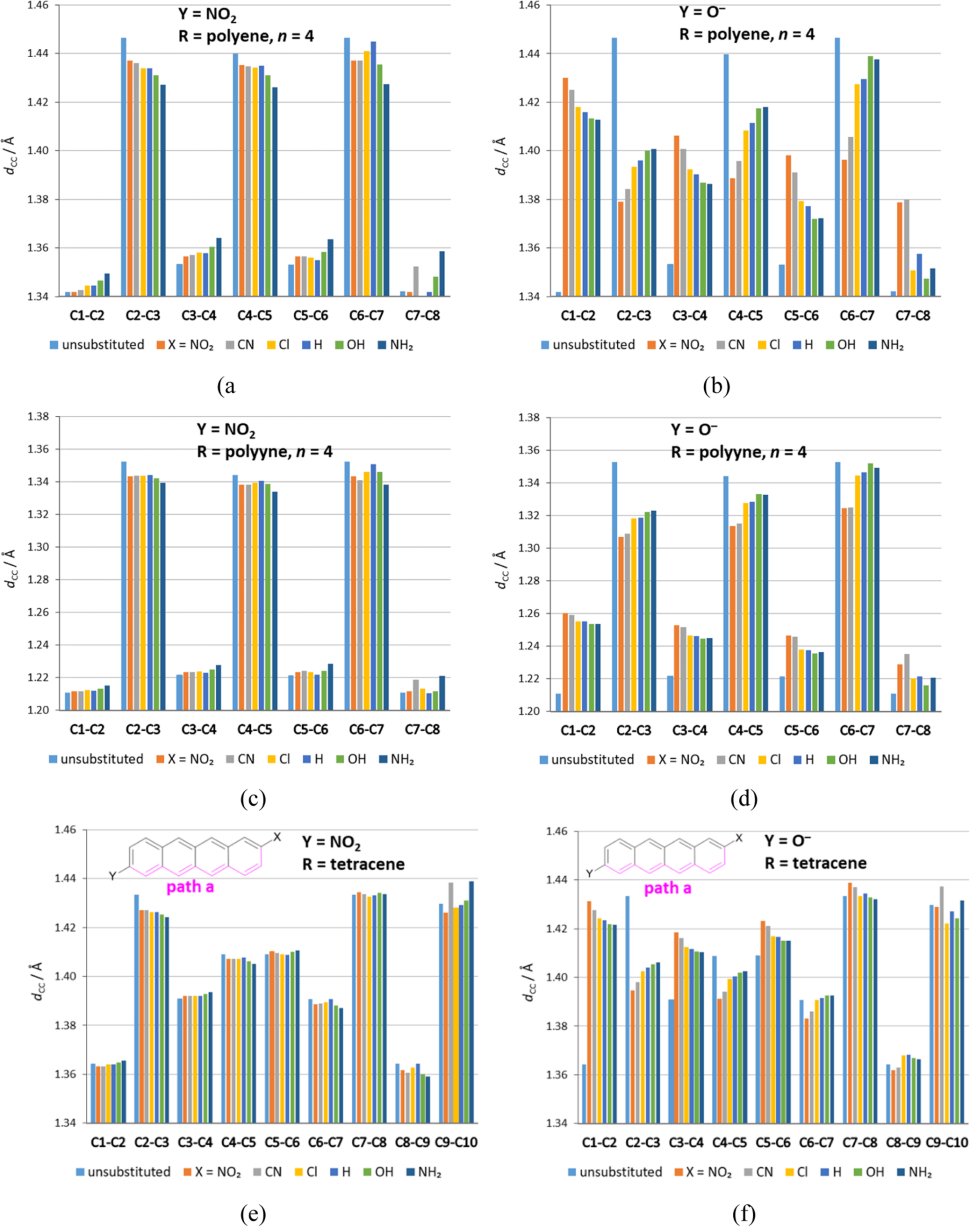
Lengths of CC bonds within the spacers R for each X derivative of six exemplary R–Y systems. The first carbon atom (C1) is the one connected to the Y group and the last (C8 or C10) to the X group. In tetracene, bonds along path a, indicated in the figure, are presented.

In Y–R–X systems, the transmitting moiety R is also subject to SEs. In order to describe them, the bond lengths of the R moieties were analyzed. Additionally, two parameters useful for evaluating the electron delocalization were calculated: for linear systems, the BLA index, and for acenes, the HOMA aromaticity index. The values of *r*^2^ (Table S20) show that statistically significant correlations between SESE and HOMA are observed only in Y = O^–^ systems. Therefore, among the Y groups, only O^–^ has enough resonance capabilities to significantly alter the π-electron structure of aromatic rings in a way that is correlated with the SE strength. Similarly, in polyenes and polyynes, statistically significant correlations between SESE and BLA exist only in Y = O^–^ systems (Table S20). Moreover, the values of slopes of SESE vs HOMA and BLA relations are in all cases higher for the Y = O^–^ derivatives than for the Y = NO_2_ systems. So, we can say again that significant and monotonic changes in the structure of R are induced only by the O^–^ group due to its strong resonance capabilities. It is also worth noting that SESE correlates much better with BLA than HOMA in linear systems (Table S20). Considering the Y–R–X systems with varying X, the ranges of variation of HOMA (for path a in acenes, see Figure 4e) and BLA (for polyene and polyyne) are higher in Y = O^–^ systems than in Y = NO_2_ (Table S21). Thus, when the interactions between Y and X are stronger, they are also reflected in the π-electron delocalization of the spacer. The ranges also decrease monotonically with an increase of *n*, which comes from the weakening of the SE. In the case of acenes, a comparison of the ranges of variation of HOMA for the bonding paths connecting Y and X indicates that the SE influences the electron delocalization along path a (shown in Figure 4e) more than for other paths (Table S21). This suggests that most of the interaction between X and Y is realized via this path.

Changes in the CC bond lengths in R due to the SEs are illustrated in Figures 4 and S4–S6 (percentage changes in relation to the unsubstituted R molecule are shown in Table S24). In Y = O^–^ systems, they are much more substantial than in Y = NO_2_. Additionally, in the former, the bond lengths are more equalized, in line with the strong resonance effect of this group (Table S1). The equalization depends on the combination of properties of the Y and X groups, that is, the differences between the neighboring bonds decrease when Y and X interact strongly (electron-donating with electron-withdrawing). Thus, for Y = NO_2_, the length of the C1–C2 bond increases with the electron-donating properties of the substituent, while for the next CC bond, it decreases, and so on (Figure 4a,c,e). On the other hand, the opposite changes in the length of CC bonds are observed in the case of Y = O^–^ systems, that is, the first bond is shortened and the second one is lengthened (Figure 4b,d,f). The greater changes in the length of CC bonds in relation to unsubstituted molecules occur for O^–^ systems than for Y = NO_2_. Additionally, the monotonicity of changes is more evident for the bonds closer to the Y group and for the Y = O^–^ derivatives. Comparing different spacers, the bond lengths in polyenes are more prone to change upon substitution than in polyynes and acenes, as evidenced by respective ranges of variability.

**Figure 5:**
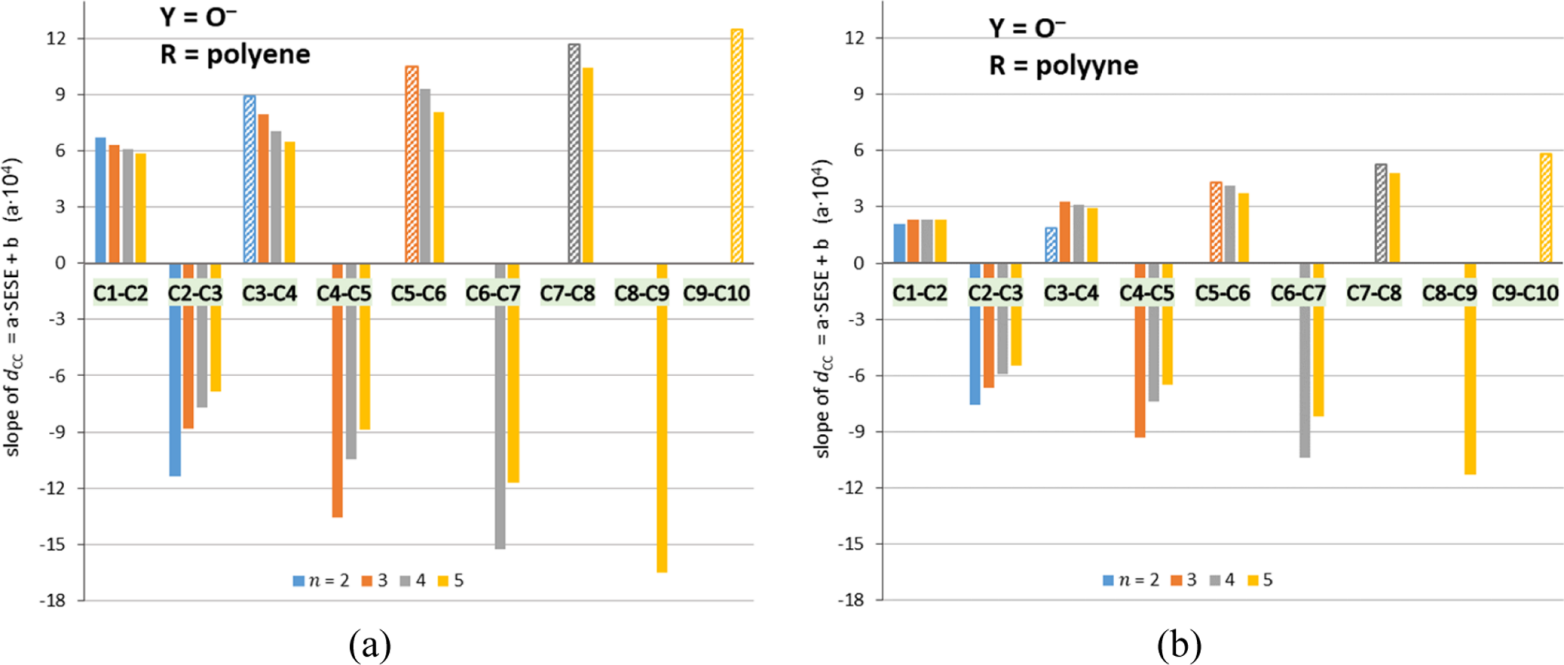
Values of slopes, *a*, of *d*_CC_ = *a*SESE + *b* correlations, multiplied by 10^4^, in X–R–Y systems with Y = O^–^ and R = polyene (a) and polyyne (b). Each correlation was calculated for a series of substituents X in the system with a given number of repeatable units, *n*. Patterned bars indicate the bond closest to the X group.

The lengths of particular CC bonds, *d*_CC_, correlate well with SESE (*r*^2^ > 0.9) only in Y = O^–^ systems. The slope values of these relations are shown in Figure 5. In polyynes, their absolute values are lower than in polyenes, which indicates smaller sensitivity of the CC bonds within the polyyne spacer to the SE. In Y = O^–^ polyenes and polyynes, slope values monotonically increase as we move further from the Y and closer to the X group (numbering of C atoms is from Y to X). Therefore, bonds closer to the X group are more sensitive to the SE. For the CC bond closest to X, *r*^2^ values are slightly lower (∼0.8), possibly due to including various X groups in the series.

## Conclusions

In summary, the computational study on Y–R–X systems (reaction sites Y = NO_2_, O^–^; substituents X = NO_2_, CN, Cl, H, OH, NH_2_; spacers R = polyene, polyyne, acene with *n* = 1–5 repeatable units) allowed us to describe the SE, emphasizing its dependence on the number of repeatable units (*n*) in the spacers and their type (R). The systems were characterized by geometric parameters and the SE by SESE and σ_p_.

The use of SESE characteristics proved to be particularly fruitful. Their relations with substituent constants, as well as their variability for individual spacers, allowed us to reveal the dependence of the substituent properties on the reaction site (Y) and spacers, both their length and type. It was shown that both inductive and resonance components of the SE as well as the total SE decay with the increase in spacer length. For polyenes and polyynes, in Y = NO_2_ systems, the inductive effect dominates, while in Y = O^–^ derivatives and both acene series, resonance. The ratio between the strengths of both effects changes with an elongation of the spacer and depends on the reaction center considered.

Taking into account the systems with the same number of CC bonds between Y and X groups and based on the correlations between SESE and σ_p_, the sequence of the SE strength transmitted through spacers was determined: polyene > polyyne > acene (for Y = NO_2_) and polyyne > polyene > acene (for Y = O^–^). However, in the latter case, for *n* ≥ 3, the sequence changes to a similar one as for Y = NO_2_ derivatives.

The observed differences in the properties of the substituents (SE strength) and their inductive/resonance character for various Y, R, and *n* are examples of the reverse SE. In addition, changes in substituent properties were also documented by the obtained relationships between SESE and C–X bond length. It was shown that chlorine behaves like an electron-withdrawing substituent in all systems, except in the Y = NO_2_, R = polyyne, where it is electron-donating. Moreover, the ability of the substituent to withdraw or donate electrons varies with the spacer length.

The geometric parameters of the studied systems are a rich source of data for the description of the SE. They generally are mutually well correlated and also correlate well with the SE descriptors. However, some relations including them are hard to interpret due to the changes in the inductive/resonance character of the substituents. The sequence of the SE strength in different spacers determined using the transmission coefficients from *d*_CY_ vs SESE relations differs from the sequence from SESE vs σ_p_ relations. This is due to the differences in length of C–Y bonds in polyenes, acenes, and polyynes and their different propensities to change. In addition, changes in the slope of the *d*_CY_ vs *d*_CX_ linear relations for individual O^–^–R–X systems (R and X are fixed, n varies) show the strength of intramolecular interactions and their dependence on the resonance and inductive nature of the substituent.

The SE on the geometry of spacers R (determined by bond lengths, their alternation, and HOMA) is negligible in the Y = NO_2_ derivatives, while in the Y = O^–^, the changes in geometry are evident, monotonic, and correlated with the strength of the SE. This results from the strong resonance effect of the O^–^ group. Comparing systems with double bonds, that is, polyenes and acenes, higher variability of bond lengths due to substitution is observed in polyenes. This may be related to the high resistance of the aromatic π-electron structure of acenes to the SE, as previously reported.^47^ This fact may also be the reason for the observed weakest transmission of the SE through acene linkers.

## Computational Methods

All calculations were performed with the density functional theory at the B3LYP/6-311++G(d,p) level (tight convergence criteria, UltraFine grid) in Gaussian 09 program.^51^ This functional was chosen due to its good overall performance in predicting the geometry and energetics.^52,53^ After each geometry optimization, calculation of vibrational frequencies was performed to confirm that the geometry corresponds to the minimum on the potential energy surface. In X = OH derivatives, the lowest energy conformation of the OH group was considered. SESE was calculated using reactions of the type shown in Scheme 2. The energy of each reagent was corrected for the zero point energy.

The HOMA index^46^ allows us to evaluate the aromaticity of a selected molecular fragment (local aromaticity) or for the entire molecule (global aromaticity) from its bond lengths. It can be calculated from formula 4
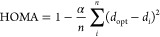
4where α is a normalization constant (chosen to give HOMA = 0 for a model non-aromatic system and HOMA = 1 for a system where all bond lengths are equal to *d*_opt_), *n* is the number of bonds in the molecular fragment (e.g., ring), and *d*_*i*_ are the experimental or computed bond lengths of the fragment. The HOMA value can be interpreted as a normalized sum of differences between the actual and reference bond lengths, where the reference ones are assumed to represent a fully aromatic system with HOMA = 1.

The BLA^44,45^ index is determined as the sum of the absolute values of the deviations of the particular CC bond lengths from the average bond lengths divided by the number of bonds taken into account.

## Data Availability

The data underlying this study are available in the published article and its Supporting Information.

## Supporting Information Available

Obtained values of SESE, HOMA, and BLA; structural parameters of the studied systems; determination coefficients and slopes of the discussed linear correlations; and Cartesian coordinates and electronic, zero point, and Gibbs energies of the optimized structures (PDF)
Folder with .xyz files of optimized structures (ZIP)
