# Supplementary material for: Substituent Effects from the Point of View of Energetics and Molecular Geometry in Acene, Polyene, and Polyyne Derivatives
Source: J Org Chem. 2023 Jun 2;88(13):7940–52. doi: 10.1021/acs.joc.2c02936 (PMC10336965; doi:10.1021/acs.joc.2c02936)
Supplement: Supplementary file 1 — jo2c02936_si_001.pdf [file jo2c02936_si_001.pdf]

Supporting Information

Substituent effects from the point of view of energetics and molecular geometry in acene, polyene and polyyne derivatives.

Mozhgan Shahamirian,[1] Paweł A. Wieczorkiewicz,[2,*] Tadeusz M. Krygowski,[3] Halina Szatylowicz[2,*]

[1] Department of Chemistry, Faculty of Science, Sarvestan Branch, Islamic Azad University, Sarvestan, 73451-173, Iran
[2] Faculty of Chemistry, Warsaw University of Technology, Noakowskiego 3, 00-664 Warsaw, Poland
[3] Faculty of Chemistry, University of Warsaw, Pasteura 1, 02-093 Warsaw, Poland
* corresponding authors; Email: pawel.wieczorkiewicz.dokt@pw.edu.pl and halina.szatylowicz@pw.edu.pl

Table of contents

Table S1. Substituent constants of groups X and Y and their field (*F*) and resonance (*R*) parameters S2
Table S2. The obtained SESE values (in kcal/mol) for the Y-R-X systems. S2
Table S3. Fitting parameters for dependences of substituent effect strength on spacer length *n* (*n* from 1 to 10). Equation form $y = a\cdot\exp(b/(n+c))$. S3
Table S4. Statistical data on SESE = $\alpha\cdot F+\beta\cdot R$ correlations in Y-R-X systems, Y = NO_2, X = NO_2, CN, Cl, H, OH, NH_2. S3
Table S5. Statistical data on SESE = $\alpha\cdot F+\beta\cdot R$ correlations in Y-R-X systems, Y = O^-, X = NO_2, CN, Cl, H, OH, NH_2. S4
Table S6. Statistical data on $d_{CY} = a\cdot$SESE + b correlations in Y-R-X systems with a given number (*n*) of repeatable units in R for a series of substituents X= NO_2, CN, Cl, H, OH, NH_2. S4
Table S7. Structural parameters of Y-R-X systems, where R = polyene, Y = NO_2. All bond lengths in Å, angles in degrees. Row unsub. contain the values for unsubstituted R moiety (so Y = H and X = H). S5
Table S8. Structural parameters of Y-R-X systems, where R = polyyne, Y = NO_2. Tables with X = NO_2, CN and NH_2 rows contain the parameters of the X groups. S8
Table S9. Structural parameters of Y-R-X systems, where R = acene, Y = NO_2. Tables with X = NO_2, CN and NH_2 rows contain the parameters of the X groups. S11
Table S10. Structural parameters of Y-R-X systems, where R= polyene, Y = O^-. Tables with X = NO_2, CN and NH_2 rows contain the parameters of the X groups. S13
Table S11. Structural parameters of Y-R-X systems, where R= polyyne, Y = O^-. Tables with X = NO_2, CN and NH_2 rows contain the parameters of the X groups. S16
Table S12. Structural parameters of Y-R-X systems, where R= acene, Y = O^-. Tables with X = NO_2, CN and NH_2 rows contain the parameters of the X groups. S19
Table S13. Statistical data on the SESE = $a\cdot\sigma_p(^-) + b$ correlations in Y-R-X systems (Y= O^- and X= NO_2. CN. Cl. H. OH. NH2) S22
Table S14. The obtained determination coefficients and slopes (± estimated standard deviation) of the relations between SESE and d_{CX} bond lengths, Y = NO_2. S22
Table S15. The obtained determination coefficients and slopes (± estimated standard deviation) of the relations between SESE and d_{CX} bond lengths, Y = O^-. S23
Table S16. The obtained determination coefficients and slopes (± estimated standard deviation) of the relations between d_{CY} and d_{CX} bond lengths S23
Table S17. The transmission coefficient, γ, in Y-R-X systems on the basis of the relations: $d_{CY} = a\cdot$SESE + b, where R = polyyne, polyene and acene. S24
Table S18. The obtained determination coefficients and slopes (± estimated standard deviation) for the relations between geometric parameters of the nitro group in Y = NO_2 systems. S24
Table S19. The obtained determination coefficients and slopes (± estimated standard deviation) of the linear dependence of P(X) = SESE, d_{CN}, ∠ONO or d_{CO} on the pyramidalization of the amino group, Φ_{NH2}, in X = NH_2 derivatives (Y, R, X fixed, *n* varies). S25
Table S20. The obtained determination coefficients and slopes (± estimated standard deviation) of the relations between SESE and HOMA or BLA. S25
Table S21. The ranges of variation of HOMA and BLA indices for the Y-R-X systems (Y, R and *n* fixed, X varies). S27
Scheme S1. Resonance structures in acene, polyene and polyyne derivatives. S28
Figure S1. Relative strength of the SE as a function of the reciprocal of spacer length, 1/*n*. S29
Figure S2. Dependence of the range of variation of SESE on the length of the transmitting fragment for Y = NO_2 (a) and Y = O^- (b) Y-R-X derivatives (X = NO_2, CN, Cl, H, OH, NH_2). S29
Figure S3. Relationships between C-Y and C-X bond lengths (d_{CY} and d_{CX}) for Y=NO_2 and O^-acene (a,b), polyene (c,d) and polyyne (e,f) systems (*n*=2-5, *n*=1-4 for acenes). S30
Figure S4. Lengths of CC bonds within the spacers R for each X derivative of six polyene R-Y systems. S31
Figure S5. Lengths of CC bonds within the spacers R for each X derivative of six polyyne R-Y systems. S32
Figure S6. Lengths of CC bonds within the spacers R for each X derivative of anthracene-Y systems. S34
Table S22. CC bond lengths along the delocalization paths between X and Y = NO_2 in acenes. S34
Table S23. CC bond lengths along the delocalization paths between X and Y = O^- in acenes. S36
Table S24. Changes in C-C bond lengths in percentages, relative to the unsubstituted R molecule. S39
Table S25. Changes in C-Y (C-N or C-O) and C-X bond lengths in percentages, relative to the monosubstituted R derivative. S47
Table S26. Conformational analysis of the X = OH polyene and acene derivatives. S49
Computational details. Cartesian coordinates, electronic energies (E), zero point energies (ZPE) and Gibbs energies (G), B3LYP/6-311++G(d,p) method S50

Table S1. Substituent constants of groups X and Y and their field (*F*) and resonance (*R*) parameters, taken from Ref: Hansch, Corwin.; Leo, A.; Taft, R. W. A *Chem. Rev.* **1991**, *91* (2), 165–195.

Group	σ_m	σ_p	σ_p^-	*F*	*R*
NO_2	0.71	0.78	1.27	0.65	0.13
CN	0.56	0.66	1.00	0.51	0.15
Cl	0.37	0.23	0.19	0.42	−0.19
H	0.00	0.00	0.00	0.00	0.00
OH	0.12	−0.17	−0.37	0.33	−0.70
NH_2	−0.16	−0.66	−0.15	0.08	−0.74
O^-	−0.47	−0.81	−0.82	−0.26	−0.55
C_6H_5	0.06	−0.01	0.02	0.12	−0.13
$CH{=}CH_2$	0.06	−0.04		0.13	−0.17

Table S2. The obtained SESE values (in kcal/mol) for the Y-R-X systems. In italics are given the ratios between the ranges of variation for R and R=acene.

	NO_2-R-X					O^--R-X				
X \ R	polyene		polyyne		acene	polyene		polyyne		acene
	n=1		*n*=1			*n*=1		*n*=1		
NO_2	−8.69		−4.14			37.85		39.74		
CN	−4.88		−3.78			29.53		34.79		
Cl	−2.98		0.29			7.14		4.77		
H	0.00		0.00			0.00		0.00		
OH	0.21		3.13			−8.96		−12.54		
NH_2	6.17		8.11			−9.83		−16.84		
range	14.85		12.25			47.68		56.58		
	n=2		*n*=2		*n*=1	*n*=2		*n*=2		*n*=1
NO_2	−4.40		−2.59		−3.74	31.43		29.32		25.67
CN	−3.17		−2.66		−2.91	22.01		25.12		19.05
Cl	−0.44		0.74		−0.97	5.97		0.56		5.84
H	0.00		0.00		0.00	0.00		0.00		0.00
OH	2.35		2.78		1.17	−5.53		−9.48		−3.62
NH_2	5.67		5.78		3.21	−9.10		−12.34		−6.08
range	10.07	*1.45*	8.45	*1.21*	6.95	40.54	*1.28*	41.66	*1.31*	31.75
	n=3		*n*=3		*n*=2	*n*=3		*n*=3		*n*=2
NO_2	−3.39		−2.20		−2.72	25.67		23.24		18.83
CN	−2.50		−2.22		−2.15	17.62		19.14		13.77
Cl	−0.36		0.26		−0.74	4.38		−0.51		4.56
H	0.00		0.00		0.00	0.00		0.00		0.00
OH	1.70		1.89		0.70	−4.63		−7.52		−1.98
NH_2	3.97		4.20		1.85	−8.56		−10.36		−4.73
range	7.35	*1.61*	6.42	*1.41*	4.57	34.23	*1.45*	33.59	*1.43*	23.56
	n=4		*n*=4		*n*=3	*n*=4		*n*=4		*n*=3
NO_2	−2.95		−1.86		−1.80	21.74		18.99		14.84
CN	−2.05		−1.87		−1.47	14.72		15.44		10.63

Cl	−0.31		−0.15		−0.46	3.58		−0.34		3.52
H	0.00		0.00		0.00	0.00		0.00		0.00
OH	1.17		1.45		0.48	−3.92		−6.50		−1.39
NH_2	2.91		3.24		1.25	−7.76		−9.10		−3.84
range	5.86	*1.92*	5.12	*1.68*	3.05	29.50	*1.58*	28.09	*1.50*	18.67
	n=5		*n*=5		*n*=4	*n*=5		*n*=5		*n*=4
NO_2	−2.38		−0.87		−1.33	18.92		16.12		12.12
CN	−1.68		−1.52		−1.13	12.81		12.84		8.56
Cl	−0.27		−1.31		−0.33	2.98		−1.05		2.75
H	0.00		0.00		0.00	0.00		0.00		0.00
OH	1.00		1.48		0.37	−4.71		−5.75		−1.12
NH_2	2.33		2.29		0.91	−6.95		−7.69		−3.32
range	4.71	*2.11*	3.82	*1.71*	2.24	25.87	*1.68*	23.81	*1.54*	15.44
	n=10		*n*=10			*n*=10		*n*=10		
NO_2	-1.17		-0.70			11.86		8.14		
CN	-0.82		-0.61			7.73		6.60		
Cl	-0.16		0.07			1.67		-0.30		
H	0.00		0.00			0.00		0.00		
OH	0.39		0.42			-3.69		-2.82		
NH_2	-1.17		0.78			11.86		-4.27		
range	2.14		1.48			16.40		12.41		

Table S3. Fitting parameters for dependencies of substituent effect strength on spacer length *n* (*n* from 1 to 10). Equation form y = a·exp(b/(*n*+c)). Fitting against 1/*n*, as suggested in some literature (Ref. 41 in the manuscript) did not yield good results, as shown in Figure S6.

	**Y = NO_2**			**Y = O^-**		
	R = polyene	R = polyyne	R = acene	R = polyene	R = polyyne	R = acene
**a**	1.993	0.4198	0.0817	17.4328	6.4918	8.7231
**b**	59.5659	104.3075	114.8048	18.3963	35.7181	15.6595
**c**	12.0195	16.3203	14.1418	7.3714	9.6743	4.4199
r^2	0.9998	0.9985	0.9995	0.9998	0.9994	1.0000

Table S4. Statistical data on SESE = $\alpha \cdot F + \beta \cdot R$ correlations in Y-R-X systems, Y = NO_2, X = NO_2, CN, Cl, H, OH, NH_2. α/β is the ratio between coefficients for field (*a*) and resonance (*b*) parameter.

R	*n*	α	$\pm\,\alpha$	β	$\pm\,\beta$	r^2	α/β
polyene	1	−7.39	1.37	−10.63	1.46	0.945	0.70
	2	−7.06	0.68	−5.03	0.73	0.971	1.40
	3	−5.11	0.43	−3.91	0.46	0.978	1.31
	4	−3.87	0.36	−3.36	0.39	0.975	1.15
	5	−3.15	0.26	−2.73	0.28	0.980	1.15
polyyne	1	−9.32	1.20	−4.89	1.29	0.942	1.91
	2	−6.92	0.77	−2.87	0.82	0.955	2.41
	3	−5.08	0.54	−2.62	0.58	0.959	1.94
	4	−3.96	0.38	−2.37	0.40	0.969	1.67
	5	−2.89	0.65	−1.87	0.69	0.853	1.55
acene	1	−4.42	0.27	−4.72	0.29	0.991	0.94
	2	−2.80	0.13	−3.47	0.14	0.996	0.81

	3	−1.90	0.10	−2.31	0.10	0.995	0.82
	4	−1.41	0.07	−1.72	0.08	0.995	0.82

Table S5. Statistical data on SESE = $\alpha \cdot F+\beta \cdot R$ correlations in Y-R-X systems, Y = O^-, X = NO_2, CN, Cl, H, OH, NH_2. α/β is the ratio between coefficients for field (*a*) and resonance (*b*) parameter.

R	*n*	α	$\pm\alpha$	β	$\pm\beta$	r^2	α/β
polyene	1	27.97	5.80	45.91	6.19	0.943	0.61
	2	22.12	4.16	37.16	4.45	0.954	0.60
	3	18.85	3.32	29.98	3.55	0.956	0.63
	4	16.28	2.76	25.26	2.95	0.958	0.64
	5	15.08	2.53	21.66	2.70	0.954	0.70
polyyne	1	36.79	6.09	49.00	6.51	0.951	0.75
	2	27.37	5.51	34.57	5.88	0.925	0.79
	3	21.99	4.51	26.59	4.82	0.919	0.83
	4	18.55	3.52	21.62	3.76	0.927	0.86
	5	15.87	3.28	17.83	3.50	0.911	0.89
acene	1	16.80	3.34	31.46	3.56	0.957	0.53
	2	12.03	2.19	23.24	2.34	0.965	0.52
	3	9.41	1.70	18.20	1.82	0.966	0.52
	4	7.78	1.39	14.75	1.48	0.966	0.53

Table S6. Statistical data on d_{CY} = $a \cdot$SESE + b correlations in Y-R-X systems with a given number (*n*) of repeatable units in R for a series of substituents X= NO_2, CN, Cl, H, OH, NH_2.

R	*n*	Y=NO_2			Y=O^-		
		$a \cdot 10^4$	$\pm a \cdot 10^4$	r^2	$a \cdot 10^4$	$\pm a \cdot 10^4$	r^2
polyene	1	−26.2	9.7	0.644	−6.00	0.76	0.940
	2	−24.6	2.0	0.974	−5.40	0.39	0.979
	3	−24.8	1.1	0.992	−5.05	0.22	0.992
	4	−24.9	1.1	0.992	−4.79	0.18	0.995
	5	−25.3	0.8	0.996	−4.53	0.12	0.997
polyyne	1	−21.6	3.5	0.905	−5.36	1.06	0.864
	2	−21.2	2.2	0.958	−5.02	0.34	0.982
	3	−20.4	1.7	0.973	−4.78	0.13	0.997
	4	−19.6	1.3	0.983	−4.47	0.14	0.996
	5	−17.7	4.1	0.822	−4.25	0.13	0.997
acene	1	−34.9	5.9	0.898	−5.85	0.62	0.957
	2	−29.8	3.6	0.946	−5.80	0.22	0.994
	3	−28.5	1.8	0.985	−5.49	0.23	0.993
	4	−27.7	1.2	0.993	−5.42	0.23	0.993

Table S7. Structural parameters of Y-R-X systems, where R = polyene, Y = NO_2. All bond lengths in Å, angles in degrees. Row unsub. contain the values for unsubstituted R moiety (so Y = H and X = H).

Y = NO_2, R=polyene, *n*=1						
	d_{CY}	d_{NO1}	d_{NO2}	∠ONO	d_{CX}	C1-C2
unsub.						1.329
X=NO_2	1.471	1.222	1.222	126.2	1.471	1.323
CN	1.469	1.222	1.224	125.9	1.421	1.332
Cl	1.462	1.225	1.225	125.4	1.723	1.328
H	1.472	1.223	1.226	125.4		1.323
OH	1.445	1.229	1.229	124.8	1.339	1.340
NH_2	1.432	1.235	1.233	124.0	1.352	1.350

Y = NO_2, R=polyene, *n*=1								
	d_{NO1}	d_{NO2}	∠ONO	d_{CN}	d_{NH1}	d_{NH2}	∠HNH	Φ_{NH2}
X=NO_2	1.222	1.222	126.2					
CN				1.155				
NH_2					1.005	1.007	117.5	360.0

Y = NO_2, R=polyene, *n*=2										
	d_{CY}	d_{NO1}	d_{NO2}	∠ONO	d_{CX}	C1-C2	C2-C3	C3-C4	HOMA	BLA
unsub.						1.338	1.456	1.338	0.177	0.0524
X=NO_2	1.463	1.224	1.226	125.5	1.463	1.336	1.446	1.336	0.251	0.0488
CN	1.460	1.225	1.227	125.3	1.421	1.338	1.444	1.348	0.376	0.0449
Cl	1.455	1.227	1.229	124.9	1.731	1.339	1.443	1.339	0.328	0.0463
H	1.456	1.227	1.229	124.9		1.338	1.448	1.340	0.271	0.0487
OH	1.447	1.230	1.231	124.5	1.344	1.343	1.435	1.348	0.501	0.0397
NH_2	1.438	1.233	1.234	124.0	1.357	1.349	1.426	1.360	0.679	0.0317

Y = NO_2, R=polyene, *n*=2								
	d_{NO1}	d_{NO2}	∠ONO	d_{CN}	d_{NH1}	d_{NH2}	∠HNH	Φ_{NH2}
X=NO_2	1.224	1.226	125.5					
CN				1.156				
NH_2					1.004	1.007	117.4	359.7

Y = NO_2, R=polyene, *n*=3												
	d_{CY}	d_{NO1}	d_{NO2}	∠ONO	d_{CX}	C1-C2	C2-C3	C3-C4	C4-C5	C5-C6	HOMA	BLA
unsub.						1.341	1.449	1.350	1.449	1.341	0.315	0.0504
X=NO_2	1.458	1.226	1.228	125.2	1.458	1.340	1.440	1.353	1.440	1.340	0.417	0.0461
CN	1.455	1.227	1.229	125.0	1.419	1.341	1.439	1.354	1.439	1.351	0.484	0.0435
Cl	1.450	1.229	1.230	124.7	1.735	1.343	1.437	1.354	1.442	1.340	0.444	0.0450
H	1.450	1.229	1.231	124.7		1.342	1.438	1.353	1.446	1.341	0.419	0.0461
OH	1.446	1.230	1.232	124.4	1.348	1.345	1.433	1.357	1.435	1.348	0.558	0.0402
NH_2	1.439	1.233	1.234	124.1	1.362	1.349	1.427	1.363	1.427	1.359	0.690	0.0336

Y = NO_2, R=polyene, *n*=3								
	d_{NO1}	d_{NO2}	∠ONO	d_{CN}	d_{NH1}	d_{NH2}	∠HNH	Φ_{NH2}
X=NO_2	1.228	1.226	125.2					
CN				1.157				
NH_2					1.005	1.007	116.6	357.3

Y = NO_2, R=polyene, *n*=4														
	d_{CY}	d_{NO1}	d_{NO2}	∠ONO	d_{CX}	C1-C2	C2-C3	C3-C4	C4-C5	C5-C6	C6-C7	C7-C8	HOMA	BLA
unsub.						1.342	1.446	1.353	1.440	1.353	1.446	1.342	0.404	0.0473
X=NO_2	1.454	1.227	1.229	125.0	1.454	1.342	1.437	1.357	1.435	1.357	1.437	1.342	0.512	0.0427

CN	1.452	1.228	1.230	124.9	1.419	1.343	1.436	1.357	1.435	1.357	1.437	1.352	0.552	0.0410
Cl	1.448	1.229	1.231	124.6	1.737	1.344	1.434	1.358	1.434	1.356	1.441	1.340	0.515	0.0425
H	1.447	1.230	1.231	124.6		1.345	1.434	1.358	1.435	1.355	1.445	1.342	0.501	0.0432
OH	1.444	1.233	1.231	124.4	1.350	1.346	1.431	1.360	1.431	1.358	1.435	1.348	0.598	0.0388
NH_2	1.439	1.233	1.235	124.1	1.365	1.349	1.427	1.364	1.426	1.364	1.427	1.359	0.703	0.0333

Y = NO_2, R=polyene, *n*=4								
	d_{NO1}	d_{NO2}	∠ONO	d_{CN}	d_{NH1}	d_{NH2}	∠HNH	Φ_{NH2}
X=NO_2	1.227	1.229	125.0					
CN				1.157				
NH_2					1.006	1.008	116.1	355.9

Y = NO_2, R=polyene, *n*=5																
	d_{CY}	d_{NO1}	d_{NO2}	∠ONO	d_{CX}	C1-C2	C2-C3	C3-C4	C4-C5	C5-C6	C6-C7	C7-C8	C8-C9	C9-C10	HOMA	BLA
unsub.						1.342	1.446	1.355	1.437	1.357	1.437	1.355	1.446	1.342	0.464	0.0449
X=NO_2	1.451	1.228	1.230	124.8	1.451	1.343	1.435	1.358	1.433	1.359	1.433	1.358	1.435	1.343	0.571	0.0402
CN	1.450	1.229	1.231	124.7	1.418	1.344	1.434	1.359	1.432	1.359	1.433	1.358	1.436	1.353	0.597	0.0390
Cl	1.446	1.230	1.232	124.5	1.739	1.346	1.432	1.360	1.431	1.360	1.433	1.357	1.441	1.341	0.563	0.0404
H	1.445	1.230	1.232	124.5		1.346	1.432	1.360	1.431	1.360	1.434	1.356	1.445	1.342	0.553	0.0409
OH	1.443	1.231	1.233	124.4	1.351	1.347	1.430	1.362	1.429	1.362	1.431	1.359	1.436	1.348	0.627	0.0375
NH_2	1.439	1.233	1.235	124.1	1.367	1.350	1.427	1.365	1.425	1.365	1.426	1.364	1.428	1.358	0.715	0.0327

Y = NO_2, R=polyene, *n*=5								
	d_{NO1}	d_{NO2}	∠ONO	d_{CN}	d_{NH1}	d_{NH2}	∠HNH	Φ_{NH2}
X=NO_2	1.228	1.230	124.8					
CN				1.158				
NH_2					1.006	1.008	115.8	354.9

Table S8. Structural parameters of Y-R-X systems, where R = polyyne, Y = NO_2. Tables with X = NO_2, CN and NH_2 rows contain the parameters of the X groups.

Y = NO_2, R=polyyne, *n*=1						
	d_{CY}	d_{NO1}	d_{NO2}	∠ONO	d_{CX}	C1-C2
unsub.						1.199
X=NO_2	1.402	1.221	1.221	127.5	1.402	1.197
CN	1.401	1.222	1.222	127.2	1.366	1.203
Cl	1.397	1.225	1.225	126.2	1.634	1.200
H	1.401	1.224	1.224	126.5		1.197
OH	1.389	1.228	1.227	125.8	1.292	1.199
NH_2	1.375	1.232	1.232	125.4	1.315	1.208

Y = NO_2, R=polyyne, *n*=1								
	d_{NO1}	d_{NO2}	∠ONO	d_{CN}	d_{NH1}	d_{NH2}	∠HNH	Φ_{NH2}
X=NO_2	1.221	1.221	127.5					
CN				1.158				
NH_2					1.008	1.008	118.1	360.0

Y = NO_2, R=polyyne, *n*=2										
	d_{CY}	d_{NO1}	d_{NO2}	∠ONO	d_{CX}	C1-C2	C2-C3	C3-C4	HOMA	BLA
unsub.						1.207	1.365	1.207	−4.657	0.0700
X=NO_2	1.394	1.224	1.224	127.0	1.394	1.208	1.352	1.208	−4.692	0.0640
CN	1.395	1.224	1.224	126.8	1.361	1.208	1.351	1.214	−4.484	0.0619
Cl	1.390	1.227	1.227	126.2	1.631	1.208	1.353	1.210	−4.590	0.0640
H	1.392	1.226	1.226	126.3		1.207	1.358	1.207	−4.694	0.0671
OH	1.385	1.229	1.229	125.8	1.292	1.210	1.352	1.209	−4.582	0.0632
NH_2	1.377	1.232	1.232	125.6	1.315	1.214	1.344	1.219	−4.227	0.0565

Y = NO_2, R=polyyne, *n*=2								
	d_{NO1}	d_{NO2}	∠ONO	d_{CN}	d_{NH1}	d_{NH2}	∠HNH	$Φ_{NH2}$
X=NO_2	1.224	1.224	127.0					
CN				1.160				
NH_2					1.009	1.009	117.7	360.0

Y = NO_2, R=polyyne, *n*=3												
	d_{CY}	d_{NO1}	d_{NO2}	∠ONO	d_{CX}	C1-C2	C2-C3	C3-C4	C4-C5	C5-C6	HOMA	BLA
unsub.						1.210	1.356	1.218	1.356	1.210	−3.876	0.0687
X=NO_2	1.391	1.225	1.225	126.7	1.391	1.211	1.346	1.220	1.346	1.211	−3.887	0.0634
CN	1.392	1.226	1.226	126.6	1.359	1.210	1.346	1.221	1.344	1.218	−3.756	0.0617
Cl	1.388	1.228	1.228	126.1	1.631	1.211	1.346	1.220	1.348	1.213	−3.819	0.0636
H	1.389	1.227	1.227	126.2		1.211	1.348	1.219	1.353	1.210	−3.885	0.0659
OH	1.384	1.229	1.229	125.9	1.293	1.212	1.345	1.221	1.347	1.211	−3.810	0.0630
NH_2	1.378	1.231	1.231	125.7	1.315	1.215	1.340	1.226	1.339	1.221	−3.577	0.0572

Y = NO_2, R=polyyne, *n*=3								
	d_{NO1}	d_{NO2}	∠ONO	d_{CN}	d_{NH1}	d_{NH2}	∠HNH	$Φ_{NH2}$
X=NO_2	1.225	1.225	126.7					
CN				1.161				
NH_2					1.009	1.009	117.7	360.0

Y = NO_2, R=polyyne, *n*=4														
	d_{CY}	d_{NO1}	d_{NO2}	∠ONO	d_{CX}	C1-C2	C2-C3	C3-C4	C4-C5	C5-C6	C6-C7	C7-C8	HOMA	BLA
unsub.						1.211	1.353	1.221	1.344	1.221	1.353	1.211	−3.518	0.0655
X=NO_2	1.390	1.226	1.226	126.6	1.390	1.212	1.344	1.223	1.338	1.223	1.344	1.212	−3.522	0.0609

CN	1.390	1.226	1.226	126.5	1.358	1.212	1.344	1.223	1.338	1.224	1.341	1.219	−3.428	0.0595
Cl	1.387	1.228	1.228	126.1	1.631	1.212	1.344	1.224	1.340	1.223	1.346	1.213	−3.472	0.0612
H	1.388	1.228	1.228	126.2		1.212	1.344	1.223	1.341	1.222	1.351	1.211	−3.520	0.0630
OH	1.384	1.229	1.229	126.0	1.293	1.213	1.342	1.225	1.339	1.224	1.346	1.212	−3.482	0.0607
NH_2	1.380	1.231	1.231	125.8	1.316	1.215	1.339	1.228	1.334	1.229	1.338	1.221	−3.291	0.0559

Y = NO_2, R=polyyne, *n*=4								
	d_{NO1}	d_{NO2}	∠ONO	d_{CN}	d_{NH1}	d_{NH2}	∠HNH	Φ_{NH2}
X=NO_2	1.226	1.226	126.6					
CN				1.161				
NH_2					1.009	1.009	117.6	359.7

Y = NO_2, R=polyyne, *n*=5															
	d_{CY}	d_{NO1}	d_{NO2}	∠ONO	d_{CX}	C1-C2	C2-C3	C3-C4	C4-C5	C5-C6	C6-C7	C7-C8	C8-C9	C9-C10	HOMA	BLA
unsub.						1.211	1.351	1.223	1.341	1.226	1.341	1.223	1.351	1.211	−3.312	0.0628
X=NO_2	1.389	1.227	1.227	126.5	1.389	1.212	1.343	1.225	1.336	1.227	1.336	1.225	1.343	1.212	−3.314	0.0588
CN	1.389	1.227	1.227	126.4	1.358	1.212	1.343	1.225	1.336	1.227	1.335	1.226	1.340	1.220	−3.238	0.0576
Cl	1.386	1.228	1.228	126.1	1.632	1.213	1.342	1.225	1.336	1.227	1.337	1.225	1.345	1.214	−3.272	0.0590
H	1.387	1.228	1.228	126.2		1.213	1.343	1.225	1.337	1.227	1.338	1.223	1.350	1.211	−3.309	0.0605
OH	1.384	1.229	1.229	126.0	1.294	1.214	1.341	1.226	1.335	1.228	1.337	1.225	1.346	1.212	−3.265	0.0586
NH_2	1.381	1.230	1.230	125.8	1.317	1.215	1.339	1.228	1.332	1.231	1.332	1.230	1.338	1.221	−3.129	0.0546

Y = NO_2, R=polyyne, *n*=5								
	d_{NO1}	d_{NO2}	∠ONO	d_{CN}	d_{NH1}	d_{NH2}	∠HNH	Φ_{NH2}
X=NO_2	1.227	1.227	126.5					
CN				1.162				
NH_2					1.009	1.009	117.3	358.9

Table S9. Structural parameters of Y-R-X systems, where R = acene, Y = NO_2. Tables with X = NO_2, CN and NH_2 rows contain the parameters of the X groups.

Y = NO_2, R=acene, *n*=1						
	d_{CY}	d_{NO1}	d_{NO2}	∠ONO	d_{CX}	HOMA (I)
unsub.						0.989
X=NO_2	1.486	1.222	1.222	125.2	1.486	0.998
CN	1.484	1.223	1.223	125.1	1.432	0.980
Cl	1.478	1.225	1.225	124.8	1.749	0.995
H	1.481	1.225	1.225	124.7		0.993
OH	1.469	1.226	1.227	124.5	1.358	0.985
NH_2	1.461	1.229	1.229	124.1	1.378	0.956

Y = NO_2, R=acene, *n*=1								
	d_{NO1}	d_{NO2}	∠ONO	d_{CN}	d_{NH1}	d_{NH2}	∠HNH	Φ_{NH2}
X=NO_2	1.222	1.222	125.2					
CN				1.155				
NH_2					1.007	1.007	114.8	351.5

Y = NO_2, R=acene, *n*=2							
	d_{CY}	d_{NO1}	d_{NO2}	∠ONO	d_{CX}	HOMA (I)	HOMA (II)
unsub.						0.784	0.783
X=NO_2	1.483	1.224	1.224	124.9	1.483	0.792	0.792
CN	1.481	1.224	1.224	124.9	1.431	0.793	0.774
Cl	1.478	1.225	1.225	124.6	1.754	0.796	0.79

H	1.478	1.225	1.226	124.6		0.789	0.786
OH	1.473	1.227	1.226	124.5	1.362	0.799	0.776
NH_2	1.469	1.228	1.228	124.2	1.385	0.789	0.761

Y = NO_2, R=acene, *n*=2								
	d_{NO1}	d_{NO2}	∠ONO	d_{CN}	d_{NH1}	d_{NH2}	∠HNH	Φ_{NH2}
X=NO_2	1.224	1.224	124.9					
CN				1.155				
NH_2					1.008	1.008	113.8	348.5

Y = NO_2, R=acene, *n*=3								
	d_{CY}	d_{NO1}	d_{NO2}	∠ONO	d_{CX}	HOMA (I)	HOMA (II)	HOMA (III)
unsub.						0.630	0.721	0.629
X=NO_2	1.480	1.225	1.225	124.7	1.479	0.646	0.719	0.646
CN	1.478	1.225	1.225	124.7	1.431	0.644	0.723	0.622
Cl	1.476	1.226	1.226	124.6	1.755	0.646	0.724	0.637
H	1.475	1.226	1.226	124.5		0.642	0.718	0.634
OH	1.473	1.227	1.227	124.4	1.364	0.654	0.717	0.626
NH_2	1.470	1.228	1.228	124.3	1.387	0.653	0.707	0.617

Y = NO_2, R=acene, *n*=3								
	d_{NO1}	d_{NO2}	∠ONO	d_{CN}	d_{NH1}	d_{NH2}	∠HNH	Φ_{NH2}
X=NO_2	1.225	1.225	124.8					
CN				1.156				
NH_2					1.008	1.009	113.5	347.4

Y = NO_2, R=acene, *n*=4									
	d_{CY}	d_{NO1}	d_{NO2}	∠ONO	d_{CX}	HOMA (I)	HOMA (II)	HOMA (III)	HOMA (IV)
unsub.						0.538	0.632	0.632	0.538
X=NO_2	1.477	1.226	1.225	124.6	1.477	0.560	0.633	0.633	0.560
CN	1.476	1.226	1.226	124.6	1.430	0.558	0.632	0.638	0.533
Cl	1.474	1.227	1.226	124.5	1.756	0.559	0.631	0.639	0.547
H	1.474	1.227	1.227	124.4		0.557	0.628	0.635	0.545
OH	1.473	1.227	1.227	124.4	1.365	0.565	0.633	0.632	0.537
NH_2	1.471	1.228	1.228	124.3	1.389	0.568	0.628	0.625	0.527

Y = NO_2, R=acene, *n*=4								
	d_{NO1}	d_{NO2}	∠ONO	d_{CN}	d_{NH1}	d_{NH2}	∠HNH	Φ_{NH2}
X=NO_2	1.225	1.226	124.6					
CN				1.156				
NH_2					1.009	1.009	113.3	347.0

Table S10. Structural parameters of Y-R-X systems, where R= polyene, Y = O^-. Tables with X = NO_2, CN and NH_2 rows contain the parameters of the X groups.

Y = O^-, R=polyene, *n*=1			
	d_{CY}	d_{CX}	C1-C2
unsub.			1.329
X=NO_2	1.246	1.380	1.410
CN	1.250	1.400	1.405
Cl	1.268	1.814	1.376
H	1.267		1.384
OH	1.277	1.436	1.367
NH_2	1.270	1.459	1.381

Y = O^-, R=polyene, *n*=1								
	d_{NO1}	d_{NO2}	∠ONO	d_{CN}	d_{NH1}	d_{NH2}	∠HNH	Φ_{NH2}
X=NO_2	1.260	1.262	120.6					
CN				1.171				
NH_2					1.016	1.017	106.7	331.9

Y = O^-, R=polyene, *n*=2							
	d_{CY}	d_{CX}	C1-C2	C2-C3	C3-C4	HOMA	BLA
unsub.			1.338	1.456	1.338	0.177	0.0589
X=NO_2	1.238	1.388	1.424	1.386	1.387	0.891	0.0126
CN	1.243	1.403	1.415	1.395	1.387	0.932	0.0106
Cl	1.253	1.796	1.403	1.419	1.355	0.805	0.0265
H	1.256		1.401	1.420	1.366	0.858	0.0205
OH	1.260	1.417	1.395	1.434	1.350	0.689	0.0291
NH_2	1.258	1.452	1.398	1.427	1.359	0.788	0.0244

Y = O^-, R=polyene, *n*=2								
	d_{NO1}	d_{NO2}	∠ONO	d_{CN}	d_{NH1}	d_{NH2}	∠HNH	Φ_{NH2}
X=NO_2	1.260	1.256	121.3					
CN				1.168				
NH_2					1.020	1.015	106.6	329.1

Y = O^-, R=polyene, *n*=3									
	d_{CY}	d_{CX}	C1-C2	C2-C3	C3-C4	C4-C5	C5-C6	HOMA	BLA
unsub.			1.341	1.449	1.350	1.449	1.341	0.315	0.0504
X=NO_2	1.235	1.393	1.427	1.382	1.403	1.393	1.382	0.904	0.0142
CN	1.240	1.405	1.421	1.389	1.396	1.402	1.383	0.929	0.0107

Cl	1.246	1.784	1.412	1.401	1.384	1.425	1.353	0.827	0.0212
H	1.249		1.410	1.404	1.382	1.427	1.361	0.845	0.0202
OH	1.252	1.403	1.406	1.411	1.377	1.438	1.348	0.740	0.0268
NH_2	1.252	1.438	1.407	1.410	1.378	1.435	1.354	0.774	0.0249

Y = O^-, R=polyene, *n*=3								
	d_{NO1}	d_{NO2}	∠ONO	d_{CN}	d_{NH1}	d_{NH2}	∠HNH	Φ_{NH2}
X=NO_2	1.254	1.257	121.7					
CN				1.167				
NH_2					1.018	1.015	107.4	330.1

Y = O^-, R=polyene, *n*=4											
	d_{CY}	d_{CX}	C1-C2	C2-C3	C3-C4	C4-C5	C5-C6	C6-C7	C7-C8	HOMA	BLA
unsub.			1.342	1.446	1.353	1.440	1.353	1.446	1.342	0.404	0.0473
X=NO_2	1.233	1.397	1.430	1.379	1.406	1.389	1.398	1.396	1.379	0.910	0.0126
CN	1.237	1.406	1.425	1.384	1.401	1.396	1.391	1.406	1.380	0.926	0.0111
Cl	1.242	1.778	1.418	1.393	1.392	1.408	1.379	1.427	1.351	0.839	0.0191
H	1.244		1.416	1.396	1.390	1.411	1.377	1.430	1.358	0.846	0.0190
OH	1.246	1.395	1.413	1.400	1.387	1.417	1.372	1.439	1.347	0.774	0.0238
NH_2	1.247	1.429	1.413	1.401	1.386	1.418	1.372	1.438	1.352	0.790	0.0231

Y = O^-, R=polyene, *n*=4								
	d_{NO1}	d_{NO2}	∠ONO	d_{CN}	d_{NH1}	d_{NH2}	∠HNH	Φ_{NH2}
X=NO_2	1.252	1.255	121.9					
CN				1.166				
NH_2					1.016	1.014	108.2	332.2

Y = O^-, R=polyene, *n*=5													
	d_{CY}	d_{CX}	C1-C2	C2-C3	C3-C4	C4-C5	C5-C6	C6-C7	C7-C8	C8-C9	C9-C10	HOMA	BLA
unsub.			1.342	1.446	1.355	1.437	1.357	1.437	1.355	1.446	1.342	0.464	0.0449
X=NO_2	1.232	1.400	1.432	1.377	1.409	1.386	1.401	1.392	1.395	1.399	1.376	0.914	0.0124
CN	1.235	1.407	1.428	1.381	1.404	1.392	1.396	1.399	1.388	1.408	1.377	0.924	0.0114
Cl	1.239	1.773	1.422	1.388	1.397	1.401	1.387	1.412	1.376	1.430	1.349	0.846	0.0184
H	1.241		1.420	1.390	1.395	1.404	1.385	1.415	1.374	1.432	1.355	0.847	0.0187
OH	1.242	1.389	1.418	1.393	1.393	1.407	1.382	1.421	1.369	1.440	1.347	0.794	0.0221
NH_2	1.243	1.422	1.417	1.395	1.392	1.409	1.381	1.422	1.369	1.439	1.351	0.805	0.0218

Y = O^-, R=polyene, *n*=5								
	d_{NO1}	d_{NO2}	∠ONO	d_{CN}	d_{NH1}	d_{NH2}	∠HNH	Φ_{NH2}
X=NO_2	1.250	1.254	122.1					
CN				1.165				
NH_2					1.015	1.014	108.9	334.0

Table S11. Structural parameters of Y-R-X systems, where R= polyyne, Y = O^-. Tables with X = NO_2, CN and NH_2 rows contain the parameters of the X groups.

Y = O^-, R=polyyne, *n*=1			
	d_{CY}	d_{CX}	C1-C2
unsub.			1.199
X=NO_2	1.194	1.372	1.273
CN	1.208	1.344	1.246
Cl	1.211	1.779	1.278
H	1.223		1.254
OH	1.224	1.422	1.271
NH_2	1.232	1.432	1.255

Y = O^-, R=polyyne, *n*=1								
	d_{NO1}	d_{NO2}	∠ONO	d_{CN}	d_{NH1}	d_{NH2}	∠HNH	Φ_{NH2}
X=NO_2	1.255	1.260	120.2					
CN				1.177				
NH_2					1.014	1.022	105.9	327.5

Y = O^-, R=polyyne, *n*=2							
	d_{CY}	d_{CX}	C1-C2	C2-C3	C3-C4	HOMA	BLA
unsub.			1.207	1.365	1.207	−4.657	0.070
X=NO_2	1.198	1.347	1.254	1.321	1.233	−2.976	0.034
CN	1.201	1.346	1.253	1.322	1.240	−2.816	0.034
Cl	1.211	1.688	1.248	1.339	1.234	−2.906	0.044
H	1.214		1.247	1.346	1.228	−3.063	0.048
OH	1.217	1.364	1.247	1.346	1.236	−2.852	0.047
NH_2	1.220	1.380	1.245	1.354	1.224	−3.175	0.053

Y = O^-, R=polyyne, *n*=2								
	d_{NO1}	d_{NO2}	∠ONO	d_{CN}	d_{NH1}	d_{NH2}	∠HNH	Φ_{NH2}
X=NO_2	1.255	1.255	121.9					
CN				1.174				
NH_2					1.017	1.017	108.3	334.7

Y = O^-, R=polyyne, *n*=3									
	d_{CY}	d_{CX}	C1-C2	C2-C3	C3-C4	C4-C5	C5-C6	HOMA	BLA
unsub.			1.210	1.356	1.218	1.356	1.210	−3.876	0.069
X=NO_2	1.194	1.351	1.258	1.312	1.248	1.323	1.231	−2.667	0.034
CN	1.196	1.347	1.257	1.314	1.248	1.324	1.237	−2.567	0.034
Cl	1.205	1.663	1.252	1.327	1.240	1.344	1.223	−2.767	0.046
H	1.205		1.252	1.328	1.240	1.346	1.224	−2.747	0.047

OH	1.209	1.336	1.250	1.333	1.238	1.352	1.219	−2.824	0.051
NH_2	1.210	1.368	1.250	1.333	1.239	1.351	1.222	−2.775	0.051

Y = O^-, R=polyyne, *n*=3								
	d_{NO1}	d_{NO2}	∠ONO	d_{CN}	d_{NH1}	d_{NH2}	∠HNH	Φ_{NH2}
X=NO_2	1.251	1.251	122.7					
CN				1.172				
NH_2					1.015	1.015	109.7	338.1

Y = O^-, R=polyyne, *n*=4										
	d_{CY}	d_{CX}	C1-C2	C2-C3	C3-C4	C4-C5	C5-C6	C6-C7	C7-C8	HOMA	BLA
unsub.			1.211	1.353	1.221	1.344	1.221	1.353	1.211	−3.518	0.066
X=NO_2	1.191	1.354	1.260	1.307	1.253	1.314	1.246	1.325	1.229	−2.539	0.033
CN	1.193	1.348	1.259	1.309	1.252	1.315	1.246	1.325	1.235	−2.474	0.034
Cl	1.199	1.656	1.255	1.318	1.246	1.328	1.238	1.345	1.220	−2.632	0.044
H	1.200		1.255	1.319	1.246	1.329	1.237	1.347	1.221	−2.619	0.045
OH	1.203	1.327	1.254	1.322	1.245	1.333	1.235	1.352	1.216	−2.686	0.048
NH_2	1.203	1.359	1.254	1.323	1.245	1.333	1.236	1.349	1.221	−2.619	0.047

Y = O^-, R=polyyne, *n*=4								
	d_{NO1}	d_{NO2}	∠ONO	d_{CN}	d_{NH1}	d_{NH2}	∠HNH	Φ_{NH2}
X=NO_2	1.248	1.248	123.3					
CN				1.170				
NH_2					1.014	1.014	110.8	340.7

Y = O^-, R=polyyne, n=5													
	d_{CY}	d_{CX}	C1-C2	C2-C3	C3-C4	C4-C5	C5-C6	C6-C7	C7-C8	C8-C9	C9-C10	HOMA	BLA
unsub.			1.211	1.351	1.223	1.341	1.226	1.341	1.223	1.351	1.211	−3.312	0.063
X=NO_2	1.189	1.357	1.261	1.304	1.255	1.309	1.251	1.315	1.244	1.326	1.227	−2.484	0.032
CN	1.190	1.350	1.261	1.305	1.254	1.311	1.250	1.317	1.244	1.326	1.233	−2.428	0.033
Cl	1.196	1.652	1.257	1.313	1.250	1.320	1.244	1.329	1.236	1.345	1.219	−2.559	0.042
H	1.196		1.257	1.313	1.250	1.320	1.244	1.329	1.235	1.347	1.219	−2.552	0.043
OH	1.198	1.321	1.256	1.316	1.248	1.323	1.242	1.333	1.233	1.351	1.215	−2.601	0.045
NH_2	1.199	1.354	1.256	1.316	1.248	1.324	1.243	1.333	1.235	1.348	1.220	−2.540	0.044

Y = O^-, R=polyyne, n=5								
	d_{NO1}	d_{NO2}	∠ONO	d_{CN}	d_{NH1}	d_{NH2}	∠HNH	Φ_{NH2}
X=NO_2	1.246	1.246	123.7					
CN				1.169				
NH_2					1.013	1.013	111.5	342.6

Table S12. Structural parameters of Y-R-X systems, where R= acene, Y = O^-. Tables with X = NO_2, CN and NH_2 rows contain the parameters of the X groups.

Y = O^-, R=acene, n=1			
	d_{CY}	d_{CX}	HOMA(I)
unsub.			0.989
X=NO_2	1.253	1.412	0.514
CN	1.258	1.413	0.557
Cl	1.267	1.793	0.698
H	1.269		0.683
OH	1.273	1.411	0.730
NH_2	1.270	1.455	0.695

Y = O^{-}, R=acene, *n*=1								
	d_{NO1}	d_{NO2}	∠ONO	d_{CN}	d_{NH1}	d_{NH2}	∠HNH	Φ_{NH2}
X=NO_2	1.251	1.251	121.4					
CN				1.165				
NH2					1.017	1.017	106.6	329.0

Y = O^{-}, R=acene, *n*=2				
	d_{CY}	d_{CX}	HOMA(I)	HOMA(II)
unsub.			0.784	0.783
X=NO_2	1.254	1.426	0.316	0.648
CN	1.257	1.418	0.369	0.649
Cl	1.263	1.785	0.442	0.732
H	1.265		0.443	0.719
OH	1.267	1.397	0.482	0.741
NH_2	1.267	1.435	0.487	0.735

Y = O^{-}, R=acene, *n*=2								
	d_{NO1}	d_{NO2}	∠ONO	d_{CN}	d_{NH1}	d_{NH2}	∠HNH	Φ_{NH2}
X=NO_2	1.246	1.247	122.1					
CN				1.162				
NH_2					1.015	1.013	108.1	331.0

Y = O^{-}, R=acene, *n*=3					
	d_{CY}	d_{CX}	HOMA(I)	HOMA(II)	HOMA(III)
unsub.			0.630	0.721	0.629
X=NO_2	1.253	1.432	0.203	0.509	0.617

CN	1.256	1.421	0.226	0.536	0.601
Cl	1.260	1.780	0.265	0.586	0.654
H	1.262		0.272	0.589	0.643
OH	1.263	1.390	0.296	0.615	0.648
NH_2	1.263	1.425	0.299	0.618	0.642

Y = O^-, R=acene, *n*=3								
	d_{NO1}	d_{NO2}	∠ONO	d_{CN}	d_{NH1}	d_{NH2}	∠HNH	Φ_{NH2}
X=NO_2	1.244	1.243	122.5					
CN				1.161				
NH_2					1.013	1.013	109.0	333.7

Y = O^-, R=acene, *n*=4						
	d_{CY}	d_{CX}	HOMA(I)	HOMA(II)	HOMA(III)	HOMA(IV)
unsub.			0.538	0.632	0.632	0.538
X=NO_2	1.252	1.437	0.130	0.432	0.521	0.582
CN	1.255	1.422	0.141	0.443	0.539	0.558
Cl	1.258	1.775	0.163	0.471	0.579	0.593
H	1.259		0.170	0.476	0.580	0.582
OH	1.260	1.385	0.181	0.490	0.596	0.583
NH_2	1.261	1.419	0.189	0.495	0.600	0.573

Y = O^-, R=acene, *n*=4								
	d_{NO1}	d_{NO2}	∠ONO	d_{CN}	d_{NH1}	d_{NH2}	∠HNH	Φ_{NH2}
X=NO_2	1.242	1.242	122.7					
CN				1.160				
NH_2					1.012	1.012	109.7	335.6

Table S13. Statistical data on the SESE = $a{\cdot}\sigma_p(^{-}) + b$ correlations in Y-R-X systems (Y= O^- and X= NO_2. CN. Cl. H. OH. NH2), where n is the number of repeatable units in the transmitter R. Slopes a, their standard error (se) and determination coefficients r^2, %shortest and %benz indicate how the slopes relate to the n=1 and the benzene system (in %).

		SESE vs σ_p Y = O^-				SESE vs σ_p^- Y = O^-			
R	n	a ± se	r^2	% shortest	% benz	a ± se	r^2	% shortest	% benz
Polyene	1	35.23±6.22	0.889	100	155	30.14±1.98	0.983	100	157
	2	28.46±4.51	0.909	81	125	23.84±2.34	0.963	79	124
	3	23.73±3.53	0.919	67	104	19.67±2.17	0.953	65	103
	4	20.30±2.90	0.925	58	89	16.72±1.98	0.947	55	87
	5	18.05±2.71	0.917	51	79	15.01±1.57	0.958	50	78
Polyyne	1	42.46±6.37	0.918	100	187	35.39±3.49	0.963	100	185
	2	30.95±5.37	0.893	73	136	26.18±2.51	0.964	74	137
	3	24.52±4.30	0.890	58	108	20.69±2.16	0.958	58	108
	4	20.39±3.40	0.900	48	90	17.09±1.85	0.955	48	89
	5	17.20±3.11	0.884	41	76	14.54±1.59	0.955	41	76
Acene	1	22.75±3.73	0.903	100	100	19.17±1.75	0.968	100	100
	2	16.64±2.54	0.915	73	73	13.82±1.52	0.954	72	72
	3	13.05±1.96	0.917	57	57	10.80±1.26	0.948	56	56
	4	10.70±1.58	0.920	47	47	8.82±1.08	0.944	46	46

Table S14. The obtained determination coefficients and slopes (± estimated standard deviation) of the relations between SESE and d_{CX} bond lengths (bond between the X substituent and the carbon atom to which it is attached) in Y-R-X systems (Y = NO_2, R and X fixed, n varies).

**R**	SESE vs. d_{CN} X=NO_2	SESE vs. d_{CC} X=CN	SESE vs. d_{CCl} X=Cl	SESE vs. d_{CO} X=OH	SESE vs. d_{CN} X=NH_2
Polyene	0.910<br>−304.0 ± 55.1<br>0.991<br>−164.6 ± 10.9	0.878<br>−972.9 ± 209.8<br>0.990<br>−629.7 ± 45.5	0.836<br>172.6 ± 44.2<br>0.999<br>22.65 ± 0.4	0.082<br>45.6 ±88.3<br>0.991<br>−197.0 ± 13.2	0.953<br>−256.9 ± 32.9<br>0.999<br>−332.5±7.3
Polyyne	0.916<br>−203.8 ± 35.8<br>0.778<br>−81.2±106.1	0.971<br>−245.6 ± 24.4<br>0.933<br>−356.4±67.7	0.011<br>85 ± 466<br>0.947<br>−4777 ± 802	0.889<br>−25.3±168.3<br>0.941<br>−82.5 ± 120.0	0.513<br>−1785±1004<br>0.928<br>−356.4±267.6
Acene	0.995<br>−261.9 ± 13.4	0.989<br>−998.2 ± 74.3	0.892<br>92.3 ± 22.7	0.999<br>−128.7 ± 3.3	0.997<br>−206.0 ± 8.7

Green values – the shorter transmitter is not taken into account.

Table S15. The obtained determination coefficients and slopes (± estimated standard deviation) of the relations between SESE and d_{CX} bond lengths (bond between the X substituent and the carbon atom to which it is attached) in Y-R-X systems (Y = O^{-}, R and X fixed, n varies).

**R**	SESE vs. d_{CN} X=NO_2	SESE vs. d_{CC} X=CN	SESE vs. d_{CCl} X=Cl	SESE vs. d_{CO} X=OH	SESE vs. d_{CN} X=NH_2
Polyene	0.993<br>−994.5 ± 48.6<br>0.991<br>−1085.5±70.9	0.988<br>−2318.2 ± 150.0<br>0.980<br>−2047.9 ± 206.2	0.976<br>101.9 ± 9.3<br>1.000<br>127.8 ± 1.4	0.840<br>−96.7 ± 24.4<br>0.557<br>−40.7 ± 25.7	0.969<br>−71.2 ± 7.4<br>0.941<br>−70.0 ± 12.5
Polyyne	0.309<br>545.8 ± 471.5<br>0.984<br>−1248.2±110.7	0.992<br>−3805.4 ± 202.9<br>0.980<br>−3479.1±354.9	0.995<br>44.9 ± 1.9<br>0.874<br>38.9 ± 10.4	0.979<br>−64.9 ± 5.5<br>0.985<br>−84.6 ± 7.5	0.969<br>−109.0 ± 11.3<br>0.990<br>−169.4 ± 11.9
Acene	0.999<br>−548.1 ± 12.2	0.995<br>−1140.1 ± 54.6	0.996<br>178.4 ± 7.8	0.980<br>−98.9 ± 10.1	0.993<br>−76.5 ± 4.5

Green values – the shortest (n=1) transmitter is not taken into account.

Table S16. The obtained determination coefficients and slopes (± estimated standard deviation) of the relations between d_{CY} and d_{CX} bond lengths (bonds between the Y or X groups and the carbon atom to which they are attached) in Y-R-X systems (Y, R and X fixed, n varies).

**R**		d_{CY} vs. d_{CN} X=NO_2	d_{CY} vs. d_{CC} X=CN	d_{CY} vs. d_{CCl} X=Cl	d_{CY} vs. d_{CO} X=OH	d_{CY} vs. d_{CN} X=NH_2
Polyene	Y = NO_2	1.00<br>1.000 ±0.000<br>1.00<br>1.000 ±0.000	0.926<br>6.075 ± 0.989<br>0.993<br>4.466 ± 0.272	0.997<br>−0.981 ± 0.034<br>0.999<br>−1.089 ± 0.030	0.252<br>−0.156 ± 0.156<br>0.996<br>−0.584 ± 0.025	0.761<br>0.433 ± 0.140<br>0.882<br>0.111 ± 0.029
	Y = O^{-}	0.974<br>−0.713 ± 0.067<br>0.997<br>−0.545 ± 0.023	0.997<br>−2.072 ± 0.063<br>0.995<br>−1.965 ± 0.094	0.989<br>0.670 ± 0.040<br>0.993<br>0.566 ± 0.034	0.992<br>0.732 ± 0.039<br>0.999<br>0.632 ± 0.011	0.938<br>0.653 ± 0.097<br>0.998<br>0.494 ± 0.016
Polyyne	Y = NO_2	1.00<br>1.001 ± 0.002<br>1.00<br>1.009 ± 0.005	0.982<br>1.415 ± 0.111<br>0.990<br>1.907 ± 0.138	0.777<br>3.628 ± 1.121<br>0.668<br>−7.768 ± 3.870	0.091<br>−0.506 ± 0.921<br>0.262<br>0.282 ± 0.334	0.712<br>2.130 ± 0.782<br>0.946<br>1.842 ± 0.313
	Y = O^{-}	0.092<br>−0.116 ± 0.210<br>0.985<br>−0.918 ± 0.079	0.997<br>−3.052 ± 0.097<br>0.991<br>−2.993 ±0.201	0.549<br>0.097 ± 0.051<br>0.901<br>0.393 ± 0.092	0.881<br>0.237 ± 0.050<br>0.944<br>0.417 ± 0.072	0.895<br>0.402 ± 0.080<br>0.999<br>0.815 ± 0.007
Acene	Y = NO_2	1.00<br>0.982 ± 0.011	0.990<br>4.357 ± 0.308	0.557<br>−0.416 ± 0.262	0.778<br>0.567 ± 0.214	0.979<br>0.876 ± 0.090
	Y = O^{-}	0.404<br>−0.036 ± 0.031	0.837<br>−0.338 ± 0.106	0.998<br>0.515 ± 0.017	0.999<br>0.508 ± 0.011	0.910<br>0.250 ± 0.055

Green values – the shortest (n=1) transmitter is not taken into account.

Table S17. The transmission coefficient, γ, in Y-R-X systems on the basis of the relations: d_{CY} = a·SESE + b, where R = polyyne, polyene and acene.

	$Y=NO_2$			$Y=O^-$		
n\R	polyyne	polyene	acene	polyyne	polyene	acene
	SESE					
2	0.607	0.706	1.0	0.858	0.923	1.0
3	0.585	0.712	0.854	0.818	0.862	0.991
4	0.562	0.715	0.816	0.764	0.819	0.938
5	0.506	0.725	0.793	0.726	0.775	0.926

Table S18. The obtained determination coefficients and slopes (± estimated standard deviation) for the relations between geometric parameters of the nitro group in Y = NO_2 systems.

**R**	n	d_{NO1} vs. d_{CN} $Y=NO_2$	d_{NO2} vs. d_{CN} $Y=NO_2$	ONO angle vs. d_{CN} $Y=NO_2$
Polyene	1	0.970<br>−0.295 ± 0.026	0.859<br>−0.207 ± 0.042	0.852<br>44.95 ± 9.37
	2	0.999<br>−0.356 ± 0.006	0.991<br>−0.321 ± 0.015	0.979<br>59.05 ± 4.36
	3	0.999<br>−0.360 ± 0.005	0.999<br>−0.358 ± 0.007	0.993<br>59.40 ± 2.43
	4	0.908<br>−0.408 ± 0.065	0.858<br>−0.335 ± 0.068	0.997<br>58.43 ± 1.70
	5	0.999<br>−0.368 ± 0.004	0.999<br>−0.390 ± 0.007	0.998<br>57.99 ± 1.24
Polyyne	1	0.929<br>−0.382 ± 0.053	0.936<br>−0.370 ± 0.048	0.731<br>66.64 ± 20.23
	2	0.958<br>−0.425 ± 0.045	0.966<br>−0.418 ± 0.039	0.827<br>70.96 ± 16.23
	3	0.972<br>−0.446 ± 0.038	0.974<br>−0.443 ± 0.036	0.865<br>74.56 ± 14.73
	4	0.989<br>−0.451 ± 0.024	0.982<br>−0.458 ± 0.031	0.908<br>77.30 ± 12.28
	5	0.987<br>−0.476 ± 0.027	0.989<br>−0.474 ± 0.025	0.930<br>81.26 ± 11.12
Acene	1	0.967<br>−0.243 ± 0.023	0.977<br>−0.250 ± 0.019	0.911<br>41.12 ± 6.44
	2	0.992<br>−0.289 ± 0.013	0.981<br>−0.271 ± 0.019	0.959<br>47.24 ± 4.90
	3	0.996<br>−0.314 ± 0.010	0.998<br>−0.328 ± 0.007	0.990<br>54.02 ± 2.76
	4	0.998<br>−0.331 ± 0.008	0.998<br>−0.336 ± 0.007	0.993<br>55.08 ± 2.30

Table S19. The obtained determination coefficients and slopes (± estimated standard deviation) of the linear dependence of P(X) = SESE, d_{CN}, ∠ONO or d_{CO} on the pyramidalization of the amino group, Φ_{NH2}, in X = NH_2 derivatives (Y, R, X fixed, *n* varies).

R	SESE vs. Φ_{NH2} Y=NO_2	SESE vs. Φ_{NH2} Y=O^-	Φ_{NH2} vs. d_{CN} Y=NO_2	Φ_{NH2} vs. ONO angle Y=NO_2	Φ_{NH2} vs. d_{CO} Y=O^-
Polyene	0.996<br>0.740 ± 0.026<br>0.999<br>0.707 ± 0.014	0.388<br>0.367 ± 0.266<br>0.996<br>0.430 ± 0.020	0.501<br>−505.6 ± 291.3<br>0.887<br>−3751 ± 948	0.995<br>−36.64 ± 1.51<br>0.998<br>−38.66 ± 1.13	0.157<br>−71.9 ± 96.4<br>0.938<br>−328.3 ± 60.0
Polyyne	0.523<br>3.716 ± 2.047<br>0.704<br>2.520 ± 1.157	0.997<br>0.590 ± 0.017<br>0.996<br>0.578 ±0.026	0.670<br>−157.2 ± 63.8<br>0.733<br>−211.8 ± 90.4	0.556<br>−2.063 ± 1.064<br>0.712<br>−3.647 ± 1.642	0.981<br>−436.2 ± 35.1<br>0.995<br>−359.5 ± 18.6
Acene	0.996<br>0.493 ± 0.023	0.954<br>0.408 ± 0.063	0.981<br>−466.7 ± 45.8	0.997<br>−30.67 ± 1.11	1.0<br>−703.7 ± 4.6

Green values – the shortest (*n*=1) transmitter is not taken into account.

Table S20. The obtained determination coefficients and slopes (± estimated standard deviation) of the relations between SESE and HOMA or BLA. In the case of acene derivatives, the individual rings and the π-electron delocalization paths were taken into account. Values of r^2 > 0.8 in bold.

		SESE vs. HOMA Y=NO_2		SESE vs. HOMA Y=O^-		SESE vs. BLA Y=NO_2		SESE vs. BLA Y=O^-	
**R**	***n***	*a* ±err	r^2	*a* ±err	r^2	*a* ±err	r^2	*a* ±err	r^2
**Polyene**	1	13.6 ±5.7	0.591	−206.2 ±172.7	0.263				
	2	19.5 ±5.7	0.748	143.0 ±59.8	0.589	−480.2 ±138.6	0.750	−1813.4 ±551.0	0.730
	3	21.2 ±7.0	0.696	162.7 ±41.9	0.790	−465.6 ±152.2	0.700	−1937.5 ±474.5	**0.807**
	4	21.5 ±8.7	0.605	170.3 ±35.4	**0.853**	−446.6 ±173.9	0.622	−2023.9 ±371.2	**0.881**
	5	21.0 ±9.7	0.541	178.8 ±28.4	**0.908**	−425.0 ±184.4	0.570	−2124.0 ±308.9	**0.922**
**Polyyne**	1	7.3 ±4.4	0.405	−2 ±14	0.004				
	2	12.6 ±6.8	0.460	54 ±59	0.172	−566.8 ±363.4	0.378	−2137.3 ±336.6	**0.909**
	3	14.9 ±7.7	0.482	131.6 ±36.7	0.762	−502.9 ±342.7	0.350	−1804.4 ±131.5	**0.979**
	4	15.4 ±8.2	0.467	131.4 ±39.1	0.738	−477.7 ±338.6	0.332	−1700.9 ±151.3	**0.969**
	5	14.0 ±9.2	0.367	133.7 ±43.9	0.699	−422.8 ±336.3	0.283	−1660.5 ±150.7	**0.968**

Table S20. Continued.

			SESE vs. HOMA $Y=NO_2$		SESE vs. HOMA $Y=O^-$	
**R**	***n***		*a*±err	r^2	*a*±err	r^2
Acene	1		−115.5 ±60.2	0.480	−138.5 ±22.8	**0.902**
	2	Ring(I)	−38 ±220	0.007	−136.2 ±12.6	**0.967**
		Ring(II)	−94.5 ±56.2	0.414	−200.5 ±38.1	**0.874**
		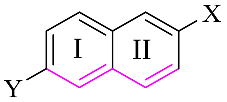 **path a**	−22 ±47	0.052	−145.7 ±29.3	**0.861**
		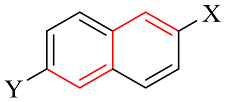 **path b**	71.2 ±4.6	0.390	−403.2 ±55.5	**0.930**
		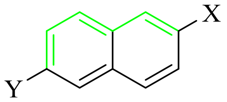 **path c**	27 ±101	0.018	−350.8 ±21.3	**0.985**
	3	Ring(I)	151.1 ±88.3	0.423	−188.1 ±13.8	**0.979**
		Ring(II)	−141.8 ±64.3	0.548	−164.9 ±13.1	**0.976**
		Ring(III)	−66.0 ±43.5	0.366	−276.6 ±111.3	0.607
		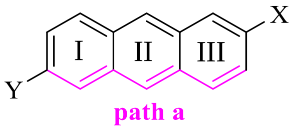 **path a**	−7 ±34	0.010	−158.5 ±39.9	0.798
		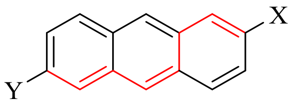 **path b**	36.7 ±34.9	0.216	−554.2 ±398.7	0.326
		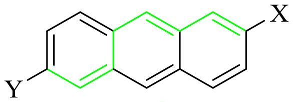 **path c**	37.3 ±33.9	0.232	−210 ±614	0.029
		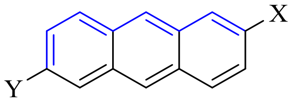 **path d**	7 ±47	0.006	−640.6 ±501.7	0.290
	4	Ring(I)	142.8 ±70.0	0.510	−259.5 ±15.1	**0.987**
		Ring(II)	−226.0 ±148.6	0.366	−234.4 ±15.5	**0.983**
		Ring(III)	−112.0 ±64.6	0.429	−185.1 ±14.8	**0.975**
		Ring(IV)	−46.2 ±28.5	0.395	−113 ±46	0.050
		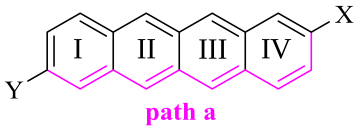 **path a**	−4 ±28	0.006	−163.4 ±46.7	0.754
		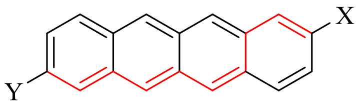 **path b**	23 ±35	0.103	−113 ±519	0.012

		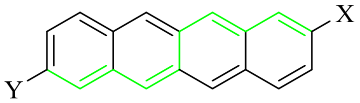 path c	25 ±30	0.146	451.4 ±251.7	0.446
		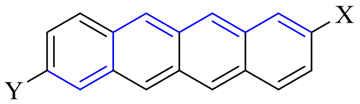 path d	18.7 ±31.7	0.080	413.7 ±281.5	0.351
		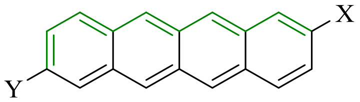 path e	−2 ±37	0.001	449.4 ±315.2	0.337

Table S21. The ranges of variation of HOMA and BLA indices for the Y-R-X systems (Y, R and *n* fixed, X varies).

	R=polyene		R=polyyne	
n	Y=NO_2	Y=O^-	Y=NO_2	Y=O^-
		HOMA		
2	0.428	0.243	0.467	0.359
3	0.273	0.189	0.310	0.257
4	0.202	0.152	0.231	0.212
5	0.162	0.130	0.185	0.173
		BLA		
2	0.0171	0.0185	0.0106	0.0198
3	0.0125	0.0161	0.0087	0.0167
4	0.0098	0.0127	0.0071	0.0149
5	0.0081	0.0107	0.0058	0.0130

					R=acene					
	Y=NO_2					Y=O^-				
					HOMA					
path	a	b	c	d	e	a	b	c	d	e
n										
2	0.041	0.039	0.019			0.136	0.061	0.070		
3	0.040	0.039	0.039	0.029		0.089	0.020	0.013	0.016	
4	0.036	0.030	0.035	0.034	0.027	0.068	0.014	0.023	0.023	0.020

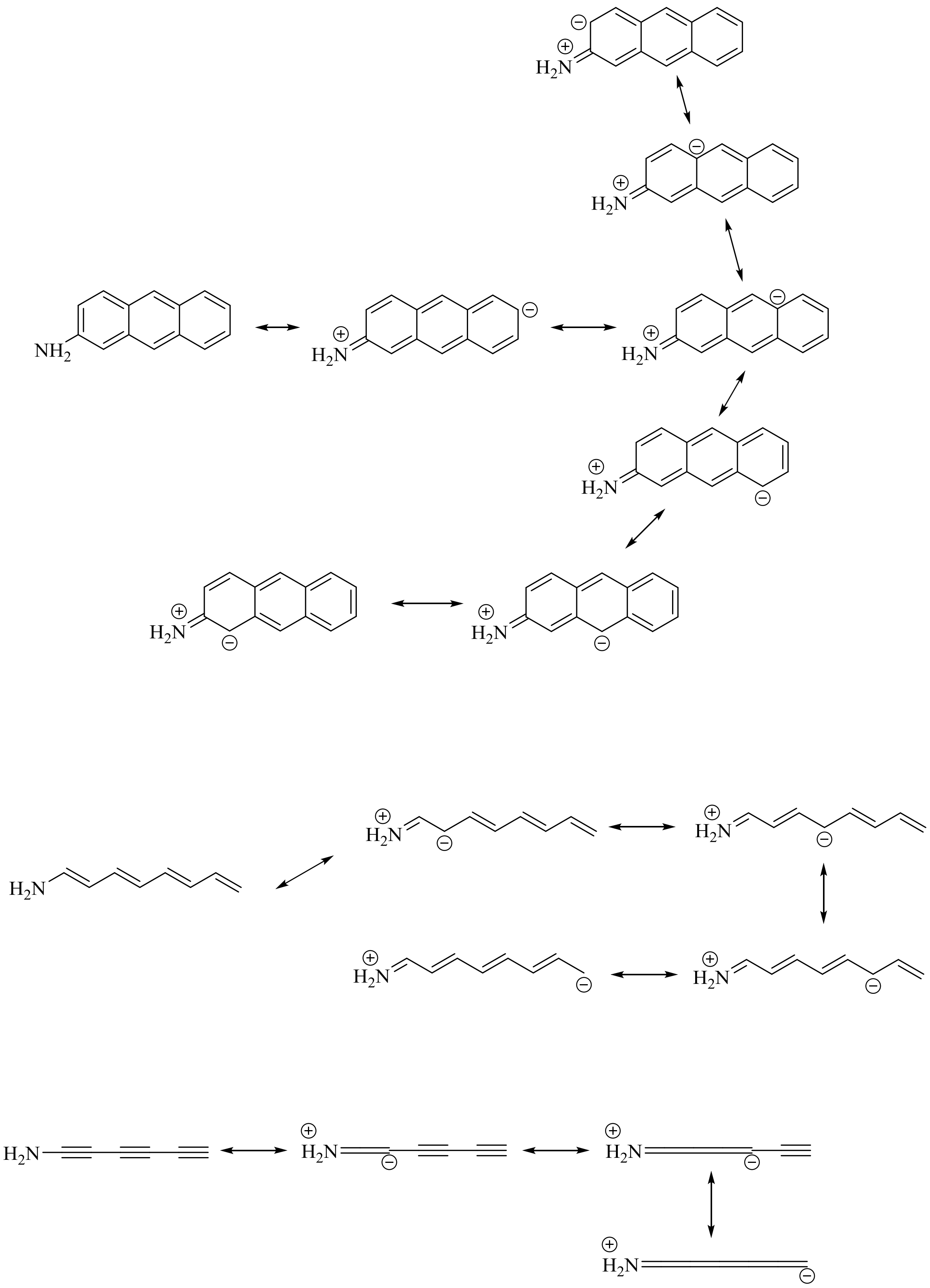

Scheme S1. Resonance structures in acene, polyene and polyyne derivatives. Examples for electron-donating NH_2 group in 2-aminoanthracene, 1-aminooctatetraene and 1-aminohexatriyne. In the case of electron-withdrawing NO_2 group, the structures are similar, but a positive charge instead of negative is delocalized, whereas NO_2 is negatively charged.

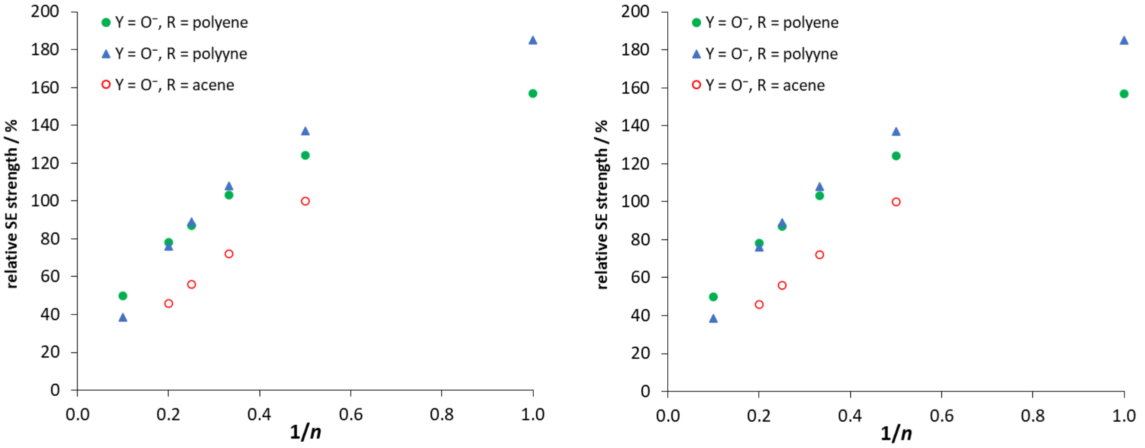

Figure S1. Relative strength of the SE as a function of the reciprocal of spacer length, 1/*n*.

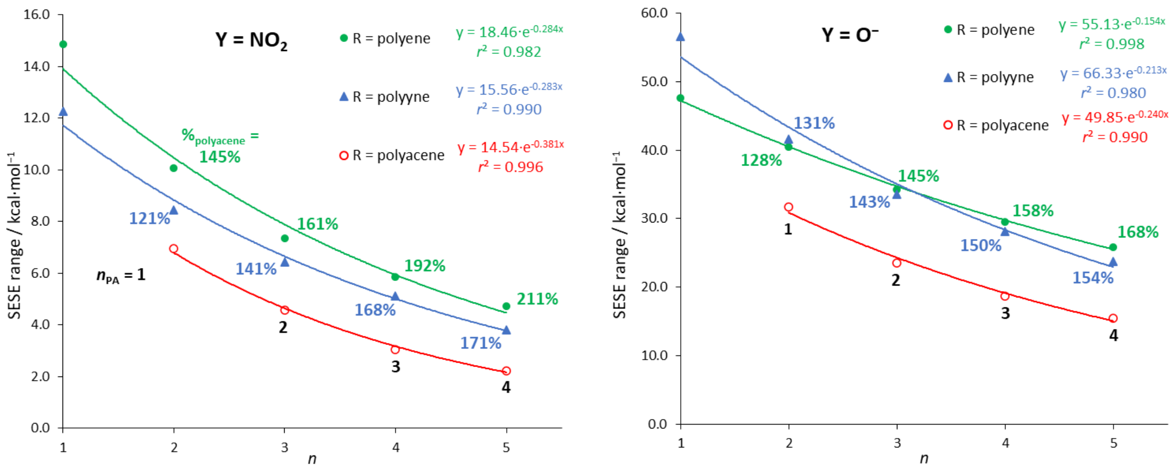

(a) (b)

Figure S2. Dependence of the range of variation of SESE on the length of the transmitting fragment for Y = NO_2 (a) and Y = O^- (b) Y-R-X derivatives (X = NO_2, CN, Cl, H, OH, NH_2). Percentages indicate the relative strength of the substituent effect in reference to the acene system with the same number of C-C bonds between Y and X (for example n_{PA} = 1 for n = 2 systems).

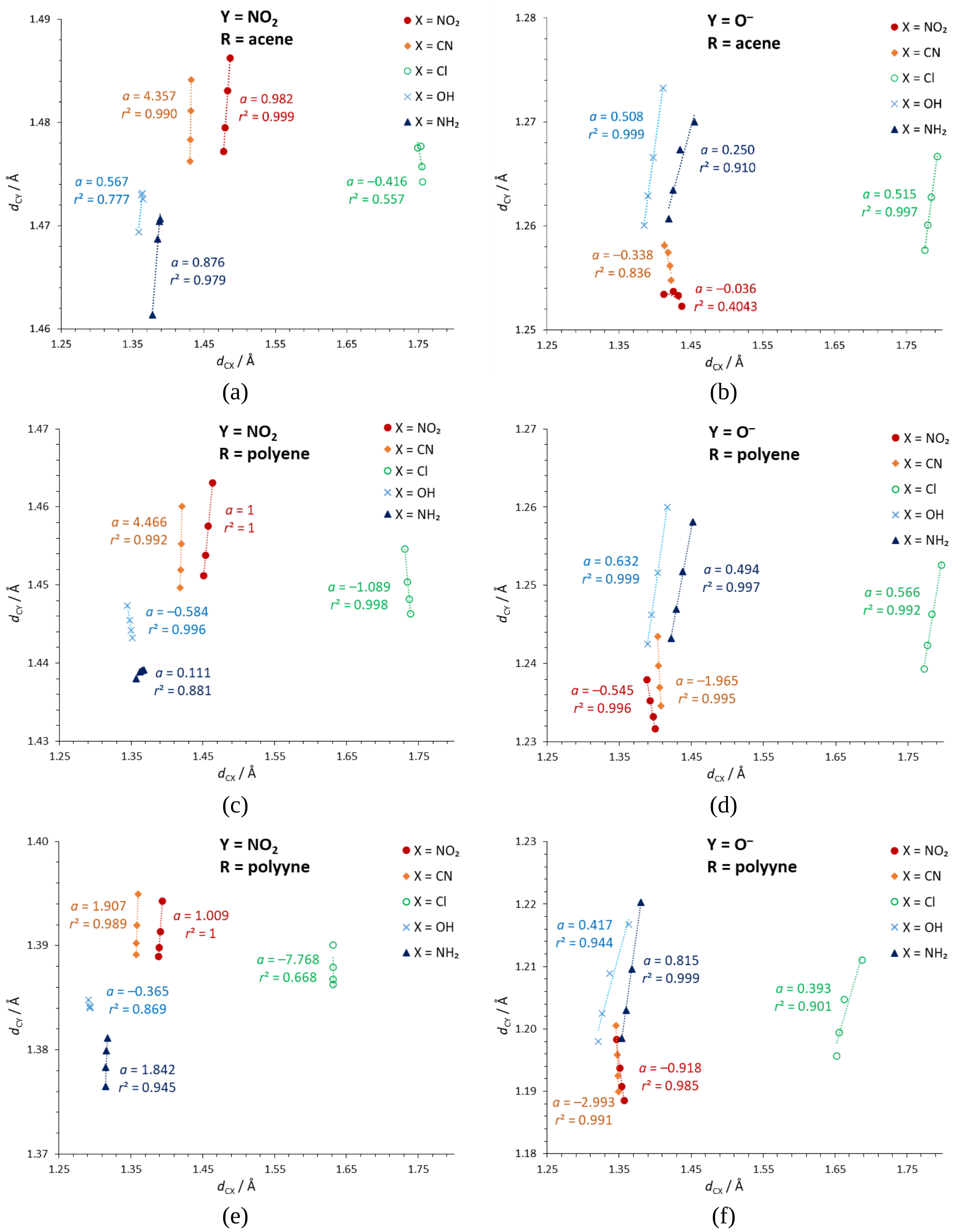

Figure S3. Relationships between C-Y and C-X bond lengths (d_{CY} and d_{CX}) for Y=NO_2 and O^-acene (a,b), polyene (c,d) and polyyne (e,f) systems (n=2-5, n=1-4 for acenes). Values of slopes (a) and r^2 of $d_{CY} = a{\cdot}d_{CX} + b$ correlation for each substituent X are included.

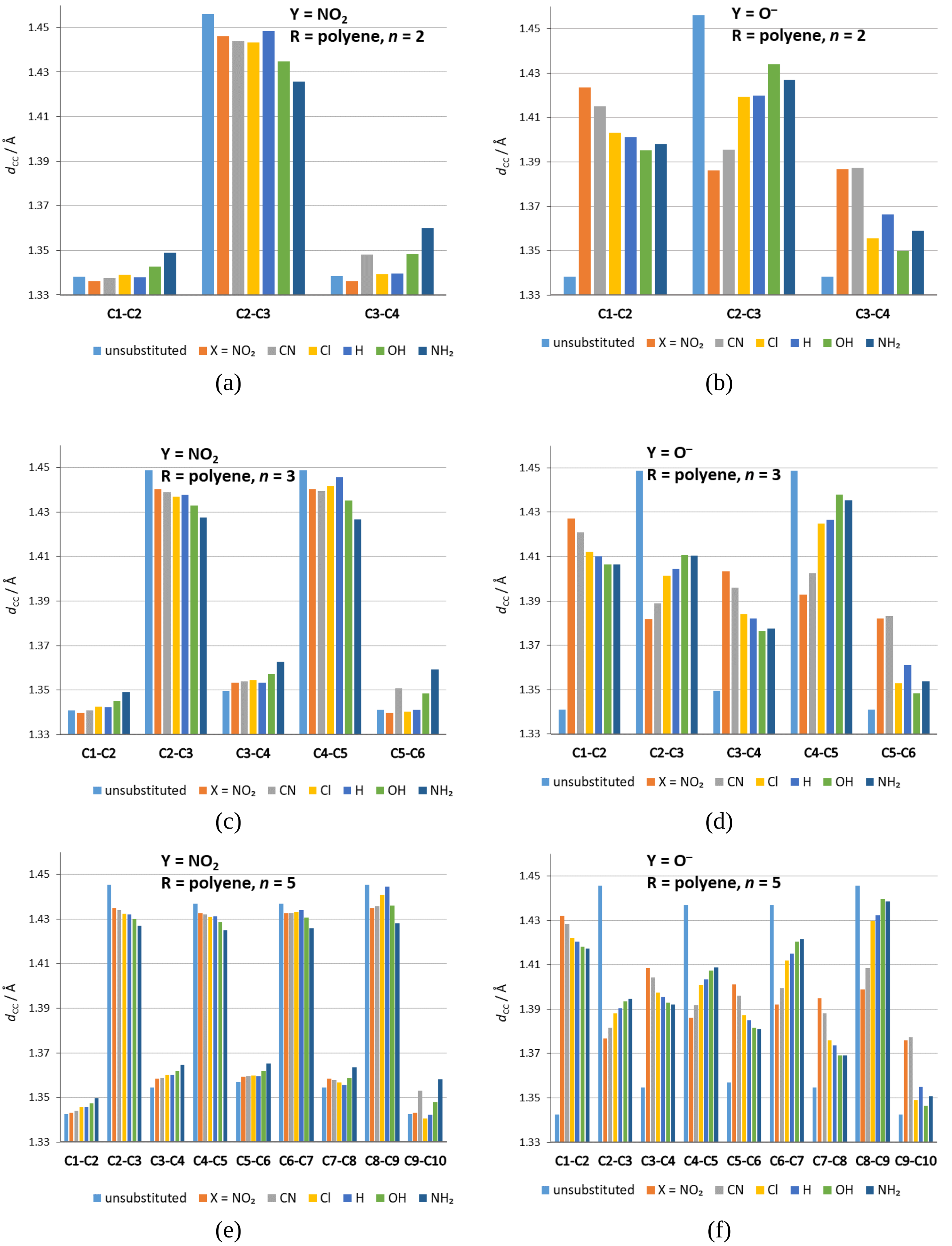

Figure S4. Lengths of CC bonds within the spacers R for each X derivative of six polyene R-Y systems. First carbon atom (C1) is the one connected to the Y group, while the last (C8 or C10) to the X group.

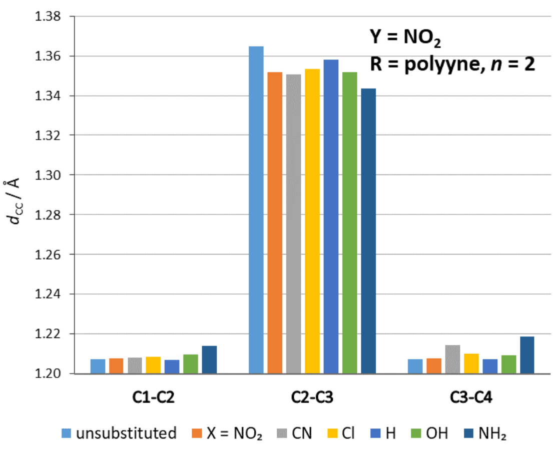

(a)

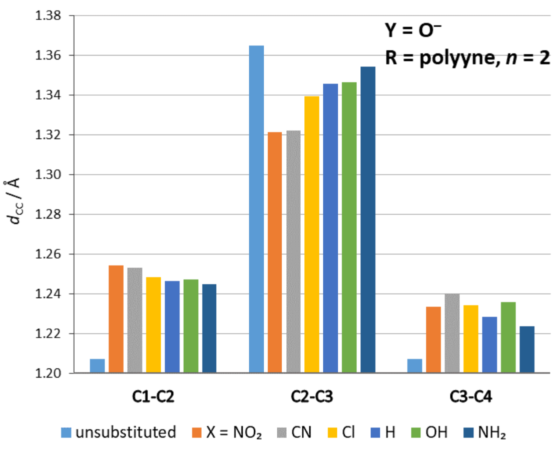

(b)

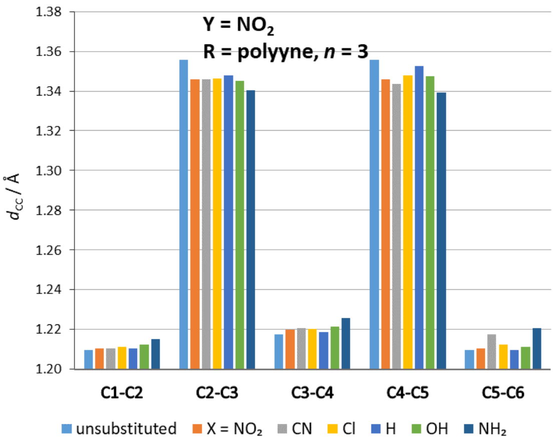

(c)

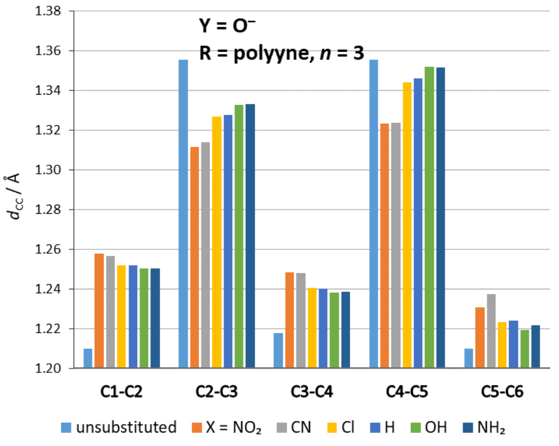

(d)

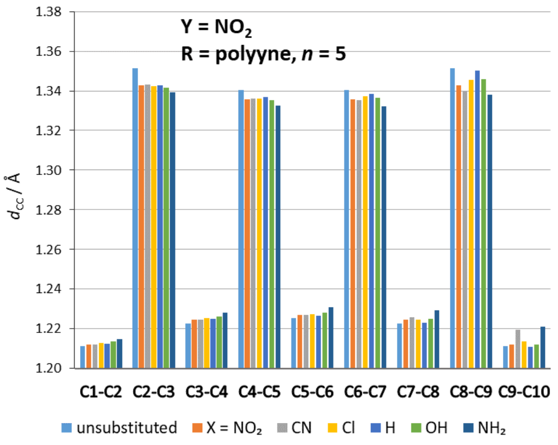

(e)

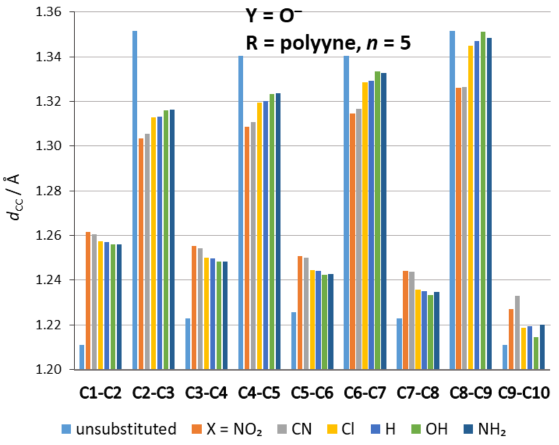

(f)

Figure S5. Lengths of CC bonds within the spacers R for each X derivative of six polyyne R-Y systems. First carbon atom (C1) is the one connected to the Y group, while the last (C8 or C10) to the X group.

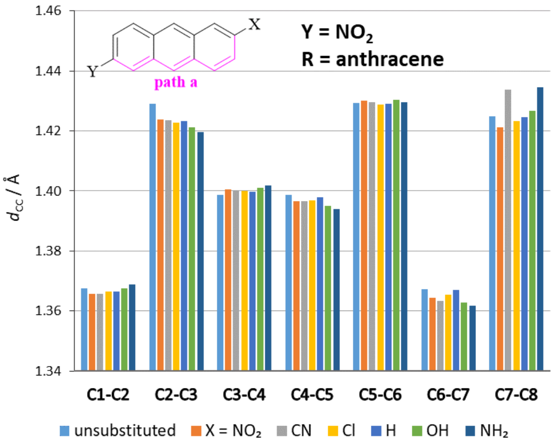
Y = NO_2
R = anthracene
path a
X
Y
d_{cc} / Å
1.34
1.36
1.38
1.40
1.42
1.44
1.46
C1-C2
C2-C3
C3-C4
C4-C5
C5-C6
C6-C7
C7-C8
unsubstituted
X = NO_2
CN
Cl
H
OH
NH_2

(a)

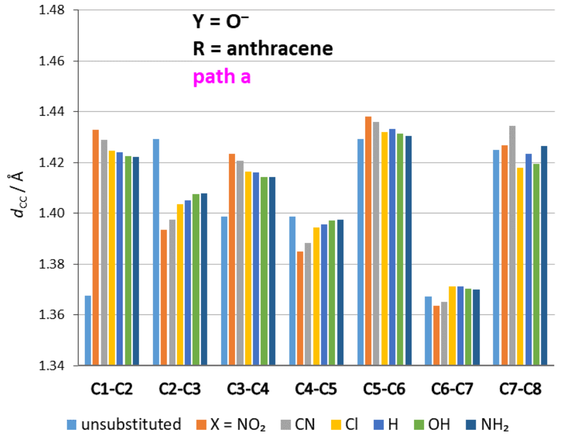
Y = O^-
R = anthracene
path a
d_{cc} / Å
1.34
1.36
1.38
1.40
1.42
1.44
1.46
1.48
C1-C2
C2-C3
C3-C4
C4-C5
C5-C6
C6-C7
C7-C8
unsubstituted
X = NO_2
CN
Cl
H
OH
NH_2

(b)

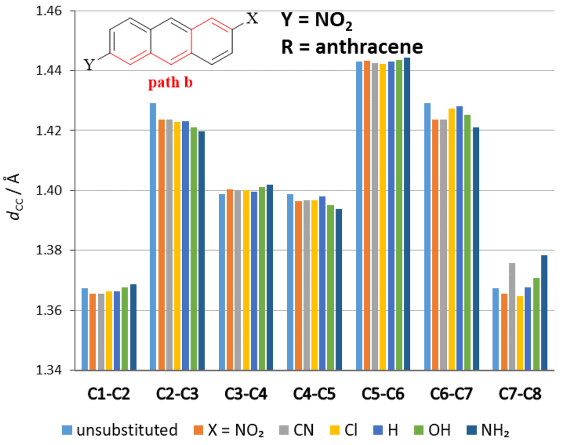
Y = NO_2
R = anthracene
path b
X
Y
d_{cc} / Å
1.34
1.36
1.38
1.40
1.42
1.44
1.46
C1-C2
C2-C3
C3-C4
C4-C5
C5-C6
C6-C7
C7-C8
unsubstituted
X = NO_2
CN
Cl
H
OH
NH_2

(c)

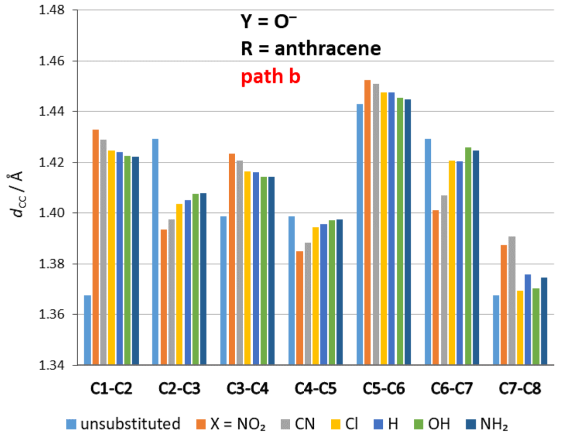
Y = O^-
R = anthracene
path b
d_{cc} / Å
1.34
1.36
1.38
1.40
1.42
1.44
1.46
1.48
C1-C2
C2-C3
C3-C4
C4-C5
C5-C6
C6-C7
C7-C8
unsubstituted
X = NO_2
CN
Cl
H
OH
NH_2

(d)

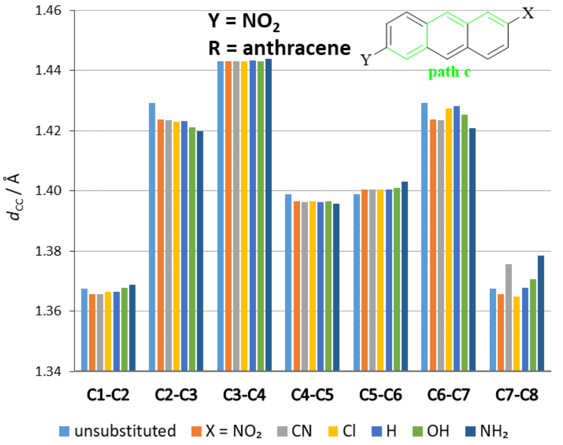
Y = NO_2
R = anthracene
path c
X
Y
d_{cc} / Å
1.34
1.36
1.38
1.40
1.42
1.44
1.46
C1-C2
C2-C3
C3-C4
C4-C5
C5-C6
C6-C7
C7-C8
unsubstituted
X = NO_2
CN
Cl
H
OH
NH_2

(e)

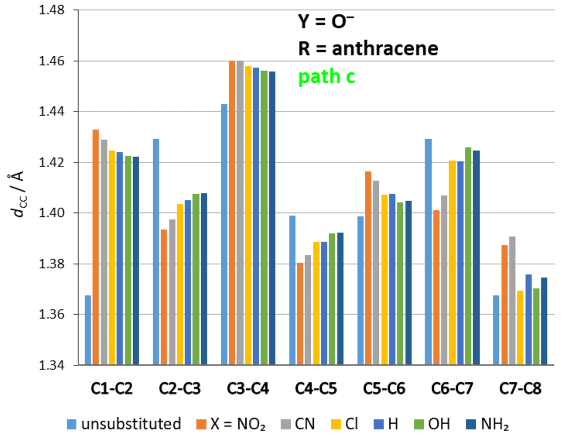
Y = O^-
R = anthracene
path c
d_{cc} / Å
1.34
1.36
1.38
1.40
1.42
1.44
1.46
1.48
C1-C2
C2-C3
C3-C4
C4-C5
C5-C6
C6-C7
C7-C8
unsubstituted
X = NO_2
CN
Cl
H
OH
NH_2

(f)

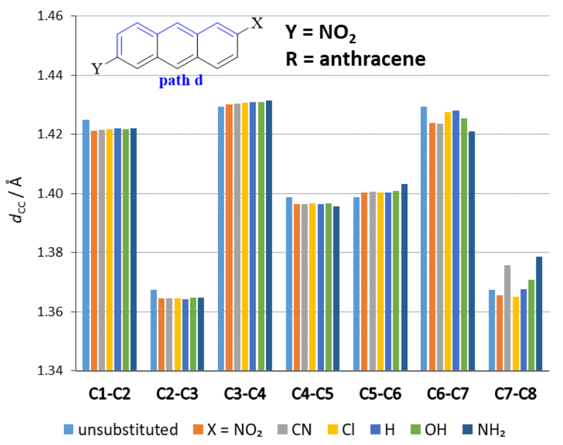

(g)

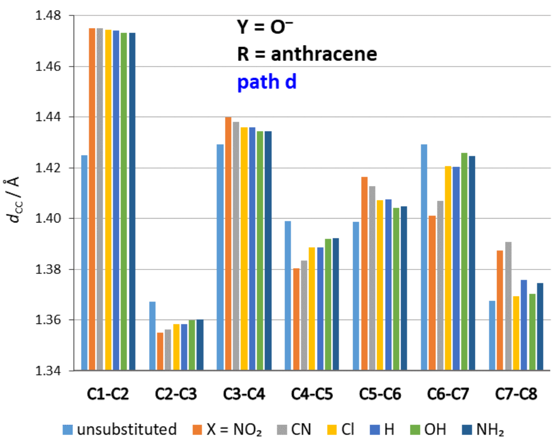

(h)

Figure S6. Lengths of CC bonds within the spacers R for each X derivative of anthracene-Y systems. First carbon atom (C1) is the one connected to the Y group, while the last (C8) to the X group. Bonds along paths (a, b, c, d) indicated in the figure, are presented.

Table S22. CC bond lengths along the delocalization paths between X and Y = NO_2 in acenes. Visualization of paths a, b, c, d and e is given in Table S20. All bond lengths in Å.

path a

	C1-C2	C2-C3	C3-C4	C4-C5	C5-C6	C6-C7	C7-C8	C8-C9	C9-C10	HOMA
naphthalene	1.374	1.420	1.420	1.374	1.415					0.837
X= NO_2	1.372	1.415	1.421	1.371	1.411					0.849
CN	1.372	1.415	1.421	1.370	1.424					0.811
Cl	1.373	1.414	1.420	1.372	1.414					0.852
H	1.373	1.415	1.420	1.373	1.415					0.846
OH	1.375	1.412	1.422	1.369	1.418					0.837
NH_2	1.376	1.410	1.422	1.368	1.426					0.815
anthracene	1.367	1.429	1.399	1.399	1.429	1.367	1.425			0.785
X= NO_2	1.366	1.424	1.400	1.396	1.430	1.364	1.421			0.800
CN	1.366	1.424	1.400	1.397	1.430	1.363	1.434			0.764
Cl	1.366	1.423	1.400	1.397	1.429	1.365	1.423			0.804
H	1.366	1.423	1.400	1.398	1.429	1.367	1.425			0.801
OH	1.368	1.421	1.401	1.395	1.430	1.363	1.427			0.792
NH_2	1.369	1.420	1.402	1.394	1.430	1.362	1.435			0.772
tetracene	1.364	1.433	1.391	1.409	1.409	1.391	1.433	1.364	1.430	0.776
X= NO_2	1.363	1.427	1.392	1.407	1.410	1.389	1.434	1.362	1.426	0.790
CN	1.363	1.427	1.392	1.407	1.410	1.389	1.434	1.361	1.438	0.761
Cl	1.364	1.426	1.392	1.407	1.409	1.389	1.433	1.363	1.428	0.797
H	1.364	1.426	1.392	1.408	1.409	1.391	1.433	1.364	1.429	0.795
OH	1.365	1.425	1.393	1.406	1.410	1.388	1.434	1.360	1.431	0.785
NH_2	1.366	1.424	1.394	1.405	1.411	1.387	1.434	1.359	1.439	0.766

path b

	C1-C2	C2-C3	C3-C4	C4-C5	C5-C6	C6-C7	C7-C8	C8-C9	C9-C10	HOMA
naphthalene	1.374	1.420	1.431	1.420	1.374					0.779
X= NO_2	1.372	1.415	1.432	1.415	1.372					0.798
CN	1.372	1.415	1.431	1.414	1.383					0.816
Cl	1.373	1.414	1.431	1.417	1.373					0.802
H	1.373	1.415	1.432	1.418	1.375					0.796
OH	1.375	1.412	1.432	1.415	1.378					0.819
NH_2	1.376	1.410	1.434	1.411	1.387					0.835
anthracene	1.367	1.429	1.399	1.399	1.443	1.429	1.367			0.724
X= NO_2	1.366	1.424	1.400	1.396	1.443	1.424	1.366			0.749
CN	1.366	1.424	1.400	1.397	1.442	1.424	1.376			0.765
Cl	1.366	1.423	1.400	1.397	1.442	1.427	1.365			0.745
H	1.366	1.423	1.400	1.398	1.443	1.428	1.368			0.742
OH	1.368	1.421	1.401	1.395	1.444	1.425	1.371			0.760
NH_2	1.369	1.420	1.402	1.394	1.444	1.421	1.379			0.781
tetracene	1.364	1.433	1.391	1.409	1.409	1.391	1.450	1.433	1.364	0.716
X= NO_2	1.363	1.427	1.392	1.407	1.410	1.389	1.450	1.427	1.363	0.743
CN	1.363	1.427	1.392	1.407	1.410	1.389	1.449	1.427	1.373	0.757
Cl	1.364	1.426	1.392	1.407	1.409	1.389	1.449	1.432	1.362	0.737
H	1.364	1.426	1.392	1.408	1.409	1.391	1.450	1.433	1.365	0.736
OH	1.365	1.425	1.393	1.406	1.410	1.388	1.450	1.430	1.367	0.748
NH_2	1.366	1.424	1.394	1.405	1.411	1.387	1.450	1.426	1.375	0.766

path c

	C1-C2	C2-C3	C3-C4	C4-C5	C5-C6	C6-C7	C7-C8	C8-C9	C9-C10	HOMA
naphthalene	1.415	1.374	1.420	1.420	1.374					0.837
X= NO_2	1.411	1.371	1.421	1.415	1.372					0.849
CN	1.412	1.371	1.422	1.414	1.383					0.860
Cl	1.412	1.371	1.422	1.417	1.373					0.841
H	1.412	1.371	1.422	1.418	1.375					0.841
OH	1.412	1.371	1.422	1.415	1.378					0.854
NH_2	1.413	1.371	1.424	1.411	1.387					0.859
anthracene	1.367	1.429	1.443	1.399	1.399	1.429	1.367			0.724
X= NO_2	1.366	1.424	1.443	1.396	1.400	1.424	1.366			0.749
CN	1.366	1.424	1.443	1.396	1.401	1.424	1.376			0.763
Cl	1.366	1.423	1.443	1.397	1.400	1.427	1.365			0.741
H	1.366	1.423	1.443	1.396	1.400	1.428	1.368			0.742
OH	1.368	1.421	1.443	1.397	1.401	1.425	1.371			0.761
NH_2	1.369	1.420	1.444	1.396	1.403	1.421	1.379			0.780

tetracene	1.364	1.433	1.391	1.409	1.450	1.409	1.391	1.433	1.364	0.715
X= NO_2	1.363	1.427	1.392	1.407	1.450	1.407	1.392	1.427	1.363	0.744
CN	1.363	1.427	1.392	1.407	1.450	1.407	1.392	1.427	1.373	0.755
Cl	1.364	1.426	1.392	1.407	1.450	1.408	1.392	1.432	1.362	0.734
H	1.364	1.426	1.392	1.408	1.450	1.407	1.392	1.433	1.365	0.735
OH	1.365	1.425	1.393	1.406	1.450	1.408	1.392	1.430	1.367	0.750
NH_2	1.366	1.424	1.394	1.405	1.451	1.407	1.394	1.426	1.375	0.769

path d

	C1-C2	C2-C3	C3-C4	C4-C5	C5-C6	C6-C7	C7-C8	C8-C9	C9-C10	HOMA
anthracene	1.425	1.367	1.429	1.399	1.399	1.429	1.367			0.785
X= NO_2	1.421	1.364	1.430	1.396	1.400	1.424	1.366			0.800
CN	1.421	1.364	1.430	1.396	1.401	1.424	1.376			0.812
Cl	1.422	1.364	1.431	1.397	1.400	1.427	1.365			0.785
H	1.422	1.364	1.431	1.396	1.400	1.428	1.368			0.786
OH	1.422	1.365	1.431	1.397	1.401	1.425	1.371			0.800
NH_2	1.422	1.365	1.431	1.396	1.403	1.421	1.379			0.814
tetracene	1.364	1.433	1.450	1.391	1.409	1.409	1.391	1.433	1.364	0.716
X= NO_2	1.363	1.427	1.450	1.389	1.410	1.407	1.392	1.427	1.363	0.743
CN	1.363	1.427	1.450	1.388	1.410	1.407	1.392	1.427	1.373	0.754
Cl	1.364	1.426	1.450	1.388	1.411	1.408	1.392	1.432	1.362	0.731
H	1.364	1.426	1.450	1.388	1.411	1.407	1.392	1.433	1.365	0.732
OH	1.365	1.425	1.450	1.388	1.411	1.408	1.392	1.430	1.367	0.746
NH_2	1.366	1.424	1.450	1.388	1.412	1.407	1.394	1.426	1.375	0.765

path e

	C1-C2	C2-C3	C3-C4	C4-C5	C5-C6	C6-C7	C7-C8	C8-C9	C9-C10	HOMA
tetracene	1.430	1.364	1.433	1.391	1.409	1.409	1.391	1.433	1.364	0.775
X= NO_2	1.426	1.362	1.434	1.389	1.410	1.407	1.392	1.427	1.363	0.790
CN	1.426	1.361	1.434	1.388	1.410	1.407	1.392	1.427	1.373	0.800
Cl	1.427	1.362	1.435	1.388	1.411	1.408	1.392	1.432	1.362	0.774
H	1.427	1.361	1.435	1.388	1.411	1.407	1.392	1.433	1.365	0.775
OH	1.427	1.362	1.435	1.388	1.411	1.408	1.392	1.430	1.367	0.786
NH_2	1.427	1.362	1.435	1.388	1.412	1.407	1.394	1.426	1.375	0.801

Table S23. CC bond lengths along the delocalization paths between X and Y = O^- in acenes. Visualization of paths a, b, c, d and e is given in Table S20.

path a

	C1-C2	C2-C3	C3-C4	C4-C5	C5-C6	C6-C7	C7-C8	C8-C9	C9-C10	HOMA
naphthalene	1.374	1.420	1.420	1.374	1.415					0.837
X= NO_2	1.439	1.388	1.439	1.366	1.422					0.651
CN	1.434	1.393	1.436	1.369	1.428					0.666
Cl	1.430	1.402	1.431	1.377	1.410					0.775

H	1.429	1.403	1.432	1.377	1.417					0.755
OH	1.427	1.407	1.429	1.378	1.410					0.787
NH_2	1.427	1.407	1.429	1.377	1.417					0.769
anthracene	1.367	1.429	1.399	1.399	1.429	1.367	1.425			0.785
X= NO_2	1.433	1.394	1.424	1.385	1.438	1.364	1.427			0.708
CN	1.429	1.398	1.421	1.388	1.436	1.365	1.434			0.712
Cl	1.425	1.404	1.416	1.394	1.432	1.371	1.418			0.796
H	1.424	1.405	1.416	1.396	1.433	1.371	1.423			0.780
OH	1.423	1.407	1.414	1.397	1.431	1.370	1.419			0.797
NH_2	1.422	1.408	1.414	1.397	1.430	1.370	1.426			0.782
tetracene	1.364	1.433	1.391	1.409	1.409	1.391	1.433	1.364	1.430	0.776
X= NO_2	1.431	1.395	1.418	1.391	1.423	1.383	1.439	1.362	1.429	0.741
CN	1.428	1.398	1.416	1.394	1.421	1.386	1.437	1.363	1.437	0.741
Cl	1.424	1.402	1.412	1.399	1.417	1.391	1.433	1.368	1.422	0.808
H	1.423	1.404	1.412	1.400	1.417	1.392	1.434	1.368	1.427	0.796
OH	1.422	1.405	1.411	1.402	1.415	1.393	1.433	1.367	1.424	0.809
NH_2	1.421	1.406	1.410	1.402	1.415	1.392	1.432	1.366	1.431	0.793

path b

	**C1-C2**	**C2-C3**	**C3-C4**	**C4-C5**	**C5-C6**	**C6-C7**	**C7-C8**	**C8-C9**	**C9-C10**	**HOMA**
naphthalene	1.374	1.420	1.431	1.420	1.374					0.779
X= NO_2	1.439	1.388	1.451	1.392	1.396					0.661
CN	1.434	1.393	1.449	1.397	1.399					0.688
Cl	1.430	1.402	1.444	1.411	1.377					0.706
H	1.429	1.403	1.444	1.410	1.384					0.714
OH	1.427	1.407	1.441	1.416	1.377					0.711
NH_2	1.427	1.407	1.440	1.415	1.381					0.722
anthracene	1.367	1.429	1.399	1.399	1.443	1.429	1.367			0.724
X= NO_2	1.433	1.394	1.424	1.385	1.452	1.401	1.387			0.719
CN	1.429	1.398	1.421	1.388	1.451	1.407	1.391			0.737
Cl	1.425	1.404	1.416	1.394	1.448	1.421	1.369			0.728
H	1.424	1.405	1.416	1.396	1.448	1.420	1.376			0.736
OH	1.423	1.407	1.414	1.397	1.445	1.426	1.370			0.728
NH_2	1.422	1.408	1.414	1.397	1.445	1.425	1.374			0.739
tetracene	1.364	1.433	1.391	1.409	1.409	1.391	1.450	1.433	1.364	0.716
X= NO_2	1.431	1.395	1.418	1.391	1.423	1.383	1.454	1.407	1.383	0.745
CN	1.428	1.398	1.416	1.394	1.421	1.386	1.453	1.412	1.386	0.758
Cl	1.424	1.402	1.412	1.399	1.417	1.391	1.451	1.426	1.366	0.744
H	1.423	1.404	1.412	1.400	1.417	1.392	1.451	1.426	1.371	0.751
OH	1.422	1.405	1.411	1.402	1.415	1.393	1.449	1.430	1.367	0.746
NH_2	1.421	1.406	1.410	1.402	1.415	1.392	1.449	1.429	1.372	0.755

path c

	C1-C2	C2-C3	C3-C4	C4-C5	C5-C6	C6-C7	C7-C8	C8-C9	C9-C10	HOMA
naphthalene	1.415	1.374	1.420	1.420	1.374					0.837
X= NO_2	1.469	1.360	1.434	1.392	1.396					0.511
CN	1.468	1.362	1.431	1.397	1.399					0.534
Cl	1.466	1.366	1.426	1.411	1.377					0.557
H	1.465	1.367	1.426	1.410	1.384					0.565
OH	1.464	1.369	1.423	1.416	1.377					0.574
NH_2	1.464	1.369	1.424	1.415	1.381					0.581
anthracene	1.367	1.429	1.443	1.399	1.399	1.429	1.367			0.724
X= NO_2	1.433	1.394	1.460	1.380	1.416	1.401	1.387			0.696
CN	1.429	1.398	1.460	1.383	1.413	1.407	1.391			0.709
Cl	1.425	1.404	1.458	1.388	1.407	1.421	1.369			0.696
H	1.424	1.405	1.457	1.389	1.407	1.420	1.376			0.707
OH	1.423	1.407	1.456	1.392	1.404	1.426	1.370			0.698
NH_2	1.422	1.408	1.456	1.392	1.405	1.425	1.374			0.705
tetracene	1.364	1.433	1.391	1.409	1.450	1.409	1.391	1.433	1.364	0.715
X= NO_2	1.431	1.395	1.418	1.391	1.459	1.390	1.409	1.407	1.383	0.750
CN	1.428	1.398	1.416	1.394	1.459	1.393	1.405	1.412	1.386	0.758
Cl	1.424	1.402	1.412	1.399	1.457	1.398	1.399	1.426	1.366	0.735
H	1.423	1.404	1.412	1.400	1.457	1.399	1.399	1.426	1.371	0.744
OH	1.422	1.405	1.411	1.402	1.456	1.402	1.397	1.430	1.367	0.735
NH_2	1.421	1.406	1.410	1.402	1.456	1.402	1.397	1.429	1.372	0.743

path d

	C1-C2	C2-C3	C3-C4	C4-C5	C5-C6	C6-C7	C7-C8	C8-C9	C9-C10	HOMA
anthracene	1.425	1.367	1.429	1.399	1.399	1.429	1.367			0.785
X= NO_2	1.475	1.355	1.440	1.380	1.416	1.401	1.387			0.544
CN	1.475	1.356	1.438	1.383	1.413	1.407	1.391			0.554
Cl	1.474	1.358	1.436	1.388	1.407	1.421	1.369			0.543
H	1.474	1.359	1.436	1.389	1.407	1.420	1.376			0.553
OH	1.473	1.360	1.434	1.392	1.404	1.426	1.370			0.551
NH_2	1.473	1.360	1.434	1.392	1.405	1.425	1.374			0.559
tetracene	1.364	1.433	1.450	1.391	1.409	1.409	1.391	1.433	1.364	0.716
X= NO_2	1.431	1.395	1.464	1.375	1.423	1.390	1.409	1.407	1.383	0.714
CN	1.428	1.398	1.465	1.377	1.421	1.393	1.405	1.412	1.386	0.723
Cl	1.424	1.402	1.464	1.380	1.418	1.398	1.399	1.426	1.366	0.700
H	1.423	1.404	1.464	1.380	1.418	1.399	1.399	1.426	1.371	0.709
OH	1.422	1.405	1.463	1.382	1.416	1.402	1.397	1.430	1.367	0.701
NH_2	1.421	1.406	1.463	1.382	1.416	1.402	1.397	1.429	1.372	0.710

path e

	C1-C2	C2-C3	C3-C4	C4-C5	C5-C6	C6-C7	C7-C8	C8-C9	C9-C10	HOMA

tetracene	1.430	1.364	1.433	1.391	1.409	1.409	1.391	1.433	1.364	0.775
X= NO_2	1.478	1.352	1.443	1.375	1.423	1.390	1.409	1.407	1.383	0.578
CN	1.479	1.353	1.443	1.377	1.421	1.393	1.405	1.412	1.386	0.582
Cl	1.479	1.354	1.441	1.380	1.418	1.398	1.399	1.426	1.366	0.562
H	1.478	1.354	1.441	1.380	1.418	1.399	1.399	1.426	1.371	0.571
OH	1.478	1.355	1.440	1.382	1.416	1.402	1.397	1.430	1.367	0.564
NH_2	1.478	1.355	1.440	1.382	1.416	1.402	1.397	1.429	1.372	0.574

Table S24. Changes in C-C bond lengths in percentages, relative to the unsubstituted R molecule. Cell shading proportional to the value – high positive in red, high negative in green.

Y = O^-, R = acene; **path a**

	**C1-C2**	**C2-C3**	**C3-C4**	**C4-C5**	**C5-C6**	**C6-C7**	**C7-C8**	**C8-C9**	**C9-C10**
naphthalene									
X= NO_2	4.69	-2.21	1.30	-0.60	0.47				
CN	4.37	-1.86	1.11	-0.42	0.92				
Cl	4.02	-1.27	0.77	0.18	-0.40				
H	3.95	-1.17	0.84	0.17	0.09				
OH	3.82	-0.93	0.64	0.26	-0.39				
NH_2	3.81	-0.93	0.63	0.20	0.11				
anthracene									
X= NO_2	4.79	-2.48	1.77	-0.99	0.62	-0.28	0.12		
CN	4.49	-2.21	1.57	-0.74	0.47	-0.17	0.67		
Cl	4.18	-1.79	1.26	-0.31	0.18	0.28	-0.50		
H	4.13	-1.67	1.23	-0.23	0.26	0.29	-0.11		
OH	4.03	-1.52	1.11	-0.11	0.14	0.22	-0.38		
NH_2	4.01	-1.48	1.10	-0.10	0.08	0.20	0.10		
tetracene									
X= NO_2	4.90	-2.68	2.00	-1.25	1.01	-0.54	0.39	-0.17	-0.06
CN	4.63	-2.46	1.83	-1.04	0.86	-0.34	0.25	-0.10	0.53
Cl	4.38	-2.14	1.57	-0.68	0.56	0.01	-0.01	0.27	-0.52
H	4.32	-2.04	1.52	-0.60	0.54	0.07	0.07	0.29	-0.17
OH	4.21	-1.94	1.44	-0.49	0.43	0.14	-0.04	0.18	-0.38
NH_2	4.18	-1.89	1.42	-0.46	0.43	0.13	-0.08	0.14	0.13

Y = O^-, R = acene; **path b**

	**C1-C2**	**C2-C3**	**C3-C4**	**C4-C5**	**C5-C6**	**C6-C7**	**C7-C8**	**C8-C9**	**C9-C10**
naphthalene									
X= NO_2	4.69	-2.21	1.35	-2.00	1.57				
CN	4.37	-1.86	1.21	-1.63	1.79				
Cl	4.02	-1.27	0.89	-0.65	0.16				
H	3.95	-1.17	0.89	-0.69	0.69				
OH	3.82	-0.93	0.70	-0.27	0.20				
NH_2	3.81	-0.93	0.64	-0.34	0.48				

anthracene									
X= NO_2	4.79	-2.48	1.77	-0.99	0.64	-1.96	1.47		
CN	4.49	-2.21	1.57	-0.74	0.55	-1.57	1.70		
Cl	4.18	-1.79	1.26	-0.31	0.31	-0.59	0.13		
H	4.13	-1.67	1.23	-0.23	0.32	-0.62	0.60		
OH	4.03	-1.52	1.11	-0.11	0.16	-0.24	0.22		
NH_2	4.01	-1.48	1.10	-0.10	0.13	-0.31	0.52		
tetracene									
X= NO_2	4.90	-2.68	2.00	-1.25	1.01	-0.54	0.32	-1.85	1.34
CN	4.63	-2.46	1.83	-1.04	0.86	-0.34	0.25	-1.48	1.60
Cl	4.38	-2.14	1.57	-0.68	0.56	0.01	0.05	-0.52	0.08
H	4.32	-2.04	1.52	-0.60	0.54	0.07	0.08	-0.52	0.51
OH	4.21	-1.94	1.44	-0.49	0.43	0.14	-0.03	-0.23	0.22
NH_2	4.18	-1.89	1.42	-0.46	0.43	0.13	-0.07	-0.30	0.53

Y = O^-, R = acene; **path c**

	**C1-C2**	**C2-C3**	**C3-C4**	**C4-C5**	**C5-C6**	**C6-C7**	**C7-C8**	**C8-C9**	**C9-C10**
naphthalene									
X= NO_2	3.77	-1.07	0.98	-2.00	1.57				
CN	3.70	-0.88	0.74	-1.63	1.79				
Cl	3.56	-0.58	0.41	-0.65	0.16				
H	3.55	-0.56	0.44	-0.69	0.69				
OH	3.44	-0.37	0.23	-0.27	0.20				
NH_2	3.41	-0.36	0.27	-0.34	0.48				
anthracene									
X= NO_2	4.79	-2.48	1.18	-1.32	1.26	-1.96	1.47		
CN	4.49	-2.21	1.16	-1.11	0.99	-1.57	1.70		
Cl	4.18	-1.79	1.04	-0.74	0.59	-0.59	0.13		
H	4.13	-1.67	0.99	-0.73	0.61	-0.62	0.60		
OH	4.03	-1.52	0.90	-0.49	0.39	-0.24	0.22		
NH_2	4.01	-1.48	0.89	-0.47	0.43	-0.31	0.52		
tetracene									
X= NO_2	4.90	-2.68	2.00	-1.25	0.62	-1.37	1.31	-1.85	1.34
CN	4.63	-2.46	1.83	-1.04	0.62	-1.15	1.05	-1.48	1.60
Cl	4.38	-2.14	1.57	-0.68	0.51	-0.74	0.62	-0.52	0.08
H	4.32	-2.04	1.52	-0.60	0.48	-0.72	0.63	-0.52	0.51
OH	4.21	-1.94	1.44	-0.49	0.41	-0.49	0.43	-0.23	0.22
NH_2	4.18	-1.89	1.42	-0.46	0.39	-0.46	0.46	-0.30	0.53

Y = O^-, R = acene; **path d**

	**C1-C2**	**C2-C3**	**C3-C4**	**C4-C5**	**C5-C6**	**C6-C7**	**C7-C8**	**C8-C9**	**C9-C10**
anthracene									

X= NO_2	3.51	-0.91	0.74	-1.32	1.26	-1.96	1.47		
CN	3.52	-0.82	0.63	-1.11	0.99	-1.57	1.70		
Cl	3.47	-0.67	0.46	-0.74	0.59	-0.59	0.13		
H	3.45	-0.65	0.47	-0.73	0.61	-0.62	0.60		
OH	3.39	-0.54	0.36	-0.49	0.39	-0.24	0.22		
NH_2	3.38	-0.53	0.36	-0.47	0.43	-0.31	0.52		
tetracene									
X= NO_2	4.90	-2.68	1.00	-1.13	1.02	-1.37	1.31	-1.85	1.34
CN	4.63	-2.46	1.02	-1.01	0.87	-1.15	1.05	-1.48	1.60
Cl	4.38	-2.14	0.99	-0.80	0.64	-0.74	0.62	-0.52	0.08
H	4.32	-2.04	0.97	-0.78	0.65	-0.72	0.63	-0.52	0.51
OH	4.21	-1.94	0.94	-0.66	0.52	-0.49	0.43	-0.23	0.22
NH_2	4.18	-1.89	0.91	-0.63	0.51	-0.46	0.46	-0.30	0.53

Y = O^-, R = acene; **path e**

	C1-C2	C2-C3	C3-C4	C4-C5	C5-C6	C6-C7	C7-C8	C8-C9	C9-C10
tetracene									
X= NO_2	3.41	-0.90	0.71	-1.13	1.02	-1.37	1.31	-1.85	1.34
CN	3.44	-0.85	0.65	-1.01	0.87	-1.15	1.05	-1.48	1.60
Cl	3.42	-0.75	0.54	-0.80	0.64	-0.74	0.62	-0.52	0.08
H	3.40	-0.73	0.53	-0.78	0.65	-0.72	0.63	-0.52	0.51
OH	3.39	-0.68	0.48	-0.66	0.52	-0.49	0.43	-0.23	0.22
NH_2	3.36	-0.66	0.47	-0.63	0.51	-0.46	0.46	-0.30	0.53

Y = NO_2, R = acene; **path a**

	C1-C2	C2-C3	C3-C4	C4-C5	C5-C6	C6-C7	C7-C8	C8-C9	C9-C10
naphthalene									
X= NO_2	-0.18	-0.33	0.09	-0.22	-0.30				
CN	-0.16	-0.35	0.06	-0.32	0.59				
Cl	-0.11	-0.41	0.01	-0.17	-0.11				
H	-0.12	-0.33	0.03	-0.06	-0.01				
OH	0.02	-0.59	0.16	-0.41	0.18				
NH_2	0.12	-0.73	0.11	-0.47	0.75				
anthracene									
X= NO_2	-0.13	-0.38	0.11	-0.16	0.06	-0.21	-0.26		
CN	-0.13	-0.39	0.10	-0.15	0.02	-0.30	0.61		
Cl	-0.08	-0.44	0.09	-0.14	-0.04	-0.14	-0.11		
H	-0.07	-0.42	0.06	-0.05	-0.02	-0.03	-0.02		
OH	0.02	-0.56	0.17	-0.26	0.07	-0.34	0.12		
NH_2	0.10	-0.66	0.22	-0.35	0.03	-0.40	0.67		
tetracene									
X= NO_2	-0.09	-0.42	0.11	-0.13	0.08	-0.15	0.07	-0.20	-0.24
CN	-0.08	-0.44	0.09	-0.11	0.04	-0.12	0.01	-0.28	0.61

Cl	-0.03	-0.48	0.10	-0.12	0.01	-0.09	-0.05	-0.12	-0.12
H	-0.02	-0.48	0.09	-0.08	-0.02	-0.01	-0.03	-0.01	-0.03
OH	0.02	-0.55	0.16	-0.20	0.07	-0.19	0.06	-0.32	0.10
NH_2	0.08	-0.63	0.22	-0.28	0.12	-0.27	0.02	-0.38	0.65

Y = NO_2, R = acene; **path b**

	**C1-C2**	**C2-C3**	**C3-C4**	**C4-C5**	**C5-C6**	**C6-C7**	**C7-C8**	**C8-C9**	**C9-C10**
naphthalene									
X= NO_2	-0.18	-0.33	0.04	-0.34	-0.18				
CN	-0.16	-0.35	-0.02	-0.39	0.61				
Cl	-0.11	-0.41	-0.02	-0.21	-0.13				
H	-0.12	-0.33	0.03	-0.15	0.05				
OH	0.02	-0.59	0.06	-0.35	0.30				
NH_2	0.12	-0.73	0.16	-0.66	0.90				
anthracene									
X= NO_2	-0.13	-0.38	0.11	-0.16	0.01	-0.38	-0.13		
CN	-0.13	-0.39	0.10	-0.15	-0.03	-0.40	0.60		
Cl	-0.08	-0.44	0.09	-0.14	-0.04	-0.13	-0.19		
H	-0.07	-0.42	0.06	-0.05	0.01	-0.08	0.02		
OH	0.02	-0.56	0.17	-0.26	0.04	-0.27	0.24		
NH_2	0.10	-0.66	0.22	-0.35	0.09	-0.58	0.81		
tetracene									
X= NO_2	-0.09	-0.42	0.11	-0.13	0.08	-0.15	-0.01	-0.43	-0.09
CN	-0.08	-0.44	0.09	-0.11	0.04	-0.12	-0.05	-0.41	0.62
Cl	-0.03	-0.48	0.10	-0.12	0.01	-0.09	-0.06	-0.11	-0.21
H	-0.02	-0.48	0.09	-0.08	-0.02	-0.01	-0.02	-0.05	0.01
OH	0.02	-0.55	0.16	-0.20	0.07	-0.19	0.01	-0.23	0.21
NH_2	0.08	-0.63	0.22	-0.28	0.12	-0.27	0.05	-0.53	0.76

Y = NO_2, R = acene; **path c**

	**C1-C2**	**C2-C3**	**C3-C4**	**C4-C5**	**C5-C6**	**C6-C7**	**C7-C8**	**C8-C9**	**C9-C10**
naphthalene									
X= NO_2	-0.30	-0.22	0.09	-0.34	-0.18				
CN	-0.26	-0.25	0.12	-0.39	0.61				
Cl	-0.24	-0.24	0.12	-0.21	-0.13				
H	-0.20	-0.24	0.11	-0.15	0.05				
OH	-0.26	-0.22	0.15	-0.35	0.30				
NH_2	-0.19	-0.26	0.29	-0.66	0.90				
anthracene									
X= NO_2	-0.13	-0.38	0.01	-0.17	0.11	-0.38	-0.13		
CN	-0.13	-0.39	0.01	-0.18	0.13	-0.40	0.60		
Cl	-0.08	-0.44	0.01	-0.17	0.11	-0.13	-0.19		
H	-0.07	-0.42	0.03	-0.19	0.12	-0.08	0.02		
OH	0.02	-0.56	0.01	-0.16	0.15	-0.27	0.24		
NH_2	0.10	-0.66	0.06	-0.23	0.31	-0.58	0.81		

tetracene									
X= NO_2	-0.09	-0.42	0.11	-0.13	0.01	-0.13	0.10	-0.43	-0.09
CN	-0.08	-0.44	0.09	-0.11	0.01	-0.13	0.10	-0.41	0.62
Cl	-0.03	-0.48	0.10	-0.12	0.01	-0.10	0.07	-0.11	-0.21
H	-0.02	-0.48	0.09	-0.08	0.02	-0.12	0.07	-0.05	0.01
OH	0.02	-0.55	0.16	-0.20	0.00	-0.10	0.11	-0.23	0.21
NH_2	0.08	-0.63	0.22	-0.28	0.04	-0.16	0.27	-0.53	0.76

Y = NO_2, R = acene; **path d**

	**C1-C2**	**C2-C3**	**C3-C4**	**C4-C5**	**C5-C6**	**C6-C7**	**C7-C8**	**C8-C9**	**C9-C10**
anthracene									
X= NO_2	-0.26	-0.21	0.06	-0.17	0.11	-0.38	-0.13		
CN	-0.24	-0.22	0.08	-0.18	0.13	-0.40	0.60		
Cl	-0.22	-0.22	0.10	-0.17	0.11	-0.13	-0.19		
H	-0.20	-0.23	0.11	-0.19	0.12	-0.08	0.02		
OH	-0.23	-0.20	0.11	-0.16	0.15	-0.27	0.24		
NH_2	-0.20	-0.20	0.15	-0.23	0.31	-0.58	0.81		
tetracene									
X= NO_2	-0.09	-0.42	-0.01	-0.15	0.08	-0.13	0.10	-0.43	-0.09
CN	-0.08	-0.44	0.00	-0.17	0.09	-0.13	0.10	-0.41	0.62
Cl	-0.03	-0.48	0.01	-0.17	0.11	-0.10	0.07	-0.11	-0.21
H	-0.02	-0.48	0.02	-0.19	0.13	-0.12	0.07	-0.05	0.01
OH	0.02	-0.55	0.01	-0.16	0.13	-0.10	0.11	-0.23	0.21
NH_2	0.08	-0.63	0.02	-0.18	0.18	-0.16	0.27	-0.53	0.76

Y = NO_2, R = acene; **path e**

	**C1-C2**	**C2-C3**	**C3-C4**	**C4-C5**	**C5-C6**	**C6-C7**	**C7-C8**	**C8-C9**	**C9-C10**
tetracene									
X= NO_2	-0.24	-0.20	0.07	-0.15	0.08	-0.13	0.10	-0.43	-0.09
CN	-0.22	-0.21	0.08	-0.17	0.09	-0.13	0.10	-0.41	0.62
Cl	-0.20	-0.21	0.09	-0.17	0.11	-0.10	0.07	-0.11	-0.21
H	-0.18	-0.21	0.10	-0.19	0.13	-0.12	0.07	-0.05	0.01
OH	-0.21	-0.19	0.10	-0.16	0.13	-0.10	0.11	-0.23	0.21
NH_2	-0.20	-0.19	0.12	-0.18	0.18	-0.16	0.27	-0.53	0.76

Y = NO_2, R = polyene

n=1			
X= NO_2	-0.462		
CN	0.211		
Cl	-0.053		
H	-0.431		
OH	0.819		
NH_2	1.601		
n=2			
X= NO_2	-0.15	-0.69	-0.15
CN	-0.06	-0.84	0.73
Cl	0.06	-0.88	0.07

H	-0.03	-0.54	0.10
OH	0.34	-1.46	0.75
NH_2	0.79	-2.09	1.61

n=3					
X= NO_2	-0.09	-0.59	0.27	-0.59	-0.09
CN	-0.01	-0.68	0.33	-0.65	0.73
Cl	0.12	-0.81	0.35	-0.48	-0.06
H	0.11	-0.76	0.27	-0.20	0.02
OH	0.32	-1.09	0.59	-0.94	0.55
NH_2	0.62	-1.47	0.98	-1.53	1.36

n=4							
X= NO_2	-0.01	-0.66	0.25	-0.32	0.25	-0.65	-0.01
CN	0.05	-0.73	0.29	-0.35	0.25	-0.65	0.76
Cl	0.18	-0.87	0.36	-0.39	0.20	-0.37	-0.12
H	0.19	-0.87	0.35	-0.33	0.13	-0.11	-0.01
OH	0.34	-1.06	0.53	-0.60	0.38	-0.76	0.45
NH_2	0.56	-1.33	0.80	-0.95	0.76	-1.32	1.24

n=5									
X= NO_2	0.06	-0.73	0.28	-0.30	0.17	-0.30	0.27	-0.73	0.06
CN	0.11	-0.79	0.32	-0.33	0.18	-0.29	0.25	-0.68	0.79
Cl	0.23	-0.92	0.40	-0.41	0.20	-0.25	0.15	-0.32	-0.14
H	0.25	-0.93	0.41	-0.40	0.19	-0.20	0.08	-0.06	-0.01
OH	0.36	-1.08	0.54	-0.57	0.35	-0.43	0.30	-0.66	0.40
NH_2	0.53	-1.28	0.74	-0.82	0.60	-0.77	0.67	-1.21	1.16

Y = O^-, R = polyene

n=1	
X= NO_2	6.10
CN	5.71
Cl	3.53
H	4.13
OH	2.88
NH_2	3.89

n=2			
X= NO_2	6.37	-4.81	3.62
CN	5.73	-4.17	3.66
Cl	4.84	-2.53	1.28
H	4.70	-2.49	2.10
OH	4.25	-1.52	0.86
NH_2	4.47	-2.00	1.55

n=3					
X= NO_2	6.44	-4.63	3.97	-3.86	3.07
CN	5.97	-4.14	3.43	-3.20	3.14

Cl	5.32	-3.28	2.56	-1.65	0.90				
H	5.16	-3.06	2.41	-1.53	1.50				
OH	4.88	-2.63	2.00	-0.75	0.56				
NH_2	4.89	-2.65	2.07	-0.92	0.95				
n=4									
X= NO_2	6.56	-4.66	3.93	-3.55	3.33	-3.47	2.73		
CN	6.19	-4.29	3.52	-3.05	2.81	-2.82	2.82		
Cl	5.66	-3.68	2.90	-2.19	1.93	-1.31	0.65		
H	5.51	-3.49	2.75	-1.97	1.77	-1.16	1.15		
OH	5.31	-3.22	2.49	-1.55	1.39	-0.52	0.39		
NH_2	5.27	-3.16	2.45	-1.51	1.41	-0.61	0.72		
n=5									
X= NO_2	6.68	-4.75	3.98	-3.54	3.26	-3.12	2.97	-3.23	2.50
CN	6.39	-4.43	3.66	-3.14	2.87	-2.61	2.47	-2.56	2.60
Cl	5.93	-3.97	3.16	-2.51	2.23	-1.74	1.58	-1.10	0.48
H	5.80	-3.81	3.02	-2.32	2.07	-1.52	1.41	-0.92	0.94
OH	5.63	-3.61	2.83	-2.05	1.82	-1.14	1.07	-0.41	0.30
NH_2	5.57	-3.53	2.76	-1.96	1.76	-1.07	1.07	-0.48	0.61

Y = NO_2, R = polyyne

n=1							
X= NO_2	-0.18						
CN	0.29						
Cl	0.04						
H	-0.18						
OH	-0.02						
NH_2	0.73						
n=2							
X= NO_2	0.04	-0.95	0.04				
CN	0.09	-1.04	0.58				
Cl	0.10	-0.84	0.24				
H	-0.02	-0.49	-0.00				
OH	0.21	-0.96	0.17				
NH_2	0.57	-1.56	0.95				
n=3							
X= NO_2	0.06	-0.73	0.18	-0.72	0.07		
CN	0.05	-0.70	0.26	-0.88	0.63		
Cl	0.11	-0.67	0.22	-0.57	0.22		
H	0.06	-0.58	0.10	-0.21	-0.02		
OH	0.21	-0.78	0.31	-0.60	0.12		
NH_2	0.44	-1.13	0.69	-1.20	0.89		
n=4							
X= NO_2	0.08	-0.67	0.16	-0.43	0.16	-0.67	0.07

CN	0.06	-0.64	0.16	-0.44	0.24	-0.86	0.66
Cl	0.13	-0.66	0.20	-0.35	0.16	-0.48	0.21
H	0.10	-0.62	0.14	-0.25	0.04	-0.12	-0.03
OH	0.21	-0.76	0.28	-0.41	0.23	-0.48	0.08
NH_2	0.36	-0.98	0.51	-0.75	0.60	-1.06	0.85

n=5

X= NO_2	0.09	-0.65	0.15	-0.36	0.11	-0.36	0.15	-0.65	0.09
CN	0.08	-0.63	0.15	-0.34	0.12	-0.39	0.25	-0.86	0.69
Cl	0.14	-0.67	0.20	-0.33	0.13	-0.26	0.14	-0.45	0.21
H	0.12	-0.64	0.17	-0.28	0.07	-0.16	0.02	-0.09	-0.02
OH	0.21	-0.74	0.28	-0.40	0.20	-0.30	0.19	-0.42	0.07
NH_2	0.31	-0.89	0.43	-0.61	0.42	-0.62	0.55	-0.98	0.82

Y = O^-, R = polyyne

n=1

X= NO_2	6.10
CN	5.71
Cl	3.53
H	4.13
OH	2.88
NH_2	3.89

n=2

X= NO_2	6.37	-4.81	3.62
CN	5.73	-4.17	3.66
Cl	4.84	-2.53	1.28
H	4.70	-2.49	2.10
OH	4.25	-1.52	0.86
NH_2	4.47	-2.00	1.55

n=3

X= NO_2	6.44	-4.63	3.97	-3.86	3.07
CN	5.97	-4.14	3.43	-3.20	3.14
Cl	5.32	-3.28	2.56	-1.65	0.90
H	5.16	-3.06	2.41	-1.53	1.50
OH	4.88	-2.63	2.00	-0.75	0.56
NH_2	4.89	-2.65	2.07	-0.92	0.95

n=4

X= NO_2	6.56	-4.66	3.93	-3.55	3.33	-3.7	2.73
CN	6.19	-4.29	3.52	-3.05	2.81	-2.82	2.82
Cl	5.66	-3.68	2.90	-2.19	1.93	-1.31	0.65
H	5.51	-3.49	2.75	-1.97	1.77	-1.16	1.15
OH	5.31	-3.22	2.49	-1.55	1.39	-0.52	0.39
NH_2	5.27	-3.16	2.45	-1.51	1.41	-0.61	0.72

n=5

X= NO_2	6.68	-4.75	3.98	-3.54	3.26	-3.12	2.97	-3.23	2.50
CN	6.39	-4.43	3.66	-3.14	2.87	-2.61	2.47	-2.56	2.59
Cl	5.93	-3.97	3.16	-2.51	2.23	-1.74	1.58	-1.10	0.48
H	5.80	-3.81	3.02	-2.32	2.07	-1.52	1.41	-0.92	0.94
OH	5.63	-3.61	2.83	-2.05	1.82	-1.14	1.07	-0.41	0.30
NH_2	5.57	-3.53	2.76	-1.96	1.76	-1.07	1.07	-0.48	0.61

Table S25. Changes in C-Y (C-N or C-O) and C-X bond lengths in percentages, relative to the monosubstituted R derivative. Cell shading proportional to the value – high positive in red, high negative in green. Substituent effect strength described by SESE in kcal/mol.

	Y = NO_2, R = polyene			Y = NO_2, R = polyyne			Y = NO_2, R = acene		
	SESE	d_{CN} %	d_{CX} %	SESE	d_{CN} %	d_{CX} %	SESE	d_{CN} %	d_{CX} %
n=1									
X= NO_2	-8.69	-0.10	-0.10	-4.14	0.10	0.10	-3.74	0.37	0.38
CN	-4.88	-0.24	-0.46	-3.78	0.00	-0.24	-2.91	0.22	0.01
Cl	-2.98	-0.73	-1.57	0.29	-0.32	-0.80	-0.97	-0.22	-0.63
OH	0.21	-1.87	-1.74	3.13	-0.82	-1.28	1.17	-0.77	-0.85
NH_2	6.17	-2.76	-2.88	8.11	-1.83	-2.53	3.21	-1.31	-1.47
	range	2.67	2.78	range	1.93	2.63	range	1.68	1.84
n=2									
X= NO_2	-4.40	0.49	0.49	-2.59	0.16	0.16	-2.72	0.36	0.36
CN	-3.17	0.28	-0.01	-2.66	0.21	-0.04	-2.15	0.23	0.04
Cl	-0.44	-0.09	-0.80	0.74	-0.14	-0.50	-0.74	0.00	-0.36
OH	2.35	-0.59	-1.12	2.78	-0.52	-0.87	0.70	-0.32	-0.52
NH_2	5.67	-1.23	-2.02	5.78	-1.11	-1.71	1.85	-0.61	-0.88
	range	1.72	2.51	range	1.32	1.87	range	0.97	1.24
n=3									
X= NO_2	-3.39	0.49	0.49	-2.20	0.17	0.17	-1.80	0.29	0.29
CN	-2.50	0.33	0.03	-2.22	0.22	0.00	-1.47	0.21	0.04
Cl	-0.36	-0.01	-0.56	0.26	-0.08	-0.35	-0.46	0.04	-0.25
OH	1.70	-0.34	-0.80	1.89	-0.34	-0.60	0.48	-0.14	-0.35
NH_2	3.97	-0.79	-1.46	4.20	-0.76	-1.26	1.25	-0.32	-0.59
	range	1.28	1.95	range	0.98	1.43	range	0.62	0.88
n=4									
X= NO_2	-2.95	0.44	0.44	-1.86	0.16	0.16	-1.33	0.25	0.25
CN	-2.05	0.31	0.04	-1.87	0.19	0.01	-1.13	0.18	0.04
Cl	-0.31	0.05	-0.43	-0.15	-0.06	-0.26	-0.33	0.05	-0.19
OH	1.17	-0.23	-0.62	1.21	-0.13	-0.44	0.37	-0.06	-0.24
NH_2	2.91	-0.58	-1.13	3.24	-0.55	-0.95	0.91	-0.19	-0.44
	range	1.02	1.56	range	0.74	1.11	range	0.44	0.69
n=5									
X= NO_2	-2.38	0.40	0.40	-0.87	0.15	0.15			
CN	-1.68	0.29	0.05	-1.52	0.17	0.02			
Cl	-0.27	0.06	-0.34	-1.31	-0.04	-0.20			
OH	1.00	-0.15	-0.50	1.48	-0.20	-0.35			

NH_2	2.33	-0.44	-0.91	2.29	-0.41	-0.72
	range	0.84	1.30	range	0.58	0.87

	Y = O^-, R = polyene			Y = O^-, R = polyyne			Y = O^-, R = acene		
	SESE	d_{CO} %	d_{CX} %	SESE	d_{CO} %	d_{CX} %	SESE	d_{CO} %	d_{CX} %
n=1									
X= NO_2	37.85	-1.72	-6.26	39.74	-2.40	-2.08	25.67	-1.26	-4.62
CN	29.53	-1.40	-1.94	34.79	-1.25	-1.85	19.05	-0.89	-1.30
Cl	7.14	0.02	3.65	4.77	-0.96	8.04	5.84	-0.21	1.84
OH	-8.96	0.78	5.38	-12.54	0.07	8.69	-3.62	0.30	3.00
NH_2	-9.83	0.20	4.86	-16.84	0.71	6.13	-6.08	0.05	4.07
	range	2.50	11.63	range	3.11	10.78	range	1.56	8.69
n=2									
X= NO_2	31.43	-1.42	-4.64	29.32	-1.33	-3.41	18.83	-0.88	-3.53
CN	22.01	-0.98	-1.26	25.12	-1.14	-1.07	13.77	-0.58	-0.87
Cl	5.97	-0.25	2.93	0.56	-0.28	3.48	4.56	-0.16	1.41
OH	-5.53	0.34	4.20	-9.48	0.19	5.59	-1.98	0.14	2.07
NH_2	-9.10	0.19	4.84	-12.34	0.49	4.99	-4.73	0.20	2.70
	range	1.76	9.48	range	1.81	9.00	range	1.08	6.22
n=3									
X= NO_2	25.67	-1.07	-3.98	23.24	-0.97	-2.93	14.84	-0.65	-2.90
CN	17.62	-0.71	-1.02	19.14	-0.80	-0.84	10.63	-0.43	-0.64
Cl	4.38	-0.18	2.24	-0.51	-0.06	1.94	3.52	-0.12	1.14
OH	-4.63	0.24	3.24	-7.52	0.28	3.40	-1.39	0.11	1.59
NH_2	-8.56	0.25	4.03	-10.36	0.35	3.97	-3.84	0.15	2.12
	range	1.33	8.01	range	1.32	6.89	range	0.81	5.02
n=4									
X= NO_2	21.74	-0.87	-3.51	18.99	-0.76	-2.58	12.12	-0.53	-2.47
CN	14.72	-0.57	-0.86	15.44	-0.61	-0.70	8.56	-0.33	-0.51
Cl	3.58	-0.13	1.88	-0.34	-0.04	1.52	2.75	-0.10	0.93
OH	-3.92	0.18	2.66	-6.50	0.22	2.59	-1.12	0.09	1.27
NH_2	-7.76	0.24	3.47	-9.10	0.26	3.29	-3.32	0.14	1.75
	range	1.10	6.98	range	1.03	5.87	range	0.67	4.21
n=5									
X= NO_2	18.92	-0.73	-3.15	16.12	-0.62	-2.28			
CN	12.81	-0.50	-0.73	12.84	-0.49	-0.59			
Cl	2.98	-0.12	1.63	-1.05	-0.02	1.27			
OH	-4.71	0.14	2.26	-5.75	0.18	2.13			
NH_2	-6.95	0.20	3.06	-7.69	0.22	2.77			
	range	0.93	6.21	range	0.84	5.04			

Table S26. Conformational analysis of the X = OH polyene and acene derivatives.

	Y = NO_2			Y = O^-		
	OH1<br>*E*/hartree	OH2<br>*E*/hartree	Δ*E*/<br>kcal·mol^{-1}	OH1<br>*E*/hartree	OH2<br>*E*/hartree	Δ*E* /<br>kcal·mol^{-1}
benzene	-512.124			-382.236		
naphthalene	-665.801	-665.802	-0.548	-535.925	-535.926	-0.638
anthracene	-819.473	-819.474	-0.808	-689.605	-689.606	-0.850
tetracene	-973.142	-973.143	-0.897	-843.280	-843.281	-0.970
ethylene	-358.429	-358.430	-0.306	-228.519	-228.524	-3.421
butadiene	-435.858	-435.859	-0.644	-305.970	-305.973	-2.301
hexatriene	-513.285	-513.286	-0.721	-383.411	-383.415	-2.080
octatetraene	-590.713	-590.714	-0.757	-460.849	-460.852	-1.925
decapentaene	-668.141	-668.142	-0.747	-538.285	-538.288	-1.808

OH1 conformation

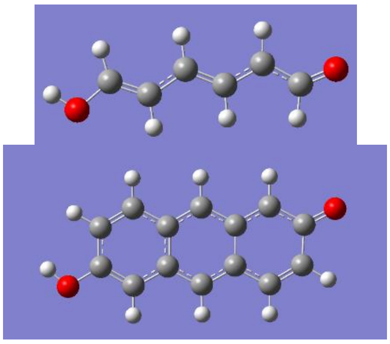

OH2 conformation

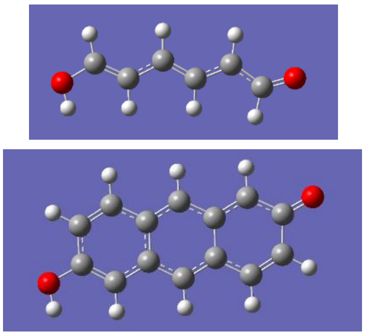

Computational details: B3LYP/6-311++G(d,p) method, UltraFine grid, tight convergence criteria.

Cartesian coordinates, electronic energies (E), zero point energies (ZPE) and Gibbs energies (G).

**Disubstituted systems**	
Y = NO2, R = polyene	S50-S57
Y = NO2, R = polyyne	S57-S61
Y = NO2, R = acene	S61-S67
Y = O^-, R = polyene	S67-S73
Y = O^-, R = polyyne	S73-S76
Y = O^-, R = acene	S77-S81
**Monosubstituted systems**	
R = polyene	S82-S89
R = polyyne	S89-S93
R = acene	S93-S100

Y = NO2, R = polyene, *n* = 1

X = NO2 E = -487.727617 a.u. ZPE = 0.057177 a.u. G = -487.703326 a.u.

C	0.05292	0.65925	0.00000
H	-0.76956	1.35862	0.00000
C	-0.05292	-0.65925	0.00000
H	0.76956	-1.35862	0.00000
O	2.36909	0.56559	0.00000
O	1.38327	2.50935	0.00000
O	-2.36909	-0.56559	0.00000
O	-1.38327	-2.50935	0.00000
N	1.38327	1.28710	0.00000
N	-1.38327	-1.28710	0.00000

X = CN E = -375.437778 a.u. ZPE = 0.053482 a.u. G = -375.415299 a.u.

C	0.00000	0.48291	0.00000
H	0.29653	1.52117	0.00000
C	0.80179	-0.58034	0.00000
H	0.37428	-1.57806	0.00000
O	-1.90886	-0.83450	0.00000
O	-2.11358	1.33493	0.00000
N	-1.45767	0.30158	0.00000
C	2.21774	-0.45821	0.00000
N	3.37074	-0.38911	0.00000

X = Cl E = -742.798286 a.u. ZPE = 0.045674 a.u. G = -742.783079 a.u.

C	0.00000	0.53541	0.00000
H	0.50916	1.48654	0.00000
C	0.56129	-0.66834	0.00000
H	-0.02144	-1.57933	0.00000
O	-2.13507	-0.36807	0.00000
O	-1.89626	1.79621	0.00000
N	-1.45707	0.65228	0.00000
Cl	2.27028	-0.88828	0.00000

X = H E = -283.179125 a.u. ZPE = 0.054604 a.u. G = -283.152373 a.u.

C	-0.60038	-0.78187	0.00000
H	-1.67938	-0.73888	0.00000
C	0.15769	-1.86632	0.00000
H	1.23765	-1.79068	0.00000
O	1.21824	0.66864	0.00000
O	-0.79342	1.49726	0.00000
N	0.00000	0.56265	0.00000
H	-0.30067	-2.84706	0.00000

X = OH E = -357.134348 a.u. ZPE = 0.035842 a.u. G = -357.128154 a.u.

C	1.44690	-0.01477	0.00007
C	0.24781	-0.02025	0.00046
O	-1.72017	-1.07875	-0.00008
O	-1.68143	1.10655	-0.00020
N	-1.14146	0.00327	0.00010
O	2.73572	-0.10126	-0.00035
H	3.14900	0.77493	0.00108

X = NH2 E = -338.568119 a.u. ZPE = 0.071946 a.u. G = -338.526102 a.u.

C	0.20380	-0.55405	0.00006
H	0.22279	-1.63265	0.00006
C	1.27452	0.26841	-0.00006
H	1.09460	1.33749	-0.00018
O	-1.26443	1.22532	0.00005
O	-2.04877	-0.80682	-0.00006

N	-1.11658	-0.00035	0.00002
N	2.57018	-0.11649	-0.00006
H	3.30688	0.56642	0.00015
H	2.83625	-1.08760	0.00033

Y = NO2, R = polyene, *n* = 2

X = NO2 E = -565.164607 a.u. ZPE = 0.090960 a.u. G = -565.109438 a.u.

C	-0.36223	1.79000	0.00000
H	-1.43748	1.89650	0.00000
C	0.32679	0.64502	0.00000
H	1.41086	0.69444	0.00000
C	-0.32679	-0.64502	0.00000
H	-1.41086	-0.69444	0.00000
C	0.36223	-1.79000	0.00000
H	1.43748	-1.89650	0.00000
O	-1.55105	-3.09809	0.00000
O	0.39958	-4.06826	0.00000
N	-0.32679	-3.08069	0.00000
O	1.55105	3.09809	0.00000
O	-0.39958	4.06826	0.00000
N	0.32679	3.08069	0.00000

X = CN E = -452.870576 a.u. ZPE = 0.087044 a.u. G = -452.818112 a.u.

C	2.09827	-0.97250	0.00000
H	2.67183	-0.05054	0.00000
C	0.75034	-0.94937	0.00000
H	0.19998	-1.88504	0.00000
C	0.00000	0.28433	0.00000
H	0.53718	1.22774	0.00000
C	-1.33717	0.31783	0.00000
H	-2.00404	-0.53215	0.00000
O	-1.41523	2.63361	0.00000
O	-3.27985	1.50726	0.00000
N	-2.05564	1.58892	0.00000
C	2.85013	-2.17794	0.00000
N	3.48224	-3.14623	0.00000

X = Cl E = -820.229348 a.u. ZPE = 0.079123 a.u. G = -820.184241 a.u.

C	-1.42106	-1.57731	0.00000
H	-2.32987	-0.98904	0.00000
C	-0.19309	-1.04261	0.00000
H	0.67833	-1.68906	0.00000
C	0.00000	0.38776	0.00000
H	-0.87197	1.03460	0.00000
C	1.20705	0.96765	0.00000
H	2.16308	0.46539	0.00000
O	0.32754	3.11055	0.00000
O	2.48957	2.84909	0.00000
N	1.33959	2.41621	0.00000
Cl	-1.71241	-3.28385	0.00000

X = H E = -360.606429 a.u. ZPE = 0.088053 a.u. G = -360.549856 a.u.

C	2.76627	-1.74581	0.00000
H	2.23476	-2.69177	0.00000
C	2.11292	-0.57628	0.00000
H	2.67289	0.35496	0.00000
C	0.66742	-0.48572	0.00000
H	0.09026	-1.40557	0.00000
C	0.00000	0.67378	0.00000
H	0.43098	1.66409	0.00000
O	-2.07778	-0.35326	0.00000
O	-1.96817	1.82113	0.00000
N	-1.45564	0.70415	0.00000
H	3.84851	-1.78960	0.00000

X = OH E = -435.859140 a.u. ZPE = 0.093372 a.u. G = -435.798784 a.u.

C	1.06903	2.42622	0.00000
H	0.11129	2.93471	0.00000
C	1.16808	1.08148	0.00000
H	2.14859	0.61089	0.00000
C	0.00000	0.24816	0.00000
H	-0.97551	0.72602	0.00000
C	0.02330	-1.09444	0.00000
H	0.90076	-1.72328	0.00000
O	-2.28449	-1.26696	0.00000
O	-1.08175	-3.08279	0.00000
N	-1.20622	-1.85812	0.00000
O	2.08624	3.30525	0.00000
H	2.93593	2.84594	0.00000

X = NH2 E = -415.994973 a.u. ZPE = 0.105032 a.u. G = -415.924022 a.u.

C	2.61205	0.36526	0.00016
H	2.48570	1.44375	-0.00032
C	1.51422	-0.43728	0.00056
H	1.63642	-1.51716	0.00086
C	0.19750	0.10948	-0.00043
H	0.08921	1.19079	-0.00126
C	-0.94280	-0.61117	0.00013
H	-1.01855	-1.68780	0.00048
O	-2.29005	1.26750	0.00027
O	-3.21360	-0.70577	0.00024
N	-2.22708	0.03575	0.00023
N	3.90536	-0.04611	-0.01108
H	4.65935	0.61520	0.04404
H	4.14326	-1.02386	0.02555

Y = NO2, R = polyene, *n* = 3

X = NO2 E = -642.596142 a.u. ZPE = 0.124243 a.u. G = -642.511272 a.u.

C	1.20354	2.80321	0.00000
H	0.33674	3.44781	0.00000
C	1.20354	1.46347	0.00000
H	2.16258	0.95458	0.00000
C	-0.00275	0.67666	0.00000
H	-0.95291	1.20460	0.00000
C	0.00275	-0.67666	0.00000
H	0.95291	-1.20460	0.00000
O	2.34106	4.77589	0.00000
O	3.51584	2.94127	0.00000
N	2.45331	3.55321	0.00000
C	-1.20354	-1.46347	0.00000
H	-2.16258	-0.95458	0.00000
C	-1.20354	-2.80321	0.00000
H	-0.33674	-3.44781	0.00000
O	-3.51584	-2.94127	0.00000
O	-2.34106	-4.77589	0.00000
N	-2.45331	-3.55321	0.00000

X = CN E = -530.301201 a.u. ZPE = 0.120525 a.u. G = -530.218625 a.u.

C	3.57487	-0.52468	0.00000
H	3.51864	-1.60894	0.00000
C	2.45346	0.22831	0.00000
H	2.54413	1.31077	0.00000
C	1.12732	-0.33113	0.00000
H	1.04205	-1.41537	0.00000
C	0.00000	0.41887	0.00000
H	0.08173	1.50289	0.00000
C	-1.31957	-0.15501	0.00000
H	-1.42045	-1.23607	0.00000
C	-2.44194	0.57839	0.00000
H	-2.50614	1.65656	0.00000
O	-3.82342	-1.28044	0.00000
O	-4.71498	0.70718	0.00000
N	-3.75184	-0.05559	0.00000
C	4.87987	0.03372	0.00000
N	5.95230	0.46780	0.00000

X = Cl E = -897.658059 a.u. ZPE = 0.112476 a.u. G = -897.583123 a.u.

C	2.84291	-1.83878	0.00000
H	2.37105	-2.81292	0.00000
C	2.16473	-0.68286	0.00000
H	2.70860	0.25698	0.00000
C	0.72369	-0.63806	0.00000
H	0.19663	-1.58972	0.00000
C	0.00000	0.50670	0.00000
H	0.51534	1.46386	0.00000
C	-1.43699	0.51580	0.00000
H	-1.96364	-0.43396	0.00000
C	-2.17650	1.63626	0.00000
H	-1.80494	2.65023	0.00000
O	-4.18307	0.48168	0.00000
O	-4.20921	2.65966	0.00000
N	-3.62567	1.57661	0.00000
Cl	4.57575	-1.92329	0.00000

X = H E = -438.034866 a.u. ZPE = 0.121418 a.u. G = -437.948395 a.u.

C	1.71519	-4.27259	0.00000
H	2.74275	-3.92341	0.00000
C	0.68404	-3.41484	0.00000
H	-0.33283	-3.80014	0.00000
C	0.83783	-1.97725	0.00000
H	1.85563	-1.59184	0.00000
C	-0.19217	-1.09946	0.00000
H	-1.21257	-1.47481	0.00000
C	0.00000	0.32535	0.00000
H	1.01447	0.71277	0.00000
C	-1.00587	1.21429	0.00000
H	-2.06172	0.98807	0.00000
O	0.41541	3.04217	0.00000
O	-1.73790	3.36935	0.00000
N	-0.74589	2.64127	0.00000
H	1.56131	-5.34465	0.00000

X = OH E = -513.286357 a.u. ZPE = 0.126521 a.u. G = -513.196454 a.u.

C	-3.92718	0.44639	0.00001
H	-3.86792	1.52908	-0.00002
C	-2.82879	-0.33568	0.00001
H	-2.94097	-1.41828	0.00003
C	-1.50100	0.20878	-0.00002
H	-1.41699	1.29409	-0.00004
C	-0.35719	-0.52196	-0.00002
H	-0.41613	-1.60747	0.00000
C	0.94223	0.08202	-0.00005
H	1.00956	1.16616	-0.00007
C	2.09764	-0.60686	-0.00005
H	2.20278	-1.68137	-0.00002
O	3.40247	1.30371	0.00005
O	4.37194	-0.64743	0.00008
N	3.37295	0.07371	-0.00007
O	-5.20954	0.03111	0.00003
H	-5.25427	-0.93347	0.00005

X = NH2 E = -493.421351 a.u. ZPE = 0.138559 a.u. G = -493.319651 a.u.

C	3.91670	0.43170	0.00149
H	3.80296	1.51174	0.00123
C	2.81205	-0.36028	0.00192
H	2.92953	-1.44145	0.00135
C	1.49061	0.17724	-0.00065
H	1.40488	1.26326	-0.00231
C	0.33682	-0.54802	-0.00024
H	0.39022	-1.63385	0.00005
C	-0.95263	0.06439	-0.00031
H	-1.00895	1.14935	-0.00053
C	-2.12177	-0.60897	0.00002
H	-2.23885	-1.68209	0.00012
O	-3.39944	1.31787	-0.00034
O	-4.39506	-0.62064	0.00149
N	-3.38223	0.08512	0.00036
N	5.21025	0.00588	-0.03239
H	5.96098	0.65400	0.13073
H	5.42839	-0.97223	0.07106

Y = NO2, R = polyene, *n* = 4

X = NO2 E = -720.026456 a.u. ZPE = 0.157787 a.u. G = -719.911221 a.u.

C	-0.46738	4.25635	0.00000
H	-1.54198	4.36454	0.00000
C	0.21936	3.10350	0.00000
H	1.30365	3.16085	0.00000
C	-0.40565	1.80956	0.00000
H	-1.49205	1.76999	0.00000
C	0.30079	0.65154	0.00000
H	1.38755	0.69941	0.00000
O	1.44657	5.55928	0.00000
O	-0.50267	6.53232	0.00000
N	0.21936	5.53771	0.00000
C	-0.30079	-0.65154	0.00000
H	-1.38755	-0.69941	0.00000
C	0.40565	-1.80956	0.00000
H	1.49205	-1.76999	0.00000
C	-0.21936	-3.10350	0.00000
H	-1.30365	-3.16085	0.00000
C	0.46738	-4.25635	0.00000
H	1.54198	-4.36454	0.00000
O	-1.44657	-5.55928	0.00000
O	0.50267	-6.53232	0.00000
N	-0.21936	-5.53771	0.00000

X = CNE = -607.730923 a.u. ZPE = 0.153795 a.u. G = -607.618619 a.u.

C	-3.68512	0.12073	0.00000
H	-3.98250	-0.91747	0.00000
C	-2.42763	0.59139	0.00000
H	-2.29300	1.66886	0.00000
C	-1.26400	-0.25002	0.00000
H	-1.41457	-1.32668	0.00000
C	0.00000	0.24387	0.00000
H	0.13970	1.32285	0.00000
O	-4.62642	2.23564	0.00000
O	-5.93075	0.49068	0.00000
N	-4.82239	1.02333	0.00000
C	1.18129	-0.57046	0.00000
H	1.04311	-1.64959	0.00000
C	2.44234	-0.07036	0.00000
H	2.58483	1.00802	0.00000
C	3.62084	-0.89259	0.00000
H	3.48152	-1.97010	0.00000
C	4.87869	-0.39633	0.00000
H	5.05514	0.67487	0.00000
C	6.03351	-1.22055	0.00000
N	6.98863	-1.87410	0.00000

X = Cl E = -975.086599 a.u. ZPE = 0.145718 a.u. G = -974.981970 a.u

C	-2.69487	2.85384	0.00000
H	-3.64063	2.33278	0.00000
C	-1.47395	2.29109	0.00000
H	-0.61597	2.95689	0.00000
C	-1.24512	0.87555	0.00000
H	-2.11150	0.21880	0.00000
C	0.00000	0.33351	0.00000
H	0.85740	1.00402	0.00000
O	-1.85735	5.01126	0.00000
O	-4.01472	4.70724	0.00000
N	-2.85526	4.29311	0.00000
C	0.27373	-1.07440	0.00000
H	-0.57896	-1.74995	0.00000
C	1.52585	-1.59458	0.00000
H	2.37564	-0.91517	0.00000
C	1.81148	-3.00706	0.00000
H	0.97401	-3.69837	0.00000
C	3.06202	-3.48964	0.00000
H	3.94804	-2.86827	0.00000
Cl	3.42356	-5.18884	0.00000

.

X = H E = -515.463245 a.u. ZPE = 0.154684 a.u. G = -515.346997 a.u

C	-2.31090	1.40380	0.00000
H	-2.08186	2.45898	0.00000
C	-1.42169	0.39526	0.00000
H	-1.81262	-0.61793	0.00000

C	0.00000	0.58191	0.00000
H	0.38067	1.60030	0.00000
C	0.87643	-0.45529	0.00000
H	0.48082	-1.46930	0.00000
O	-4.13945	-0.01456	0.00000
O	-4.46361	2.13977	0.00000
N	-3.73537	1.14672	0.00000
C	2.30452	-0.31438	0.00000
H	2.70648	0.69672	0.00000
C	3.16220	-1.36329	0.00000
H	2.75674	-2.37351	0.00000
C	4.60174	-1.23869	0.00000
H	5.00533	-0.22868	0.00000
C	5.44414	-2.28331	0.00000
H	5.07935	-3.30547	0.00000
H	6.51840	-2.14584	0.00000

X = OH E = -590.713991 a.u. ZPE = 0.159813 a.u. G = -590.594255 a.u.

C	1.39571	5.04317	0.00000
H	0.36608	5.38279	0.00000
C	1.72489	3.73583	0.00000
H	2.77518	3.44935	0.00000
C	0.74005	2.69147	0.00000
H	-0.30311	3.00325	0.00000
C	1.01284	1.36080	0.00000
H	2.04990	1.03211	0.00000
C	-0.00000	0.34969	0.00000
H	-1.03435	0.68983	0.00000
C	0.23520	-0.99015	-0.00000
H	1.26107	-1.35014	-0.00000
C	-0.81807	-1.95898	-0.00000
H	-1.84581	-1.60762	-0.00000
C	-0.62121	-3.29099	-0.00000
H	0.33293	-3.79621	-0.00000
O	-2.88384	-3.77133	-0.00000
O	-1.44734	-5.40994	-0.00000
N	-1.73428	-4.21114	-0.00000
O	2.25079	6.08789	0.00000
H	3.16473	5.77676	0.00000

X = NH2 E = -570.848446 a.u. ZPE = 0.171919 a.u. G = -570.716722 a.u.

C	-3.31752	-0.58644	0.00056
H	-3.46286	-1.65615	0.00169
C	-2.13031	0.05507	-0.00062
H	-2.15468	1.14106	-0.00182
C	-0.86026	-0.59611	-0.00026
H	-0.84068	-1.68318	0.00078
C	0.31793	0.09112	-0.00100
H	0.27214	1.17942	-0.00221
O	-4.54378	1.37327	0.00021
O	-5.59039	-0.53865	0.00103
N	-4.55943	0.14058	0.00057
C	1.61699	-0.49722	-0.00016
H	1.67913	-1.58333	0.00094
C	2.76898	0.23226	-0.00084
H	2.68164	1.31838	-0.00336
C	4.09221	-0.30283	0.00271
H	4.20899	-1.38432	0.00232
C	5.19868	0.48558	0.00217
H	5.08920	1.56607	0.00150
N	6.49336	0.05425	-0.03962
H	7.24046	0.69629	0.16205
H	6.70237	-0.92356	0.08621

Y = NO2, R = polyene, *n* = 5

X = NO2 E = -797.456169 a.u. ZPE = 0.191138 a.u. G = -797.311675 a.u.

C	0.81387	2.96832	0.00000
H	1.88875	2.80544	0.00000
C	-0.02046	1.89649	0.00000
H	-1.09438	2.07133	0.00000
C	0.42095	0.53358	0.00000
H	1.49441	0.35643	0.00000
C	-0.42095	-0.53359	0.00000
H	-1.49441	-0.35643	0.00000
C	0.02046	-1.89650	0.00000
H	1.09438	-2.07134	0.00000
C	-0.81387	-2.96832	0.00000
H	-1.88875	-2.80544	0.00000
C	-0.33977	-4.32271	0.00000
H	0.73132	-4.50124	0.00000
C	-1.14956	-5.39441	0.00000
H	-2.22945	-5.38326	0.00000
O	0.60902	-6.89932	0.00000
O	-1.43564	-7.65194	0.00000
N	-0.60903	-6.74119	0.00000
C	0.33977	4.32271	0.00000
H	-0.73132	4.50124	0.00000
C	1.14956	5.39441	0.00000
H	2.22945	5.38326	0.00000
O	-0.60903	6.89932	0.00000
O	1.43564	7.65194	0.00000
N	0.60902	6.74119	0.00000

X = CNE = -685.160098 a.u. ZPE = 0.187244 a.u. G = -685.017691 a.u.

C	-2.45154	0.12991	0.00000
H	-2.58950	1.20826	0.00000
C	-1.19154	-0.37884	0.00000

H	-1.06721	-1.45991	0.00000
C	0.00000	0.41564	0.00000
H	-0.12025	1.49695	0.00000
C	1.25527	-0.10632	0.00000
H	1.37258	-1.18814	0.00000
C	2.45145	0.68228	0.00000
H	2.33332	1.76404	0.00000
C	3.70604	0.16263	0.00000
H	3.83214	-0.91781	0.00000
C	4.89529	0.96686	0.00000
H	4.77031	2.04628	0.00000
C	6.14831	0.45617	0.00000
H	6.31252	-0.61692	0.00000
C	-3.62276	-0.69769	0.00000
H	-3.49898	-1.77651	0.00000
C	-4.87746	-0.21601	0.00000
H	-5.16478	0.82494	0.00000
O	-5.83723	-2.32182	0.00000
O	-7.12588	-0.56502	0.00000
N	-6.02108	-1.10682	0.00000
C	7.31194	1.26721	0.00000
N	8.27462	1.91002	0.00000

X = Cl E = -1052.515017 a.u.   ZPE = 0.179123 a.u.   G = -1052.380348 a.u

C	-2.26590	1.48845	0.00000
H	-1.84590	2.49122	0.00000
C	-1.42858	0.41678	0.00000
H	-1.86473	-0.58052	0.00000
C	0.00000	0.50094	0.00000
H	0.44309	1.49458	0.00000
C	0.81793	-0.58535	0.00000
H	0.36669	-1.57603	0.00000
C	2.24982	-0.52286	0.00000
H	2.70532	0.46528	0.00000
C	3.05367	-1.61563	0.00000
H	2.59679	-2.60301	0.00000
C	4.49333	-1.55623	0.00000
H	4.96392	-0.57742	0.00000
C	5.26259	-2.65412	0.00000
H	4.87347	-3.66385	0.00000
C	-3.69210	1.35664	0.00000
H	-4.12190	0.35933	0.00000
C	-4.54299	2.39898	0.00000
H	-4.27329	3.44451	0.00000
O	-6.42337	1.05116	0.00000
O	-6.66559	3.21658	0.00000
N	-5.97505	2.19654	0.00000
Cl	7.00041	-2.59632	0.00000

X = H   E = -592.891494 a.u.   ZPE = 0.187923 a.u.   G = -592.745631 a.u

C	-1.14437	0.96696	0.00000
H	-2.10409	0.45604	0.00000
C	0.00000	0.23187	0.00000
H	0.95247	0.75906	0.00000
C	0.05012	-1.19833	0.00000
H	-0.89749	-1.73308	0.00000
C	1.20832	-1.91054	0.00000
H	2.15208	-1.36768	0.00000
C	1.28289	-3.34264	0.00000
H	0.34207	-3.88989	0.00000
C	2.44550	-4.03980	0.00000
H	3.38633	-3.49200	0.00000
C	2.53049	-5.48188	0.00000
H	1.58863	-6.02642	0.00000
C	3.68404	-6.16831	0.00000
H	4.64444	-5.66282	0.00000
C	-1.14482	2.39901	0.00000
H	-0.19133	2.91899	0.00000
C	-2.26080	3.15115	0.00000
H	-3.27718	2.78659	0.00000
O	-1.09039	5.14659	0.00000
O	-3.26877	5.19007	0.00000
N	-2.19006	4.59487	0.00000
H	3.69963	-7.25119	0.00000

X = OH   E = -668.141857 a.u.   ZPE = 0.193054 a.u.   G = -667.948803 a.u.

C	-4.04549	0.31106	0.00004
H	-3.96811	1.39709	0.00006
C	-2.89750	-0.41562	0.00002
H	-2.96021	-1.50195	0.00000
C	-1.59082	0.16711	0.00002
H	-1.53787	1.25489	0.00003
C	-0.42383	-0.53467	-0.00001
H	-0.46245	-1.62197	-0.00003
C	0.86611	0.07949	-0.00001
H	0.89349	1.16795	0.00001
C	2.05025	-0.59319	-0.00003
H	2.04334	-1.68038	-0.00005
C	3.31620	0.07179	-0.00003
H	3.33167	1.15780	-0.00002
C	4.50474	-0.56258	-0.00006
H	4.65831	-1.63124	-0.00007
O	5.71921	1.40551	-0.00002
O	6.77796	-0.49943	-0.00006
N	5.74549	0.17463	-0.00006
C	-5.37105	-0.24104	0.00005
H	-5.47293	-1.32508	0.00003
C	-6.47947	0.52591	0.00007

H	-6.43693	1.60920	0.00009
O	-7.75901	0.09088	0.00007
H	-7.78673	-0.87407	0.00006

X = NH2 E = -648.275955 a.u. ZPE = 0.205277 a.u. G = -648.114206 a.u.

C	-2.06726	-0.61305	0.00022
H	-2.07106	-1.70029	0.00095
C	-0.87350	0.04811	-0.00036
H	-0.89328	1.13703	-0.00114
C	0.40948	-0.57219	0.00024
H	0.44388	-1.65962	0.00108
C	1.58274	0.12585	-0.00014
H	1.52925	1.21402	-0.00125
C	2.88591	-0.45287	0.00042
H	2.95363	-1.53886	0.00126
C	4.03592	0.27992	-0.00061
H	3.94650	1.36588	-0.00308
C	5.36071	-0.25316	0.00279
H	5.47775	-1.33478	0.00189
C	6.46746	0.53397	0.00233
H	6.35953	1.61461	0.00216
C	-3.32292	0.06489	0.00012
H	-3.32472	1.15108	-0.00007
C	-4.52285	-0.55287	0.00023
H	-4.68921	-1.61953	0.00030
O	-5.70986	1.43063	-0.00038
O	-6.79435	-0.46027	0.00076
N	-5.75020	0.19857	0.00021
N	7.76346	0.10053	-0.04462
H	8.50713	0.73910	0.18078
H	7.96736	-0.87693	0.09344

Y = NO2, R = polyyne, *n* = 1

X = NO2 E = -486.421916 a.u. ZPE = 0.033486 a.u. G = -486.421646 a.u.

C	0.59857	-0.00011	-0.00004
C	-0.59857	-0.00010	-0.00003
O	-2.54060	0.73721	-0.80981
O	-2.54062	-0.73717	0.80981
O	2.54046	0.80989	0.73722
O	2.54075	-0.80975	-0.73715
N	2.00093	-0.00002	0.00001
N	-2.00092	-0.00001	-0.00003

X = CN E = -374.153181 a.u. ZPE = 0.030108 a.u. G = -374.153771 a.u.

C	0.16245	-0.00010	0.00005
C	-1.04039	-0.00010	0.00008
O	2.10672	1.09499	0.00000
O	2.10688	-1.09490	0.00000
N	1.56345	0.00002	-0.00007
C	-2.40608	0.00005	0.00001
N	-3.56412	0.00000	-0.00005

X = Cl E = -741.505178 a.u. ZPE = 0.022205 a.u. G = -741.513411 a.u

C	0.00000	0.27656	0.00000
C	-0.00032	-0.92324	0.00000
O	-1.09244	2.22750	0.00000
O	1.09361	2.22681	0.00000
N	0.00038	1.67307	0.00000
Cl	-0.00060	-2.55682	0.00000

X = H E = -281.893002 a.u. ZPE = 0.030648 a.u. G = -281.889748 a.u

C	0.00012	-0.87775	0.00000
C	-0.00017	-2.07492	0.00000
O	-1.09293	1.07454	0.00000
O	1.09302	1.07450	0.00000
N	0.00000	0.52323	0.00000
H	-0.00038	-3.13885	0.00000

X = OH E = -357.134348 a.u. ZPE = 0.035842 a.u. G = -357.128154 a.u.

C	1.44690	-0.01477	0.00007
C	0.24781	-0.02025	0.00046
O	-1.72017	-1.07875	-0.00008
O	-1.68143	1.10655	-0.00020
N	-1.14146	0.00327	0.00010
O	2.73572	-0.10126	-0.00035
H	3.14900	0.77493	0.00108

X = NH2 E = -337.280593 a.u. ZPE = 0.047307 a.u. G = -337.264251 a.u.

C	-0.21983	-0.00003	-0.00040
C	-1.42792	-0.00003	-0.00010
O	1.72040	-1.09500	0.00012
O	1.72035	1.09503	0.00013
N	1.15553	-0.00000	-0.00001
N	-2.74339	0.00001	-0.00027
H	-3.26222	0.86457	0.00149
H	-3.26226	-0.86454	0.00154

Y = NO2, R = polyyne, *n* = 2

X = NO2 E = -562.599703 a.u. ZPE = 0.043400 a.u. G = -562.593186 a.u.

C	-0.67596	-0.00035	-0.00108
C	-1.88374	-0.00161	-0.00158
O	-3.82402	-0.77680	0.77246

O	-3.82420	0.77753	-0.77119
N	-3.27797	-0.00023	-0.00005
C	0.67592	0.00149	0.00090
C	1.88369	0.00201	0.00106
O	3.82246	-0.77582	-0.77434
O	3.82583	0.77418	0.77362
N	3.27796	-0.00004	0.00002

X = CNE = -450.332107 a.u. ZPE = 0.040429 a.u. G = -450.325712 a.u.

C	-1.23805	-0.00056	0.00055
C	-2.45238	-0.00084	0.00027
C	0.11252	0.00043	0.00068
C	1.32086	0.00030	0.00055
O	3.26423	1.09487	-0.00040
O	3.26337	-1.09507	-0.00041
N	2.71581	0.00015	-0.00005
C	-3.81292	-0.00021	-0.00023
N	-4.97309	0.00084	-0.00058

X = Cl E = -817.681378 a.u. ZPE = 0.031119 a.u. G = -817.684904 a.u

C	1.05795	-0.00098	0.00015
C	2.26811	0.00092	-0.00007
C	-0.29536	-0.00022	0.00006
C	-1.50378	-0.00030	0.00006
O	-3.44926	1.09438	-0.00004
O	-3.44982	-1.09396	-0.00004
N	-2.89387	0.00007	-0.00001
Cl	3.89930	-0.00002	-0.00003

X = H E = -358.067632 a.u. ZPE = 0.040705 a.u. G = -358.057799 a.u

C	2.37302	0.00003	-0.00008
C	3.58029	-0.00000	0.00041
C	1.01488	0.00004	-0.00054
C	-0.19221	-0.00009	-0.00077
O	-2.13821	-1.09409	0.00028
O	-2.13808	1.09412	0.00028
N	-1.58423	0.00002	0.00004
H	4.64403	-0.00024	0.00105

X = OH E = -433.308947 a.u. ZPE = 0.045743 a.u. G = -433.296314 a.u.

C	0.30973	-0.01639	-0.00112
C	-0.90005	-0.00832	-0.00199
O	-2.85573	-1.08277	0.00098
O	-2.83272	1.10605	0.00085
N	-2.28475	0.00551	0.00011
C	1.66142	-0.02664	-0.00140
C	2.87045	0.00119	0.00013
O	4.15885	-0.08846	0.00116
H	4.58073	0.78375	0.00171

X = NH2 E = -413.454482 a.u. ZPE = 0.057211 a.u. G = -413.431350 a.u.

C	-1.63842	-0.00034	0.00019
C	-2.85713	0.00040	0.00000
C	-0.29485	-0.00048	0.00009
C	0.91933	0.00009	-0.00010
O	2.85919	-1.09553	0.00013
O	2.85916	1.09568	-0.00011
N	2.29583	0.00006	-0.00003
N	-4.17197	0.00007	-0.00053
H	-4.69348	-0.86355	0.00017
H	-4.69387	0.86347	0.00257

Y = NO2, R = polyyne, *n* = 3

X = NO2 E = -638.778694 a.u. ZPE = 0.053677 a.u. G = -638.765465 a.u.

C	1.95567	-0.00325	0.00046
C	3.16621	-0.00326	0.00065
C	0.60994	-0.00148	-0.00010
C	-0.60995	0.00084	-0.00068
O	5.10902	-0.77312	0.77418
O	5.10439	0.77639	-0.77440
N	4.55757	0.00020	0.00009
C	-1.95567	0.00288	-0.00124
C	-3.16621	0.00263	-0.00119
O	-5.10592	-0.77588	-0.77352
O	-5.10749	0.77365	0.77506
N	-4.55757	0.00002	0.00021

X = CN E = -526.511549 a.u. ZPE = 0.050380 a.u. G = -526.498575 a.u.

C	2.60154	0.00002	-0.00001
C	3.81904	0.00002	0.00001
C	1.25798	-0.00003	0.00003
C	0.03719	-0.00008	0.00007
C	-1.30883	-0.00010	0.00016
C	-2.51931	-0.00011	0.00019
O	-4.46166	-1.09501	-0.00010
O	-4.46145	1.09517	-0.00010
N	-3.91127	0.00003	-0.00003
C	5.17777	0.00003	-0.00003
N	6.33879	0.00002	-0.00008

X = Cl E = -893.859294 a.u. ZPE = 0.041971 a.u. G = -893.855091 a.u

C	-2.37945	-0.00030	0.01702
C	-3.59160	0.00011	-0.01345
C	-1.03168	-0.00031	0.00489

C	0.18862	-0.00014	0.01159
C	1.53506	-0.00006	0.00597
C	2.74628	0.00002	0.00638
O	4.69033	-1.09454	-0.00645
O	4.69025	1.09472	-0.00645
N	4.13414	0.00007	-0.00278
Cl	-5.22277	0.00013	-0.00422

X = H E = -434.245457 a.u. ZPE = 0.050943 a.u. G = -434.228751 a.u.

C	-3.78534	-0.00004	0.00006
C	-4.99497	0.00009	0.00010
C	-2.43272	-0.00010	-0.00008
C	-1.21388	-0.00004	-0.00009
C	0.13388	-0.00004	-0.00013
C	1.34444	-0.00002	-0.00013
O	3.28854	-1.09452	0.00008
O	3.28847	1.09459	0.00008
N	2.73342	0.00002	0.00003
H	-6.05851	0.00023	0.00019

X = OH E = -509.486026 a.u. ZPE = 0.056286 a.u. G = -509.466280 a.u.

C	-3.03273	-0.02287	-0.00035
C	-4.24357	0.00900	0.00006
C	-1.68538	-0.01602	-0.00073
C	-0.46400	-0.01450	-0.00040
C	0.88100	-0.00850	0.00000
C	2.09340	-0.00236	0.00013
O	4.02925	1.10507	0.00011
O	4.04376	-1.08452	0.00015
N	3.47756	0.00646	0.00013
O	-5.53279	-0.08324	0.00052
H	-5.95695	0.78781	0.00061

X = NH2 E = -489.631092 a.u. ZPE = 0.066970 a.u. G = -489.602108 a.u.

C	3.01264	-0.00017	0.00046
C	4.23327	0.00002	-0.00018
C	1.67337	-0.00030	0.00062
C	0.44736	-0.00036	0.00064
C	-0.89285	-0.00029	0.00065
C	-2.10796	-0.00024	0.00106
O	-4.04861	-1.09541	-0.00058
O	-4.04803	1.09593	-0.00068
N	-3.48631	0.00012	-0.00016
N	5.54869	0.00031	-0.00303
H	6.07052	0.86377	0.00691
H	6.07093	-0.86291	0.00596

Y = NO2, R = polyyne, *n* = 4

X = NO2 E = -714.958181 a.u. ZPE = 0.062775 a.u. G = -714.939402 a.u.

C	3.23609	-0.00959	0.00672
C	4.44773	-0.00283	0.00098
C	1.89251	-0.01496	0.00732
C	0.66923	-0.01970	0.00163
C	-0.66922	-0.02144	-0.00329
C	-1.89252	-0.02028	-0.00612
O	6.39895	-0.97993	0.45465
O	6.37803	1.01091	-0.45983
N	5.83752	0.00981	-0.00170
C	-3.23608	-0.01449	-0.00613
C	-4.44775	-0.00638	-0.00362
O	-6.39759	-0.44035	-0.99185
O	-6.37943	0.47402	0.99895
N	-5.83748	0.01029	0.00167

X = CN E = -602.691224 a.u. ZPE = 0.059325 a.u. G = -602.673123 a.u.

C	-3.94167	-0.00003	0.00009
C	-5.16049	-0.00001	0.00002
C	-2.60071	-0.00001	0.00014
C	-1.37636	-0.00006	0.00014
C	-0.03807	-0.00001	0.00009
C	1.18528	0.00003	0.00010
C	2.52928	0.00002	0.00008
C	3.74079	0.00001	0.00004
O	5.68278	-1.09512	-0.00010
O	5.68279	1.09512	-0.00010
N	5.13101	0.00000	-0.00007
C	-6.51845	-0.00001	-0.00010
N	-7.67989	0.00005	-0.00020

X = Cl E = -970.038057 a.u. ZPE = 0.051650 a.u. G = -970.027392 a.u

C	-3.69023	-0.00003	0.00001
C	-4.90355	0.00001	-0.00001
C	-2.34414	-0.00007	0.00004
C	-1.12075	-0.00006	0.00006
C	0.21881	-0.00006	0.00007
C	1.44262	-0.00004	0.00005
C	2.78633	-0.00002	0.00003
C	3.99868	0.00001	0.00002
O	5.94180	1.09491	-0.00006
O	5.94182	-1.09485	-0.00006
N	5.38546	0.00003	0.00002
Cl	-6.53493	0.00005	-0.00004

X = H   E = -510.424197 a.u.   ZPE = 0.059767 a.u.   G = -510.402286 a.u.

C	-5.15414	0.00000	-0.00001
C	-6.36466	0.00003	0.00009
C	-3.80318	0.00001	-0.00002
C	-2.58132	-0.00003	0.00000
C	-1.24042	-0.00001	-0.00006
C	-0.01735	-0.00002	-0.00008
C	1.32698	-0.00001	-0.00008
C	2.53894	-0.00000	-0.00006
O	4.48206	1.09487	0.00009
O	4.48208	-1.09484	0.00009
N	3.92654	0.00001	-0.00005
H	-7.42810	0.00002	0.00016

X = OH   E = -585.664278 a.u.   ZPE = 0.065232 a.u.   G = -585.639239 a.u.

C	4.37723	-0.01961	-0.00121
C	5.58863	0.01268	0.00178
C	3.03107	-0.01517	-0.00351
C	1.80692	-0.01366	-0.00377
C	0.46823	-0.01202	-0.00378
C	-0.75663	-0.00738	-0.00291
C	-2.09909	-0.00358	-0.00154
C	-3.31239	0.00111	0.00005
O	-5.25950	-1.08598	0.00247
O	-5.25040	1.10411	0.00198
N	-4.69640	0.00669	0.00161
O	6.87847	-0.07969	0.00447
H	7.30248	0.79135	0.00683

X = NH2   E = -565.809106 a.u.   ZPE = 0.075570 a.u.   G = -565.775550 a.u.

C	-4.35927	0.00055	-0.00018
C	-5.58040	0.00053	0.00023
C	-3.02103	0.00070	-0.00026
C	-1.79232	-0.00054	-0.00032
C	-0.45822	-0.00014	-0.00048
C	0.76946	-0.00120	-0.00019
C	2.10886	-0.00100	0.00007
C	3.32399	-0.00076	0.00129
O	5.26396	1.09632	-0.00049
O	5.26572	-1.09486	0.00022
N	4.70388	0.00028	0.00027
N	-6.89653	-0.00016	-0.00984
H	-7.41714	-0.86356	0.03041
H	-7.41820	0.86222	0.03768

Y = NO2, R = polyyne, *n* = 5

X = NO2   E = -791.137818 a.u.   ZPE = 0.071645 a.u.   G = -791.114606 a.u.

C	4.51647	-0.00021	-0.00125
C	5.72870	0.00017	-0.00135
C	3.17388	-0.00090	-0.00187
C	1.94919	-0.00253	-0.00021
C	0.61346	-0.00207	-0.00263
C	-0.61346	-0.00338	0.00249
C	-1.94919	-0.00264	-0.00005
C	-3.17389	-0.00174	0.00136
O	7.66936	-0.77376	0.77460
O	7.66997	0.77739	-0.77245
N	7.11765	0.00134	0.00038
C	-4.51647	-0.00069	0.00076
C	-5.72870	0.00026	0.00054
O	-7.67003	-0.77392	-0.77348
O	-7.66930	0.77802	0.77278
N	-7.11764	0.00158	-0.00013

X = CNE = -678.870931 a.u.   ZPE = 0.069679 a.u.   G = -678.845713 a.u.

C	-3.76474	-0.00073	0.00004
C	-4.97685	-0.00051	0.00002
C	-2.42178	-0.00121	0.00008
C	-1.19714	0.00063	0.00009
C	0.13882	-0.00252	0.00020
C	1.36594	0.00413	-0.00012
C	2.70129	-0.00031	0.00011
C	3.92718	0.00260	-0.00013
O	-6.91838	1.09524	-0.00007
O	-6.91903	-1.09509	-0.00007
N	-6.36598	-0.00009	-0.00002
C	5.26697	0.00108	-0.00008
C	6.48648	0.00004	-0.00006
C	7.84403	-0.00103	-0.00001
N	9.00571	-0.00193	0.00003

X = Cl E = -1046.217213 a.u.   ZPE = 0.062992 a.u.   G = -1046.198019 a.u

C	4.04439	-0.00002	-0.00001
C	5.25730	0.00000	-0.00004
C	2.70195	-0.00004	0.00001
C	1.47663	-0.00004	0.00002
C	0.14048	-0.00006	0.00002
C	-1.08678	-0.00006	0.00002
C	-2.42388	-0.00006	0.00003
C	-3.64850	-0.00003	0.00001
O	7.19996	1.09506	-0.00002
O	7.20001	-1.09497	-0.00002
N	6.64355	0.00003	0.00004
C	-4.99388	0.00002	-0.00001

C	-6.20757	0.00004	-0.00001
Cl	-7.83915	0.00003	-0.00001

X = H E = -586.603346 a.u. ZPE = 0.069580 a.u. G = -586.575123 a.u

C	-2.54509	0.00023	0.00408
C	-3.75775	0.00009	0.00053
C	-1.20228	-0.00150	0.00843
C	0.02261	0.00058	0.00925
C	1.35942	-0.00359	0.00815
C	2.58592	0.00390	0.00581
C	3.92437	-0.00093	0.00301
C	5.14745	0.00136	-0.00090
O	-5.70033	-1.09487	-0.00607
O	-5.70039	1.09516	-0.00606
N	-5.14457	0.00018	-0.00424
C	6.49769	0.00016	-0.00537
C	7.70863	-0.00064	-0.00962
H	8.77199	-0.00151	-0.01344

X = OH E = -661.843042 a.u. ZPE = 0.073924 a.u. G = -661.813803 a.u.

C	5.70509	-0.01699	0.00063
C	6.91674	0.01516	0.00235
C	4.35927	-0.01377	-0.00093
C	3.13410	-0.01274	-0.00275
C	1.79753	-0.01040	-0.00160
C	0.56942	-0.01164	-0.00595
C	-0.76582	-0.00641	-0.00355
C	-1.99203	-0.00573	-0.00347
C	-3.33346	-0.00153	-0.00154
C	-4.54713	0.00208	0.00000
O	-6.48545	1.10424	0.00282
O	-6.49338	-1.08611	0.00323
N	-5.93120	0.00701	0.00214
O	8.20698	-0.07704	0.00401
H	8.63078	0.79409	0.00533

X = NH2 E = -641.987760 a.u. ZPE = 0.086656 a.u. G = -641.945623 a.u.

C	-3.34180	-0.00097	0.00014
C	-4.55674	-0.00034	0.00027
C	-2.00241	-0.00188	-0.00042
C	-0.77435	-0.00032	-0.00088
C	0.55808	-0.00371	-0.00139
C	1.78887	0.00343	-0.00168
C	3.12108	-0.00124	-0.00200
C	4.35066	0.00265	-0.00205
O	-6.49859	-1.09477	0.00134
O	-6.49737	1.09628	0.00108
N	-5.93789	0.00044	0.00094
C	5.68879	0.00134	-0.00218
C	6.90993	0.00129	0.00089
N	8.22712	-0.00155	-0.01550
H	8.74712	0.85907	0.07066
H	8.74324	-0.86480	0.06769

Y = NO2, R = acene, *n* = 1

X = NO2 E = -641.431747 a.u. ZPE = 0.104527 a.u. G = -641.364074 a.u.

C	0.69469	1.22024	0.00006
C	-0.69489	1.22024	0.00005
C	-1.36250	0.00000	0.00002
C	-0.69488	-1.22024	0.00006
C	0.69469	-1.22024	0.00005
C	1.36234	0.00000	0.00012
H	1.25914	2.14209	0.00003
H	-1.25936	2.14208	0.00003
H	-1.25937	-2.14208	0.00004
H	1.25915	-2.14208	0.00003
O	-3.41094	1.08535	-0.00010
O	-3.41093	-1.08535	-0.00006
O	3.41118	1.08528	-0.00006
O	3.41117	-1.08528	-0.00010
N	-2.84875	0.00000	0.00004
N	2.84874	0.00000	-0.00001

X = CN E = -529.136270 a.u. ZPE = 0.100927 a.u. G = -529.070134 a.u.

C	1.08356	1.21704	0.00002
C	-0.30460	1.21870	0.00003
C	-0.97499	0.00001	0.00002
C	-0.30458	-1.21869	0.00002
C	1.08356	-1.21702	0.00000
C	1.78187	0.00003	-0.00000
H	1.63029	2.15116	0.00002
H	-0.86846	2.14108	0.00001
H	-0.86845	-2.14107	0.00001
H	1.63034	-2.15111	-0.00000
O	-3.02277	1.08559	-0.00004
O	-3.02274	-1.08562	-0.00001
C	3.21386	0.00001	-0.00001
N	4.36888	-0.00003	-0.00002
N	-2.45913	-0.00001	0.00001

X = Cl E = -896.495961 a.u. ZPE = 0.092844 a.u. G = -896.437314 a.u

C	-0.96247	1.21621	0.00002
C	0.42698	1.21657	0.00006
C	1.10233	0.00001	0.00008

C	0.42696	-1.21657	0.00005
C	-0.96246	-1.21620	0.00001
C	-1.64420	0.00002	-0.00000
H	-1.51278	2.14784	0.00001
H	0.98748	2.14117	0.00009
H	0.98750	-2.14115	0.00006
H	-1.51281	-2.14780	-0.00001
O	3.14739	1.08537	-0.00012
O	3.14738	-1.08539	-0.00009
Cl	-3.39350	-0.00001	-0.00004
N	2.57988	-0.00000	0.00012

X = H E = -436.874726 a.u. ZPE = 0.102523 a.u. G = -436.804235 a.u

C	-1.82148	-1.21013	0.00002
C	-0.43013	-1.21862	-0.00004
C	0.24180	-0.00001	-0.00007
C	-0.43014	1.21863	-0.00004
C	-1.82146	1.21014	0.00002
C	-2.51581	-0.00001	0.00005
H	-2.36262	-2.14873	0.00005
H	0.13471	-2.14051	-0.00008
H	0.13474	2.14050	-0.00006
H	-2.36264	2.14872	0.00005
O	2.29129	-1.08475	0.00009
O	2.29128	1.08476	0.00008
H	-3.59976	0.00002	0.00009
N	1.72262	-0.00000	-0.00014

X = OH E = -512.124155 a.u. ZPE = 0.106528 a.u. G = -512.051019 a.u.

C	-1.37369	1.22628	0.00001
C	0.01112	1.21987	0.00004
C	0.68850	0.00069	0.00005
C	0.00333	-1.21078	0.00005
C	-1.38422	-1.20224	0.00002
C	-2.07529	0.01507	-0.00001
H	-1.92921	2.15544	-0.00002
H	0.57553	2.14210	0.00006
H	0.55874	-2.13842	0.00006
H	-1.93128	-2.13954	-0.00001
O	2.73468	1.07547	-0.00005
O	2.72422	-1.09508	-0.00007
N	2.15787	-0.00680	0.00005
O	-3.43173	0.08619	-0.00005
H	-3.81480	-0.79798	-0.00007

X = NH2 E = -492.256203 a.u. ZPE = 0.118963 a.u. G = -492.170733 a.u.

C	-1.36329	1.21243	-0.00424
C	0.01983	1.21343	-0.00277
C	0.70791	-0.00000	-0.00178
C	0.01984	-1.21341	-0.00267
C	-1.36330	-1.21244	-0.00415
C	-2.08101	-0.00003	-0.00362
H	-1.90192	2.15378	-0.00894
H	0.57788	2.13971	-0.00137
H	0.57789	-2.13970	-0.00123
H	-1.90187	-2.15382	-0.00864
O	2.74485	1.08577	0.00438
O	2.74486	-1.08576	0.00409
N	-3.45801	0.00001	-0.04781
H	-3.94428	-0.84886	0.19257
H	-3.94425	0.84891	0.19246
N	2.16928	-0.00000	0.00248

Y = NO2, R = acene, *n* = 2

X = NO2 E = -795.112684 a.u. ZPE = 0.151204 a.u. G = -795.001599 a.u.

C	2.49589	0.11735	-0.00000
C	1.61818	-0.93691	0.00020
C	0.22668	-0.67908	0.00014
C	-0.22668	0.67908	-0.00013
C	0.72841	1.73158	-0.00033
C	2.07316	1.46361	-0.00027
H	-0.37966	-2.75785	0.00055
H	1.99473	-1.95129	0.00040
C	-0.72842	-1.73159	0.00034
C	-1.61818	0.93691	-0.00018
H	0.37966	2.75785	-0.00053
H	2.81368	2.25085	-0.00042
C	-2.49589	-0.11735	0.00002
C	-2.07316	-1.46361	0.00028
H	-1.99473	1.95129	-0.00038
H	-2.81369	-2.25084	0.00043
O	-4.30211	1.34055	-0.00030
O	-4.71172	-0.79044	0.00009
O	4.30211	-1.34055	0.00027
O	4.71172	0.79044	-0.00015
N	-3.95119	0.16834	-0.00004
N	3.95119	-0.16834	0.00006

X = CN E = -682.816264 a.u. ZPE = 0.147565 a.u. G = -682.706999 a.u.

C	-2.92309	0.07175	0.00000
C	-2.01084	-0.96722	0.00000
C	-0.62139	-0.70241	0.00001
C	-0.17531	0.65730	0.00000
C	-1.13733	1.70283	-0.00000
C	-2.47854	1.42428	-0.00000
H	0.00057	-2.77702	0.00000

H	-2.35908	-1.99314	0.00000
C	0.34172	-1.74810	0.00001
C	1.21418	0.92468	0.00000
H	-0.79507	2.73142	0.00000
H	-3.21113	2.22125	-0.00000
C	2.10039	-0.12280	0.00002
C	1.68443	-1.47176	0.00000
H	1.58248	1.94209	0.00000
H	2.42952	-2.25466	-0.00001
O	3.89609	1.34653	0.00001
O	4.31920	-0.78218	-0.00002
C	-4.32720	-0.20653	-0.00000
N	-5.46218	-0.42277	-0.00001
N	3.55194	0.17179	-0.00001

X = Cl E = -1050.174739 a.u. ZPE = 0.139419 a.u. G = -1050.073058 a.u

C	2.77181	0.07040	-0.00005
C	1.87701	-0.97034	0.00017
C	0.48568	-0.70123	0.00013
C	0.03880	0.65823	-0.00014
C	1.00397	1.70004	-0.00035
C	2.34676	1.41881	-0.00031
H	-0.14177	-2.77412	0.00056
H	2.22733	-1.99497	0.00037
C	-0.48043	-1.74428	0.00035
C	-1.34930	0.92802	-0.00017
H	0.66682	2.73059	-0.00055
H	3.08401	2.21098	-0.00047
C	-2.23968	-0.11694	0.00002
C	-1.82284	-1.46592	0.00031
H	-1.71519	1.94639	-0.00036
H	-2.56816	-2.24870	0.00049
O	-4.03235	1.35490	-0.00019
O	-4.45853	-0.77302	0.00017
Cl	4.49382	-0.26121	0.00000
N	-3.68737	0.17922	0.00003

X = H E = -590.553022 a.u. ZPE = 0.149066 a.u. G = -590.439561 a.u

C	-3.58791	0.17962	-0.00005
C	-2.62425	1.16029	0.00017
C	-1.24734	0.82155	0.00014
C	-0.87871	-0.56196	-0.00013
C	-1.89848	-1.55074	-0.00035
C	-3.22312	-1.18777	-0.00032
H	-0.50403	2.85455	0.00058
H	-2.90702	2.20743	0.00038
C	-0.22307	1.80723	0.00036
C	0.49366	-0.90782	-0.00016
H	-1.61329	-2.59710	-0.00056
H	-3.99564	-1.94790	-0.00049
C	1.44142	0.08518	0.00001
C	1.10217	1.45626	0.00032
H	0.80213	-1.94503	-0.00036
H	1.89014	2.19597	0.00050
O	3.14887	-1.48624	-0.00006
O	3.69475	0.61370	0.00003
H	-4.63734	0.45166	-0.00002
N	2.86998	-0.29291	0.00003

X = OH E = -665.802059 a.u. ZPE = 0.153036 a.u. G = -665.686012 a.u.

C	3.18260	-0.02283	0.00002
C	2.25323	-1.04089	0.00021
C	0.86904	-0.74677	0.00013
C	0.44758	0.62197	-0.00016
C	1.43793	1.64274	-0.00034
C	2.77133	1.33403	-0.00026
H	0.19165	-2.80453	0.00053
H	2.57556	-2.07811	0.00042
C	-0.12217	-1.76658	0.00031
C	-0.93181	0.92138	-0.00024
H	1.12161	2.67992	-0.00056
H	3.53292	2.10398	-0.00040
C	-1.84854	-0.10287	-0.00006
C	-1.45858	-1.45947	0.00023
H	-1.27449	1.94792	-0.00045
H	-2.21929	-2.22737	0.00037
O	-3.60561	1.40804	-0.00014
O	-4.07925	-0.70997	0.00028
N	-3.28470	0.22442	-0.00015
O	4.52799	-0.23517	0.00009
H	4.71624	-1.18016	0.00028

X = NH2 E = -645.932681 a.u. ZPE = 0.165448 a.u. G = -645.804353 a.u.

C	-3.19095	0.03180	-0.00267
C	-2.24061	1.04161	-0.00224
C	-0.86112	0.74685	-0.00398
C	-0.43451	-0.62177	-0.00285
C	-1.42763	-1.63892	-0.00285
C	-2.75947	-1.32724	-0.00536
H	-0.17985	2.80432	-0.00545
H	-2.55681	2.08011	-0.00367
C	0.13350	1.76616	-0.00362
C	0.94277	-0.92183	-0.00041
H	-1.11453	-2.67736	-0.00251
H	-3.50415	-2.11639	-0.01389
C	1.86212	0.10197	0.00016
C	1.46926	1.45890	-0.00161
H	1.28507	-1.94855	0.00089

H	2.22947	2.22731	-0.00172
O	3.61739	-1.40912	0.00460
O	4.09138	0.70896	0.00421
N	-4.54707	0.30788	-0.05268
H	-5.17825	-0.41497	0.25590
H	-4.84012	1.23921	0.19867
N	3.29404	-0.22469	0.00326

Y = NO2, R = acene, *n* = 3

X = NO2 E = -948.786294 a.u. ZPE = 0.197487 a.u. G = -948.632430 a.u.

C	-3.69120	-0.10698	0.00003
C	-2.70861	0.84140	-0.00005
C	-1.34355	0.43692	-0.00005
C	-1.03584	-0.97303	0.00000
C	-2.11345	-1.91330	0.00006
C	-3.41411	-1.50087	0.00006
C	-0.30215	1.37318	-0.00007
C	0.30209	-1.37316	-0.00000
C	1.34348	-0.43690	-0.00002
C	1.03577	0.97305	-0.00002
C	2.11339	1.91332	0.00001
H	1.88081	2.97215	0.00003
C	3.41405	1.50088	0.00003
C	3.69115	0.10699	0.00006
C	2.70855	-0.84138	-0.00001
H	-0.54163	2.43131	-0.00009
H	-2.97215	1.89087	-0.00011
H	-1.88086	-2.97214	0.00009
H	-4.23761	-2.20060	0.00009
H	0.54157	-2.43128	0.00000
H	4.23755	2.20061	0.00002
H	2.97208	-1.89085	-0.00004
O	5.96359	0.53894	0.00021
O	5.33009	-1.53672	-0.00023
O	-5.32991	1.53670	-0.00008
O	-5.96344	-0.53900	0.00007
N	5.10365	-0.33324	-0.00001
N	-5.10361	0.33323	0.00001

X = CNE = -836.489413 a.u. ZPE = 0.193931 a.u. G = -836.337320 a.u.

C	4.12581	0.02419	0.00001
C	3.10835	-0.90171	0.00001
C	1.74695	-0.48560	-0.00000
C	1.45208	0.92643	0.00001
C	2.53881	1.85520	0.00001
C	3.83335	1.42765	0.00001
C	0.69461	-1.40979	0.00001
C	0.11848	1.34136	0.00001
C	-0.93306	0.41691	0.00001
C	-0.63907	-0.99599	0.00001
C	-1.72702	-1.92466	0.00003
H	-1.50564	-2.98602	0.00003
C	-3.02323	-1.49867	0.00003
C	-3.28671	-0.10188	-0.00002
C	-2.29367	0.83570	-0.00003
H	0.92153	-2.47091	0.00001
H	3.33831	-1.96068	-0.00001
H	2.31674	2.91654	0.00002
H	4.65346	2.13443	0.00001
H	-0.10886	2.40232	-0.00001
H	-3.85391	-2.19002	0.00005
H	-2.54554	1.88813	-0.00008
O	-5.56292	-0.51033	-0.00005
O	-4.90767	1.55871	0.00004
C	5.49001	-0.40660	-0.00001
N	6.59549	-0.74355	-0.00004
N	-4.69340	0.35268	-0.00003

X = Cl E = -1203.847095 a.u. ZPE = 0.185726 a.u. G = -1203.702668 a.u

C	3.96463	0.03200	-0.00001
C	2.96802	-0.90057	-0.00003
C	1.60313	-0.48291	-0.00002
C	1.30579	0.92846	0.00002
C	2.39221	1.85636	0.00003
C	3.68890	1.42843	0.00001
C	0.54943	-1.40523	-0.00001
C	-0.02757	1.34448	0.00004
C	-1.08015	0.42136	0.00003
C	-0.78430	-0.99109	0.00002
C	-1.87332	-1.91890	0.00004
H	-1.65223	-2.98043	0.00005
C	-3.16966	-1.49337	0.00004
C	-3.43479	-0.09651	0.00005
C	-2.43993	0.84016	0.00005
H	0.77480	-2.46673	-0.00002
H	3.20254	-1.95791	-0.00006
H	2.17255	2.91840	0.00004
H	4.51182	2.13146	0.00002
H	-0.25379	2.40585	0.00005
H	-3.99983	-2.18538	0.00003
H	-2.69099	1.89285	0.00005
O	-5.71017	-0.50485	-0.00008
O	-5.05481	1.56418	-0.00012
Cl	5.64430	-0.47716	-0.00003
N	-4.83892	0.35757	0.00005

X = H E = -744.224811 a.u. ZPE = 0.195402 a.u. G = -744.068573 a.u

C	-4.75563	0.28944	-0.00005
C	-3.69905	1.15785	-0.00004
C	-2.35479	0.67566	-0.00003
C	-2.13317	-0.75032	0.00002
C	-3.26578	-1.62165	0.00002
C	-4.53677	-1.11825	-0.00001
C	-1.25295	1.54003	-0.00002
C	-0.82187	-1.23498	0.00006
C	0.27756	-0.36886	0.00004
C	0.05731	1.05760	0.00001
C	1.19419	1.92637	0.00003
H	1.02932	2.99818	0.00003
C	2.46635	1.43343	0.00004
C	2.65698	0.02416	0.00006
C	1.61386	-0.85850	0.00007
H	-1.42254	2.61217	-0.00004
H	-3.86666	2.22962	-0.00009
H	-3.09684	-2.69322	0.00005
H	-5.38847	-1.78881	0.00001
H	-0.65128	-2.30685	0.00007
H	3.33202	2.08040	0.00002
H	1.80893	-1.92301	0.00008
O	4.95079	0.31167	-0.00006
O	4.18757	-1.71986	-0.00016
H	-5.77108	0.66889	-0.00007
N	4.03460	-0.50338	0.00007

X = OH E = -819.473819 a.u. ZPE = 0.199355 a.u. G = -819.315036 a.u.

C	4.36480	0.10516	-0.00000
C	3.33642	1.01143	0.00000
C	1.98152	0.56888	0.00000
C	1.71256	-0.84942	-0.00000
C	2.82107	-1.75330	-0.00001
C	4.10536	-1.29762	-0.00001
C	0.90353	1.46359	0.00001
C	0.39104	-1.29655	-0.00000
C	-0.68551	-0.39982	0.00000
C	-0.42030	1.01870	0.00001
C	-1.53151	1.91999	0.00001
H	-1.33597	2.98662	0.00002
C	-2.81796	1.46447	0.00001
C	-3.05200	0.06225	0.00000
C	-2.03332	-0.85035	0.00000
H	1.10115	2.53084	0.00001
H	3.54170	2.07797	0.00001
H	2.62323	-2.81964	-0.00001
H	4.95041	-1.97507	-0.00001
H	0.19118	-2.36336	-0.00001
H	-3.66343	2.13762	0.00001

X = NH2 E = -799.604002 a.u. ZPE = 0.211780 a.u. G = -799.432900 a.u.

C	-4.37467	0.11228	-0.00163
C	-3.32724	1.00846	0.00002
C	-1.97647	0.56736	-0.00289
C	-1.70172	-0.85057	-0.00153
C	-2.81134	-1.75206	-0.00155
C	-4.09408	-1.29451	-0.00515
C	-0.89560	1.46208	-0.00267
C	-0.38152	-1.29773	-0.00027
C	0.69569	-0.40055	-0.00040
C	0.42763	1.01824	-0.00149
C	1.53950	1.91966	-0.00157
H	1.34352	2.98626	-0.00270
C	2.82620	1.46510	-0.00046
C	3.06248	0.06290	0.00080
C	2.04244	-0.84982	0.00073
H	-1.09362	2.52930	-0.00396
H	-3.52698	2.07550	-0.00007
H	-2.61573	-2.81906	-0.00185
H	-4.92380	-1.99391	-0.01606
H	-0.18115	-2.36464	0.00052
H	3.67086	2.13928	-0.00058
H	2.26873	-1.90813	0.00151
O	5.34498	0.41900	0.00201
O	4.64193	-1.63454	0.00366
N	-5.69912	0.52203	-0.05701
H	-6.39342	-0.12665	0.28066
H	-5.89221	1.48091	0.18891
N	4.45064	-0.42197	0.00224

Y = NO2, R = acene, *n* = 4

X = NO2 E = -1102.456463 a.u. ZPE = 0.243690 a.u. G = -1102.259792 a.u.

C	0.11095	-0.71655	-0.00000
C	-0.11095	0.71655	0.00001
C	-1.43307	1.19821	0.00001
C	1.00095	1.58370	0.00001
C	1.43307	-1.19821	-0.00001
C	-1.00095	-1.58370	-0.00000
C	-2.52321	0.33238	0.00001
C	2.30270	1.10042	0.00000
C	2.52321	-0.33238	-0.00000
C	-2.30270	-1.10042	0.00000
C	-3.86405	0.82113	0.00001
C	3.44404	1.96916	-0.00000
C	3.86405	-0.82113	-0.00000
C	-3.44404	-1.96916	0.00001
C	-4.90287	-0.06147	0.00001
C	4.71281	1.47487	-0.00000

C	4.90287	0.06147	-0.00000
C	-4.71281	-1.47487	0.00001
H	-1.60501	2.26954	0.00002
H	0.83071	2.65542	0.00001
H	1.60501	-2.26954	-0.00001
H	-0.83071	-2.65542	-0.00001
H	-4.06047	1.88531	0.00002
H	3.28005	3.04092	-0.00000
H	4.06047	-1.88531	-0.00001
H	-3.28005	-3.04092	0.00001
H	5.57989	2.11978	-0.00000
H	-5.57989	-2.11978	0.00001
O	7.19617	0.35188	-0.00001
O	6.43616	-1.68124	-0.00000
O	-6.43616	1.68124	-0.00002
O	-7.19617	-0.35188	-0.00002
N	6.28288	-0.46548	0.00000
N	-6.28288	0.46548	0.00001

X = CN E = -990.159273 a.u. ZPE = 0.240135 a.u. G = -989.964449 a.u.

C	-0.31069	0.67863	0.00000
C	-0.51766	-0.75676	-0.00000
C	-1.83462	-1.25235	-0.00000
C	0.60440	-1.61104	-0.00000
C	1.00611	1.17518	0.00000
C	-1.43202	1.53267	0.00001
C	-2.93514	-0.39989	-0.00000
C	1.90052	-1.11339	0.00000
C	2.10575	0.32183	0.00000
C	-2.72849	1.03430	0.00000
C	-4.27197	-0.90019	-0.00000
C	3.05188	-1.96905	0.00000
C	3.44092	0.82538	-0.00000
C	-3.87789	1.89091	0.00000
C	-5.34368	-0.04207	0.00000
C	4.31493	-1.46075	0.00000
C	4.49019	-0.04511	0.00000
C	-5.13964	1.38179	0.00000
H	-1.99333	-2.32590	-0.00000
H	0.44646	-2.68470	-0.00000
H	1.16582	2.24847	0.00000
H	-1.27453	2.60643	0.00001
H	-4.43401	-1.97167	0.00000
H	2.90001	-3.04262	0.00001
H	3.62495	1.89178	-0.00001
H	-3.72473	2.96444	-0.00001
H	5.18885	-2.09634	0.00001
H	-6.00375	2.03402	-0.00000
O	6.78604	-0.31048	0.00001
O	6.00365	1.71432	-0.00001
C	-6.67803	-0.55701	0.00000
N	-7.76091	-0.96143	-0.00000
N	5.86341	0.49670	-0.00001

X = Cl E = -1357.516518 a.u. ZPE = 0.231923 a.u. G = -1357.329371 a.u

C	-0.15675	0.68384	0.00001
C	-0.36675	-0.75102	-0.00000
C	-1.68458	-1.24539	-0.00001
C	0.75575	-1.60517	-0.00001
C	1.16020	1.17971	0.00001
C	-1.27762	1.53781	0.00001
C	-2.78528	-0.39389	-0.00001
C	2.05232	-1.10887	0.00000
C	2.25986	0.32615	0.00001
C	-2.57459	1.03959	0.00000
C	-4.12690	-0.89384	-0.00001
C	3.20360	-1.96494	0.00001
C	3.59484	0.82834	0.00001
C	-3.72208	1.89734	0.00000
C	-5.17606	-0.02597	-0.00000
C	4.46729	-1.45811	0.00001
C	4.64466	-0.04243	0.00001
C	-4.98663	1.38937	-0.00000
H	-1.84284	-2.31901	-0.00001
H	0.59750	-2.67888	-0.00001
H	1.32032	2.25308	0.00001
H	-1.12010	2.61171	0.00001
H	-4.29540	-1.96366	-0.00001
H	3.05091	-3.03849	0.00001
H	3.77937	1.89469	0.00000
H	-3.56982	2.97116	-0.00001
H	5.34036	-2.09485	0.00001
H	-5.85244	2.03879	-0.00000
O	6.93975	-0.30936	0.00001
O	6.15877	1.71597	-0.00002
Cl	-6.82342	-0.63341	0.00000
N	6.01633	0.49792	-0.00000

X = H E = -897.894004 a.u. ZPE = 0.241606 a.u. G = -897.695035 a.u

C	0.95513	-0.57125	0.00002
C	1.10277	0.87153	-0.00001
C	2.39866	1.42011	-0.00002
C	-0.05528	1.67729	-0.00001
C	-0.34045	-1.12194	0.00003
C	2.11095	-1.37649	0.00002
C	3.53548	0.61733	-0.00002
C	-1.32955	1.12693	0.00001
C	-1.47523	-0.31587	0.00002
C	3.38735	-0.82463	0.00000

C	4.85821	1.16746	-0.00002
C	-2.51651	1.93299	0.00002
C	-2.78784	-0.87409	0.00002
C	4.56955	-1.63443	-0.00000
C	5.95554	0.35637	-0.00000
C	-3.75756	1.37319	0.00002
C	-3.87416	-0.04901	0.00001
C	5.80945	-1.06532	-0.00000
H	2.51175	2.49973	-0.00004
H	0.05767	2.75681	-0.00002
H	-0.45535	-2.20114	0.00004
H	1.99838	-2.45618	0.00004
H	4.97016	2.24651	-0.00002
H	-2.40958	3.01211	0.00003
H	-2.92684	-1.94735	0.00001
H	4.45776	-2.71351	-0.00001
H	-4.65691	1.97215	0.00002
H	6.69564	-1.68963	-0.00000
O	-6.17834	0.12027	0.00001
O	-5.31238	-1.87014	-0.00006
H	6.95052	0.78681	0.00002
N	-5.22092	-0.64692	-0.00001

X = OH E = -973.142962 a.u. ZPE = 0.245460 a.u. G = -972.941582 a.u.

C	-0.73414	0.80748	0.00000
C	-0.55105	-0.63094	0.00000
C	0.75423	-1.15364	0.00001
C	-1.69072	-1.46107	0.00000
C	-2.04219	1.32728	-0.00000
C	0.40646	1.63776	0.00000
C	1.87244	-0.32307	0.00001
C	-2.97611	-0.93713	0.00000
C	-3.16254	0.50082	-0.00000
C	1.69299	1.11566	0.00000
C	3.19603	-0.85184	0.00001
C	-4.14018	-1.77481	-0.00000
C	-4.49520	1.01935	-0.00000
C	2.86173	1.94792	0.00001
C	4.26443	-0.00273	0.00001
C	-5.39278	-1.24493	-0.00000
C	-5.57066	0.17498	-0.00000
C	4.11529	1.41606	0.00001
H	0.89224	-2.23020	0.00001
H	-1.55574	-2.53821	0.00000
H	-2.17647	2.40440	-0.00000
H	0.27056	2.71464	0.00000
H	3.35892	-1.92173	0.00001
H	-4.00486	-2.85090	0.00000
H	-4.63917	2.09593	-0.00000
H	2.73070	3.02440	0.00000
H	-6.27683	-1.87066	-0.00000
H	5.00075	2.03541	0.00001
O	5.74204	-1.79128	-0.00002
O	6.56395	0.21784	-0.00002
N	5.62328	-0.57017	0.00001
O	-6.86921	0.59441	-0.00001
H	-6.90539	1.55735	-0.00001

X = NH2 E = -953.272902 a.u. ZPE = 0.257952 a.u. G = -953.059142 a.u.

C	-0.54253	-0.63186	0.00045
C	-0.72861	0.80687	-0.00063
C	-2.03624	1.32533	-0.00206
C	0.41284	1.63720	-0.00099
C	0.76197	-1.15365	0.00099
C	-1.68253	-1.46267	0.00072
C	-3.15935	0.49894	-0.00238
C	1.69953	1.11596	-0.00042
C	1.88083	-0.32271	0.00058
C	-2.96669	-0.93866	-0.00066
C	-4.48834	1.01524	0.00085
C	2.86841	1.94835	-0.00087
C	3.20360	-0.85071	0.00104
C	-4.13131	-1.77462	-0.00098
C	-5.58157	0.18172	-0.00157
C	4.12232	1.41713	-0.00033
C	4.27302	-0.00160	0.00062
C	-5.38230	-1.24333	-0.00544
H	-2.17092	2.40244	-0.00343
H	0.27673	2.71410	-0.00193
H	0.90051	-2.23027	0.00156
H	-1.54707	-2.53993	0.00156
H	-4.62685	2.09199	0.00151
H	2.73714	3.02485	-0.00169
H	3.36666	-1.92061	0.00166
H	-3.99779	-2.85120	-0.00151
H	5.00730	2.03717	-0.00069
H	-6.25226	-1.89208	-0.01812
O	6.57184	0.21977	0.00093
O	5.75052	-1.78976	0.00196
N	-6.88417	0.65963	-0.05968
H	-7.60797	0.05201	0.29266
H	-7.02474	1.62903	0.18135
N	5.63019	-0.56808	0.00119

Y = O^-, R = polyene, *n* = 1

X = NO2 E = -357.915075 a.u. ZPE = 0.046547 a.u. G = -357.897627 a.u.

C	1.39534	0.40076	0.00000
H	1.70953	-0.66031	0.00000

C	0.00000	0.60316	0.00000
H	-0.44284	1.58733	0.00000
O	2.25493	1.30232	0.00000
O	-0.52606	-1.63679	0.00000
O	-2.13876	-0.15276	0.00000
N	-0.90850	-0.43609	0.00000

X = CNE = -245.605155 a.u. ZPE = 0.042102 a.u. G = -245.590522 a.u.

C	-1.04736	-0.38413	0.00000
H	-0.70166	-1.44613	0.00000
C	0.00000	0.55199	0.00000
H	-0.23150	1.61246	0.00000
O	-2.27685	-0.16031	0.00000
C	1.34721	0.17107	0.00000
N	2.47841	-0.13106	0.00000

X = Cl E = -612.925766 a.u. ZPE = 0.033098 a.u. G = -612.920179 a.u

C	1.18747	-0.13958	0.00000
H	1.05899	-1.25011	0.00000
C	0.00000	0.55505	0.00000
H	-0.12824	1.62671	0.00000
O	2.36245	0.33618	0.00000
Cl	-1.58560	-0.32699	0.00000

X = H E = -153.289944 a.u. ZPE = 0.041501 a.u. G = -153.272668 a.u

C	0.00000	0.36675	0.00000
H	0.29089	1.45579	0.00000
C	1.09375	-0.48096	0.00000
H	0.97231	-1.56210	0.00000
O	-1.24063	0.10759	0.00000
H	2.09932	-0.06916	0.00000

X = OH E = -228.524481 a.u. ZPE = 0.045756 a.u. G = -228.505537 a.u.

C	-0.26725	-0.65807	-0.00000
H	0.69380	-1.26714	0.00000
C	0.00000	0.68277	-0.00000
H	-0.73858	1.47283	-0.00000
O	-1.37088	-1.30118	-0.00000
O	1.33816	1.20355	0.00000
H	1.91006	0.42712	0.00000

X = NH2 E = -208.653300 a.u. ZPE = 0.058690 a.u. G = -208.621364 a.u.

C	-0.68232	-0.29063	-0.00012
H	-0.44019	-1.39586	0.00013
C	0.45716	0.48885	-0.00034
H	0.38802	1.57423	-0.00026
N	1.81133	-0.05489	-0.00004
H	2.00123	-0.62984	0.81637
H	2.00089	-0.63282	-0.81462
O	-1.90978	0.03490	0.00018

Y = O^{-}, R = polyene, *n* = 2

X = NO2 E = -435.351332 a.u. ZPE = 0.079737 a.u. G = -435.304106 a.u.

C	-0.41828	-1.00049	0.00000
H	-1.45178	-1.31169	0.00000
C	0.00000	0.32176	0.00000
H	1.07804	0.47000	0.00000
C	-0.80661	1.44899	0.00000
H	-1.89049	1.36316	0.00000
C	-0.24802	2.75837	0.00000
H	0.86843	2.77204	0.00000
O	-0.85838	3.83538	0.00000
O	0.00412	-3.22984	0.00000
O	1.71631	-1.85948	0.00000
N	0.47669	-2.06196	0.00000

X = CNE = -323.039892 a.u. ZPE = 0.075383 a.u. G = -322.995535 a.u.

C	-1.14467	0.94863	0.00000
H	-2.12634	0.48479	0.00000
C	0.00000	0.16470	0.00000
H	0.94972	0.70276	0.00000
C	0.09345	-1.22766	0.00000
H	-0.80539	-1.84048	0.00000
C	1.34502	-1.88783	0.00000
H	2.21911	-1.18885	0.00000
O	1.56692	-3.11131	0.00000
C	-1.11633	2.35123	0.00000
N	-1.11961	3.51970	0.00000

X = Cl E = -690.367794 a.u. ZPE = 0.066513 a.u. G = -690.332151 a.u

C	-1.22645	-0.51995	0.00000
H	-1.44874	-1.57573	0.00000
C	0.00000	0.05716	0.00000
H	0.02129	1.14840	0.00000
C	1.27145	-0.57356	0.00000
H	1.34417	-1.65892	0.00000
C	2.46053	0.17121	0.00000
H	2.28834	1.27995	0.00000
O	3.64454	-0.23758	0.00000
Cl	-2.72909	0.46458	0.00000

X = H E = -230.734470 a.u. ZPE = 0.075253 a.u. G = -230.687166 a.u

C	2.45413	-0.22182	0.00000
H	2.81973	0.80192	0.00000
C	1.11800	-0.50813	0.00000
H	0.85477	-1.57074	0.00000
C	0.00000	0.36708	0.00000
H	0.15884	1.44464	0.00000
C	-1.31970	-0.10380	0.00000
H	-1.38828	-1.22451	0.00000
O	-2.39493	0.54482	0.00000
H	3.19983	-1.00989	0.00000

X = OH E = -305.973468 a.u. ZPE = 0.079406 a.u. G = -305.924498 a.u.

C	0.86600	1.61499	0.00000
H	1.94427	1.52611	0.00000
C	0.00000	0.57946	0.00000
H	-1.06417	0.84861	0.00000
C	0.25137	-0.83234	0.00000
H	1.27820	-1.19375	0.00000
C	-0.77994	-1.77193	0.00000
H	-1.80207	-1.30257	0.00000
O	-0.71864	-3.03044	0.00000
O	0.48118	2.97842	0.00000
H	-0.48107	2.99675	0.00000

X = NH2 E = -286.099357 a.u. ZPE = 0.092117 a.u. G = -286.037757 a.u.

C	1.75931	0.47253	0.00150
H	1.85305	1.55485	-0.03942
C	0.54202	-0.13157	0.01869
H	0.55572	-1.22774	0.05051
C	-0.76525	0.43977	-0.01289
H	-0.87885	1.52191	-0.05554
C	-1.92319	-0.34351	0.01192
H	-1.70850	-1.44594	0.04748
N	3.02777	-0.23065	-0.07087
H	3.44520	-0.36500	0.84953
H	2.89755	-1.15590	-0.46827
O	-3.12949	0.01363	-0.00044

Y = O$^-$, R = polyene, *n* = 3

X = NO2 E = -512.782971 a.u. ZPE = 0.112786 a.u. G = -512.706250 a.u.

C	-2.06742	-0.62826	0.00000
H	-2.77085	0.19055	0.00000
C	-0.69342	-0.47785	0.00000
H	-0.11785	-1.40032	0.00000
C	0.00000	0.73013	0.00000
H	-0.56441	1.66080	0.00000
C	1.40125	0.80430	0.00000
H	1.93053	-0.15112	0.00000
C	2.18776	1.94036	0.00000
H	1.73643	2.92943	0.00000
C	3.61294	1.86424	0.00000
H	4.01029	0.82066	0.00000
O	4.41078	2.80723	0.00000
O	-1.99027	-2.92763	0.00000
O	-3.93557	-1.91571	0.00000
N	-2.67862	-1.87979	0.00000

X = CN E = -400.473296 a.u. ZPE = 0.108589 a.u. G = -400.399183 a.u.

C	2.43969	1.13166	0.00000
H	2.28051	2.20560	0.00000
C	1.36054	0.26654	0.00000
H	1.59130	-0.79909	0.00000
C	0.00000	0.60646	0.00000
H	-0.26878	1.66165	0.00000
C	-1.02446	-0.34183	0.00000
H	-0.70729	-1.38796	0.00000
C	-2.39643	-0.12596	0.00000
H	-2.79644	0.88523	0.00000
C	-3.32402	-1.20244	0.00000
H	-2.84261	-2.21142	0.00000
O	-4.56195	-1.13582	0.00000
C	3.77575	0.69840	0.00000
N	4.89321	0.36223	0.00000

X = Cl E = -767.804987 a.u. ZPE = 0.099873 a.u. G = -767.739380 a.u

C	-2.46648	0.29631	0.00000
H	-2.77414	1.33099	0.00000
C	-1.19303	-0.16089	0.00000
H	-1.05864	-1.24253	0.00000
C	0.00000	0.61802	0.00000
H	-0.09524	1.70259	0.00000
C	1.26595	0.05844	0.00000
H	1.30521	-1.03492	0.00000
C	2.51115	0.70120	0.00000
H	2.56625	1.78786	0.00000
C	3.72560	-0.01962	0.00000
H	3.58507	-1.13085	0.00000
O	4.88937	0.42646	0.00000
Cl	-3.86486	-0.81092	0.00000

X = H E = -308.174031 a.u. ZPE = 0.108605 a.u. G = -308.096928 a.u

C	3.62409	-0.79874	0.00000
H	3.63801	-1.88524	0.00000
C	2.45569	-0.10063	0.00000
H	2.53511	0.98963	0.00000

C	1.12066	-0.60333	0.00000
H	0.98733	-1.68506	0.00000
C	0.00000	0.20566	0.00000
H	0.19055	1.28348	0.00000
C	-1.35621	-0.15933	0.00000
H	-1.63813	-1.21055	0.00000
C	-2.39059	0.79902	0.00000
H	-2.01832	1.85586	0.00000
O	-3.62504	0.61130	0.00000
H	4.58386	-0.29448	0.00000

X = OH E = -383.414606 a.u. ZPE = 0.112888 a.u. G = -383.335465 a.u.

C	0.16716	-3.04771	0.00000
H	-0.84136	-3.44026	0.00000
C	0.47514	-1.73487	0.00000
H	1.54122	-1.48361	0.00000
C	-0.42131	-0.61059	0.00000
H	-1.49025	-0.82035	0.00000
C	0.00000	0.69990	0.00000
H	1.08400	0.85415	0.00000
C	-0.76703	1.88384	0.00000
H	-1.85410	1.82612	0.00000
C	-0.17676	3.16037	0.00000
H	0.94425	3.13114	0.00000
O	-0.73305	4.28159	0.00000
O	1.10451	-4.09145	0.00000
H	1.98137	-3.69391	0.00000

X = NH2 E = -363.540510 a.u. ZPE = 0.125696 a.u. G = -363.448499 a.u.

C	3.01609	0.38928	0.01421
H	3.15695	1.46684	0.00052
C	1.77668	-0.15517	0.01127
H	1.72261	-1.24856	0.02163
C	0.51019	0.51988	-0.01745
H	0.52185	1.60921	-0.04760
C	-0.70403	-0.13043	-0.00121
H	-0.66024	-1.22419	0.02137
C	-2.00595	0.41189	-0.00943
H	-2.14352	1.49166	-0.02951
C	-3.15700	-0.39620	0.01005
H	-2.92890	-1.49376	0.03089
N	4.24238	-0.35446	-0.09289
H	4.84241	-0.22174	0.71862
H	4.05183	-1.34866	-0.16969
O	-4.35944	-0.04813	0.00741

Y = O$^-$, R = polyene, *n* = 4

X = NO2 E = -590.214004 a.u. ZPE = 0.146012 a.u. G = -590.107417 a.u.

C	0.56120	5.31041	0.00000
H	1.64506	5.04311	0.00000
C	-0.31634	4.18141	0.00000
H	-1.38573	4.37636	0.00000
C	0.16636	2.88962	0.00000
H	1.25284	2.77712	0.00000
C	-0.57865	1.69690	0.00000
H	-1.66474	1.77051	0.00000
C	0.00000	0.43455	0.00000
H	1.09058	0.39419	0.00000
C	-0.66649	-0.79456	0.00000
H	-1.75507	-0.78932	0.00000
C	-0.00173	-2.02244	0.00000
H	1.08531	-2.01910	0.00000
C	-0.60108	-3.26408	0.00000
H	-1.66791	-3.42875	0.00000
O	0.23809	6.50054	0.00000
O	1.39027	-4.41694	0.00000
O	-0.49753	-5.53048	0.00000
N	0.13905	-4.44855	0.00000

X = CNE = -477.905640 a.u. ZPE = 0.141817 a.u. G = -477.801697 a.u.

C	-0.80209	-4.68498	0.00000
H	-1.86977	-4.35615	0.00000
C	0.13225	-3.60898	0.00000
H	1.18997	-3.86057	0.00000
C	-0.27955	-2.28725	0.00000
H	-1.35960	-2.12111	0.00000
C	0.51905	-1.13634	0.00000
H	1.60076	-1.25969	0.00000
C	0.00000	0.15942	0.00000
H	-1.08880	0.24376	0.00000
C	0.71169	1.35482	0.00000
H	1.79969	1.31137	0.00000
C	0.09813	2.61952	0.00000
H	-0.99161	2.62931	0.00000
C	0.73060	3.84589	0.00000
H	1.81462	3.90379	0.00000
O	-0.55181	-5.89631	0.00000
C	0.03981	5.07049	0.00000
N	-0.51143	6.09775	0.00000

X = Cl E = -845.240154 a.u. ZPE = 0.133106 a.u. G = -845.144811 a.u

C	1.87965	4.60323	0.00000
H	2.84147	4.03215	0.00000
C	0.72742	3.77680	0.00000
H	-0.24460	4.26497	0.00000

C	0.81837	2.38645	0.00000
H	1.83326	1.97933	0.00000
C	-0.21302	1.45102	0.00000
H	-1.24072	1.81181	0.00000
C	0.00000	0.05889	0.00000
H	1.04454	-0.26206	0.00000
C	-0.94983	-0.94124	0.00000
H	-2.00260	-0.66407	0.00000
C	-0.62389	-2.33102	0.00000
H	0.43681	-2.57913	0.00000
C	-1.49898	-3.35994	0.00000
H	-2.57551	-3.27670	0.00000
O	1.92835	5.84462	0.00000
Cl	-0.96222	-5.05461	0.00000

X = H E = -385.610479 a.u. ZPE = 0.141946 a.u. G = -385.503481 a.u

C	-3.73187	-0.13192	0.00000
H	-3.60215	-1.24344	0.00000
C	-2.50882	0.58148	0.00000
H	-2.55730	1.66830	0.00000
C	-1.27304	-0.06800	0.00000
H	-1.31499	-1.16094	0.00000
C	0.00000	0.49094	0.00000
H	0.09009	1.57664	0.00000
C	1.18789	-0.27135	0.00000
H	1.05403	-1.35636	0.00000
C	2.48765	0.18376	0.00000
H	2.66639	1.25872	0.00000
C	3.62963	-0.67632	0.00000
H	3.40402	-1.74499	0.00000
C	4.93991	-0.32117	0.00000
H	5.24668	0.72094	0.00000
O	-4.88781	0.32780	0.00000
H	5.72758	-1.06573	0.00000

X = OH E = -460.852294 a.u. ZPE = 0.146252 a.u. G = -460.743182 a.u.

C	1.01761	-4.27198	0.00000
H	2.06688	-3.88133	0.00000
C	0.03690	-3.25439	0.00000
H	-1.00792	-3.55820	-0.00000
C	0.37504	-1.89590	0.00000
H	1.44771	-1.68065	0.00000
C	-0.46403	-0.79173	-0.00000
H	-1.54113	-0.95645	-0.00000
C	0.00000	0.54763	-0.00000
H	1.08676	0.66834	0.00000
C	-0.74440	1.70020	-0.00000
H	-1.83082	1.62250	-0.00000
C	-0.17686	3.02243	-0.00000
H	0.91616	3.07041	0.00000
C	-0.83997	4.19523	-0.00000
H	-1.91888	4.28232	-0.00000
O	0.84856	-5.50670	0.00000
O	-0.24313	5.45570	-0.00000
H	0.71197	5.33220	0.00000

X = NH2 E = -440.978596 a.u. ZPE = 0.159102 a.u. G = -440.856506 a.u.

C	-4.38310	-0.46378	0.00937
H	-4.13310	-1.55510	0.01799
C	-3.24638	0.37490	-0.00292
H	-3.41109	1.45048	-0.01193
C	-1.94249	-0.13698	-0.00271
H	-1.86800	-1.22845	0.00650
C	-0.73947	0.55185	-0.01162
H	-0.76423	1.64127	-0.02069
C	0.53021	-0.07975	-0.00760
H	0.51194	-1.17295	0.00337
C	1.76801	0.51260	-0.01512
H	1.82570	1.60073	-0.03235
C	3.00946	-0.21189	0.00707
H	2.92233	-1.30192	0.00528
C	4.25810	0.30560	0.01857
H	4.41771	1.38042	0.01899
N	5.46410	-0.45285	-0.09030
H	6.13172	-0.22918	0.64213
H	5.27972	-1.44989	-0.05751
O	-5.58593	-0.13510	0.01125

Y = O^{-}, R = polyene, *n* = 5

X = NO2 E = -667.644406 a.u. ZPE = 0.179283 a.u. G = -667.507891 a.u.

C	-0.11653	-4.15714	0.00000
H	0.97475	-4.11395	0.00000
C	-0.78614	-2.91798	0.00000
H	-1.87461	-2.92442	0.00000
C	-0.13114	-1.69646	0.00000
H	0.95993	-1.72343	0.00000
C	-0.72388	-0.42676	0.00000
H	-1.81117	-0.36882	0.00000
C	0.00000	0.76228	0.00000
H	1.08759	0.67595	0.00000
C	-0.52306	2.05528	0.00000
H	-1.60521	2.17300	0.00000
C	0.27530	3.20391	0.00000
H	1.35526	3.08122	0.00000
C	-0.18585	4.50037	0.00000
H	-1.22804	4.78160	0.00000
C	-0.68196	-5.41258	0.00000

H	-1.76153	-5.53861	0.00000
C	0.12181	-6.59790	0.00000
H	1.22063	-6.40265	0.00000
O	-0.28075	-7.76192	0.00000
O	1.91932	5.43055	0.00000
O	0.16452	6.74189	0.00000
N	0.68091	5.59969	0.00000

X = CN E = -555.337096 a.u. ZPE = 0.175007 a.u. G = -555.203383 a.u.

C	-3.39624	-0.96289	0.00000
H	-3.03698	-1.99458	0.00000
C	-2.40828	0.03484	0.00000
H	-2.72957	1.07495	0.00000
C	-1.04328	-0.23670	0.00000
H	-0.75605	-1.29001	0.00000
C	0.00000	0.69080	0.00000
H	-0.25320	1.74986	0.00000
C	1.35351	0.33537	0.00000
H	1.57430	-0.73372	0.00000
C	2.44394	1.19420	0.00000
H	2.26075	2.26742	0.00000
C	3.78030	0.74940	0.00000
H	3.93352	-0.32924	0.00000
C	4.90810	1.53999	0.00000
H	4.81961	2.62194	0.00000
C	-4.76720	-0.79266	0.00000
H	-5.20399	0.20274	0.00000
C	-5.66144	-1.90625	0.00000
H	-5.14972	-2.89883	0.00000
O	-6.89551	-1.87023	0.00000
C	6.21599	1.02018	0.00000
N	7.30758	0.61336	0.00000

X = Cl E = -922.673788 a.u. ZPE = 0.166573 a.u. G = -922.548092 a.u

C	3.75429	0.33118	0.00000
H	3.76759	-0.76176	0.00000
C	2.49096	0.92835	0.00000
H	2.43442	2.01587	0.00000
C	1.29217	0.20371	0.00000
H	1.38836	-0.88444	0.00000
C	0.00000	0.70849	0.00000
H	-0.13334	1.78953	0.00000
C	-1.15783	-0.09943	0.00000
H	-0.98813	-1.17828	0.00000
C	-2.46815	0.32061	0.00000
H	-2.67661	1.38893	0.00000
C	-3.58035	-0.57771	0.00000
H	-3.34629	-1.64105	0.00000
C	-4.88305	-0.22756	0.00000

H	-5.26382	0.78302	0.00000
C	4.99557	0.95266	0.00000
H	5.07055	2.03748	0.00000
C	6.20729	0.20826	0.00000
H	6.05591	-0.89917	0.00000
O	7.36525	0.64979	0.00000
Cl	-6.18448	-1.43175	0.00000

X = H E = -463.044915 a.u. ZPE = 0.175202 a.u. G = -462.908116 a.u

C	-2.35047	0.84233	0.00000
H	-1.97731	1.86988	0.00000
C	-1.38023	-0.16063	0.00000
H	-1.71050	-1.19848	0.00000
C	0.00000	0.09384	0.00000
H	0.29283	1.14647	0.00000
C	1.03051	-0.83162	0.00000
H	0.77586	-1.89085	0.00000
C	2.40205	-0.48327	0.00000
H	2.62133	0.58702	0.00000
C	3.48075	-1.33369	0.00000
H	3.30158	-2.40832	0.00000
C	4.84313	-0.89197	0.00000
H	4.98165	0.19077	0.00000
C	5.95749	-1.66291	0.00000
H	5.89797	-2.74738	0.00000
C	-3.73371	0.70068	0.00000
H	-4.18665	-0.28804	0.00000
C	-4.60334	1.82360	0.00000
H	-4.06976	2.80609	0.00000
O	-5.84409	1.82244	0.00000
H	6.94872	-1.22484	0.00000

X = OH E = -538.287716 a.u. ZPE = 0.179559 a.u. G = -538.148726 a.u.

C	0.06271	3.16533	0.00000
H	1.15367	3.09159	0.00000
C	-0.62962	1.95671	0.00000
H	-1.71858	1.98407	-0.00000
C	0.00000	0.69792	0.00000
H	1.09277	0.71198	0.00000
C	-0.59852	-0.54738	-0.00000
H	-1.68685	-0.60021	-0.00000
C	0.12033	-1.77262	-0.00000
H	1.20927	-1.68283	0.00000
C	-0.39637	-3.04041	-0.00000
H	-1.47834	-3.16458	-0.00000
C	0.40379	-4.23720	-0.00000
H	1.48714	-4.09017	-0.00000
C	-0.04762	-5.50578	-0.00000
H	-1.09685	-5.77249	-0.00000

C	-0.45444	4.45919	0.00000
H	-1.52994	4.62181	0.00000
C	0.38423	5.60267	0.00000
H	1.47584	5.35887	0.00000
O	0.04455	6.79780	0.00000
O	0.74958	-6.64321	-0.00000
H	1.67192	-6.36540	-0.00000

X = NH2 E = -518.414474 a.u. ZPE = 0.192439 a.u. G = -518.262332 a.u.

C	-3.17611	-0.16203	-0.00106
H	-3.07487	-1.25082	0.00186
C	-1.98632	0.56040	-0.00728
H	-2.04200	1.64839	-0.01020
C	-0.70974	-0.03534	-0.00916
H	-0.69374	-1.12809	-0.00645
C	0.51758	0.59754	-0.01253
H	0.53875	1.68713	-0.01516
C	1.76506	-0.08404	-0.01029
H	1.70967	-1.17522	-0.00659
C	3.01554	0.47323	-0.01176
H	3.10112	1.55956	-0.02071
C	4.24062	-0.28060	0.00555
H	4.13445	-1.36805	-0.00383
C	5.49404	0.22251	0.02197
H	5.66225	1.29587	0.03064
C	-4.48375	0.32264	0.00198
H	-4.67216	1.39401	-0.00072
C	-5.60604	-0.54271	0.00923
H	-5.33594	-1.62819	0.01150
N	6.68844	-0.54186	-0.08540
H	7.39323	-0.27190	0.59274
H	6.51495	-1.53712	0.00054
O	-6.81002	-0.23277	0.01303

O	0.00000	0.00000	2.41152
C	0.00000	0.00000	-1.38623
N	0.00000	0.00000	-2.56303

X = Cl E = -611.679740 a.u. ZPE = 0.011735 a.u. G = -611.694911 a.u

C	1.18411	0.08794	0.00005
C	0.04417	0.66513	-0.00002
O	2.34746	-0.24935	-0.00002
Cl	-1.53820	-0.14844	-0.00000

X = H E = -152.059023 a.u. ZPE = 0.018782 a.u. G = -152.062925 a.u

C	-0.00512	0.02042	-0.00034
C	-1.25283	-0.10139	0.00008
O	1.21789	0.01117	0.00018
H	-2.19546	0.39646	0.00013

X = OH E = -227.272700 a.u. ZPE = 0.023974 a.u. G = -227.275162 a.u.

C	-0.51608	-0.46791	-0.00095
C	0.67962	-0.05354	0.00020
O	1.89280	0.14538	0.00016
O	-1.80319	0.11186	0.00082
H	-1.69816	1.07077	-0.00330

X = NH2 E = -207.408491 a.u. ZPE = 0.037077 a.u. G = -207.397747 a.u.

C	0.72601	-0.05460	-0.00259
C	-0.48474	-0.38133	0.05678
N	-1.79929	0.15668	-0.12872
H	-2.49198	-0.48267	0.24496
H	-1.92214	1.03269	0.38219
O	1.94519	0.12109	-0.00641

Y = O^{-}, R = polyyne, *n* = 1

X = NO2 E = -356.660203 a.u. ZPE = 0.023950 a.u. G = -356.665699 a.u.

C	-1.26882	0.74912	0.00000
C	0.00000	0.64416	0.00000
O	2.11428	-0.14838	0.00000
O	0.48544	-1.59811	0.00000
N	0.88532	-0.40368	0.00000
O	-2.42276	1.05475	0.00000

X = CNE = -607.730923 a.u. ZPE = 0.153795 a.u. G = -607.618619 a.u.

C	0.00000	0.00000	1.20373
C	0.00000	0.00000	-0.04266

Y = O^{-}, R = polyyne, *n* = 2

X = NO2 E = -432.847632 a.u. ZPE = 0.033736 a.u. G = -432.848077 a.u.

C	0.41679	-0.00042	0.00121
C	-0.81666	-0.00066	0.00223
C	1.73795	-0.00024	0.00092
C	2.99202	-0.00003	-0.00025
O	4.19028	0.00023	-0.00118
O	-2.77304	-1.09734	-0.00093
O	-2.77188	1.09808	-0.00093
N	-2.16336	0.00005	-0.00005

X = CNE = -320.573714 a.u. ZPE = 0.031024 a.u. G = -320.568300 a.u.

C	-0.09596	0.00259	-0.00003
C	-1.33594	0.00209	-0.00002
C	1.22623	0.00193	0.00003
C	2.47940	-0.00002	0.00006
O	3.67995	-0.00243	-0.00004
C	-2.68192	-0.00028	-0.00004
N	-3.85577	-0.00264	0.00003

X = Cl E = -687.878799 a.u. ZPE = 0.022092 a.u. G = -687.887126 a.u

C	-0.03753	0.13659	-0.00004
C	1.18130	0.33155	0.00002
C	-1.37433	0.05241	0.00013
C	-2.62004	-0.02866	0.00002
O	-3.82816	-0.11205	-0.00009
Cl	2.80758	-0.12088	-0.00000

X = H E = -228.263320 a.u. ZPE = 0.029663 a.u. G = -228.259687 a.u

C	0.00000	0.00000	-1.34610
C	0.00000	0.00000	-2.57446
C	0.00000	0.00000	-0.00044
C	0.00000	0.00000	1.24607
O	0.00000	0.00000	2.46044
H	0.00000	0.00000	-3.63390

X = OH E = -303.485406 a.u. ZPE = 0.035003 a.u. G = -303.479932 a.u.

C	-0.57762	0.05537	-0.00004
C	-1.79977	0.23748	0.00001
C	0.76815	0.01886	-0.00006
C	2.01474	-0.01267	0.00000
O	3.23098	-0.04742	0.00004
O	-3.07668	-0.24133	0.00002
H	-3.66747	0.51577	0.00009

X = NH2 E = -283.622273 a.u. ZPE = 0.047153 a.u. G = -283.605492 a.u.

C	0.55281	-0.00021	-0.01708
C	1.77650	-0.00016	-0.00399
C	-0.80147	-0.00041	-0.00352
C	-2.04629	0.00001	0.00268
N	3.15442	0.00023	-0.08769
H	3.58076	0.82464	0.32746
H	3.58125	-0.82380	0.32769
O	-3.26653	0.00027	0.01127

Y = O$^-$, R = polyyne, *n* = 3

X = NO2 E = -509.034385 a.u. ZPE = 0.044108 a.u. G = -509.027695 a.u.

C	-0.00217	-0.77383	0.00000
C	-0.00454	-2.00469	0.00000
C	-0.00001	0.54957	0.00000
C	0.00210	1.79797	0.00000
C	0.00443	3.10958	0.00000
C	0.00654	4.36738	0.00000
O	0.00861	5.56114	0.00000
O	-0.00454	-3.95486	1.09839
O	-0.00454	-3.95486	-1.09839
N	-0.00492	-3.35532	0.00000

X = CN E = -396.761366 a.u. ZPE = 0.041437 a.u. G = -396.746115 a.u.

C	-0.78329	-0.18110	1.12935
C	-1.48236	-0.34258	2.13744
C	-0.03531	-0.00835	0.05082
C	0.66977	0.15472	-0.96580
C	1.41212	0.32610	-2.03615
C	2.12221	0.49033	-3.05985
O	2.79793	0.64644	-4.03406
C	-2.24365	-0.51825	3.23503
N	-2.90577	-0.67095	4.18963

X = Cl E = -764.074425 a.u. ZPE = 0.033612 a.u. G = -764.072997 a.u

C	1.19234	0.04878	0.00012
C	2.41382	0.11253	-0.00156
C	-0.15167	0.03796	0.00074
C	-1.39201	0.01842	0.00168
C	-2.71856	-0.00338	0.00226
C	-3.97055	-0.01828	-0.00007
O	-5.17518	-0.03386	-0.00202
Cl	4.06831	-0.05325	-0.00017

X = H E = -304.460277 a.u. ZPE = 0.041043 a.u. G = -304.448028 a.u

C	0.00000	0.00000	-2.65352
C	0.00000	0.00000	-3.87770
C	0.00000	0.00000	-1.30740
C	0.00000	0.00000	-0.06742
C	0.00000	0.00000	1.26028
C	0.00000	0.00000	2.51204
O	0.00000	0.00000	3.71748
H	0.00000	0.00000	-4.93754

X = OH E = -379.685472 a.u. ZPE = 0.046016 a.u. G = -379.672029 a.u.

C	1.86211	-0.00681	-0.00000
C	3.07902	0.07135	0.00000
C	0.51004	-0.00984	0.00000
C	-0.72802	-0.00759	0.00000

C	-2.06059	-0.00408	-0.00000
C	-3.31098	0.00098	0.00000
O	-4.51983	0.00613	0.00000
O	4.40028	-0.12969	-0.00000
H	4.84682	0.72441	-0.00000

X = NH2 E = -359.824027 a.u. ZPE = 0.058392 a.u. G = -359.798447 a.u.

C	-1.84529	-0.00021	-0.02405
C	-3.06699	-0.00016	-0.00499
C	-0.49389	-0.00026	-0.02203
C	0.74467	-0.00020	-0.01374
C	2.07772	-0.00009	-0.00365
C	3.32799	0.00009	0.00815
N	-4.43331	0.00029	-0.06434
H	-4.86655	-0.82960	0.32784
H	-4.86595	0.83071	0.32740
O	4.53755	0.00022	0.01963

Y = O$^-$, R = polyyne, *n* = 4

X = NO2 E = -585.219249 a.u. ZPE = 0.053467 a.u. G = -585.206113 a.u.

C	-0.00039	-1.99109	0.00000
C	-0.00107	-3.21995	0.00000
C	-0.00082	-0.66645	0.00000
C	-0.00084	0.57996	0.00000
C	-0.00067	1.89359	0.00000
C	-0.00079	3.14653	0.00000
C	-0.00040	4.45329	0.00000
C	0.00021	5.71336	0.00000
O	0.00107	6.90408	0.00000
O	0.00107	-5.16690	1.09866
O	0.00107	-5.16690	-1.09866
N	0.00042	-4.57395	0.00000

X = CN E = -472.947063 a.u. ZPE = 0.050421 a.u. G = -472.928081 a.u.

C	2.67296	-0.00097	-0.00017
C	3.90800	-0.00050	-0.00019
C	1.34787	-0.00143	-0.00006
C	0.10204	-0.00039	0.00003
C	-1.21329	-0.00068	0.00026
C	-2.46500	-0.00009	0.00021
C	-3.77379	-0.00034	0.00016
C	-5.03266	0.00057	-0.00003
O	-6.22519	0.00101	-0.00022
C	5.25644	0.00058	-0.00004
N	6.42658	0.00164	0.00013

X = Cl E = -840.265591 a.u. ZPE = 0.042342 a.u. G = -840.258591 a.u

C	2.44475	0.02697	-0.00003
C	3.66461	0.06370	0.00166
C	1.10028	0.03541	-0.00046
C	-0.13755	0.02265	-0.00100
C	-1.46536	0.01395	-0.00121
C	-2.71181	0.00442	-0.00077
C	-4.03000	-0.00664	-0.00019
C	-5.28513	-0.01493	0.00041
O	-6.48454	-0.02322	0.00106
Cl	5.31751	-0.04044	0.00007

X = H E = -380.651970 a.u. ZPE = 0.050389 a.u. G = -380.633961 a.u

C	0.00000	0.00000	-3.94947
C	0.00000	0.00000	-5.17082
C	0.00000	0.00000	-2.60296
C	0.00000	0.00000	-1.36558
C	0.00000	0.00000	-0.03694
C	0.00000	0.00000	1.20924
C	0.00000	0.00000	2.52810
C	0.00000	0.00000	3.78304
O	0.00000	0.00000	4.98292
H	0.00000	0.00000	-6.23100

X = OH E = -455.879678 a.u. ZPE = 0.056138 a.u. G = -455.859128 a.u.

C	3.14685	-0.02251	0.00000
C	4.36149	0.03468	0.00000
C	1.79498	-0.02788	-0.00000
C	0.55966	-0.02523	-0.00000
C	-0.77344	-0.02149	-0.00000
C	-2.01798	-0.01071	-0.00000
C	-3.34029	0.00060	-0.00000
C	-4.59397	0.01320	-0.00000
O	-5.79641	0.02497	0.00000
O	5.68310	-0.08026	-0.00000
H	6.08264	0.79828	0.00000

X = NH2 E = -436.018924 a.u. ZPE = 0.067906 a.u. G = -435.987209 a.u.

C	3.13217	-0.00003	-0.02303
C	4.35260	0.00001	0.00047
C	1.78272	-0.00009	-0.02433
C	0.54623	-0.00006	-0.02173
C	-0.78663	-0.00011	-0.01580
C	-2.03137	-0.00005	-0.00747
C	-3.35416	0.00001	0.00192
C	-4.60776	0.00005	0.01199
N	5.71116	0.00009	-0.04947

H	6.15250	0.83462	0.32027
H	6.15259	-0.83431	0.32042
O	-5.81075	0.00008	0.02168

Y = O⁻, R = polyyne, *n* = 5

X = NO2 E = -661.402892 a.u. ZPE = 0.062967 a.u. G = -661.383711 a.u.

C	-5.77923	0.00243	-0.00015
C	-7.04068	0.00633	-0.00042
C	-4.47566	-0.00185	0.00012
C	-3.22052	-0.00768	0.00052
C	-1.91177	-0.00659	0.00041
C	-0.66086	-0.01349	0.00081
C	0.65390	-0.00741	0.00040
C	1.89813	-0.00695	0.00033
C	3.22410	-0.00264	0.00013
C	4.45107	0.00081	-0.00004
O	-8.22919	0.01009	-0.00061
O	6.39979	-1.09167	-0.00038
O	6.39319	1.10504	-0.00035
N	5.80840	0.00492	-0.00027

X = CN E = -549.131389 a.u. ZPE = 0.060574 a.u. G = -549.107646 a.u.

C	-5.04922	0.00319	0.00071
C	-6.30972	0.00878	-0.00032
C	-3.74386	-0.00238	0.00086
C	-2.48975	-0.00893	0.00198
C	-1.17912	-0.00974	0.00025
C	0.07080	-0.01823	0.00056
C	1.38734	-0.01059	-0.00071
C	2.63119	-0.00945	-0.00182
C	3.95747	-0.00269	-0.00120
C	5.19058	0.00332	-0.00078
O	-7.49971	0.01408	-0.00105
C	6.54007	0.00982	0.00034
N	7.70900	0.01553	0.00132

X = Cl E = -916.454007 a.u. ZPE = 0.052685 a.u. G = -916.439558 a.u

C	-5.33102	0.00608	-0.00006
C	-6.58836	0.01361	0.00066
C	-4.01828	-0.00040	-0.00039
C	-2.76832	-0.00695	-0.00062
C	-1.44870	-0.01132	-0.00134
C	-0.20436	-0.01965	-0.00124
C	1.12423	-0.02092	-0.00144
C	2.35994	-0.02947	-0.00021
C	3.70474	-0.01553	-0.00006
C	4.92327	-0.04153	0.00299
O	-7.78399	0.02053	0.00130
Cl	6.57371	0.03484	-0.00001

X = H E = -456.840565 a.u. ZPE = 0.060121 a.u. G = -456.813783 a.u

C	3.79899	-0.00129	-0.00011
C	5.05621	-0.00369	0.00003
C	2.48590	0.00174	-0.00021
C	1.23611	0.00585	0.00060
C	-0.08396	0.00468	-0.00097
C	-1.32801	0.01100	0.00081
C	-2.65728	0.00337	-0.00067
C	-3.89248	0.00067	-0.00014
C	-5.23950	-0.00414	-0.00004
C	-6.45889	-0.00812	0.00038
O	6.25210	-0.00606	0.00018
H	-7.51937	-0.01191	0.00042

X = OH E = -532.070018 a.u. ZPE = 0.065754 a.u. G = -532.043123 a.u.

C	-4.61840	0.00932	-0.00001
C	-5.87450	0.02390	-0.00000
C	-3.30266	-0.00559	0.00000
C	-2.05427	-0.02013	0.00000
C	-0.73101	-0.02854	0.00001
C	0.51138	-0.04095	0.00009
C	1.84464	-0.03762	0.00005
C	3.07795	-0.03673	0.00006
C	4.42919	-0.02310	0.00001
C	5.64239	0.03617	-0.00003
O	-7.07241	0.03780	-0.00006
O	6.96062	-0.04981	-0.00009
H	7.34605	0.83562	0.00005

X = NH2 E = -512.209807 a.u. ZPE = 0.077938 a.u. G = -512.171363 a.u.

C	-4.63057	0.00211	0.00587
C	-5.88654	0.00564	0.01515
C	-3.31423	-0.00205	-0.00261
C	-2.06588	-0.00700	-0.01076
C	-0.74229	-0.00668	-0.01659
C	0.50042	-0.01353	-0.02237
C	1.83313	-0.00649	-0.02485
C	3.06791	-0.00587	-0.02386
C	4.41620	-0.00019	-0.01906
C	5.63585	0.00478	0.00656
N	6.98885	0.00985	-0.03750
H	7.43371	0.85288	0.30572
H	7.44051	-0.82232	0.32290
O	-7.08502	0.00952	0.02362

Y = O⁻, R = acene, *n* = 1

X = NO2 E = -511.599850 a.u. ZPE = 0.093348 a.u. G = -511.539458 a.u.

C	1.42161	-1.22506	0.00012
C	0.05158	-1.22057	0.00014
C	-0.66426	0.00000	0.00005
C	0.05158	1.22057	-0.00008
C	1.42161	1.22506	-0.00010
C	2.20827	0.00000	0.00001
H	1.97239	-2.16038	0.00017
H	-0.51273	-2.14486	0.00023
H	-0.51273	2.14486	-0.00016
H	1.97239	2.16038	-0.00020
O	-2.68864	-1.09133	-0.00033
O	-2.68864	1.09133	0.00023
O	3.46169	0.00000	-0.00005
N	-2.07670	0.00000	0.00005

X = CN E = -399.292224 a.u. ZPE = 0.089474 a.u. G = -399.234382 a.u.

C	1.06183	1.21883	-0.00003
C	-0.31341	1.21243	0.00000
C	-1.04885	0.00002	-0.00002
C	-0.31342	-1.21242	0.00000
C	1.06181	-1.21883	-0.00003
C	1.85093	0.00000	-0.00019
H	1.60789	2.15760	0.00003
H	-0.85931	2.15220	0.00003
H	-0.85935	-2.15218	0.00003
H	1.60788	-2.15759	0.00003
O	3.10906	-0.00002	0.00015
C	-2.46213	0.00001	0.00001
N	-3.62716	-0.00001	0.00004

X = Cl E = -766.626974 a.u. ZPE = 0.080591 a.u. G = -766.577706 a.u

C	-1.17922	1.21150	0.00001
C	0.20875	1.20882	0.00005
C	0.90454	0.00001	-0.00007
C	0.20875	-1.20882	0.00004
C	-1.17920	-1.21150	0.00001
C	-1.97035	0.00001	-0.00044
H	-1.71739	2.15526	0.00025
H	0.75780	2.14592	0.00012
H	0.75784	-2.14590	0.00013
H	-1.71741	-2.15523	0.00025
O	-3.23703	-0.00002	0.00018
Cl	2.69740	-0.00000	0.00001

X = H E = -306.994057 a.u. ZPE = 0.089440 a.u. G = -306.933566 a.u

C	-0.28747	1.21169	0.00001
C	1.10022	1.20005	0.00007
C	1.82786	0.00000	-0.00007
C	1.10021	-1.20006	0.00007
C	-0.28746	-1.21169	0.00001
C	-1.07794	0.00001	-0.00040
H	-0.83081	2.15362	0.00025
H	1.63712	2.14846	0.00014
H	1.63713	-2.14846	0.00015
H	-0.83084	-2.15359	0.00023
O	-2.34732	-0.00001	0.00015
H	2.91348	-0.00001	0.00002

X = OH E = -382.235649 a.u. ZPE = 0.093245 a.u. G = -382.172656 a.u.

C	0.76338	1.20409	-0.00008
C	-0.62870	1.21810	-0.00012
C	-1.35163	0.02714	-0.00000
C	-0.65609	-1.18072	0.00009
C	0.73781	-1.20834	0.00014
C	1.54335	-0.01140	0.00017
H	1.31289	2.14183	-0.00021
H	-1.17082	2.16088	-0.00026
H	-1.21210	-2.12081	0.00013
H	1.26206	-2.16023	0.00018
O	2.81652	-0.02580	-0.00007
O	-2.76132	0.08860	-0.00006
H	-3.08244	-0.81724	0.00002

X = NH2 E = -362.361059 a.u. ZPE = 0.106222 a.u. G = -362.286224 a.u.

C	0.78486	1.20789	0.00012
C	-0.60417	1.21397	-0.00001
C	-1.35053	0.02861	-0.00006
C	-0.63357	-1.17892	0.00001
C	0.75416	-1.21010	0.00013
C	1.56208	-0.01000	0.00002
H	1.33621	2.14486	0.00016
H	-1.14639	2.15737	0.00002
H	-1.18831	-2.11856	0.00005
H	1.28220	-2.16048	0.00013
O	2.83193	-0.02949	-0.00014
N	-2.80426	0.09305	-0.00007
H	-3.19317	-0.37388	-0.81502
H	-3.19316	-0.37353	0.81509

Y = O⁻, R = acene, *n* = 2

X = NO2 E = -665.276405 a.u. ZPE = 0.139508 a.u. G = -665.173481 a.u.

C	-1.82693	0.09728	-0.00002
C	-0.86680	-0.91594	-0.00021
C	0.49118	-0.61204	-0.00012
C	0.92996	0.77065	0.00018
C	-0.09724	1.77772	0.00037
C	-1.42611	1.46157	0.00028
H	1.17998	-2.66872	-0.00050
H	-1.19671	-1.94756	-0.00043
C	1.50148	-1.62957	-0.00028
C	2.28511	1.07301	0.00027
H	0.20740	2.81985	0.00059
H	-2.19036	2.22675	0.00042
C	3.31305	0.06629	0.00004
C	2.82510	-1.31890	-0.00020
H	2.60983	2.10963	0.00051
H	3.58466	-2.09501	-0.00032
O	-3.54944	-1.43570	-0.00013
O	-4.05058	0.68743	-0.00023
O	4.54051	0.32145	0.00018
N	-3.21309	-0.23584	-0.00011

X = CNE = -552.970879 a.u. ZPE = 0.135749 a.u. G = -552.870393 a.u.

C	-2.23285	0.03612	0.00000
C	-1.24749	-0.95691	0.00000
C	0.11352	-0.64220	0.00000
C	0.53958	0.74233	0.00000
C	-0.49645	1.73632	0.00000
C	-1.82421	1.40483	0.00000
H	0.82591	-2.69004	-0.00000
H	-1.54682	-2.00099	-0.00000
C	1.13293	-1.64585	0.00000
C	1.89633	1.05992	0.00001
H	-0.20403	2.78223	0.00000
H	-2.58375	2.17926	-0.00000
C	2.93332	0.06886	0.00001
C	2.45524	-1.31879	0.00000
H	2.20598	2.10140	-0.00000
H	3.22138	-2.08889	0.00000
C	-3.60993	-0.30372	-0.00000
N	-4.74045	-0.57416	-0.00001
O	4.16307	0.33134	-0.00002

X = Cl E = -920.311957 a.u. ZPE = 0.127111 a.u. G = -920.219714 a.u

C	2.07430	0.04494	-0.00000
C	1.12886	-0.95552	-0.00002
C	-0.24544	-0.63667	-0.00003
C	-0.66976	0.74366	-0.00001
C	0.36288	1.73423	0.00001
C	1.69930	1.40378	0.00001
H	-0.96085	-2.68134	0.00005
H	1.43374	-1.99694	-0.00000
C	-1.26306	-1.63547	0.00002
C	-2.03434	1.06460	-0.00005
H	0.07470	2.78148	0.00003
H	2.46247	2.17340	0.00003
C	-3.07114	0.08043	-0.00019
C	-2.58837	-1.30343	-0.00001
H	-2.33674	2.10861	0.00003
H	-3.35269	-2.07608	0.00007
Cl	3.81037	-0.36879	0.00002
O	-4.30702	0.33963	0.00014

X = H E = -460.681499 a.u. ZPE = 0.136478 a.u. G = -460.577639 a.u

C	-2.89287	0.41551	0.00001
C	-1.80394	1.26929	0.00000
C	-0.47943	0.78511	-0.00002
C	-0.23473	-0.63813	-0.00001
C	-1.38576	-1.48985	0.00001
C	-2.66557	-0.98269	0.00001
H	0.49379	2.71995	0.00004
H	-1.95837	2.34653	0.00002
C	0.65965	1.64337	0.00001
C	1.07933	-1.13051	-0.00004
H	-1.22849	-2.56532	0.00003
H	-3.51367	-1.66273	0.00003
C	2.23330	-0.28826	-0.00015
C	1.93252	1.14603	-0.00001
H	1.24505	-2.20505	0.00002
H	2.78986	1.81447	0.00005
H	-3.90539	0.80657	0.00002
O	3.42778	-0.70420	0.00011

X = OH E = -535.926019 a.u. ZPE = 0.140189 a.u. G = -535.820259 a.u.

C	-2.49284	-0.10016	-0.00001
C	-1.49713	-1.05138	0.00002
C	-0.12967	-0.68309	0.00004
C	0.23681	0.71087	0.00003
C	-0.83581	1.65529	-0.00002
C	-2.15855	1.26950	-0.00003
H	0.67381	-2.69385	-0.00011
H	-1.75407	-2.11091	0.00000
C	0.92872	-1.63456	-0.00002
C	1.59140	1.09005	0.00007
H	-0.59031	2.71366	-0.00004
H	-2.95722	2.00409	-0.00006
C	2.66826	0.15405	0.00027

C	2.24166	-1.24632	0.00001
H	1.84771	2.14671	-0.00001
H	3.03666	-1.98790	-0.00009
O	3.89622	0.46450	-0.00020
O	-3.85233	-0.42380	-0.00003
H	-3.92479	-1.38287	-0.00003

X = NH2 E = -516.050833 a.u. ZPE = 0.153013 a.u. G = -515.932320 a.u.

C	2.49659	-0.10608	-0.01032
C	1.48616	-1.04726	-0.00471
C	0.11926	-0.68043	-0.01153
C	-0.25178	0.71142	-0.00449
C	0.82109	1.65513	-0.00821
C	2.14175	1.26569	-0.02077
H	-0.68142	-2.69245	-0.01413
H	1.73712	-2.10757	0.00544
C	-0.93832	-1.63367	-0.00878
C	-1.60673	1.08920	0.00440
H	0.57639	2.71400	-0.01007
H	2.92969	2.01485	-0.04732
C	-2.68153	0.15090	0.00802
C	-2.25229	-1.24831	-0.00004
H	-1.86434	2.14560	0.00966
H	-3.04580	-1.99151	0.00164
N	3.88281	-0.47158	-0.06787
H	4.43807	0.05915	0.59513
H	4.01079	-1.45681	0.13223
O	-3.91067	0.45954	0.01764

Y = O⁻, R = acene, *n* = 3

X = NO2 E = -818.948959 a.u. ZPE = 0.185551 a.u. G = -818.803582 a.u.

C	-3.03001	0.05057	0.00008
C	-1.97143	-0.84638	0.00007
C	-0.64719	-0.38831	0.00006
C	-0.37749	1.03867	0.00006
C	-1.51105	1.92378	0.00008
C	-2.79229	1.45729	0.00011
C	0.45634	-1.27618	0.00004
C	0.93286	1.48689	0.00004
C	2.04849	0.60272	0.00001
C	1.76227	-0.82888	0.00001
C	2.88088	-1.73539	-0.00001
H	2.67143	-2.80276	0.00001
C	4.15814	-1.28330	-0.00004
C	4.49366	0.15298	-0.00009
C	3.37073	1.04305	-0.00002
H	0.26227	-2.34600	0.00006
H	-2.18255	-1.90832	0.00008
H	-1.32745	2.99376	0.00007
H	-3.64144	2.12638	0.00013
H	1.12457	2.55635	0.00004
H	4.99982	-1.96934	-0.00004
H	3.58383	2.10780	0.00000
O	5.69059	0.52460	-0.00007
O	-5.30657	0.38509	-0.00039
O	-4.57193	-1.66762	0.00003
N	-4.37582	-0.43992	0.00010

X = CN E = -706.644686 a.u. ZPE = 0.181828 a.u. G = -706.501698 a.u.

C	-3.44805	-0.05856	-0.00001
C	-2.36209	-0.92722	0.00003
C	-1.03826	-0.45126	0.00003
C	-0.79098	0.97841	0.00002
C	-1.93647	1.84430	-0.00001
C	-3.21126	1.35616	-0.00002
C	0.07718	-1.31809	0.00003
C	0.51493	1.44980	0.00004
C	1.64384	0.58713	0.00003
C	1.37836	-0.84830	0.00001
C	2.51046	-1.73527	0.00001
H	2.31994	-2.80653	0.00001
C	3.78080	-1.26050	-0.00002
C	4.09735	0.18021	-0.00007
C	2.96289	1.04892	0.00001
H	-0.09606	-2.39193	0.00004
H	-2.53546	-1.99880	0.00005
H	-1.77040	2.91739	-0.00001
H	-4.06095	2.02948	-0.00005
H	0.68611	2.52294	0.00004
H	4.63283	-1.93425	-0.00002
H	3.15608	2.11771	0.00006
O	5.29327	0.56457	-0.00003
C	-4.77811	-0.55857	-0.00003
N	-5.86884	-0.95649	-0.00003

X = Cl E = -1073.989131 a.u. ZPE = 0.173323 a.u. G = -1073.854212 a.u

C	3.28087	-0.03386	0.00001
C	2.23573	-0.91846	-0.00004
C	0.89681	-0.44333	-0.00006
C	0.64724	0.98249	-0.00005
C	1.78562	1.85102	0.00000
C	3.06892	1.36803	0.00003
C	-0.21227	-1.30930	-0.00006
C	-0.66515	1.45392	-0.00004
C	-1.78947	0.59246	-0.00003
C	-1.51910	-0.84020	-0.00007
C	-2.64862	-1.72672	0.00004

H	-2.45856	-2.79839	0.00012
C	-3.92124	-1.25202	0.00003
C	-4.24568	0.18616	-0.00043
C	-3.11530	1.05325	-0.00008
H	-0.03970	-2.38345	-0.00006
H	2.41971	-1.98723	-0.00005
H	1.61840	2.92409	0.00000
H	3.91943	2.03905	0.00005
H	-0.83524	2.52745	-0.00003
H	-4.77084	-1.92955	0.00021
H	-3.30649	2.12274	0.00014
O	-5.44808	0.56305	0.00036
Cl	4.95575	-0.63527	0.00006

X = H E = -614.360302 a.u. ZPE = 0.182800 a.u. G = -614.213718 a.u

C	-4.06419	0.53358	0.00005
C	-2.91969	1.29682	0.00001
C	-1.62881	0.70414	-0.00002
C	-1.51631	-0.73903	-0.00003
C	-2.73253	-1.49690	0.00001
C	-3.96027	-0.88603	0.00006
C	-0.44107	1.45912	-0.00003
C	-0.25312	-1.33220	-0.00003
C	0.94784	-0.58195	-0.00003
C	0.81617	0.86938	-0.00005
C	2.02549	1.64372	0.00004
H	1.93855	2.72886	0.00013
C	3.24745	1.05001	0.00002
C	3.43310	-0.41227	-0.00042
C	2.22559	-1.16677	-0.00009
H	-0.51126	2.54528	-0.00000
H	-2.98792	2.38200	0.00002
H	-2.66269	-2.58127	0.00001
H	-4.86445	-1.48794	0.00007
H	-0.18509	-2.41730	-0.00003
H	4.15775	1.64376	0.00018
H	2.31399	-2.24978	0.00012
O	4.59560	-0.90228	0.00031
H	-5.04150	1.00502	0.00009

X = OH E = -689.606075 a.u. ZPE = 0.186503 a.u. G = -689.457408 a.u.

C	3.68238	-0.23110	-0.00002
C	2.59677	-1.06745	-0.00007
C	1.26849	-0.54926	-0.00002
C	1.07279	0.88277	-0.00001
C	2.24258	1.70738	-0.00002
C	3.50640	1.17738	0.00001
C	0.12820	-1.36878	-0.00001
C	-0.22280	1.40608	-0.00000
C	-1.37866	0.59098	0.00001
C	-1.16315	-0.84898	0.00000
C	-2.32585	-1.68893	-0.00001
H	-2.17929	-2.76771	-0.00004
C	-3.58035	-1.16386	0.00001
C	-3.85134	0.28416	0.00009
C	-2.68911	1.10442	0.00003
H	0.25648	-2.44958	-0.00003
H	2.73659	-2.14736	-0.00013
H	2.11302	2.78579	0.00001
H	4.38634	1.81109	0.00005
H	-0.34934	2.48583	0.00000
H	-4.45481	-1.80930	-0.00002
H	-2.83649	2.18098	0.00001
O	-5.04166	0.70614	-0.00004
O	4.99553	-0.68731	-0.00004
H	4.97852	-1.64930	0.00088

X = NH2 E = -669.731469 a.u. ZPE = 0.199263 a.u. G = -669.570185 a.u.

C	3.68877	-0.23653	-0.00484
C	2.58932	-1.06141	-0.00328
C	1.26157	-0.54482	-0.01200
C	1.06064	0.88594	-0.00720
C	2.23009	1.70953	-0.00710
C	3.49211	1.17617	-0.01370
C	0.12161	-1.36573	-0.01170
C	-0.23563	1.40788	-0.00221
C	-1.38983	0.59085	-0.00094
C	-1.17100	-0.84845	-0.00599
C	-2.33230	-1.69040	-0.00431
H	-2.18365	-2.76893	-0.00836
C	-3.58807	-1.16790	0.00248
C	-3.86230	0.27934	0.00825
C	-2.70187	1.10163	0.00572
H	0.25174	-2.44633	-0.01509
H	2.72305	-2.14159	0.00478
H	2.10224	2.78840	-0.00887
H	4.36126	1.82845	-0.03483
H	-0.36368	2.48760	0.00129
H	-4.46108	-1.81544	0.00391
H	-2.85125	2.17801	0.00939
N	5.02095	-0.73995	-0.06308
H	5.65747	-0.21907	0.52888
H	5.07029	-1.72107	0.18223
O	-5.05397	0.69913	0.01489

Y = O^-, R = acene, *n* = 4

X = NO2 E = -972.619459 a.u. ZPE = 0.231691 a.u. G = -972.431490 a.u.

C	-0.78497	0.84402	0.00001
C	-0.59546	-0.60265	0.00000
C	0.68982	-1.13090	-0.00000
C	-1.75407	-1.42927	0.00000
C	-2.07763	1.35865	0.00002
C	0.38056	1.66064	0.00001
C	1.83648	-0.31224	-0.00000
C	-3.02770	-0.91105	0.00001
C	-3.23493	0.53855	0.00001
C	1.65838	1.13127	0.00000
C	3.13582	-0.85136	-0.00001
C	-4.19483	-1.76038	0.00001
C	-4.53453	1.04494	0.00001
C	2.84505	1.94517	0.00000
C	4.24146	-0.02100	-0.00001
C	-5.44362	-1.24187	0.00001
C	-5.69991	0.21408	-0.00001
C	4.09330	1.40013	-0.00000
H	0.81823	-2.21008	-0.00001
H	-1.61931	-2.50817	0.00000
H	-2.21178	2.43662	0.00002
H	0.25495	2.73980	0.00001
H	3.28208	-1.92405	-0.00002
H	-4.03996	-2.83685	0.00002
H	-4.69365	2.11888	0.00001
H	2.72799	3.02428	0.00001
H	-6.32214	-1.87986	0.00002
H	4.98323	2.01361	-0.00000
O	-6.87636	0.64326	-0.00004
O	6.53481	0.17612	0.00000
O	5.67948	-1.82936	0.00000
N	5.55965	-0.59351	-0.00002

X = CN E = -860.316034 a.u. ZPE = 0.227896 a.u. G = -860.130570 a.u.

C	-0.36477	0.80281	0.00001
C	-0.19779	-0.64666	0.00001
C	1.08187	-1.19635	0.00001
C	-1.36848	-1.45248	0.00001
C	-1.65164	1.33922	0.00001
C	0.81280	1.59814	0.00000
C	2.23891	-0.39882	0.00000
C	-2.63483	-0.91248	0.00001
C	-2.82085	0.54025	0.00001
C	2.08412	1.04624	0.00000
C	3.53676	-0.95530	-0.00000
C	-3.81452	-1.74293	0.00001
C	-4.11594	1.06654	0.00001
C	3.28124	1.84099	0.00001
C	4.66822	-0.15445	-0.00000
C	-5.05506	-1.20333	0.00001
C	-5.29145	0.25640	0.00000
C	4.52140	1.27529	0.00000
H	1.19013	-2.27809	0.00000
H	-1.25291	-2.53381	0.00001
H	-1.76654	2.41954	0.00001
H	0.70700	2.67954	0.00000
H	3.64503	-2.03504	-0.00001
H	-3.67727	-2.82203	0.00001
H	-4.25668	2.14318	0.00000
H	3.18115	2.92200	0.00002
H	-5.94325	-1.82821	0.00001
H	5.41206	1.89281	0.00000
O	-6.46592	0.69811	-0.00004
C	5.96728	-0.73374	-0.00001
N	7.03214	-1.19442	-0.00002

X = Cl E = -1227.662566 a.u. ZPE = 0.219483 a.u. G = -1227.485054 a.u

C	-0.51771	0.80839	0.00005
C	-0.34575	-0.63888	0.00002
C	0.94148	-1.18539	-0.00001
C	-1.51217	-1.44510	0.00002
C	-1.81051	1.34373	0.00007
C	0.65360	1.60555	0.00004
C	2.09015	-0.38625	-0.00001
C	-2.78257	-0.90712	0.00005
C	-2.97499	0.54433	0.00004
C	1.93107	1.05552	0.00001
C	3.40422	-0.93979	-0.00002
C	-3.95923	-1.73904	0.00004
C	-4.27622	1.06758	0.00004
C	3.11995	1.85596	-0.00001
C	4.49572	-0.11923	-0.00003
C	-5.20179	-1.20072	0.00002
C	-5.44741	0.25729	0.00004
C	4.36872	1.29729	-0.00002
H	1.05133	-2.26706	-0.00003
H	-1.39590	-2.52656	0.00001
H	-1.92526	2.42427	0.00008
H	0.54615	2.68694	0.00006
H	3.52506	-2.01713	-0.00003
H	-3.82108	-2.81823	0.00005
H	-4.41602	2.14469	-0.00001
H	3.01613	2.93674	-0.00001
H	-6.08705	-1.83053	-0.00001
H	5.25906	1.91381	-0.00005
O	-6.62830	0.69003	-0.00021
Cl	6.13027	-0.81247	-0.00002

X = H E = -768.034924 a.u. ZPE = 0.228961 a.u. G = -767.845744 a.u

C	-0.30672	-0.68378	0.00004
C	-0.37852	0.77143	0.00001
C	-1.62677	1.40258	-0.00003
C	0.84029	1.49621	0.00002
C	0.94799	-1.30575	0.00007
C	-1.52917	-1.39946	0.00004
C	-2.82799	0.68469	-0.00002
C	2.07150	0.87307	0.00005
C	2.16360	-0.58788	0.00004
C	-2.76769	-0.76494	0.00000
C	-4.10502	1.31885	-0.00003
C	3.30269	1.62176	0.00004
C	3.42749	-1.19912	0.00005
C	-4.00842	-1.48467	-0.00001
C	-5.26753	0.59147	-0.00004
C	4.50567	0.99928	0.00003
C	4.65097	-0.47181	0.00004
C	-5.21246	-0.83463	-0.00003
H	-1.66348	2.48963	-0.00005
H	0.79775	2.58326	0.00000
H	0.98900	-2.39171	0.00009
H	-1.49451	-2.48587	0.00006
H	-4.13947	2.40503	-0.00006
H	3.23912	2.70804	0.00006
H	3.49269	-2.28340	-0.00000
H	-3.97247	-2.57036	-0.00000
H	5.43195	1.56722	0.00001
H	-6.13689	-1.40390	-0.00004
O	5.80083	-0.98430	-0.00020
H	-6.22983	1.09275	-0.00003

X = OH E = -843.281365 a.u. ZPE = 0.232662 a.u. G = -843.090067 a.u.

C	-0.11127	0.75299	-0.00000
C	0.01764	-0.69733	-0.00001
C	1.29186	-1.28201	-0.00001
C	-1.17203	-1.46580	-0.00001
C	-1.38933	1.32914	0.00000
C	1.08222	1.51309	0.00000
C	2.46249	-0.52029	-0.00001
C	-2.42728	-0.88870	-0.00001
C	-2.57649	0.56712	-0.00000
C	2.34422	0.92418	0.00000
C	3.76804	-1.10389	-0.00001
C	-3.62864	-1.68307	0.00001
C	-3.86404	1.13051	-0.00001
C	3.55445	1.69114	0.00001
C	4.88983	-0.32191	-0.00000
C	-4.85496	-1.10637	0.00001
C	-5.05763	0.35771	-0.00005
C	4.78618	1.09849	0.00000
H	1.36700	-2.36705	-0.00001
H	-1.09022	-2.55064	-0.00001
H	-1.46964	2.41295	0.00001
H	1.00892	2.59755	0.00001
H	3.85467	-2.18875	-0.00001
H	-3.52408	-2.76629	0.00003
H	-3.97052	2.21152	0.00001
H	3.47829	2.77433	0.00001
H	-5.75895	-1.70915	0.00003
H	5.69815	1.68447	0.00001
O	-6.22797	0.82465	0.00006
O	6.17696	-0.83392	-0.00001
H	6.12285	-1.79481	-0.00001

X = NH2 E = -823.407238 a.u. ZPE = 0.245459 a.u. G = -823.203282 a.u.

C	-0.12023	0.75458	-0.00531
C	0.01103	-0.69519	-0.00940
C	1.28665	-1.27797	-0.01307
C	-1.17762	-1.46501	-0.00860
C	-1.39953	1.32906	-0.00056
C	1.07216	1.51632	-0.00516
C	2.45729	-0.51536	-0.01153
C	-2.43409	-0.88966	-0.00359
C	-2.58535	0.56550	0.00090
C	2.33431	0.92815	-0.00754
C	3.76215	-1.09775	-0.00100
C	-3.63435	-1.68533	-0.00192
C	-3.87441	1.12736	0.00669
C	3.54463	1.69372	-0.00576
C	4.89766	-0.32826	-0.00023
C	-4.86165	-1.11014	0.00402
C	-5.06663	0.35326	0.00872
C	4.77410	1.09781	-0.00918
H	1.36333	-2.36293	-0.01585
H	-1.09438	-2.54977	-0.01171
H	-1.48136	2.41283	0.00246
H	0.99739	2.60082	-0.00241
H	3.84255	-2.18255	0.00620
H	-3.52853	-2.76848	-0.00541
H	-3.98183	2.20829	0.01010
H	3.47015	2.77730	-0.00791
H	-5.76468	-1.71443	0.00547
H	5.67623	1.70303	-0.02759
N	6.19954	-0.89032	-0.05687
H	6.87632	-0.37849	0.49550
H	6.21763	-1.86817	0.20264
O	-6.23829	0.81855	0.01520

Monoderivatives (R-X)

R = polyene, n = 1

X = NO2 E = -283.179125 a.u. ZPE = 0.054604 a.u. G = -283.152373 a.u.

C	-0.60038	-0.78187	0.00000
H	-1.67938	-0.73888	0.00000
C	0.15769	-1.86632	0.00000
H	1.23765	-1.79068	0.00000
O	1.21824	0.66864	0.00000
O	-0.79342	1.49726	0.00000
N	0.00000	0.56265	0.00000
H	-0.30067	-2.84706	0.00000

X = CNE = -170.882900 a.u. ZPE = 0.050593 a.u. G = -170.858180 a.u.

C	1.31776	0.98491	0.00000
H	2.02905	0.16789	0.00000
C	0.00000	0.77010	0.00000
H	-0.70462	1.59538	0.00000
C	-0.57735	-0.53567	0.00000
N	-1.06831	-1.58187	0.00000
H	1.71128	1.99375	0.00000

X = Cl E = -538.240071 a.u. ZPE = 0.042468 a.u. G = -538.223055 a.u.

C	1.29215	1.05266	0.00000
H	2.05791	0.28720	0.00000
C	0.00000	0.76520	0.00000
H	-0.78934	1.50480	0.00000
Cl	-0.62503	-0.86998	0.00000
H	1.60409	2.09049	0.00000

X = H (unsubstituted R) E = -78.615538 a.u. ZPE = 0.050775 a.u. G = -78.586943 a.u.

C	0.00001	0.66441	0.00000
H	0.92304	1.23497	0.00000
C	0.00001	-0.66447	0.00000
H	-0.92308	-1.23480	0.00000
H	0.92257	-1.23545	0.00000
H	-0.92267	1.23563	0.00000

X = NH2 E = -133.995178 a.u. ZPE = 0.068589 a.u. G = -133.951250 a.u.

C	-1.25228	-0.19657	0.01459
H	-1.33367	-1.27853	0.01952
C	-0.06946	0.42827	-0.00066
H	-0.02938	1.51380	-0.01084
N	1.18480	-0.16944	-0.07971
H	1.94914	0.34395	0.33192
H	1.22123	-1.15710	0.12963
H	-2.17046	0.37377	0.00420

X = OH E = -153.865660 a.u. ZPE = 0.056337 a.u. G = -153.833777 a.u.

C	-0.03755	0.44038	0.00001
H	0.03150	1.52195	-0.00000
C	-1.20131	-0.20638	-0.00001
H	-1.26373	-1.28991	-0.00002
O	1.20729	-0.11385	-0.00001
H	1.13491	-1.07580	0.00006
H	-2.12781	0.35055	0.00003

X = O^- E = -153.289944 a.u. ZPE = 0.041501 a.u. G = -153.272668 a.u.

C	0.00000	0.36675	0.00000
H	0.29089	1.45579	0.00000
C	1.09375	-0.48096	0.00000
H	0.97231	-1.56210	0.00000
O	-1.24063	0.10759	0.00000
H	2.09932	-0.06916	0.00000

R = polyene, n = 2

X = NO2 E = -360.606429 a.u. ZPE = 0.088053 a.u. G = -360.549856 a.u.

C	2.76627	-1.74581	0.00000
H	2.23476	-2.69177	0.00000
C	2.11292	-0.57628	0.00000
H	2.67289	0.35496	0.00000
C	0.66742	-0.48572	0.00000
H	0.09026	-1.40557	0.00000
C	0.00000	0.67378	0.00000
H	0.43098	1.66409	0.00000
O	-2.07778	-0.35326	0.00000
O	-1.96817	1.82113	0.00000
N	-1.45564	0.70415	0.00000
H	3.84851	-1.78960	0.00000

X = CN E = -248.310398 a.u. ZPE = 0.084094 a.u. G = -248.255872 a.u.

C	-0.23620	-0.88615	0.00000
H	-1.25124	-1.27144	0.00000
C	0.00000	0.44083	0.00000
H	1.02779	0.79381	0.00000
C	-1.04580	1.44345	0.00000
H	-2.07213	1.08440	0.00000
C	-0.80396	2.76102	0.00000
H	0.20857	3.15137	0.00000
C	0.80105	-1.85718	0.00000
N	1.62940	-2.66501	0.00000

H	-1.60932	3.48514	0.00000

X = Cl E = -615.664630 a.u.   ZPE = 0.075985 a.u.   G = -615.617803 a.u.

C	-0.27570	-0.74466	0.00000
H	-1.27801	-1.15231	0.00000
C	0.00000	0.56176	0.00000
H	1.03533	0.89026	0.00000
C	-1.03427	1.58351	0.00000
H	-2.06474	1.23562	0.00000
C	-0.78630	2.89905	0.00000
H	0.22760	3.28598	0.00000
Cl	0.95569	-1.98137	0.00000
H	-1.58929	3.62573	0.00000

X = H (unsubstituted R)   E = -156.040829 a.u.   ZPE = 0.084736 a.u.   G = -155.982545 a.u.

C	1.10495	-1.48317	0.00000
H	2.09514	-1.03814	0.00000
C	-0.00010	-0.72816	0.00000
H	-0.97556	-1.21074	0.00000
C	0.00000	0.72800	0.00000
H	0.97550	1.21060	0.00000
C	-1.10485	1.48326	0.00000
H	-2.09525	1.03850	0.00000
H	1.05421	-2.56530	0.00000
H	-1.05410	2.56554	0.00000

X = NH2   E = -211.420696 a.u.   ZPE = 0.102075 a.u.   G = -211.347061 a.u.

C	1.14351	0.37838	0.00131
H	1.14979	1.46489	-0.00380
C	-0.03037	-0.28426	0.00735
H	-0.02939	-1.37294	0.01007
C	-1.31428	0.38388	0.00013
H	-1.28387	1.47286	-0.00673
C	-2.51213	-0.22134	0.00303
H	-2.60319	-1.30301	0.00735
N	2.40492	-0.18905	-0.07259
H	3.16976	0.34946	0.30307
H	2.47515	-1.17632	0.12731
H	-3.43303	0.34846	-0.00015

X = OH   E = -231.289630 a.u.   ZPE = 0.089887 a.u.   G = -231.228010 a.u.

C	-1.16389	-0.39470	0.00000
H	-1.21591	-1.47756	0.00000
C	0.00000	0.27204	0.00000
H	-0.00741	1.36134	0.00000
C	1.29050	-0.39082	0.00000
H	1.26936	-1.47912	0.00000
C	2.47735	0.23092	0.00000
H	2.55341	1.31366	0.00000
O	-2.41008	0.14888	0.00000
H	-2.34870	1.11167	0.00000
H	3.40617	-0.32565	0.00000

X = O⁻ E = -230.734470 a.u.   ZPE = 0.075253 a.u.   G = -230.687166 a.u.

C	2.45413	-0.22182	0.00000
H	2.81973	0.80192	0.00000
C	1.11800	-0.50813	0.00000
H	0.85477	-1.57074	0.00000
C	0.00000	0.36708	0.00000
H	0.15884	1.44464	0.00000
C	-1.31970	-0.10380	0.00000
H	-1.38828	-1.22451	0.00000
O	-2.39493	0.54482	0.00000
H	3.19983	-1.00989	0.00000

R = polyene, n = 3

X = NO2   E = -438.034866 a.u.   ZPE = 0.121418 a.u.   G = -437.948395 a.u.

C	1.71519	-4.27259	0.00000
H	2.74275	-3.92341	0.00000
C	0.68404	-3.41484	0.00000
H	-0.33283	-3.80014	0.00000
C	0.83783	-1.97725	0.00000
H	1.85563	-1.59184	0.00000
C	-0.19217	-1.09946	0.00000
H	-1.21257	-1.47481	0.00000
C	0.00000	0.32535	0.00000
H	1.01447	0.71277	0.00000
C	-1.00587	1.21429	0.00000
H	-2.06172	0.98807	0.00000
O	0.41541	3.04217	0.00000
O	-1.73790	3.36935	0.00000
N	-0.74589	2.64127	0.00000
H	1.56131	-5.34465	0.00000

X = CN   E = -325.738279 a.u.   ZPE = 0.117474 a.u.   G = -325.653900 a.u.

C	-1.76687	1.04775	0.00000
H	-2.54911	0.29500	0.00000
C	-0.46260	0.69272	0.00000
H	0.29181	1.47507	0.00000
C	0.00000	-0.67004	0.00000
H	-0.75446	-1.45390	0.00000
C	1.30861	-1.01193	0.00000
H	2.05809	-0.22245	0.00000

C	1.79646	-2.37367	0.00000
H	1.04693	-3.16188	0.00000
C	3.09672	-2.70261	0.00000
H	3.87324	-1.94438	0.00000
C	-2.19958	2.39917	0.00000
N	-2.57476	3.49433	0.00000
H	3.42042	-3.73613	0.00000

X = Cl E = -693.091633 a.u. ZPE = 0.109323 a.u. G = -693.015020 a.u.

C	-0.98872	-1.57953	0.00000
H	-2.04066	-1.32671	0.00000
C	0.00000	-0.67801	0.00000
H	1.03060	-1.02145	0.00000
C	-0.23482	0.74874	0.00000
H	-1.27106	1.08119	0.00000
C	0.74836	1.67436	0.00000
H	1.78527	1.34267	0.00000
C	0.51995	3.10429	0.00000
H	-0.51854	3.42970	0.00000
C	1.49271	4.02734	0.00000
H	2.54112	3.74638	0.00000
Cl	-0.70710	-3.30129	0.00000
H	1.26896	5.08703	0.00000

X = H (unsubstituted R) E = -233.467699 a.u. ZPE = 0.118101 a.u. G = -233.379721 a.u.

C	-1.20198	-2.83228	0.00000
H	-0.27843	-3.40265	0.00000
C	-1.19701	-1.49129	0.00000
H	-2.14622	-0.95899	0.00000
C	-0.00025	-0.67482	0.00000
H	0.95256	-1.20186	0.00000
C	0.00000	0.67481	0.00000
H	-0.95277	1.20199	0.00000
C	1.19674	1.49134	0.00000
H	2.14582	0.95862	0.00000
C	1.20237	2.83224	0.00000
H	0.27909	3.40321	0.00000
H	-2.12663	-3.39642	0.00000
H	2.12736	3.39608	0.00000

X = NH2 E = -288.848029 a.u. ZPE = 0.135408 a.u. G = -288.744620 a.u.

C	2.36818	0.36886	0.00251
H	2.35945	1.45515	0.00000
C	1.20136	-0.31239	0.00612
H	1.22241	-1.40082	0.00645
C	-0.08640	0.33067	-0.00006
H	-0.08366	1.42062	-0.00587
C	-1.28281	-0.30213	0.00240
H	-1.30589	-1.39066	0.00575
C	-2.55794	0.38031	-0.00037
H	-2.51851	1.46877	-0.00430
C	-3.75919	-0.22012	0.00110
H	-3.85277	-1.30162	0.00467
N	3.63408	-0.18206	-0.06950
H	4.39821	0.36915	0.28803
H	3.72198	-1.16915	0.12311
H	-4.67903	0.35179	-0.00148

X = OH E = -308.716470 a.u. ZPE = 0.123200 a.u. G = -308.625126 a.u.

C	2.38268	0.38401	-0.00029
H	2.42713	1.46705	-0.00048
C	1.22192	-0.29329	-0.00007
H	1.24260	-1.38246	0.00011
C	-0.06958	0.35165	-0.00006
H	-0.06978	1.44070	-0.00024
C	-1.25780	-0.29122	0.00017
H	-1.27129	-1.37992	0.00035
C	-2.54131	0.37847	0.00018
H	-2.51604	1.46694	-0.00001
C	-3.73122	-0.24146	0.00040
H	-3.80679	-1.32431	0.00059
O	3.63126	-0.15204	-0.00031
H	3.57657	-1.11531	-0.00015
H	-4.66057	0.31471	0.00039

X = O^{-} E = -308.174031 a.u. ZPE = 0.108605 a.u. G = -308.096928 a.u.

C	3.62409	-0.79874	0.00000
H	3.63801	-1.88524	0.00000
C	2.45569	-0.10063	0.00000
H	2.53511	0.98963	0.00000
C	1.12066	-0.60333	0.00000
H	0.98733	-1.68506	0.00000
C	0.00000	0.20566	0.00000
H	0.19055	1.28348	0.00000
C	-1.35621	-0.15933	0.00000
H	-1.63813	-1.21055	0.00000
C	-2.39059	0.79902	0.00000
H	-2.01832	1.85586	0.00000
O	-3.62504	0.61130	0.00000
H	4.58386	-0.29448	0.00000

R = polyene, n = 4

X = NO2 E = -515.463245 a.u. ZPE = 0.154684 a.u. G = -515.346997 a.u.

C	-2.31090	1.40380	0.00000

H	-2.08186	2.45898	0.00000
C	-1.42169	0.39526	0.00000
H	-1.81262	-0.61793	0.00000
C	0.00000	0.58191	0.00000
H	0.38067	1.60030	0.00000
C	0.87643	-0.45529	0.00000
H	0.48082	-1.46930	0.00000
O	-4.13945	-0.01456	0.00000
O	-4.46361	2.13977	0.00000
N	-3.73537	1.14672	0.00000
C	2.30452	-0.31438	0.00000
H	2.70648	0.69672	0.00000
C	3.16220	-1.36329	0.00000
H	2.75674	-2.37351	0.00000
C	4.60174	-1.23869	0.00000
H	5.00533	-0.22868	0.00000
C	5.44414	-2.28331	0.00000
H	5.07935	-3.30547	0.00000
H	6.51840	-2.14584	0.00000

X = CN E = -403.166297 a.u. ZPE = 0.150719 a.u. G = -403.052274 a.u.

C	-1.94494	-4.97841	0.00000
H	-0.96633	-5.44763	0.00000
C	-2.08179	-3.64349	0.00000
H	-3.08041	-3.21181	0.00000
C	-0.97404	-2.71485	0.00000
H	0.02661	-3.14363	0.00000
C	-1.10767	-1.36711	0.00000
H	-2.10758	-0.93743	0.00000
C	0.00000	-0.45262	0.00000
H	0.99785	-0.88795	0.00000
C	-0.11741	0.89899	0.00000
H	-1.10927	1.34581	0.00000
C	1.00972	1.78868	0.00000
H	1.99926	1.33892	0.00000
C	0.91185	3.13855	0.00000
H	-0.05775	3.62660	0.00000
C	2.04298	3.99401	0.00000
N	2.95289	4.71021	0.00000
H	-2.80481	-5.63686	0.00000

X = Cl E = -770.519241 a.u. ZPE = 0.142679 a.u. G = -770.412722 a.u.

C	-1.62896	5.32101	0.00000
H	-0.62272	5.72783	0.00000
C	-1.84941	3.99724	0.00000
H	-2.87463	3.63215	0.00000
C	-0.80774	2.99392	0.00000
H	0.22015	3.35268	0.00000
C	-1.03499	1.65978	0.00000
H	-2.06423	1.30453	0.00000
C	0.00000	0.65994	0.00000
H	1.02883	1.01581	0.00000
C	-0.22636	-0.67495	0.00000
H	-1.25318	-1.03483	0.00000
C	0.82148	-1.66784	0.00000
H	1.85009	-1.31820	0.00000
C	0.58093	-2.98522	0.00000
H	-0.40887	-3.42179	0.00000
Cl	1.84945	-4.18316	0.00000
H	-2.44574	6.03230	0.00000

X = H (unsubstituted R) E = -310.895183 a.u. ZPE = 0.151431 a.u. G = -310.777415 a.u

C	-2.21079	3.69911	0.00000
H	-3.20494	3.26321	0.00000
C	-1.10936	2.93248	0.00000
H	-0.13243	3.41244	0.00000
C	-1.11528	1.48602	0.00000
H	-2.08900	0.99890	0.00000
C	0.00000	0.71977	0.00000
H	0.97157	1.21146	0.00000
C	-0.00028	-0.72004	0.00000
H	-0.97180	-1.21178	0.00000
C	1.11511	-1.48622	0.00000
H	2.08871	-0.99893	0.00000
C	1.10923	-2.93264	0.00000
H	0.13263	-3.41306	0.00000
C	2.21123	-3.69862	0.00000
H	3.20497	-3.26202	0.00000
H	2.15062	-4.78003	0.00000
H	-2.14955	4.78062	0.00000

X = NH2 E = -366.275792 a.u. ZPE = 0.168717 a.u. G = -366.142569 a.u.

C	-5.00103	-0.20993	0.00018
H	-5.09722	-1.29116	0.00315
C	-3.79784	0.38734	-0.00089
H	-3.75363	1.47538	-0.00410
C	-2.52778	-0.30236	0.00155
H	-2.55760	-1.39075	0.00431
C	-1.32475	0.32134	0.00040
H	-1.30939	1.41099	-0.00260
C	-0.04853	-0.33931	0.00221
H	-0.05429	-1.42804	0.00467
C	1.14371	0.30917	0.00008
H	1.13351	1.39892	-0.00472
C	2.43536	-0.31961	0.00541
H	2.46813	-1.40775	0.00441
C	3.59670	0.37373	0.00294

H	3.57747	1.45980	0.00186
N	4.86607	-0.16526	-0.06777
H	5.62885	0.39488	0.27803
H	4.96610	-1.15195	0.12018
H	-5.91943	0.36432	-0.00210

X = OH E = -386.144005 a.u. ZPE = 0.156494 a.u. G = -386.022876 a.u.

C	-3.55539	-0.72674	0.00000
H	-3.49179	-1.80875	0.00000
C	-2.46719	0.06373	0.00000
H	-2.59768	1.14526	0.00000
C	-1.11949	-0.44737	0.00000
H	-1.00842	-1.53058	0.00000
C	0.00000	0.31506	0.00000
H	-0.10231	1.39903	0.00000
C	1.33837	-0.21335	0.00000
H	1.43620	-1.29829	0.00000
C	2.46605	0.53568	0.00000
H	2.37812	1.62091	0.00000
C	3.80464	-0.01140	0.00000
H	3.88199	-1.09729	0.00000
C	4.93128	0.71868	0.00000
H	4.90366	1.80378	0.00000
O	-4.85072	-0.31742	0.00000
H	-4.89271	0.64656	0.00000
H	5.90914	0.25296	0.00000

X = O^- E = -385.610479 a.u. ZPE = 0.141946 a.u. G = -385.503481 a.u.

C	-3.73187	-0.13192	0.00000
H	-3.60215	-1.24344	0.00000
C	-2.50882	0.58148	0.00000
H	-2.55730	1.66830	0.00000
C	-1.27304	-0.06800	0.00000
H	-1.31499	-1.16094	0.00000
C	0.00000	0.49094	0.00000
H	0.09009	1.57664	0.00000
C	1.18789	-0.27135	0.00000
H	1.05403	-1.35636	0.00000
C	2.48765	0.18376	0.00000
H	2.66639	1.25872	0.00000
C	3.62963	-0.67632	0.00000
H	3.40402	-1.74499	0.00000
C	4.93991	-0.32117	0.00000
H	5.24668	0.72094	0.00000
O	-4.88781	0.32780	0.00000
H	5.72758	-1.06573	0.00000

R = polyene, n = 5

X = NO2 E = -592.891494 a.u. ZPE = 0.187923 a.u. G = -592.745631 a.u.

C	-1.14437	0.96696	0.00000
H	-2.10409	0.45604	0.00000
C	0.00000	0.23187	0.00000
H	0.95247	0.75906	0.00000
C	0.05012	-1.19833	0.00000
H	-0.89749	-1.73308	0.00000
C	1.20832	-1.91054	0.00000
H	2.15208	-1.36768	0.00000
C	1.28289	-3.34264	0.00000
H	0.34207	-3.88989	0.00000
C	2.44550	-4.03980	0.00000
H	3.38633	-3.49200	0.00000
C	2.53049	-5.48188	0.00000
H	1.58863	-6.02642	0.00000
C	3.68404	-6.16831	0.00000
H	4.64444	-5.66282	0.00000
C	-1.14482	2.39901	0.00000
H	-0.19133	2.91899	0.00000
C	-2.26080	3.15115	0.00000
H	-3.27718	2.78659	0.00000
O	-1.09039	5.14659	0.00000
O	-3.26877	5.19007	0.00000
N	-2.19006	4.59487	0.00000
H	3.69963	-7.25119	0.00000

X = CN E = -480.594408 a.u. ZPE = 0.184130 a.u. G = -480.450241 a.u.

C	3.07789	-2.73532	0.00000
H	3.83658	-1.95453	0.00000
C	1.77071	-2.37772	0.00000
H	1.01319	-3.15947	0.00000
C	1.30814	-1.01935	0.00000
H	2.06811	-0.23971	0.00000
C	0.00000	-0.65180	0.00000
H	-0.76379	-1.42700	0.00000
C	-0.44652	0.70956	0.00000
H	0.32262	1.48022	0.00000
C	-1.74925	1.09539	0.00000
H	-2.52755	0.33529	0.00000
C	-2.16479	2.46821	0.00000
H	-1.38422	3.22473	0.00000
C	-3.45757	2.87201	0.00000
H	-4.26663	2.14831	0.00000
C	3.54797	-4.10162	0.00000
H	2.78637	-4.87867	0.00000
C	4.84297	-4.45496	0.00000
H	5.63338	-3.71117	0.00000
C	-3.83926	4.23736	0.00000
N	-4.17246	5.34653	0.00000

H	5.14742	-5.49429	0.00000

X = Cl E = -847.947041 a.u. ZPE = 0.175973 a.u. G = -847.810728 a.u.

C	1.78460	3.97651	0.00000
H	2.80136	3.58730	0.00000
C	0.74892	3.10323	0.00000
H	-0.26619	3.49728	0.00000
C	0.89159	1.67381	0.00000
H	1.90648	1.27954	0.00000
C	-0.14638	0.79911	0.00000
H	-1.16160	1.19236	0.00000
C	0.00000	-0.62935	0.00000
H	1.01608	-1.02040	0.00000
C	-1.03354	-1.50640	0.00000
H	-2.05106	-1.12105	0.00000
C	-0.86972	-2.93952	0.00000
H	0.14302	-3.33265	0.00000
C	-1.90051	-3.79523	0.00000
H	-2.93964	-3.49442	0.00000
C	1.63558	5.41421	0.00000
H	0.61630	5.79561	0.00000
C	2.65717	6.28514	0.00000
H	3.68903	5.94839	0.00000
Cl	-1.69709	-5.52802	0.00000
H	2.49044	7.35529	0.00000

X = H (unsubstituted R) E = -388.322972 a.u. ZPE = 0.184657 a.u. G = -388.175540 a.u.

C	1.18840	-2.84512	0.00000
H	0.23665	-3.37377	0.00000
C	1.18525	-1.49056	0.00000
H	2.14019	-0.96717	0.00000
C	-0.00021	-0.67857	0.00000
H	-0.95571	-1.20056	0.00000
C	0.00000	0.67846	0.00000
H	0.95551	1.20044	0.00000
C	-1.18543	1.49049	0.00000
H	-2.14042	0.96719	0.00000
C	-1.18846	2.84506	0.00000
H	-0.23666	3.37361	0.00000
C	-2.38553	3.65530	0.00000
H	-3.33288	3.11949	0.00000
C	-2.39771	4.99774	0.00000
H	-1.47732	5.57307	0.00000
C	2.38559	-3.65522	0.00000
H	3.33282	-3.11916	0.00000
C	2.39804	-4.99764	0.00000
H	1.47773	-5.57312	0.00000
H	-3.32552	5.55656	0.00000
H	3.32595	-5.55623	0.00000

X = NH2 E = -443.703724 a.u. ZPE = 0.202012 a.u. G = -443.540655 a.u.

C	-3.76878	-0.29553	0.00091
H	-3.80263	-1.38376	0.00331
C	-2.56242	0.32335	0.00025
H	-2.54076	1.41267	-0.00226
C	-1.29256	-0.34601	0.00240
H	-1.30646	-1.43471	0.00447
C	-0.09225	0.29168	0.00175
H	-0.08828	1.38130	-0.00056
C	1.18661	-0.35653	0.00301
H	1.19080	-1.44531	0.00500
C	2.37520	0.30209	0.00040
H	2.35610	1.39166	-0.00400
C	3.67012	-0.31667	0.00448
H	3.71123	-1.40451	0.00247
C	4.82705	0.38549	0.00271
H	4.79994	1.47134	0.00270
C	-5.03577	0.39871	-0.00159
H	-4.98848	1.48649	-0.00410
C	-6.24022	-0.19628	-0.00116
H	-6.33838	-1.27730	0.00124
N	6.09915	-0.14444	-0.06739
H	6.86048	0.42228	0.27041
H	6.20806	-1.13069	0.11735
H	-7.15747	0.37976	-0.00327

X = OH E = -463.571766 a.u. ZPE = 0.189813 a.u. G = -463.420765 a.u.

C	2.38686	0.33279	-0.00003
H	2.37640	1.42156	-0.00005
C	1.20019	-0.32332	-0.00001
H	1.20294	-1.41214	0.00001
C	-0.08123	0.32458	-0.00001
H	-0.08071	1.41378	-0.00002
C	-1.27562	-0.32107	0.00002
H	-1.28142	-1.40985	0.00003
C	-2.55222	0.33760	0.00002
H	-2.54221	1.42678	0.00000
C	-3.74943	-0.29707	0.00004
H	-3.76851	-1.38563	0.00006
C	-5.02668	0.37926	0.00004
H	-4.99667	1.46743	0.00002
C	-6.21981	-0.23693	0.00006
H	-6.29864	-1.31949	0.00008
C	3.68005	-0.30132	-0.00003
H	3.70912	-1.39032	-0.00001
C	4.83799	0.38397	-0.00005

H	4.87611	1.46715	-0.00007
O	6.08880	-0.14545	-0.00006
H	6.03987	-1.10911	-0.00004
H	-7.14726	0.32249	0.00006

X = O⁻ E = -463.044915 a.u. ZPE = 0.175202 a.u. G = -462.908116 a.u.

C	-2.35047	0.84233	0.00000
H	-1.97731	1.86988	0.00000
C	-1.38023	-0.16063	0.00000
H	-1.71050	-1.19848	0.00000
C	0.00000	0.09384	0.00000
H	0.29283	1.14647	0.00000
C	1.03051	-0.83162	0.00000
H	0.77586	-1.89085	0.00000
C	2.40205	-0.48327	0.00000
H	2.62133	0.58702	0.00000
C	3.48075	-1.33369	0.00000
H	3.30158	-2.40832	0.00000
C	4.84313	-0.89197	0.00000
H	4.98165	0.19077	0.00000
C	5.95749	-1.66291	0.00000
H	5.89797	-2.74738	0.00000
C	-3.73371	0.70068	0.00000
H	-4.18665	-0.28804	0.00000
C	-4.60334	1.82360	0.00000
H	-4.06976	2.80609	0.00000
O	-5.84409	1.82244	0.00000
H	6.94872	-1.22484	0.00000

R = polyyne, n = 1

X = NO2 E = -281.893002 a.u. ZPE = 0.030648 a.u. G = -281.889748 a.u.

C	0.00012	-0.87775	0.00000
C	-0.00017	-2.07492	0.00000
O	-1.09293	1.07454	0.00000
O	1.09302	1.07450	0.00000
N	0.00000	0.52323	0.00000
H	-0.00038	-3.13885	0.00000

X = CN E = -169.623644 a.u. ZPE = 0.027218 a.u. G = -169.619687 a.u.

C	0.00000	0.00000	-1.83499
C	0.00000	0.00000	-0.63127
C	0.00000	0.00000	0.73766
N	0.00000	0.00000	1.89578
H	0.00000	0.00000	-2.89886

X = Cl E = -536.968801 a.u. ZPE = 0.018965 a.u. G = -536.972809 a.u.

C	0.00000	0.00000	-1.81811
C	0.00000	0.00000	-0.61766
Cl	0.00000	0.00000	1.02913
H	0.00000	0.00000	-2.88059

X = H (unsubstituted R) E = -77.356663 a.u. ZPE = 0.026985 a.u. G = -77.349286 a.u.

C	0.00000	0.00000	-0.59968
C	0.00000	0.00000	0.59968
H	0.00000	0.00000	1.66280
H	0.00000	0.00000	-1.66280

X = NH2 E = -132.731827 a.u. ZPE = 0.044144 a.u. G = -132.711534 a.u.

C	1.36544	0.00002	0.01097
C	0.16152	0.00001	-0.00310
N	-1.18595	-0.00001	-0.08020
H	-1.64322	-0.84093	0.24577
H	-1.64322	0.84093	0.24576
H	2.42632	-0.00011	0.02268

X = OH E = -152.592576 a.u. ZPE = 0.031729 a.u. G = -152.584203 a.u.

C	0.12244	-0.00546	0.00000
C	1.32174	0.01427	-0.00000
O	-1.18209	-0.10754	-0.00000
H	-1.59057	0.76766	-0.00000
H	2.38218	0.03984	0.00001

X = O⁻ E = -152.059023 a.u. ZPE = 0.018782 a.u. G = -152.062925 a.u.

C	-0.00512	0.02042	-0.00034
C	-1.25283	-0.10139	0.00008
O	1.21789	0.01117	0.00018
H	-2.19546	0.39646	0.00013

R = polyyne, n = 2

X = NO2 E = -358.067632 a.u. ZPE = 0.040705 a.u. G = -358.057799 a.u.

C	2.37302	0.00003	-0.00008
C	3.58029	-0.00000	0.00041
C	1.01488	0.00004	-0.00054
C	-0.19221	-0.00009	-0.00077
O	-2.13821	-1.09409	0.00028
O	-2.13808	1.09412	0.00028
N	-1.58423	0.00002	0.00004
H	4.64403	-0.00024	0.00105

X = CN E = -245.800080 a.u. ZPE = 0.037668 a.u. G = -245.789028 a.u.

C	0.00000	0.00000	0.57221
C	0.00000	0.00000	-0.64204
C	0.00000	0.00000	1.92775
C	0.00000	0.00000	3.13616
C	0.00000	0.00000	-2.00308
N	0.00000	0.00000	-3.16368
H	0.00000	0.00000	4.19974

X = Cl E = -613.143809 a.u. ZPE = 0.028236 a.u. G = -613.142703 a.u.

C	0.00000	0.00000	0.67739
C	0.00000	0.00000	-0.53135
C	0.00000	0.00000	2.03901
C	0.00000	0.00000	3.24703
Cl	0.00000	0.00000	-2.17072
H	0.00000	0.00000	4.30960

X = H (unsubstituted R) E = -153.531036 a.u. ZPE = 0.037619 a.u. G = -153.516794 a.u.

C	0.00000	0.00000	-0.68240
C	0.00000	0.00000	-1.88969
C	0.00000	0.00000	0.68240
C	0.00000	0.00000	1.88969
H	0.00000	0.00000	-2.95237
H	0.00000	0.00000	2.95235

X = NH2 E = -208.908960 a.u. ZPE = 0.054417 a.u. G = -208.881887 a.u.

C	0.11608	-0.00007	-0.00580
C	-1.09657	-0.00004	-0.00398
C	1.47698	0.00005	0.00119
C	2.68703	-0.00000	0.00729
N	-2.43308	0.00003	-0.06193
H	-2.90934	-0.84752	0.21473
H	-2.90920	0.84768	0.21469
H	3.74893	0.00005	0.01183

X = OH E = -228.767482 a.u. ZPE = 0.042210 a.u. G = -228.752067 a.u.

C	-1.45060	0.00151	-0.00012
C	-2.65890	0.01337	0.00004
C	-0.08712	-0.01723	0.00011
C	1.11957	-0.00180	-0.00002
O	2.41891	-0.09714	-0.00001
H	2.83199	0.77731	0.00000
H	-3.72097	0.02472	0.00003

X = O^- E = -228.263320 a.u. ZPE = 0.029663 a.u. G = -228.259687 a.u.

C	0.00000	0.00000	-1.34610
C	0.00000	0.00000	-2.57446
C	0.00000	0.00000	-0.00044
C	0.00000	0.00000	1.24607
O	0.00000	0.00000	2.46044
H	0.00000	0.00000	-3.63390

R = polyyne, n = 3

X = NO2 E = -434.245457 a.u. ZPE = 0.050943 a.u. G = -434.228751 a.u.

C	-3.78534	-0.00004	0.00006
C	-4.99497	0.00009	0.00010
C	-2.43272	-0.00010	-0.00008
C	-1.21388	-0.00004	-0.00009
C	0.13388	-0.00004	-0.00013
C	1.34444	-0.00002	-0.00013
O	3.28854	-1.09452	0.00008
O	3.28847	1.09459	0.00008
N	2.73342	0.00002	0.00003
H	-6.05851	0.00023	0.00019

X = CN E = -321.978214 a.u. ZPE = 0.047521 a.u. G = -321.954238 a.u.

C	-0.57800	0.16343	-0.35883
C	-1.58408	0.44804	-0.98336
C	0.53305	-0.15086	0.33085
C	1.54093	-0.43591	0.95656
C	2.65797	-0.75185	1.65003
C	3.65753	-1.03454	2.27054
C	-2.70646	0.76559	-1.68012
N	-3.66595	1.03708	-2.27570
H	4.53600	-1.28297	2.81589

X = Cl E = -689.321800 a.u. ZPE = 0.038904 a.u. G = -689.311673 a.u.

C	-0.52614	-0.00890	-0.00057
C	-1.73753	0.01856	0.00002
C	0.82574	0.00530	-0.00045
C	2.04430	-0.00215	-0.00070
C	3.39936	-0.00026	-0.00007
C	4.60953	-0.00154	0.00070
Cl	-3.37434	-0.00382	0.00030
H	5.67221	-0.00105	0.00148

X = H (unsubstituted R) E = -229.708615 a.u. ZPE = 0.048116 a.u. G = -229.687219 a.u.

C	0.00000	0.00000	-1.96436

C	0.00000	0.00000	-3.17421
C	0.00000	0.00000	-0.60883
C	0.00000	0.00000	0.60882
C	0.00000	0.00000	1.96438
C	0.00000	0.00000	3.17422
H	0.00000	0.00000	-4.23696
H	0.00000	0.00000	4.23693

X = NH2 E = -285.088148 a.u. ZPE = 0.064741 a.u. G = -285.054080 a.u.

C	-1.14997	-0.00018	-0.00709
C	-2.36614	0.00000	-0.00204
C	0.19987	-0.00009	-0.00385
C	1.42072	-0.00026	-0.00060
C	2.77544	0.00003	0.00261
C	3.98683	0.00020	0.00547
N	-3.69738	0.00016	-0.05159
H	-4.18402	-0.85099	0.19325
H	-4.18385	0.85142	0.19320
H	5.04900	0.00032	0.00774

X = OH E = -304.945824 a.u. ZPE = 0.053115 a.u. G = -304.922722 a.u.

C	-1.17241	-0.02185	-0.00005
C	-2.38172	0.00390	-0.00000
C	0.18135	-0.00927	-0.00006
C	1.39993	-0.00444	-0.00000
C	2.75578	0.00519	0.00003
C	3.96628	0.01309	0.00002
O	-3.67863	-0.09036	0.00004
H	-4.09483	0.78319	0.00002
H	5.02862	0.01990	0.00002

X = O^- E = -230.734470 a.u. ZPE = 0.075253 a.u. G = -230.687166 a.u.

C	0.00000	0.00000	-2.65352
C	0.00000	0.00000	-3.87770
C	0.00000	0.00000	-1.30740
C	0.00000	0.00000	-0.06742
C	0.00000	0.00000	1.26028
C	0.00000	0.00000	2.51204
O	0.00000	0.00000	3.71748
H	0.00000	0.00000	-4.93754

R = polyyne, n = 4

X = NO2 E = -510.424197 a.u. ZPE = 0.059767 a.u. G = -510.402286 a.u.

C	-5.15414	0.00000	-0.00001
C	-6.36466	0.00003	0.00009
C	-3.80318	0.00001	-0.00002
C	-2.58132	-0.00003	0.00000
C	-1.24042	-0.00001	-0.00006
C	-0.01735	-0.00002	-0.00008
C	1.32698	-0.00001	-0.00008
C	2.53894	-0.00000	-0.00006
O	4.48206	1.09487	0.00009
O	4.48208	-1.09484	0.00009
N	3.92654	0.00001	-0.00005
H	-7.42810	0.00002	0.00016

X = CN E = -398.157061 a.u. ZPE = 0.056117 a.u. G = -398.131926 a.u.

C	1.97527	-0.00240	-0.00003
C	3.19463	-0.00056	-0.00002
C	0.63384	-0.00392	-0.00013
C	-0.59053	-0.00320	0.00020
C	-1.93085	-0.00306	-0.00007
C	-3.15306	-0.00078	0.00009
C	-4.50400	0.00161	-0.00004
C	-5.71466	0.00423	-0.00002
C	4.55239	0.00201	0.00001
N	5.71426	0.00427	0.00002
H	-6.77800	0.00657	-0.00005

X = Cl E = -765.500657 a.u. ZPE = 0.047956 a.u. G = -765.482357 a.u.

C	1.75794	0.00113	-0.00015
C	2.97064	0.00057	0.00006
C	0.40914	0.00109	-0.00001
C	-0.81320	0.00100	0.00009
C	-2.15674	0.00034	0.00026
C	-3.37840	0.00062	-0.00011
C	-4.73107	-0.00084	0.00010
C	-5.94198	-0.00097	-0.00035
Cl	4.60629	-0.00094	-0.00002
H	-7.00476	-0.00174	0.00094

X = H (unsubstituted R) E = -305.887282 a.u. ZPE = 0.057037 a.u. G = -305.860584 a.u.

C	0.00000	0.00000	-3.24616
C	0.00000	0.00000	-4.45698
C	0.00000	0.00000	-1.89353
C	0.00000	0.00000	-0.67213
C	0.00000	0.00000	0.67212
C	0.00000	0.00000	1.89351
C	0.00000	0.00000	3.24619
C	0.00000	0.00000	4.45698
H	0.00000	0.00000	-5.51981
H	0.00000	0.00000	5.51976

X = NH2 E = -361.267744 a.u. ZPE = 0.073316 a.u. G = -361.228909 a.u.

C	2.42230	-0.00013	-0.00773
C	3.64009	-0.00000	-0.00028
C	1.07660	-0.00041	-0.00616
C	-0.14847	0.00034	-0.00457
C	-1.49106	-0.00022	-0.00209
C	-2.71417	0.00015	0.00046
C	-4.06666	0.00002	0.00333
C	-5.27842	0.00000	0.00602
N	4.96819	0.00014	-0.04287
H	5.46098	0.85369	0.17912
H	5.46119	-0.85327	0.17918
H	-6.34080	0.00014	0.00801

X = OH E = -381.124912 a.u. ZPE = 0.062108 a.u. G = -381.096348 a.u.

C	-2.44112	-0.02237	0.00271
C	-3.65141	0.00821	0.00050
C	-1.09066	-0.01510	0.00306
C	0.13172	-0.01098	-0.00039
C	1.47609	-0.00549	-0.00271
C	2.69803	0.00152	-0.00352
C	4.05095	0.00758	-0.00154
C	5.26217	0.01335	0.00282
O	-4.94703	-0.08354	-0.00139
H	-5.36320	0.79027	-0.00084
H	6.32470	0.01775	0.00642

X = O^- E = -380.651970 a.u. ZPE = 0.050389 a.u. G = -380.633961 a.u.

C	0.00000	0.00000	-3.94947
C	0.00000	0.00000	-5.17082
C	0.00000	0.00000	-2.60296
C	0.00000	0.00000	-1.36558
C	0.00000	0.00000	-0.03694
C	0.00000	0.00000	1.20924
C	0.00000	0.00000	2.52810
C	0.00000	0.00000	3.78304
O	0.00000	0.00000	4.98292
H	0.00000	0.00000	-6.23100

R = polyyne, n = 5

X = NO2 E = -586.603346 a.u. ZPE = 0.069580 a.u. G = -586.575123 a.u.

C	-2.54509	0.00023	0.00408
C	-3.75775	0.00009	0.00053
C	-1.20228	-0.00150	0.00843
C	0.02261	0.00058	0.00925
C	1.35942	-0.00359	0.00815
C	2.58592	0.00390	0.00581
C	3.92437	-0.00093	0.00301
C	5.14745	0.00136	-0.00090
O	-5.70033	-1.09487	-0.00607
O	-5.70039	1.09516	-0.00606
N	-5.14457	0.00018	-0.00424
C	6.49769	0.00016	-0.00537
C	7.70863	-0.00064	-0.00962
H	8.77199	-0.00151	-0.01344

X = CN E = -474.336250 a.u. ZPE = 0.066365 a.u. G = -474.304512 a.u.

C	-5.78852	0.00149	-0.00019
C	-6.99954	0.00171	-0.00039
C	-4.43818	0.00173	0.00013
C	-3.21499	-0.00180	-0.00010
C	-1.87658	0.00254	0.00075
C	-0.64974	-0.00615	-0.00021
C	0.68645	-0.00060	0.00057
C	1.91264	-0.00356	0.00007
C	3.25252	-0.00134	0.00013
C	4.47258	0.00024	-0.00004
C	5.82988	0.00179	-0.00018
N	6.99195	0.00314	-0.00036
H	-8.06283	0.00165	-0.00073

X = Cl E = -841.679911 a.u. ZPE = 0.057388 a.u. G = -841.657821 a.u.

C	6.04682	0.00829	-0.00017
C	7.25809	0.01711	-0.00025
C	4.69548	-0.00021	0.00023
C	3.47242	-0.00633	0.00058
C	2.13219	-0.01174	0.00022
C	0.90604	-0.01416	0.00021
C	-0.43356	-0.01381	-0.00025
C	-1.65763	-0.01076	-0.00051
C	-3.00486	-0.00464	-0.00039
C	-4.21817	0.00142	-0.00013
Cl	-5.85304	0.01084	0.00019
H	8.32089	0.02483	-0.00032

X = H (unsubstituted R) E = -382.066425 a.u. ZPE = 0.066598 a.u. G = -382.031400 a.u.

C	4.52763	-0.00014	0.00020
C	5.73882	0.00036	0.00035
C	3.17620	-0.00129	-0.00002
C	1.95336	0.00134	-0.00046
C	0.61281	-0.00419	-0.00040
C	-0.61281	0.00457	-0.00015
C	-1.95336	-0.00107	0.00011

C	-3.17620	0.00138	-0.00007
C	-4.52763	-0.00005	0.00003
C	-5.73881	-0.00080	0.00027
H	-6.80168	-0.00164	0.00052
H	6.80169	0.00095	0.00042

X = NH2 E = -437.447470 a.u. ZPE = 0.083815 a.u. G = -437.401349 a.u.

C	5.35404	0.00063	0.00363
C	6.56590	0.00046	0.00580
C	4.00263	0.00140	0.00107
C	2.77870	-0.00114	-0.00067
C	1.43897	0.00328	-0.00358
C	0.21129	-0.00511	-0.00351
C	-1.12677	0.00053	-0.00554
C	-2.35373	-0.00277	-0.00535
C	-3.69733	-0.00069	-0.00544
C	-4.91601	0.00036	0.00126
N	-6.24221	0.00189	-0.03828
H	-6.74050	-0.85237	0.16737
H	-6.73855	0.85744	0.16676
H	7.62841	-0.00001	0.00770

X = OH E = -457.304311 a.u. ZPE = 0.071350 a.u. G = -457.270300 a.u.

C	3.71393	-0.02002	0.00051
C	4.92475	0.01112	0.00052
C	2.36500	-0.01493	0.00027
C	1.14101	-0.01331	-0.00122
C	-0.19931	-0.00669	0.00002
C	-1.42562	-0.00874	-0.00248
C	-2.76622	0.00028	-0.00089
C	-3.98947	0.00216	-0.00049
C	-5.34110	0.00818	0.00069
C	-6.55261	0.01304	0.00166
O	6.21957	-0.07974	0.00072
H	6.63650	0.79390	0.00011
H	-7.61525	0.01740	0.00252

X = O^- E = -456.840565 a.u. ZPE = 0.060121 a.u. G = -456.813783 a.u.

C	3.79899	-0.00129	-0.00011
C	5.05621	-0.00369	0.00003
C	2.48590	0.00174	-0.00021
C	1.23611	0.00585	0.00060
C	-0.08396	0.00468	-0.00097
C	-1.32801	0.01100	0.00081
C	-2.65728	0.00337	-0.00067
C	-3.89248	0.00067	-0.00014
C	-5.23950	-0.00414	-0.00004
C	-6.45889	-0.00812	0.00038
O	6.25210	-0.00606	0.00018
H	-7.51937	-0.01191	0.00042

R = acene, n = 1

X = NO2 E = -436.874726 a.u. ZPE = 0.102523 a.u. G = -436.804235 a.u.

C	-1.82148	-1.21013	0.00002
C	-0.43013	-1.21862	-0.00004
C	0.24180	-0.00001	-0.00007
C	-0.43014	1.21863	-0.00004
C	-1.82146	1.21014	0.00002
C	-2.51581	-0.00001	0.00005
H	-2.36262	-2.14873	0.00005
H	0.13471	-2.14051	-0.00008
H	0.13474	2.14050	-0.00006
H	-2.36264	2.14872	0.00005
O	2.29129	-1.08475	0.00009
O	2.29128	1.08476	0.00008
H	-3.59976	0.00002	0.00009
N	1.72262	-0.00000	-0.00014

X = CN E = -324.577834 a.u. ZPE = 0.098829 a.u. G = -324.509266 a.u.

C	-0.09027	-1.21520	0.00000
C	-1.48083	-1.20873	-0.00001
C	-2.17654	0.00002	0.00001
C	-1.48076	1.20877	-0.00000
C	-0.09024	1.21519	-0.00001
C	0.61029	-0.00004	0.00002
H	0.45782	-2.14914	0.00001
H	-2.02166	-2.14774	-0.00001
H	-2.02160	2.14777	0.00000
H	0.45795	2.14907	-0.00000
C	2.04218	-0.00003	0.00001
N	3.19786	0.00002	-0.00001
H	-3.26054	0.00007	0.00002

X = Cl E = -691.934292 a.u. ZPE = 0.090609 a.u. G = -691.873473 a.u.

C	0.17867	-1.21366	-0.00001
C	1.57265	-1.20552	0.00002
C	2.27221	0.00000	-0.00001
C	1.57262	1.20553	0.00001
C	0.17867	1.21366	0.00000
C	-0.50216	-0.00001	-0.00004
H	-0.37278	-2.14520	0.00001
H	2.10915	-2.14751	0.00002
H	2.10915	2.14751	0.00001
H	-0.37283	2.14518	0.00002
Cl	-2.26262	0.00000	0.00001
H	3.35589	0.00003	-0.00001

X = H (unsubstituted R) E = -232.311304 a.u. ZPE = 0.100077 a.u. G = -232.238695 a.u.

C	0.68948	1.21232	0.00003
C	-0.70510	1.20323	-0.00003
C	-1.39470	-0.00903	-0.00006
C	-0.68946	-1.21226	-0.00003
C	0.70512	-1.20329	0.00003
C	1.39463	0.00903	0.00006
H	1.22577	2.15482	0.00005
H	-1.25347	2.13883	-0.00006
H	-1.22567	-2.15488	-0.00005
H	1.25357	-2.13877	0.00006
H	2.47906	0.01605	0.00011
H	-2.47910	-0.01605	-0.00011

X = NH2 E = -287.687731 a.u. ZPE = 0.116590 a.u. G = -287.600330 a.u.

C	-0.22054	1.20625	-0.00466
C	1.17070	1.20089	0.00333
C	1.87970	0.00001	0.00748
C	1.17065	-1.20093	0.00331
C	-0.22051	-1.20624	-0.00467
C	-0.93698	0.00005	-0.00751
H	-0.75973	2.14854	-0.01320
H	1.70373	2.14558	0.00696
H	1.70372	-2.14559	0.00702
H	-0.75983	-2.14846	-0.01338
N	-2.33367	-0.00004	-0.07285
H	-2.78658	-0.83735	0.26258
H	-2.78669	0.83743	0.26206
H	2.96296	-0.00002	0.01427

X = OH E = -307.558731 a.u. ZPE = 0.103942 a.u. G = -307.483844 a.u.

C	0.22076	-1.22156	-0.00001
C	-1.16979	-1.18877	0.00002
C	-1.85529	0.02725	0.00004
C	-1.13125	1.21715	0.00003
C	0.26277	1.19772	-0.00000
C	0.93814	-0.02401	-0.00002
H	0.76395	-2.15881	-0.00003
H	-1.72211	-2.12196	0.00003
H	-1.64881	2.16998	0.00005
H	0.82296	2.12841	-0.00001
O	2.30538	-0.11043	-0.00005
H	2.68738	0.77332	-0.00007
H	-2.93849	0.04573	0.00007

X = O^- E = -306.994057 a.u. ZPE = 0.089440 a.u. G = -306.933566 a.u.

C	-0.28747	1.21169	0.00001
C	1.10022	1.20005	0.00007
C	1.82786	0.00000	-0.00007
C	1.10021	-1.20006	0.00007
C	-0.28746	-1.21169	0.00001
C	-1.07794	0.00001	-0.00040
H	-0.83081	2.15362	0.00025
H	1.63712	2.14846	0.00014
H	1.63713	-2.14846	0.00015
H	-0.83084	-2.15359	0.00023
O	-2.34732	-0.00001	0.00015
H	2.91348	-0.00001	0.00002

R = acene, n = 2

X = NO2 E = -590.553022 a.u. ZPE = 0.149066 a.u. G = -590.439561 a.u.

C	-3.58791	0.17962	-0.00005
C	-2.62425	1.16029	0.00017
C	-1.24734	0.82155	0.00014
C	-0.87871	-0.56196	-0.00013
C	-1.89848	-1.55074	-0.00035
C	-3.22312	-1.18777	-0.00032
H	-0.50403	2.85455	0.00058
H	-2.90702	2.20743	0.00038
C	-0.22307	1.80723	0.00036
C	0.49366	-0.90782	-0.00016
H	-1.61329	-2.59710	-0.00056
H	-3.99564	-1.94790	-0.00049
C	1.44142	0.08518	0.00001
C	1.10217	1.45626	0.00032
H	0.80213	-1.94503	-0.00036
H	1.89014	2.19597	0.00050
O	3.14887	-1.48624	-0.00006
O	3.69475	0.61370	0.00003
H	-4.63734	0.45166	-0.00002
N	2.86998	-0.29291	0.00003

X = CN E = -478.255763 a.u. ZPE = 0.145500 a.u. G = -478.144154 a.u.

C	1.84091	0.00408	-0.00000
C	0.85143	-0.96138	-0.00000
C	-0.51539	-0.59438	0.00000
C	-0.86181	0.79421	-0.00000
C	0.17889	1.76133	-0.00000
C	1.49657	1.38573	-0.00000
H	-1.28699	-2.61617	-0.00000
H	1.12105	-2.01117	-0.00000
C	-1.55289	-1.56455	0.00000

C	-2.23307	1.15695	0.00000
H	-0.08391	2.81357	-0.00000
H	2.28586	2.12695	0.00000
C	-3.21394	0.19392	0.00000
C	-2.87131	-1.17928	0.00000
H	-2.49668	2.20920	-0.00000
H	-3.65626	-1.92672	0.00000
C	3.21937	-0.37953	0.00000
N	4.33469	-0.68313	0.00000
H	-4.25855	0.48371	0.00000

X = Cl E = -845.611928 a.u. ZPE = 0.137282 a.u. G = -845.508050 a.u.

C	1.70336	0.00976	-0.00000
C	0.73284	-0.95833	0.00000
C	-0.63680	-0.58465	0.00000
C	-0.98196	0.80355	0.00000
C	0.06197	1.76534	0.00000
C	1.38151	1.38568	-0.00000
H	-1.41610	-2.60317	0.00001
H	1.00362	-2.00716	0.00000
C	-1.67778	-1.55037	0.00000
C	-2.35271	1.17040	-0.00000
H	-0.19408	2.81952	0.00000
H	2.17584	2.12089	-0.00000
C	-3.33749	0.21170	0.00000
C	-2.99590	-1.16144	-0.00000
H	-2.61228	2.22388	0.00000
H	-3.78168	-1.90832	-0.00000
Cl	3.40141	-0.45292	-0.00000
H	-4.38142	0.50411	-0.00000

X = H (unsubstituted R) E = -385.988964 a.u. ZPE = 0.146868 a.u. G = -385.873316 a.u.

C	-2.43042	0.70772	0.00000
C	-1.24371	1.40080	0.00000
C	0.00006	0.71557	0.00000
C	-0.00002	-0.71576	0.00000
C	-1.24383	-1.40082	0.00000
C	-2.43044	-0.70761	0.00000
H	1.24222	2.48600	0.00000
H	-1.24238	2.48600	0.00000
C	1.24377	1.40083	0.00000
C	1.24373	-1.40077	0.00000
H	-1.24254	-2.48602	0.00000
H	-3.37318	-1.24320	0.00000
C	2.43045	-0.70762	0.00000
C	2.43042	0.70766	0.00000
H	1.24262	-2.48600	0.00000
H	3.37320	1.24324	0.00000
H	-3.37311	1.24338	0.00000
H	3.37316	-1.24329	0.00000

X = NH2 E = -441.365674 a.u. ZPE = 0.163243 a.u. G = -441.235278 a.u.

C	2.10942	-0.20689	-0.00541
C	1.04266	-1.08583	-0.00340
C	-0.29505	-0.62018	-0.00303
C	-0.54882	0.78827	0.00026
C	0.56493	1.66884	-0.00044
C	1.84996	1.19305	-0.00663
H	-1.22334	-2.57570	-0.00459
H	1.22379	-2.15676	-0.00613
C	-1.40643	-1.50599	-0.00166
C	-1.88823	1.25230	0.00471
H	0.38685	2.73937	0.00109
H	2.68698	1.88419	-0.01837
C	-2.94215	0.36854	0.00549
C	-2.69433	-1.02433	0.00242
H	-2.06966	2.32251	0.00691
H	-3.52975	-1.71595	0.00272
N	3.43212	-0.65231	-0.06760
H	4.12454	-0.02398	0.31250
H	3.58665	-1.60832	0.21706
H	-3.96264	0.73416	0.00827

X = OH E = -461.236778 a.u. ZPE = 0.150726 a.u. G = -461.118753 a.u.

C	-2.10510	-0.20455	0.00000
C	-1.05371	-1.09188	0.00000
C	0.28562	-0.62184	0.00000
C	0.52969	0.78787	0.00000
C	-0.58504	1.66836	0.00000
C	-1.87056	1.19191	0.00000
H	1.21883	-2.57426	0.00000
H	-1.23785	-2.16280	0.00000
C	1.39824	-1.50402	0.00000
C	1.86830	1.25631	0.00000
H	-0.40675	2.73858	0.00000
H	-2.72329	1.85987	0.00000
C	2.92516	0.37660	0.00000
C	2.68417	-1.01713	0.00000
H	2.04559	2.32707	0.00000
H	3.52258	-1.70501	0.00000
O	-3.41857	-0.59091	0.00000
H	-3.47536	-1.55244	-0.00000
H	3.94422	0.74644	0.00000

X = O^- E = -460.681499 a.u. ZPE = 0.136478 a.u. G = -460.577639 a.u.

C	-2.89287	0.41551	0.00001

C	-1.80394	1.26929	0.00000
C	-0.47943	0.78511	-0.00002
C	-0.23473	-0.63813	-0.00001
C	-1.38576	-1.48985	0.00001
C	-2.66557	-0.98269	0.00001
H	0.49379	2.71995	0.00004
H	-1.95837	2.34653	0.00002
C	0.65965	1.64337	0.00001
C	1.07933	-1.13051	-0.00004
H	-1.22849	-2.56532	0.00003
H	-3.51367	-1.66273	0.00003
C	2.23330	-0.28826	-0.00015
C	1.93252	1.14603	-0.00001
H	1.24505	-2.20505	0.00002
H	2.78986	1.81447	0.00005
H	-3.90539	0.80657	0.00002
O	3.42778	-0.70420	0.00011

R = acene, n = 3

X = NO2 E = -744.224811 a.u. ZPE = 0.195402 a.u. G = -744.068573 a.u.

C	-4.75563	0.28944	-0.00005
C	-3.69905	1.15785	-0.00004
C	-2.35479	0.67566	-0.00003
C	-2.13317	-0.75032	0.00002
C	-3.26578	-1.62165	0.00002
C	-4.53677	-1.11825	-0.00001
C	-1.25295	1.54003	-0.00002
C	-0.82187	-1.23498	0.00006
C	0.27756	-0.36886	0.00004
C	0.05731	1.05760	0.00001
C	1.19419	1.92637	0.00003
H	1.02932	2.99818	0.00003
C	2.46635	1.43343	0.00004
C	2.65698	0.02416	0.00006
C	1.61386	-0.85850	0.00007
H	-1.42254	2.61217	-0.00004
H	-3.86666	2.22962	-0.00009
H	-3.09684	-2.69322	0.00005
H	-5.38847	-1.78881	0.00001
H	-0.65128	-2.30685	0.00007
H	3.33202	2.08040	0.00002
H	1.80893	-1.92301	0.00008
O	4.95079	0.31167	-0.00006
O	4.18757	-1.71986	-0.00016
H	-5.77108	0.66889	-0.00007
N	4.03460	-0.50338	0.00007

X = CN E = -631.927373 a.u. ZPE = 0.191807 a.u. G = -631.773042 a.u.

C	3.07163	-0.10902	0.00001
C	1.98591	-0.95419	-0.00001
C	0.65945	-0.43725	-0.00001
C	0.46973	0.99290	-0.00000
C	1.62493	1.83556	0.00001
C	2.88420	1.31267	0.00002
C	-0.46012	-1.27693	-0.00001
C	-0.82965	1.50558	-0.00001
C	-1.95077	0.66740	-0.00001
C	-1.76051	-0.76313	-0.00000
C	-2.91290	-1.60820	0.00001
H	-2.76870	-2.68342	0.00003
C	-4.17238	-1.07636	0.00001
C	-4.36018	0.33584	-0.00000
C	-3.28414	1.17978	-0.00001
H	-0.31522	-2.35274	-0.00001
H	2.13493	-2.02776	-0.00002
H	1.48411	2.91097	0.00001
H	3.75468	1.95661	0.00003
H	-0.97393	2.58152	-0.00001
H	-5.03861	-1.72811	0.00002
H	-3.42748	2.25511	-0.00003
C	4.39881	-0.64170	0.00000
N	5.47615	-1.06113	-0.00001
H	-5.36688	0.73804	0.00000

X = Cl E = -999.283410 a.u. ZPE = 0.183572 a.u. G = -999.136813 a.u.

C	-2.91903	-0.08669	0.00002
C	-1.85748	-0.94284	0.00009
C	-0.52523	-0.42607	0.00005
C	-0.33063	1.00288	0.00003
C	-1.48354	1.84677	-0.00002
C	-2.74645	1.32590	-0.00005
C	0.59345	-1.26547	0.00005
C	0.97008	1.51449	0.00003
C	2.09014	0.67604	0.00002
C	1.89547	-0.75348	0.00001
C	3.04705	-1.59973	-0.00003
H	2.90119	-2.67484	-0.00007
C	4.30803	-1.07075	-0.00007
C	4.49992	0.34099	-0.00003
C	3.42492	1.18601	0.00002
H	0.44852	-2.34132	0.00007
H	-2.01337	-2.01463	0.00013
H	-1.34336	2.92244	-0.00003
H	-3.61823	1.96728	-0.00010
H	1.11484	2.59051	0.00003
H	5.17253	-1.72496	-0.00010
H	3.56961	2.26129	0.00005
Cl	-4.56115	-0.71853	-0.00004

H	5.50761	0.74106	-0.00005

X = H (unsubstituted R) E = -539.660377 a.u. ZPE = 0.193230 a.u. G = -539.501943 a.u.

C	3.65592	0.71251	0.00005
C	2.47704	1.40539	0.00006
C	1.22207	0.72147	0.00003
C	1.22209	-0.72149	-0.00000
C	2.47711	-1.40535	-0.00000
C	3.65594	-0.71241	0.00002
C	-0.00003	1.40196	0.00003
C	0.00006	-1.40206	-0.00003
C	-1.22207	-0.72161	-0.00003
C	-1.22214	0.72135	0.00000
C	-2.47706	1.40539	0.00001
H	-2.47614	2.49045	0.00003
C	-3.65592	0.71250	-0.00002
C	-3.65597	-0.71239	-0.00005
C	-2.47707	-1.40529	-0.00006
H	-0.00005	2.48787	0.00005
H	2.47640	2.49048	0.00008
H	2.47653	-2.49044	-0.00003
H	4.60054	-1.24464	0.00002
H	0.00004	-2.48798	-0.00005
H	-4.60053	1.24478	-0.00002
H	-2.47666	-2.49043	-0.00008
H	4.60051	1.24476	0.00007
H	-4.60053	-1.24469	-0.00007

X = NH2 E = -594.827967 a.u. ZPE = 0.209562 a.u. G = -594.864388 a.u.

C	3.31334	-0.33484	-0.00338
C	2.18201	-1.11708	-0.00123
C	0.87899	-0.53958	-0.00366
C	0.75043	0.89764	-0.00096
C	1.94724	1.67889	-0.00142
C	3.17760	1.09219	-0.00693
C	-0.28626	-1.31580	-0.00345
C	-0.51992	1.47716	0.00187
C	-1.68426	0.69998	0.00179
C	-1.56072	-0.73718	-0.00083
C	-2.75569	-1.52130	-0.00090
H	-2.66646	-2.60283	-0.00308
C	-3.98864	-0.92829	0.00141
C	-4.10922	0.49081	0.00398
C	-2.99002	1.27786	0.00416
H	-0.20041	-2.39833	-0.00577
H	2.27069	-2.19943	-0.00142
H	1.86282	2.76069	-0.00140
H	4.07447	1.70350	-0.02042
H	-0.60694	2.55983	0.00368
H	-4.88504	-1.53856	0.00116
H	-3.07848	2.35958	0.00589
N	4.59736	-0.87858	-0.06471
H	5.33512	-0.31083	0.32532
H	4.67817	-1.84909	0.20063
H	-5.09469	0.94277	0.00563

X = OH E = -614.908574 a.u. ZPE = 0.197134 a.u. G = -614.747693 a.u.

C	3.30599	-0.33169	0.00000
C	2.19018	-1.12499	-0.00000
C	0.88535	-0.54395	-0.00000
C	0.76502	0.89378	-0.00000
C	1.96204	1.67561	0.00000
C	3.19366	1.08926	0.00000
C	-0.27961	-1.31797	-0.00000
C	-0.50520	1.47565	-0.00000
C	-1.67087	0.70136	-0.00000
C	-1.55273	-0.73590	-0.00000
C	-2.74916	-1.51740	0.00000
H	-2.66247	-2.59904	-0.00000
C	-3.98009	-0.92075	0.00000
C	-4.09634	0.49883	0.00000
C	-2.97548	1.28294	0.00000
H	-0.19618	-2.40066	-0.00000
H	2.28351	-2.20744	-0.00000
H	1.87670	2.75708	-0.00000
H	4.10356	1.67691	0.00000
H	-0.58972	2.55834	-0.00000
H	-4.87837	-1.52825	0.00000
H	-3.06088	2.36485	0.00000
O	4.58505	-0.81784	0.00000
H	4.56764	-1.78106	0.00001
H	-5.08070	0.95337	0.00000

X = O^{-} E = -614.360302 a.u. ZPE = 0.182800 a.u. G = -614.213718 a.u.

C	-4.06419	0.53358	0.00005
C	-2.91969	1.29682	0.00001
C	-1.62881	0.70414	-0.00002
C	-1.51631	-0.73903	-0.00003
C	-2.73253	-1.49690	0.00001
C	-3.96027	-0.88603	0.00006
C	-0.44107	1.45912	-0.00003
C	-0.25312	-1.33220	-0.00003
C	0.94784	-0.58195	-0.00003
C	0.81617	0.86938	-0.00005
C	2.02549	1.64372	0.00004
H	1.93855	2.72886	0.00013
C	3.24745	1.05001	0.00002
C	3.43310	-0.41227	-0.00042

C	2.22559	-1.16677	-0.00009
H	-0.51126	2.54528	-0.00000
H	-2.98792	2.38200	0.00002
H	-2.66269	-2.58127	0.00001
H	-4.86445	-1.48794	0.00007
H	-0.18509	-2.41730	-0.00003
H	4.15775	1.64376	0.00018
H	2.31399	-2.24978	0.00012
O	4.59560	-0.90228	0.00031
H	-5.04150	1.00502	0.00009

R = acene, n = 4

X = NO2 E = -897.894004 a.u. ZPE = 0.241606 a.u. G = -897.695035 a.u.

C	0.95513	-0.57125	0.00002
C	1.10277	0.87153	-0.00001
C	2.39866	1.42011	-0.00002
C	-0.05528	1.67729	-0.00001
C	-0.34045	-1.12194	0.00003
C	2.11095	-1.37649	0.00002
C	3.53548	0.61733	-0.00002
C	-1.32955	1.12693	0.00001
C	-1.47523	-0.31587	0.00002
C	3.38735	-0.82463	0.00000
C	4.85821	1.16746	-0.00002
C	-2.51651	1.93299	0.00002
C	-2.78784	-0.87409	0.00002
C	4.56955	-1.63443	-0.00000
C	5.95554	0.35637	-0.00000
C	-3.75756	1.37319	0.00002
C	-3.87416	-0.04901	0.00001
C	5.80945	-1.06532	-0.00000
H	2.51175	2.49973	-0.00004
H	0.05767	2.75681	-0.00002
H	-0.45535	-2.20114	0.00004
H	1.99838	-2.45618	0.00004
H	4.97016	2.24651	-0.00002
H	-2.40958	3.01211	0.00003
H	-2.92684	-1.94735	0.00001
H	4.45776	-2.71351	-0.00001
H	-4.65691	1.97215	0.00002
H	6.69564	-1.68963	-0.00000
O	-6.17834	0.12027	0.00001
O	-5.31238	-1.87014	-0.00006
H	6.95052	0.78681	0.00002
N	-5.22092	-0.64692	-0.00001

X = CN E = -785.596449 a.u. ZPE = 0.238007 a.u. G = -785.399441 a.u.

C	0.68325	0.83139	0.00000
C	0.56455	-0.61394	0.00000
C	-0.72010	-1.19059	0.00000
C	1.73717	-1.39463	0.00000
C	1.96809	1.40690	-0.00000
C	-0.49074	1.61240	0.00000
C	-1.87172	-0.40940	0.00000
C	3.00197	-0.81666	0.00000
C	3.12088	0.62809	-0.00000
C	-1.75387	1.03519	0.00000
C	-3.17504	-0.99112	0.00000
C	4.20085	-1.60182	0.00000
C	4.43247	1.20512	-0.00000
C	-2.95550	1.81753	0.00000
C	-4.29914	-0.20246	0.00000
C	5.42887	-1.00759	0.00000
C	5.54618	0.41694	-0.00000
C	-4.18356	1.23171	0.00000
H	-0.81143	-2.27220	0.00000
H	1.64727	-2.47649	0.00000
H	2.05858	2.48871	-0.00000
H	-0.40091	2.69418	0.00000
H	-3.27013	-2.07081	0.00000
H	4.11124	-2.68301	0.00000
H	4.52219	2.28630	-0.00001
H	-2.86964	2.89872	0.00000
H	6.32757	-1.61383	0.00000
H	-5.08645	1.82931	0.00000
C	-5.59822	-0.79942	0.00000
N	-6.65411	-1.27062	-0.00001
H	6.53218	0.86755	-0.00000

X = Cl E = -1152.952409 a.u. ZPE = 0.229777 a.u. G = -1152.763128 a.u.

C	-0.83314	0.84094	-0.00000
C	-0.70849	-0.60349	0.00000
C	0.57893	-1.17664	0.00001
C	-1.87962	-1.38624	0.00000
C	-2.11994	1.41345	-0.00001
C	0.33845	1.62411	-0.00000
C	1.72845	-0.39421	0.00001
C	-3.14641	-0.81198	-0.00000
C	-3.27064	0.63224	-0.00000
C	1.60365	1.04937	0.00000
C	3.03905	-0.97340	0.00001
C	-4.34340	-1.60015	-0.00000
C	-4.58427	1.20517	0.00000
C	2.80132	1.83567	0.00000
C	4.13769	-0.17033	0.00000
C	-5.57359	-1.00999	-0.00000
C	-5.69596	0.41419	0.00000
C	4.03396	1.25371	0.00000

H	0.67178	-2.25814	0.00001
H	-1.78741	-2.46802	0.00000
H	-2.21281	2.49522	-0.00001
H	0.24664	2.70587	-0.00001
H	3.14337	-2.05143	0.00001
H	-4.25077	-2.68119	-0.00001
H	-4.67701	2.28622	0.00000
H	2.71403	2.91694	0.00001
H	-6.47015	-1.61957	-0.00001
H	4.93688	1.85039	0.00001
Cl	5.74899	-0.87604	-0.00001
H	-6.68344	0.86184	0.00000

X = H (unsubstituted R) E = -693.329362 a.u. ZPE = 0.239459 a.u. G = -693.128214 a.u.

C	0.00001	-0.72507	0.00000
C	0.00000	0.72500	-0.00000
C	1.23402	1.40486	-0.00001
C	-1.23408	1.40478	-0.00001
C	-1.23401	-1.40490	0.00001
C	1.23408	-1.40487	0.00001
C	2.44716	0.72490	-0.00001
C	-2.44720	0.72477	0.00000
C	-2.44714	-0.72503	0.00001
C	2.44716	-0.72487	-0.00000
C	3.70736	1.40769	-0.00002
C	-3.70737	1.40771	-0.00000
C	-3.70740	-1.40758	0.00002
C	3.70740	-1.40764	0.00000
C	4.88277	0.71485	-0.00001
C	-4.88276	0.71489	0.00001
C	-4.88281	-0.71472	0.00001
C	4.88278	-0.71477	-0.00001
H	1.23442	2.49065	-0.00002
H	-1.23445	2.49058	-0.00001
H	-1.23440	-2.49071	0.00002
H	1.23450	-2.49068	0.00001
H	3.70718	2.49276	-0.00002
H	-3.70689	2.49275	-0.00001
H	-3.70745	-2.49269	0.00002
H	3.70724	-2.49271	0.00001
H	-5.82823	1.24560	0.00000
H	5.82825	-1.24543	-0.00000
H	5.82822	1.24552	-0.00002
H	-5.82826	-1.24543	0.00002

X = NH2 E = -748.706799 a.u. ZPE = 0.255789 a.u. G = -748.490943 a.u.

C	-0.43416	0.79381	0.00051
C	-0.35891	-0.65410	-0.00156
C	0.90760	-1.27292	-0.00373
C	-1.55936	-1.39168	-0.00173
C	-1.69788	1.41335	0.00213
C	0.76724	1.53206	0.00078
C	2.08943	-0.53714	-0.00372
C	-2.80504	-0.77158	-0.00017
C	-2.87786	0.67580	0.00189
C	2.00838	0.91060	-0.00118
C	3.37804	-1.15442	0.00021
C	-4.03050	-1.51468	-0.00063
C	-4.16926	1.29593	0.00341
C	3.23461	1.65289	-0.00161
C	4.53047	-0.40890	-0.00213
C	-5.23908	-0.88045	0.00093
C	-5.31032	0.54691	0.00295
C	4.44174	1.02668	-0.00676
H	0.95793	-2.35754	-0.00571
H	-1.50795	-2.47635	-0.00342
H	-1.75038	2.49809	0.00346
H	0.71645	2.61689	0.00221
H	3.43269	-2.23894	0.00147
H	-3.97750	-2.59855	-0.00229
H	-4.22200	2.37988	0.00479
H	3.18486	2.73679	-0.00231
H	-6.15696	-1.45773	0.00049
H	5.35922	1.60652	-0.02108
N	5.79842	-0.98689	-0.06306
H	6.55183	-0.44238	0.33011
H	5.85268	-1.96191	0.19220
H	-6.28064	1.03059	0.00401

X = OH E = -768.577793 a.u. ZPE = 0.243374 a.u. G = -768.374174 a.u.

C	-0.35234	-0.65557	-0.00000
C	-0.42372	0.79215	0.00000
C	-1.68700	1.41394	0.00001
C	0.77811	1.52861	0.00000
C	0.91332	-1.27670	-0.00000
C	-1.55332	-1.39166	-0.00000
C	-2.86759	0.67824	0.00001
C	2.01985	0.90613	-0.00000
C	2.09393	-0.54183	-0.00000
C	-2.79783	-0.76938	0.00000
C	-4.15829	1.30045	0.00001
C	3.24572	1.64967	0.00000
C	3.38484	-1.16192	-0.00001
C	-4.02426	-1.51091	0.00000
C	-5.30020	0.55319	0.00001
C	4.45472	1.02458	-0.00000
C	4.52142	-0.40389	-0.00001
C	-5.23140	-0.87444	0.00000

H	-1.73767	2.49867	0.00001
H	0.72882	2.61333	0.00001
H	0.96233	-2.36135	-0.00001
H	-1.50347	-2.47636	-0.00000
H	-4.20925	2.38447	0.00001
H	3.19437	2.73325	0.00000
H	3.44511	-2.24665	-0.00001
H	-3.97275	-2.59476	-0.00000
H	5.38397	1.58107	-0.00000
H	-6.15034	-1.45009	0.00000
O	5.78629	-0.92459	-0.00001
H	5.74260	-1.88709	-0.00001
H	-6.26989	1.03834	0.00001

X = O^{-} E = -768.034924 a.u. ZPE = 0.228961 a.u. G = -767.845744 a.u.

C	-0.30672	-0.68378	0.00004
C	-0.37852	0.77143	0.00001
C	-1.62677	1.40258	-0.00003
C	0.84029	1.49621	0.00002
C	0.94799	-1.30575	0.00007
C	-1.52917	-1.39946	0.00004
C	-2.82799	0.68469	-0.00002
C	2.07150	0.87307	0.00005
C	2.16360	-0.58788	0.00004
C	-2.76769	-0.76494	0.00000
C	-4.10502	1.31885	-0.00003
C	3.30269	1.62176	0.00004
C	3.42749	-1.19912	0.00005
C	-4.00842	-1.48467	-0.00001
C	-5.26753	0.59147	-0.00004
C	4.50567	0.99928	0.00003
C	4.65097	-0.47181	0.00004
C	-5.21246	-0.83463	-0.00003
H	-1.66348	2.48963	-0.00005
H	0.79775	2.58326	0.00000
H	0.98900	-2.39171	0.00009
H	-1.49451	-2.48587	0.00006
H	-4.13947	2.40503	-0.00006
H	3.23912	2.70804	0.00006
H	3.49269	-2.28340	-0.00000
H	-3.97247	-2.57036	-0.00000
H	5.43195	1.56722	0.00001
H	-6.13689	-1.40390	-0.00004
O	5.80083	-0.98430	-0.00020
H	-6.22983	1.09275	-0.00003
